# Integrated Proteomic and Metabolomic Analyses of Chicken Ovary Revealed the Crucial Role of Lipoprotein Lipase on Lipid Metabolism and Steroidogenesis During Sexual Maturity

**DOI:** 10.3389/fphys.2022.885030

**Published:** 2022-04-29

**Authors:** Zhifu Cui, Zifan Ning, Xun Deng, Xiaxia Du, Felix Kwame Amevor, Lingbin Liu, Xincheng Kang, Yaofu Tian, Yan Wang, Diyan Li, Xiaoling Zhao

**Affiliations:** ^1^ Farm Animal Genetic Resources Exploration and Innovation Key Laboratory of Sichuan Province, Sichuan Agricultural University, Chengdu, China; ^2^ College of Animal Science and Technology, Southwest University, Chongqing, China

**Keywords:** chicken, ovary, sexual maturity, proteome, metabolome, lipid metabolism, steroidogenesis

## Abstract

During sexual maturation and ovulatory cycle in chickens, ovaries undergo dynamic morphological and functional changes. The aim of this study was to evaluate the integrated proteome and metabolome analyses of chicken ovaries to characterize the changes in protein and metabolite profiles during sexual maturity. The ovary of Rohman layers before (125 days of age) and after (139 days of age) sexual maturation were collected for proteome and metabolome sequencing. The results showed that a total of 680 differentially expressed proteins (DEPs) and 1,046 differential metabolites (DMs) were identified in the chicken ovary during sexual maturity. Among the DEPs, 595 proteins were up-regulated and 85 were down-regulated, whereas 519 metabolites were up-regulated and 527 were down-regulated. KEGG pathway enrichment analysis showed that DEPs were significantly enriched in glycerolipid metabolism, calcium signaling pathway, folate biosynthesis, fat digestion and absorption, NF-kB signaling pathway, and PPAR signaling pathway. However, DMs were significantly enriched in the metabolism pathways, PPAR signalling pathway, glycerolipid metabolism, ferroptosis, biosynthesis of amino acids, and biosynthesis of unsaturated fatty acids. The results of the integrated analyses of DEPs and DMs revealed that the PPAR signaling pathway and glycerolipid metabolism were the most significantly enriched pathways. Among the identified DEPs, lipoprotein lipase (LPL) was upregulated in sexually mature chicken ovaries and was significantly enriched in the glycerolipid metabolism pathway, which may partially explain the possible reasons for steroidogenesis and lipid reserves responsible for oocyte maturation and ovarian follicle development during sexual maturity in chickens. The results further revealed that LPL silencing decreased the content of lipid droplets (LDs), as well as the mRNA expression of lipid metabolism-related genes including; sterol regulatory element binding protein-1 (SREBP-1) and fatty acid synthase (FASN); and steroidogenesis-related genes such as; cytochrome P450 11A1 (CYP11A1) and steroidogenic acute regulatory (StAR). The present study revealed that upregulation of LPL in the chicken ovary during sexual maturity promotes granulosa cell (GC) lipid metabolism and steroidogenesis. These findings provide a theoretical support for further studies to elucidate the mechanism of lipid metabolism to regulate the function of avian GCs during sexual maturity in chickens.

## Introduction

In female animals, the ovary is the most important organ that determines reproductive performance, and it is an ideal model for studying the biology of the ovary and follicle development in poultry. The avian ovary undergoes several dynamic morphological and functional changes during the sexual maturation and ovulatory cycles (Hrabia et al., 2011; Cui et al., 2020). Sexual maturation is associated with follicular growth and differentiation in ovaries (Onagbesan et al., 2009). These processes are regulated by several hormones (Hrabia et al., 2011; Cui et al., 2020) and growth factors (Kolesarova et al., 2010; Hrabia et al., 2011). In birds, sexual maturity is an important factor that influences female reproductive performance and egg production (Kang et al., 2012). Egg production traits are determined by ovarian function and are regulated by the hypothalamo-pituitary-gonadal (HPG) axis, which secretes specific neuropeptides or hormones to stimulate oocyte maturation and ovulation (Bédécarrats et al., 2009). Gonadotropin-releasing hormone stimulates the synthesis and secretion of pituitary gonadotropins such as follicle-stimulating hormone (FSH) and luteinizing hormone (LH) from the anterior pituitary (Sharp, 1993; Stamatiades et al., 2019). Gonadotropins play critical roles in the initiation and promotion of sexual development and the maintenance of sexual maturity (Seminara et al., 2003). FSH stimulates the ovary to regulate the growth and maturation of follicles, and promotes the secretion of estrogen in coordination with luteinizing hormone (LH) (Shoham and Schachter, 1996; Riccetti et al., 2018). During lipogenesis, estrogen stimulates the liver to synthesize large amounts of very low-density lipoprotein Y (VLDLy), which is eventually transferred to the oocytes for deposition (Perry et al., 1978; Nimpf et al., 1989; Schneider, 1996). In contrast to mammals, normal development of chicken oocytes relies on the coordinated expression and function of several members of the low-density lipoprotein receptor gene family (Schneider, 2009).

Large metabolic changes occur during sexual maturation. Zheng et al. detected transcriptional changes and identified multiple genes potentially involved in sexual maturation in female *Bactrocera dorsalis,* and reported that these differentially expressed genes were involved in reproduction and oogenesis and ovary maturation during sexual maturity (Zheng et al., 2016). miR-1b-3p is highly expressed in the chicken ovary mostly after sexual maturation, and affects egg-laying traits in chickens (Li et al., 2020). During reproductive maturation in chicken ovaries, many epigenetic changes are associated with the upregulation of the estrogen receptor alpha mRNA transcript (Guo et al., 2020). It has been reported that changes in DNA methylation in pig ovaries during sexual maturity affect the transcription of genes related to the PI3K-Akt signaling pathway, GnRH signaling pathway, and Insulin secretion (Yuan et al., 2019). Kang et al. (2013) identified gga-miR-1a and gga-miR-21 as important microRNAs responsible for follicular growth and ovulation mechanisms in sexually immature and mature chicken ovaries (Kang et al., 2013).

Until now, studies on sexual maturity have mainly focused on *Drosophila* (Hyun, 2013), *Bactrocera dorsalis* (Zheng et al., 2016), pigs (Kolesarova et al., 2015; Yuan et al., 2019), mice (Matsumoto et al., 2006; Ohlsson et al., 2020), goats (Shi et al., 2015; An et al., 2021), and cows (Tahir et al., 2019). However, there are few studies on the key regulatory proteins and metabolites in ovarian tissue during sexual maturity in chickens. In the present study, we integrated proteomic and metabolomic analyses to characterize the changes in protein and metabolism in chicken ovaries during sexual maturation.

## Materials and Methods

### Animals and Sample Collection

In this study, three hundred and six Rohman layers were fed in the poultry breeding farm of Sichuan Agricultural University (Ya’an, Sichuan Province, China). The standard management conditions, including the breeding system, environmental parameters, light program, and nutrition have been described in our previous study (Cui et al., 2020). In our recent study, we observed the morphology and histology of the ovary, as well as determined the serum biochemical parameters and the expression abundance of the critical genes from the age of 90 days (D90) to D153. The results showed that the initiation of sexual development occurred at D125 whereas first egg laying for laying hens occurred at D139. Therefore, in this study, D125 and D139 were considered as the two typical stages for sexual maturity. Nine birds each at D125 and D139 were randomly selected, blood sampled (via the wing vein), and euthanized (cervical dislocation), then the ovarian tissues were collected and immediately frozen in liquid nitrogen and later stored at −80°C for subsequent analysis.

### Protein Extraction and Quality Control

An appropriate amount of sample was weighed and transferred into a 2 ml centrifuge tube, and then 2 steel beads, 1 × Cocktail with an appropriate amount of SDS L3 and EDTA were added. After ice cooling for 5 min, DTT was added at a concentration of 10 mM; thereafter, the tissues were ground using a grinder (power 60 HZ, 2 min), and then centrifuged at 25,000 *g* × 4°C for 15 min to obtain the supernatant. Furthermore, DTT was added to the supernatant at a concentration of 10 mM and then put on water bath at 56°C for 1 h; this was proceeded by the addition of IAM at a concentration of 55 mM before keeping it in a dark room for 45 min. Then a cold acetone was added to the protein solution at a ratio of 1:5, and was temporarily stored in the refrigerator at −20°C for 30 min, followed by centrifugation at 25,000 *g* × 4°C for 15 min. The supernatant was discarded; however, the precipitate was air-dried and then lysis buffer was added without SDS L3; and was centrifuged for 15 min at 25,000 *g* × 4°C to obtain the supernatant which was considered the protein solution.

Protein extraction quality control was performed using Bradford quantification and SDS-PAGE. 1) Bradford quantification: Standard proteins (0.2 μg/μL BSA) 0, 2, 4, 6, 8, 10, 12, 14, 16, and 18 μL were sequentially added to the 96-well microtiter plates A1 to A10, then pure water 20, 18, 16, 14, 12, 10, 8, 6, 4, and 2 μL were added proceeded by the addition of Coomassie Brilliant Blue G-250 Quantitative Working Solution (180 μL) to each well. The OD 595 was measured using a microplate reader, and a linear standard curve was drawn based on the OD 595 and protein concentration. The protein solution was diluted and tested several times, then 180 μL of the quantitative working solution was added to 20 μL of the protein solution, and then read at OD 595. The sample protein concentration was calculated from the standard curve and the sample OD was 595. 2) SDS-PAGE: 10 μg each of protein solution was mixed with an appropriate amount of a loading buffer and was heated at 95°C for 5 min before centrifugation at 25,000 *g* for 5 min to obtain the supernatant, then the supernatant was loaded into a well containing 12% SDS polyacrylamide gel, and then 120 V constant pressure electrophoresis was performed for 120 min. After the electrophoresis, Coomassie blue staining was performed for 2 h, after, the solution was decolorized by adding an appropriate amount of decolorizing solution (40% ethanol, 10% acetic acid) to the shaker 3 to 5 times for 30 min each.

### Protein Enzymatic Hydrolysis and High pH RP Separation

A total of 100 μg protein solution per sample was taken and diluted with 50 mM NH_4_HCO_3_ by 4 times volumes; 2.5 μg of Trypsin enzyme was added in the ratio of protein: enzyme = 40:1, and digested for 4 h at 37°C; Trypsin was added once more in the above ratio and digestion was continued for 8 h at 37°C. Then the enzymatic peptides were desalted by drying using a Strata X column and vacuumed. 10 μg was collected per sample respectively, and mixed, furthermore, 200 μg of the mixture was diluted with 2 ml of mobile phase A (5% ACN pH 9.8) and then injection was performed subsequently. The elution peak was monitored at a wavelength of 214 nm and the components were collected every minute. The components were combined into ten fractions, which were then frozen.

### DDA and DIA Analysis by Nano-LC-MS/MS

DDA (data dependent acquisition) fractions and Data-dependent acquisition (DDA) samples were performed on a Q-Exactive HF mass spectrometer (Thermo Fisher Scientific) coupled with an Ultimate 3,000 RSLCnano system (Thermo Fisher Scientific). A nano-LC column (150 μm × 30 cm, 1.8 μm, 100 Å) was packed in-house for peptide separation at a flow rate of 500 nL/min. For DDA analysis, peptides were loaded onto a C18 trap column (300 μm × 5 mm, 5 μm, Thermo Scientific) with buffer A (2% ACN, 0.1% FA) for 5 min, then it was eluted with a gradient from 5 to 25% buffer B (98% ACN, 0.1% FA) for 155 min, 25–30% buffer B for 10 min, and 30–80% buffer B for 5 min. The mass spectrometry parameters were set as follows: MS scan range 350–1,500 m/z; loop count 30; NCE 28; MS resolution 120,000, maximal injection time (MIT) 50 ms; MS/MS HCD scans with resolution 30,000, MIT 100 ms; dynamic exclusion duration 30 s; isolation window 2.0 m/z; intensity threshold 2.0 e4; charge exclusion, excluding 1, 7, 8, >8. For DIA (data independent acquisition) analysis, the same nano-LC system and gradient were for used as DDA analysis. The DIA MS parameters were set as follows: full scan range 400–1250 m/z at resolution 120,000 with MIT 50 ms; DIA isolation window was set to 17 m/z with loop count 50 and automatic MIT, scanned at resolution 30,000; stepped NCE: 22.5, 25, and 27.5; AGC target 1e6.

### Bioinformatic Analysis

The selection of a database is an important step in MS based protein identification, and the final identified protein sequences were from the selected database. Currently, the UniProt protein database (Swiss-Prot, TrEMBL, and PIR-PSD) and protein databases are available according to genome annotation (GenBank, RefSeq, SwissProt, PDB). MaxQuant (Cox and Mann, 2008) (http://www.maxquant.org) is a software (Max Planck Institutes for high-resolution MS data) used for the identification and quantification of free proteins. In the present study, we used MaxQuant (Cox and Mann, 2008) software to identify the DDA data and served as a spectrum library for subsequent DIA analysis. In the analysis, the raw data were used as input files, and were set as corresponding parameters and databases, and identification and quantitative analyses were performed. We found that the identified peptides satisfied a false discovery rate (FDR) ≤ 0.01, hence they were used to construct the final spectral library. The DIA data were analyzed using Spectronaut (Bruderer et al., 2015), which uses iRT peptides for retention time calibration. Spectronaut™ integrates the mProphet scoring algorithm, which accurately reflects the matching level of the ion pairs. Then, based on the target-decoy model applicable to SWATH-MS, false positive control was performed with FDR 1%, therefore, obtaining significant quantitative results. MSstats (Choi et al., 2014) is an R package from the Bioconductor repository. It can be used for statistical evaluation of significant differences in proteins or peptides from different samples, and is widely used in targeted proteomics MRM, label free quantitation, and SWATH quantitative experiments. The core algorithm was a linear mixed effect model. This process was employed to preprocess the data according to the predefined comparison group, and then a significance test was performed based on the model. Thereafter, we performed differential protein screening based on a fold change ≥2 and adj_*p*-value < 0.05 as the criteria for a significant difference. Simultaneously, enrichment analysis was performed on differentially expressed proteins.

### Metabolite Extraction

After thawing the samples slowly at 4°C, weigh 25 mg and placed them in a 1.5 ml Eppendorf tube. Then, 800 μL extract (methanol: acetonitrile: Water = 2:2:1, V: V: V, −20°C precooling) +10 μL internal standard 1 + 10 μL internal standard 2, add two small steel balls, put them into the tissue grinding instrument for grinding (50 Hz, 5min), 4°C water bath ultrasounds for 10 min, then stand for 1 h in −20°C refrigerator. Centrifugation at 4°C and 25,000 RPM for 15 min. After centrifugation, take 600 μL supernatant, placed it in a cryogenic vacuum concentrator, drain it dry, add 200 μL complex solution (methanol: H_2_O = 1:9, V: V) for resolution, swirled for 1 min, then ultrasonic for 10 min in 4°C water baths, centrifugation at 4°C for 15 min at 25,000 RPM to obtain the supernatant into a sample bottle. Then, 20 μL of each sample supernatant was mixed with QC samples to evaluate the repeatability and stability of the LC-MS analysis process.

### LC-MS/MS Analysis and Database

In this study, a Waters 2D UPLC (Waters, United States) tandem Q Exactive high resolution mass spectrometer (Thermo Fisher Scientific, United States) was used for metabolite separation and detection. The chromatographic column was BEH C18 (1.7 μm 2.1*100 mm, Waters, United States). Primary and secondary mass spectrometry data were collected using a Q Exactive mass spectrometer (Thermo Fisher Scientific, United States). Databases used for the metabolites contained the BGI Library, mzCloud database, Chemspider database, human metabolome database (HMDB), Kyoto Encyclopedia of Genes and Genomes (KEGG), and Lipidmaps database.

### Cell Culture and Transfection

All pre-ovulatory follicles were dissected from the ovaries and placed in sterile Hank’s balanced salt solution. Chicken primary granulosa cells (GCs) of the follicles were harvested according to a previously described method (Gilbert et al., 1977). The granular layers were digested using *β*-II collagenase (BaiTai Biotechnology, Chengdu, China), filtered with 200 mesh cell sieves, and then resuspended in Dulbecco’s modified Eagle medium (DMEM) + 10% fetal bovine serum (Gibco, Grand Island, NY, United States) + 0.1% mixture of penicillin-streptomycin (Invitrogen, Carlsbad, CA, United States). Thereafter, the GCs were cultured in an incubator at 37°C, 5% CO_2_, and 95% air saturated humidity and the medium was changed every 24 h. When the confluence of the GCs reached 70–80%, the transfection procedure was performed with small interfering RNA (siRNA) using lipofectamine 3,000 reagent (Invitrogen, United States) according to the manufacturer’s instructions. Three small RNA interference (si-494, si-1043, and si-1056) were used to knockdown LPL expression. Oligonucleotide sequences are listed in Table 1.

**TABLE 1:** Oligonucleotide sequences of small RNA interference vector.

Name	Sense (5′-3′)	Antisense (5′-3′)
ggaLPL (si-494)	ACA​CGA​AGC​UGG​UGG​GAA​ATT	UUU​CCC​ACC​AGC​UUC​GUG​UTT
ggaLPL (si-1043)	AAG​AAA​GCC​UCU​AAA​GAA​ATT	UUU​CUU​UAG​AGG​CUU​UCU​UTT
ggaLPL (si-1056)	GCA​ACA​ACU​UGG​GUU​AUA​ATT	UUA​UAA​CCC​AAG​UUG​UUG​CTT

RNA, extraction and quantitative real time PCR (qRT-PCR).

Total RNA was isolated from the tissues and GCs using TRIzol reagent (Takara, Tokyo, Japan) according to the manufacturer’s instructions. The RNA concentration and purity were estimated by determining the A260/A280 absorbance ratio, and the 18S and 28S bands in a 1% agarose gel. Reverse transcription and qRT-PCR were performed as previously described (Cui et al., 2020). *GAPDH* was used as an endogenous control to normalize gene expression using the 2^−ΔΔCt^ method (Livak and Schmittgen, 2001). The primer sequences used in this study are summarized in Table 2.

**TABLE 2:** Primers used for qRT-PCR.

Gene	Sequence (5–3′)	Product length (bp)	Accession number
*LPL*	F: CTC​TCC​GCC​TGA​TTG​CTG​AA	274	NM_205282.2
	R: TTG​TAG​GGC​ATC​TGA​GCA​CG		
*AGK*	F: ATC​TGA​CAC​GCA​CTG​AGG​AC	228	XM_004937875.4
	R: GCT​TCA​CCT​CCA​CAG​GCA​TA		
*GLA*	F: TTT​CTA​CCT​GAG​GCC​CAT​GC	141	XM_420183.7
	R: CTG​TCC​TGG​TGA​AGT​GCT​GT		
*AGPAT2*	F: CTA​CGT​GTT​GTG​CAT​CGT​CT	113	XM_001235299.6
	R: TGA​AGG​ACT​TGA​CCA​CGG​TT		
*AKR1B1*	F: CCG​CCA​CTT​TGA​TTG​TGC​TT	292	NM_001277899.2
	R: ATC​CGT​GTT​GCC​AGG​TAT​TGA		
*SREBP1*	F: CGA​GGG​AGA​CCA​TCT​ACA​GC	158	NM_204126.3
	R: ATC​CGA​AAA​GCA​CCC​CTC​T		
*CPT1*	F: AGC​CCC​TCT​AGC​TGG​CTT​AT	191	XM_015286794.3
	R: GTG​ACG​ATA​AGG​GCA​ACC​CA		
*FASN*	F: AGC​CCA​AGT​ATT​CAG​GCA​CC	232	NM_205155.4
	R: TTC​AGG​ATG​CCC​ACA​TCA​CC		
*PPARα*	F: GGG​ATG​CTG​GTA​GCC​TAT​GG	188	NM_001001464.1
	R: AGA​CCA​GGA​CGA​TCT​CCA​CA		
*PPARγ*	F: TGT​CGT​TCG​AAC​CCC​TCA​AG	152	NM_205381.1
	R: TTG​CAG​TAA​CTC​GTC​GGG​TC		
*PPARβ*	F: ATA​ACG​CAA​TCC​GCT​TTG​GC	190	NM_204728.2
	R: CCG​GTC​AAG​ATA​CCT​CTC​GC		
*CYP11A1*	F: GAC​TAC​CGC​AAC​AAG​CCC​TA	147	NM_001001756.2
	R: CAC​AAA​GTC​CTG​GCT​CAC​CT		
*StAR*	F: CAG​AGG​GTT​GGG​AAG​GAC​AC	176	NM_204686.3
	R: ATA​AAT​CCC​TGC​TGC​TCG​GG		
*3β-HSD*	F: TTT​GGC​TAT​GTG​CCC​CGT​TA	83	NM_205118.2
	R: CTC​CTC​TGC​GGG​ACT​ATG​GA		
*GAPDH* ^*^	F: TCCTCCACCTTTGATGCG	144	NM_204305.1
	R: GTGCCTGGCTCACTCCTT		

F: forward primer; R: reverse primer. *House-keeping gene for data normalization.

### Oil Red O Staining

GC Oil red O staining was performed according to the manufacturer’s protocol. Briefly, GCs were washed in a phosphate buffered saline (PBS) and fixed with ORO Fixative for 30 min, the stationary fluid was discarded and then washed twice in PBS. Thereafter, 60% isopropanol was added and maintained for 5 min, then ORO Stain was added and retained for 20 min. Furthermore, Mayer Hematoxylin staining solution was added and incubated for 2 min after the GCs were washed 5 times. This was followed by another washing of the GCs followed by the addition of ORO Buffer and sustained for 1 min. After washing with PBS, the GCs were covered with distilled water. All sections and GCs were viewed under an electronic microscope (DP80Digital, Olympus, Tokyo, Japan) and ten fields were randomly selected for statistical analysis.

### Statistical Analysis

All data were analyzed using SPSS software (version 20.0; SPSS Inc., United States) and are presented as least squares means ± standard error mean (SEM). Unpaired Student’s t-test was used for two-group comparison analysis, and one-way analysis of variance (ANOVA) followed by Tukey’s test was used for multiple comparison analysis. The values were significantly different at *p* < 0.05.

## Results

### Comparison of Ovarian Morphological and Histological Characteristics Between Pre-laying-hens and Laying-Hens

**FIGURE 1:**
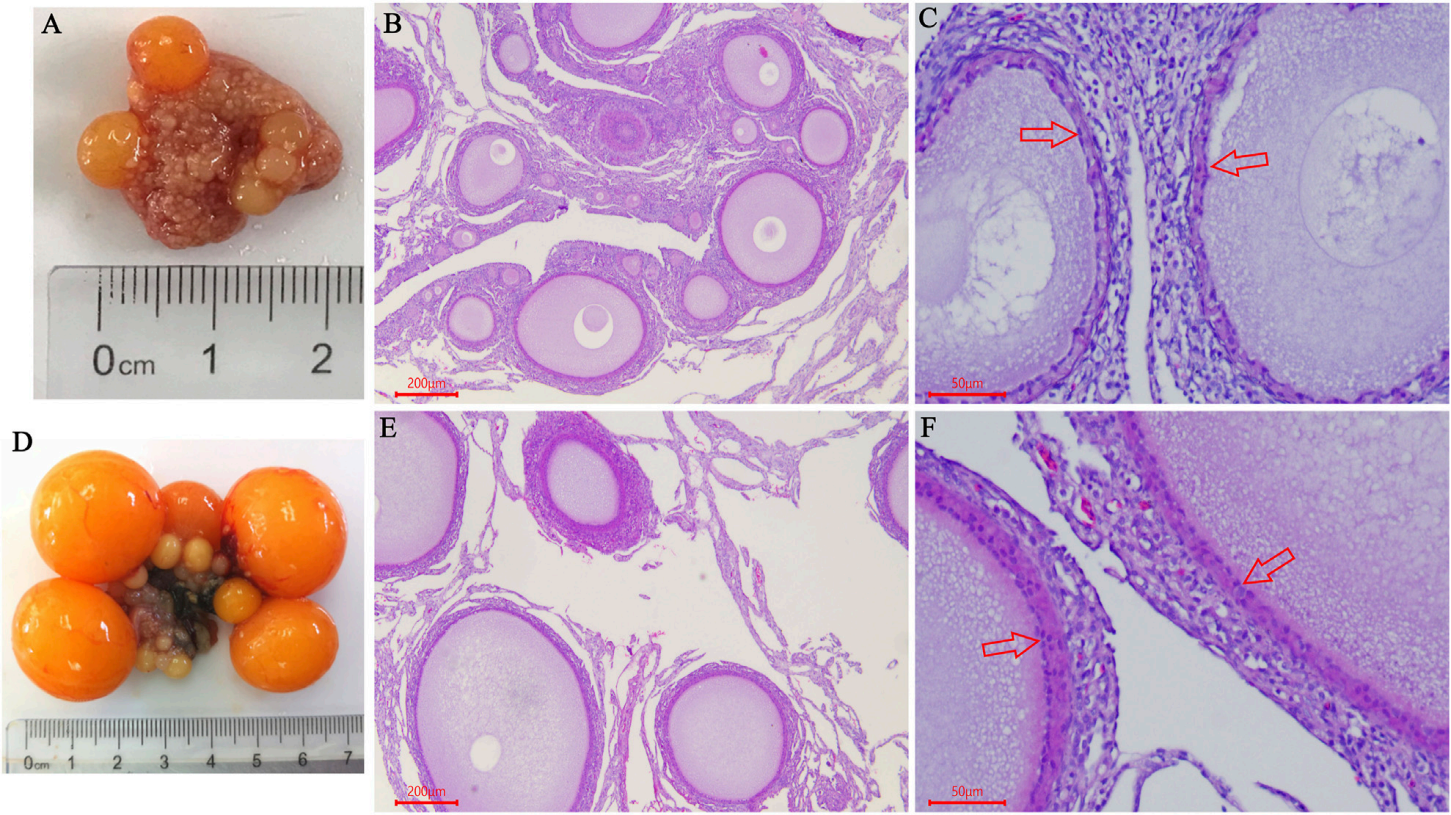
Comparison of ovarian morphological and histological characteristics between pre-laying-hens and laying-hens. **(A)** Chicken ovary on the age of D125; **(B,C)** HE staining of chicken ovary on the age of D125 (B 40×, C 200×); **(D)** Chicken ovary on the age of D139; **(E,F)** HE staining of chicken ovary on the age of D139 (B 40×, C 200×); red arrow indicates the “granulosa cells”.

In this study, the ovarian morphological and histological characteristics showed that the ovary contained a large number of primordial follicles, and white follicles (WF) and yellow follicles (YF) began to appear on D125 (Figure 1A). On D139, we observed that five hierarchical follicles (diameter >12 mm) were filled with large amounts of egg yolk deposits (Figure 1D). H&E staining showed that the ovary contained a large number of primordial follicles on D125 (Figure 1B). The follicles were filled with a small amount of lipoprotein (protein-rich white yolk) and a single layer of granulosa cells (Figure 1C). Furthermore, on D139, large yolk deposition occurred in the follicles resulting in the rapid growth and appearance of more than two layers of granulosa cells in the follicles (Figures 1E,F).

### Identification and Analysis of Differentially Expressed Proteins in the Chicken Ovary During Sexual Maturity

**FIGURE 2:**
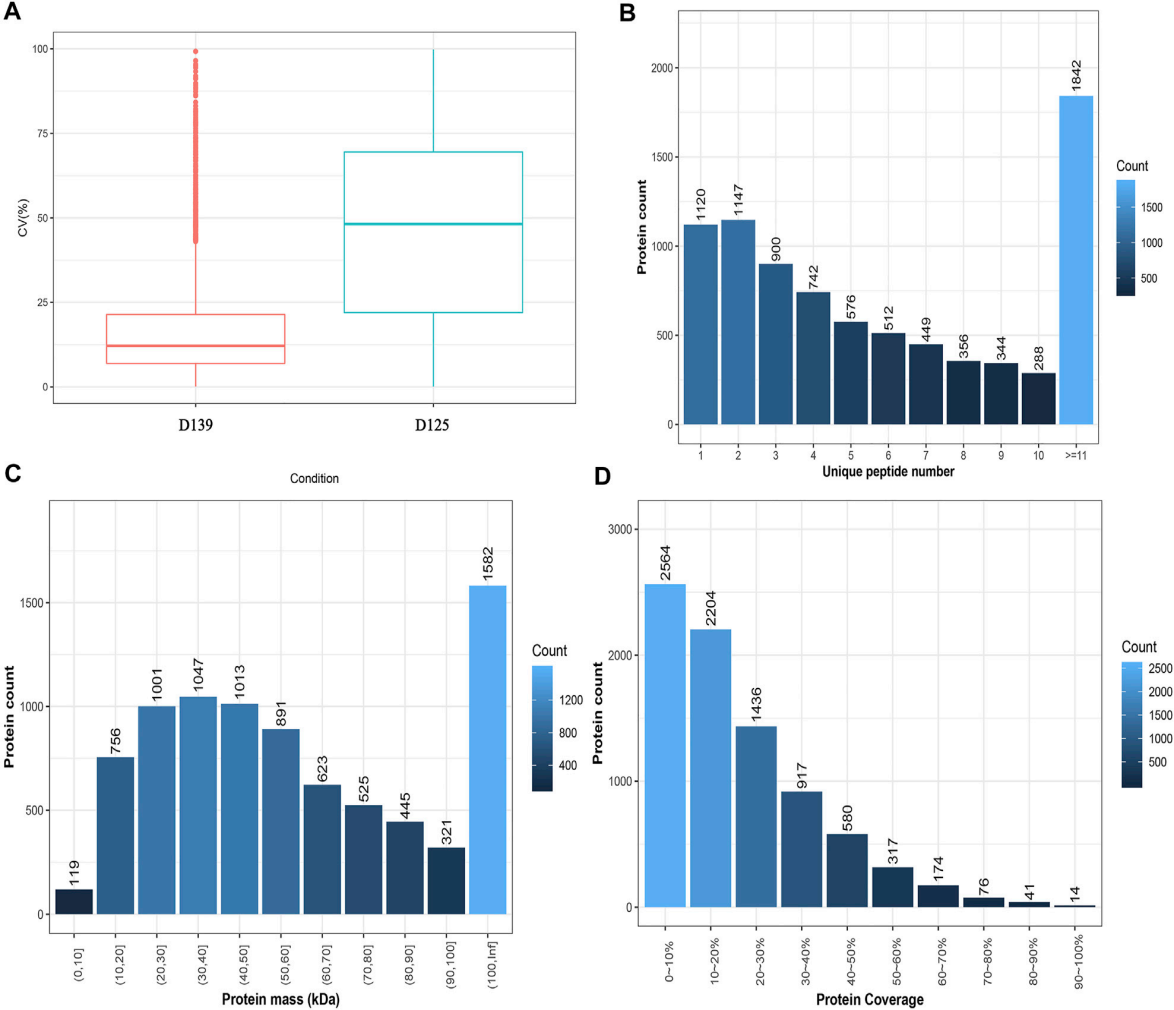
Characterization of peptides and proteins identified by DDA database. **(A)** The intra-group CV distribution of different sample groups. The *X*-axis denotes the sample group and the *Y*-axis denotes the corresponding CV. **(B)** Unique peptide distribution. The *X*-axis is the number of unique peptides for each protein, and the *Y*-axis is the number of proteins. **(C)** Protein mass distribution. The *X*-axis is protein mass interval (Kilodalton), and the *Y*-axis is the number of proteins. **(D)** Protein coverage distribution. The *X*-axis is coverage percentage interval, and the *Y*-axis is the number of proteins.

The results of the sample CV distribution are presented in Figure 2A. The number of peptides and proteins identified in the DDA database was 82,873 and 8,323, respectively. The results obtained for the unique peptide distribution showed that 1842 proteins with a peptide number ≥11 were the most numerous, whereas, those with a peptide number of 10 were represented as the least numbers (288) (Figure 2B). The protein mass distribution showed that 119 proteins with a mass between 0 and 10 kDa were recognized as the least, whereas those with a mass ≥100 kDa were noted as the highest (1,582) (Figure 2C). The results of the protein coverage distribution showed that 2,564 proteins with a protein coverage between 0 and 10% were the most numerous, whereas the lowest number of proteins 14 was found in the range of 90–100% (Figure 2D).

**FIGURE 3:**
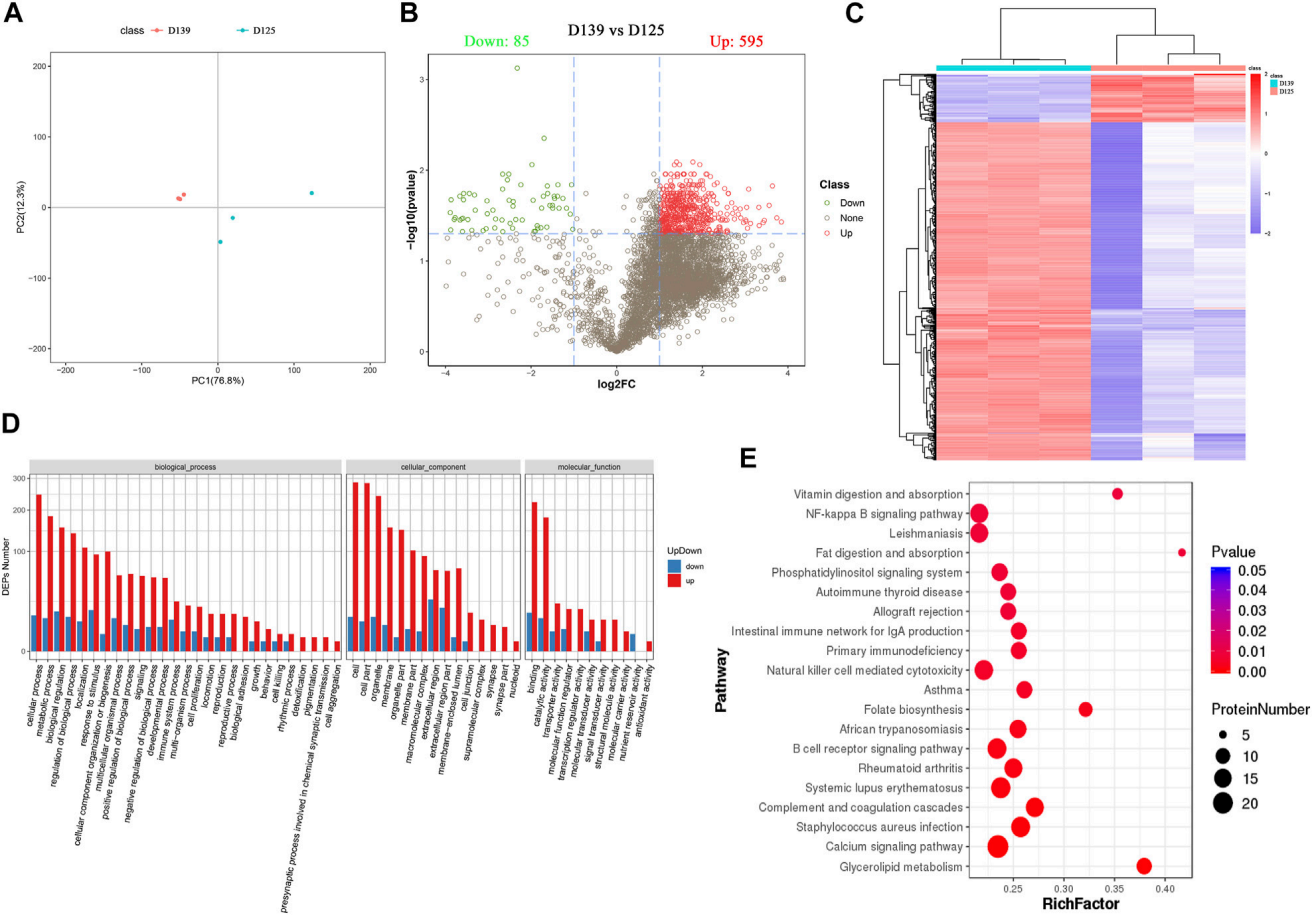
Identification and functional enrichment analyses of DEPs in chicken ovary during sexual maturity. **(A)** Principal component analysis (PCA) distribution of 6 samples. **(B)** 680 DEPs (595 upregulated and 85 downregulated) were presented in a volcano plot. **(C)** The hierarchical cluster analysis of DEPs. **(D)** The significance terms of biological process, cellular component, and molecular function; red and blue show up- and down-regulation in the sexually mature (D139) and immature chicken ovaries (D125), respectively. **(E)** Top 20 significantly changed pathways associated with DEPs.

In addition, the principal component analysis (PCA) showed a trend of separation between the groups in the experimental model, and there were no exceptional value points (Figure 3A). We identified 680 differentially expressed proteins (DEPs) in the chicken ovary during sexual maturity, including 595 proteins that were up-regulated and 85 down-regulated. Sub-cellular localization of proteins is an essential part of protein functional annotation, and only those proteins transported to the right place can participate in cellular activities. Sub-cellular localization of DEPs was performed using the bioinformatics tool WoLF PSORT (Horton et al., 2007). Detailed results are presented in Supplementary Figure S1, the DEPs are presented in a volcano plot (Figure 3B), and the hierarchical cluster analysis is shown as heatmaps (Figure 3C). The log2 conversion and Z-Score standardization were performed on the data during cluster analysis. Hierarchical clustering was adopted in the clustering algorithm, and the Euclidean distance was used in the distance calculation.

The results obtained from GO and KEGG analyses showed that DEPs were involved in several biological processes (BPs) including cellular processes, metabolic processes, biological regulation, regulation of biological processes, localization, response to stimulus, cellular component organismal processes, and multicellular organismal processes. The DEPs were involved in cellular components (CCs) such as cell, cell part, organelle, membrane, organelle part, membrane part, macromolecular complex, and extracellular region, whereas the DEPs were involved in molecular functions (MF) such as binding, catalytic activity, transporter activity, molecular function regulator, transcription regulator activity, molecular transducer activity, signal transducer activity, and structural molecule activity (Figure 3D) (Supplementary Table S1). KEGG pathway enrichment analysis showed that DEPs were significantly enriched in Glycerolipid metabolism, Calcium signaling pathway, Complement and coagulation cascades, B cell receptor signaling pathway, Folate biosynthesis, natural killer cell mediated cytotoxicity, primary immunodeficiency, fat digestion and absorption, NF-kappa B signaling pathway, and vitamin digestion and absorption (Figure 3E) (Supplementary Table S2). Pathway relationship networks are shown in Supplementary Figure S2. The number of DEPs enriched in the glycerolipid metabolism pathway were the highest, and contained 11 DEPs (LPL, TKFC, AGK, GLA, AGPAT2, AKR1B10L, AKR1B1L, ACBD6, AKR1B1, AKR, and AKR1E2). Among the DEPs, five (AKR1B10L, AKR1B1L, AKR1B1, AKR, and AKR1E2) were significantly enriched in the folate biosynthesis pathway (Table 3).

**TABLE 3:** Pathway relationship networks of DEPs enriched in the glycerolipid metabolism pathway and folate biosynthesis pathway.

Pathway	Protein_ID	Name	Description	log2Foldchange	Up/Down
Glycerolipid metabolism	sp|P11602|LIPL_CHICK	LPL	Lipoprotein lipase	1.271309075	up
Glycerolipid metabolism	tr|A0A1D5PG53|A0A1D5PG53_CHICK	TKFC	Triokinase and FMN cyclase	2.149241666	up
Glycerolipid metabolism	tr|A0A1L1RSS5|A0A1L1RSS5_CHICK	AGK	Acylglycerol kinase	1.143155235	up
Glycerolipid metabolism	tr|E1BT44|E1BT44_CHICK	GLA	Alpha-galactosidase	1.432224806	up
Glycerolipid metabolism	tr|Q90W83|Q90W83_CHICK	AKR	Aldo-keto reductase	2.578780418	up
Glycerolipid metabolism	tr|E1BVD1|E1BVD1_CHICK	AKR1B10L	Aldo-keto reductase family 1, member B10-like	3.053645346	up
Glycerolipid metabolism	tr|E1C1I6|E1C1I6_CHICK	AKR1B1L	Aldo-keto reductase family 1, member B1-like	2.42793748	up
Glycerolipid metabolism	tr|F1NT57|F1NT57_CHICK	AKR1B1	Aldo-keto reductase family 1, member B10	2.838862097	up
Glycerolipid metabolism	tr|R4GG24|R4GG24_CHICK	AKR1E2	Aldo-keto reductase family 1, member E2	3.01846642	up
Glycerolipid metabolism	tr|E1C483|E1C483_CHICK	ACBD6	Acyl-CoA binding domain containing 6	1.301430877	up
Glycerolipid metabolism	tr|E1BTF7|E1BTF7_CHICK	AGPAT2	1-acyl-sn-glycerol-3-phosphate acyltransferase	1.810838371	up
Folate biosynthesis	tr|A0A1D5PF81|A0A1D5PF81_CHICK	RMDN1	Regulator of microtubule dynamics 1	1.60485872	up
Folate biosynthesis	tr|A0A1D5PM44|A0A1D5PM44_CHICK	RMDN3	Regulator of microtubule dynamics 3	1.054522594	up
Folate biosynthesis	tr|A0A2Z5EM90|A0A2Z5EM90_CHICK	NADB-LER5	NADB-Rossmann superfamily	1.136685305	up
Folate biosynthesis	tr|Q90W83|Q90W83_CHICK	AKR	Aldo-keto reductase	2.578780418	up
Folate biosynthesis	tr|F1NT57|F1NT57_CHICK	AKR1B1	Aldo-keto reductase family 1, member B10	2.838862097	up
Folate biosynthesis	tr|E1BVD1|E1BVD1_CHICK	AKR1B10L	Aldo-keto reductase family 1, member B10-like	3.053645346	up
Folate biosynthesis	tr|E1C1I6|E1C1I6_CHICK	AKR1B1L	Aldo-keto reductase family 1, member B1-like	2.42793748	up
Folate biosynthesis	tr|R4GG24|R4GG24_CHICK	AKR1E2	Aldo-keto reductase family 1, member E2	3.01846642	up
Folate biosynthesis	tr|F1N8Y3|F1N8Y3_CHICK	CBR3	Carbonyl reductase 3	2.225924617	up

### Identification and Analysis of Differential Metabolites in the Chicken Ovary During Sexual Maturity

The base peak chromatogram of all quality control (QC) samples showed good spectral overlap, small fluctuations in retention time and peak response intensity, indicating that the instruments used were in good condition and showed a stable signal during the entire process of sample detection and analysis (Supplementary Figures S3A, S4A). The relative peak areas of all QC samples with a coefficient of variation (CV) > 30% were removed, whereas the CV distribution of the compounds in each group of the samples showed that the data quality was acceptable (Supplementary Figures S3B, S4B). The partial least square method-discriminant analysis (PLS-DA) model were performed in response to the permutation testing, and the results were showed in Supplementary Figures S3C, S4C. A total of 1,046 differential metabolites (DMs) were detected in the positive ion detection mode, including 519 up-regulated metabolites, 527 down-regulated metabolites, and 341 differential metabolites with identified information. 239 DMs were detected in the negative ion mode, including 119 up-regulated metabolites, 120 down-regulated metabolites, and 97 DMs with identified information (Table 4) (Supplementary Table S3).

**TABLE 4:** Statistics of differential metabolites.

Mode	Group	Total number of DEs	Number of DEs with identified information	Up	Down
Positive	D139_D125	1,046	341	519	527
Negative	D139_D125	239	97	119	120

**FIGURE 4:**
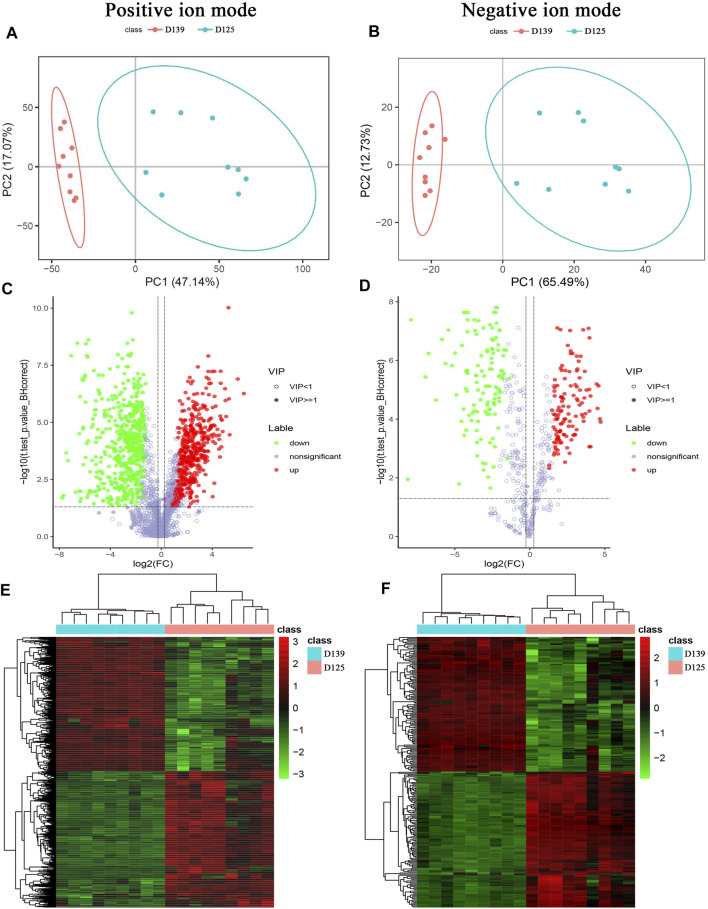
Characterization of differential metabolites (DMs) identified by positive and negative ion mode between sexually mature and immature chicken ovaries. **(A,B)** PCA distribution of 18 samples. **(C,D)** DMs identified by positive and negative ion mode were presented in a volcano plot. **(E,F)** The hierarchical cluster analysis of DMs.

**FIGURE 5:**
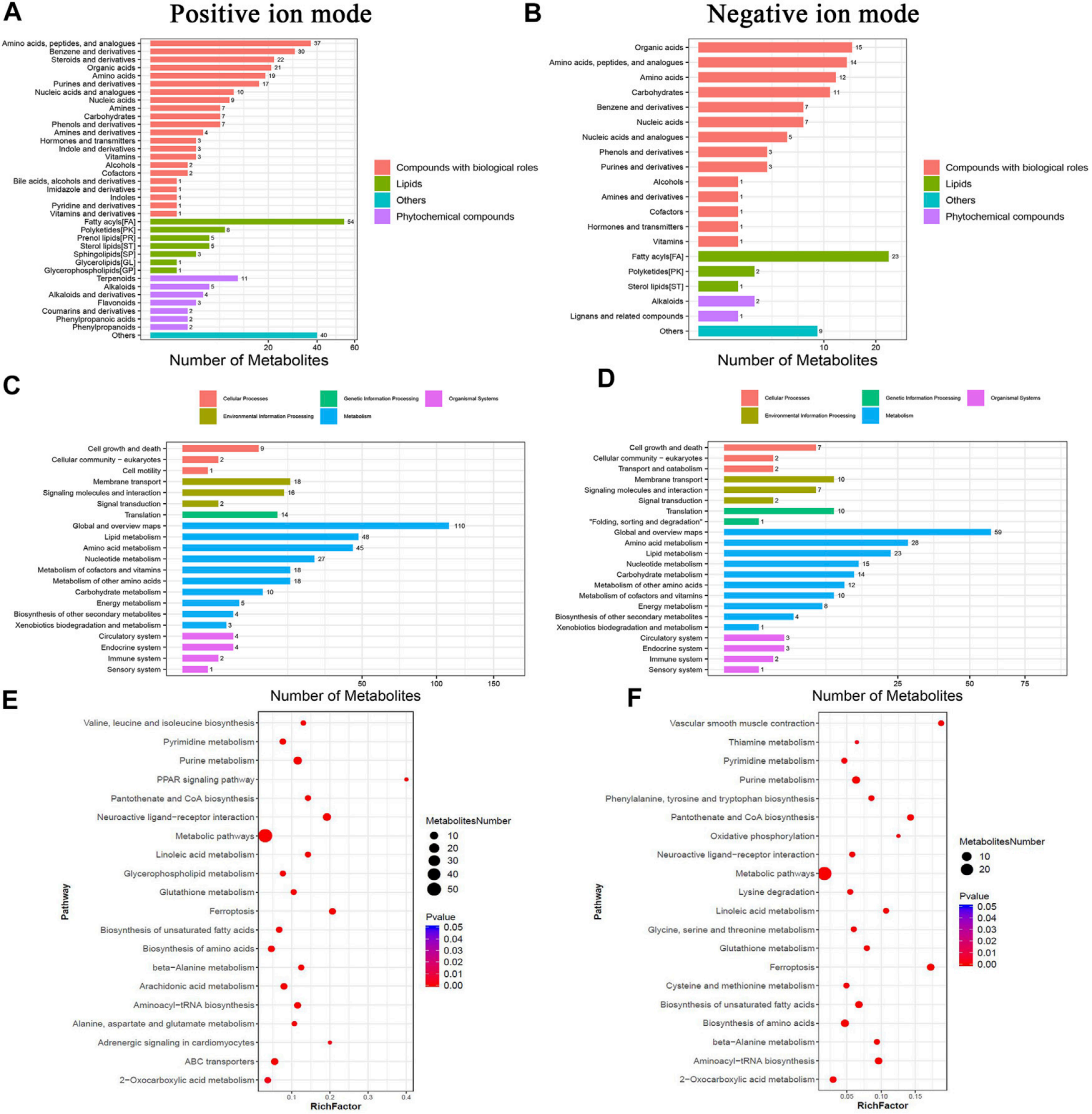
Functional enrichment analyses of DMs in chicken ovary during sexual maturity. Based on KEGG database, functional annotations were performed for DMs identified by positive and negative ion mode. **(A,B)** pathway annotation histogram, **(C,D)** DMs pathway classification, **(E,F)** significantly enriched pathway.

The principal component analysis (PCA) was performed to observe the trend of distribution and separation among the samples and the results were showed in Figures 4A,B. DMs were visualized through volcano plot (Figures 4C,D) and hierarchical cluster analysis was dotted as heatmaps (Figures 4E,F). Refer to KEGG and HMDB databases to annotate the classification of DMs to understand the classification of these metabolites (Figures 5A,B). Functional annotation of the DMs was conducted through KEGG database to determine the major biochemical metabolic pathways and signal transduction pathways involved in the metabolites (Figures 5C,D). The identification of DMs without identified information was not counted. Moreover, the metabolic pathway enrichment analysis of DMs was performed based on the KEGG database, and the significant differential metabolite enrichment was set at *p*-value < 0.05. We found that the DMs were significantly enriched in the metabolism pathways, PPAR signaling pathway, glycerophospholipid metabolism, ferroptosis, biosynthesis of amino acids, biosynthesis of unsaturated fatty acids, pyrimidine metabolism, purine metabolism, linoleic acid metabolism, and ABC transporters (Figure 5E). The DMs identified by negative ion mode were significantly enriched in metabolism pathways, ferroptosis, purine metabolism, biosynthesis of amino acids, biosynthesis of unsaturated fatty acids, oxidative phosphorylation, glycerophospholipid metabolism, FoxO signaling pathway, and GnRH signaling pathway (Figure 5F).

### Integrative Analyses Between Differential Proteins and Metabolites in the Ovarian Tissue During Sexual Maturity

**FIGURE 6:**
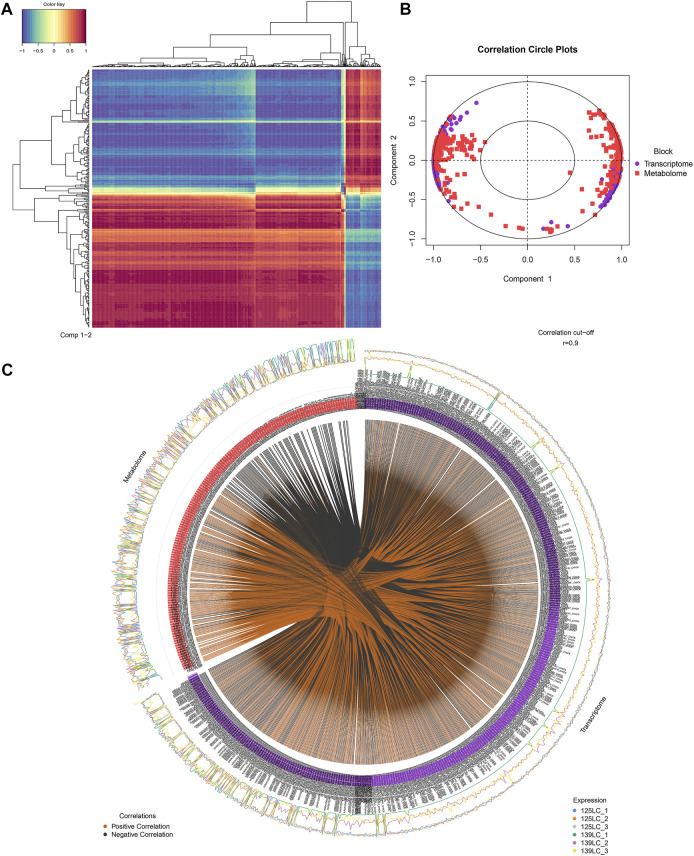
Integrative analyses between DEPs and DMs in ovary tissues during sexual maturity. **(A)** The correlation cluster heat map between DEPs and DMs. **(B)** The concentric circles of correlation between DEPs and DMs. **(C)** The circos diagram of the correlation between DEPs and DMs.

The DEPs and DMs of the ovarian tissue were analyzed based on the rCCA correlation analysis to measure the degree of association between the genes and metabolites. Figure 6A showed the correlation cluster heat map. Each row represents a DM, each column represents a DEP, the blue colors represents negative correlation, and red represents positive correlation. Moreover, the mixOmics package provides many functions for multi-variable dimensionality in reduction of omics and statistical integration of the multiple data sets to ensure relationships among omics. The block. splsda function in the mixOmics package was used to analyze DEPs and DMs, and the results were visualized by plotVar and circosPlot functions, respectively. The circos diagram of the correlation between DEPs and DMs was presented in Figure 6C. The line in the circle showed that the correlation coefficient between the DEPs and DMs was greater than or equal to 0.9, whereas the outer blue and orange curves represent the expression of DEPs and DMs between the two groups of samples.

Figure 6B showed the concentric circles of the correlation between DEPs and DMs. Each dot in the circle represents a gene, and each square represents a metabolite. The relationship between the DEPs and DMs was determined by projecting the first and second principal components. The presence of an acute angle between the DEPs and DMs, showed that the correlation is positive, but when there was a ton angle between the DEPs and DMs, then the correlation was negative. From the center of the circle, the line connects DEPs and DMs, hence, the longer the line length, the stronger the relationship, and vice versa. Therefore, in general, variables that are far from the center of the circle were highly correlated.

Furthermore, the integrated KEGG pathway analyses for DEPs and DMs showed that 4 DMs (O-phosphorylethanolamine, Citicoline, Acetylcholine, and Cdp-ethanolamine) and 2 DMs (Ethanolamine phosphate and P-dmea) identified by positive and negative ion mode respectively, and 11 DEPs (LPL, TKFC, AGK, GLA, AGPAT2, AKR1B10L, AKR1B1L, ACBD6, AKR1B1, AKR, and AKR1E2) were significantly enriched in glycerolipid metabolism pathway. These results collectively indicated that these 11 DEPs and 6 DMs enriched in glycerolipid metabolism pathway were highly correlated in chicken ovarian during sexual maturity.

### LPL Regulate GC Lipid Metabolism and Steroidogenesis During Sexual Maturity

**FIGURE 7:**
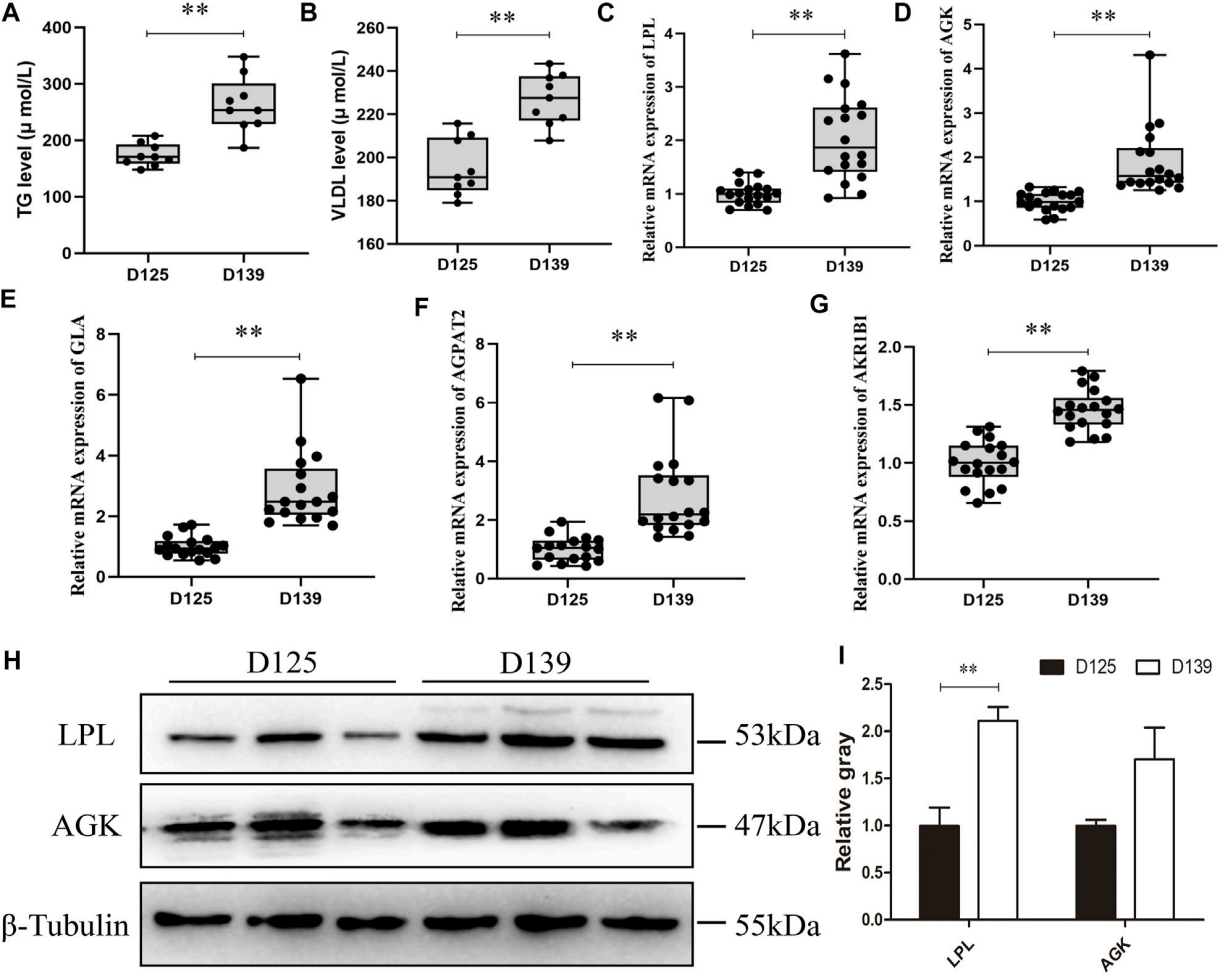
The validation results of the DEPs enriched in the glycerolipid metabolism pathway. **(A,B)** The concentration of TG and VLDL in chicken serum was detected in D125 and D139. **(C–G)** qRT-PCR and **(H,I)** western blot were performed to validate the DEPs enriched in glycerolipid metabolism pathway. Data are presented as mean ± standard error (SE); *p < 0.05 and **p < 0.01.

In comparism with D125, the concentration levels of TG and VLDL in the chicken serum were significantly increased on D139 (Figures 7A,B). Subsequently, we validated the DEPs enrichment in the glycerolipid metabolism pathway by qRT-PCR. The mRNA expression of lipoprotein lipase (LPL), acylglycerol kinase (AGK), galactosidase alpha (GLA), 1-acylglycerol-3-phosphate-O-acyltransferase 2 (AGPAT2), and aldo-keto reductase family 1-member B1 (AKR1B1) in sexually mature (D139) chicken ovaries were significantly upregulated as compared with the immature chicken ovaries (D125) (Figures 7C–G). The protein expression of LPL at D139 were significantly higher than that at D125 (Figures 7H,I), whereas there was no significant difference of the AGK protein expression between D139 and D125. These results were consistent with the proteome sequencing data. LPL is considered a key enzyme responsible for catalyzing lipid metabolism and balance, as well as transfer of TG from VLDL into the ovary, to promote their catabolism by LPL. Thus, we further explored the biological significance of the LPL on lipid metabolism of GCs during sexual maturity.

**FIGURE 8:**
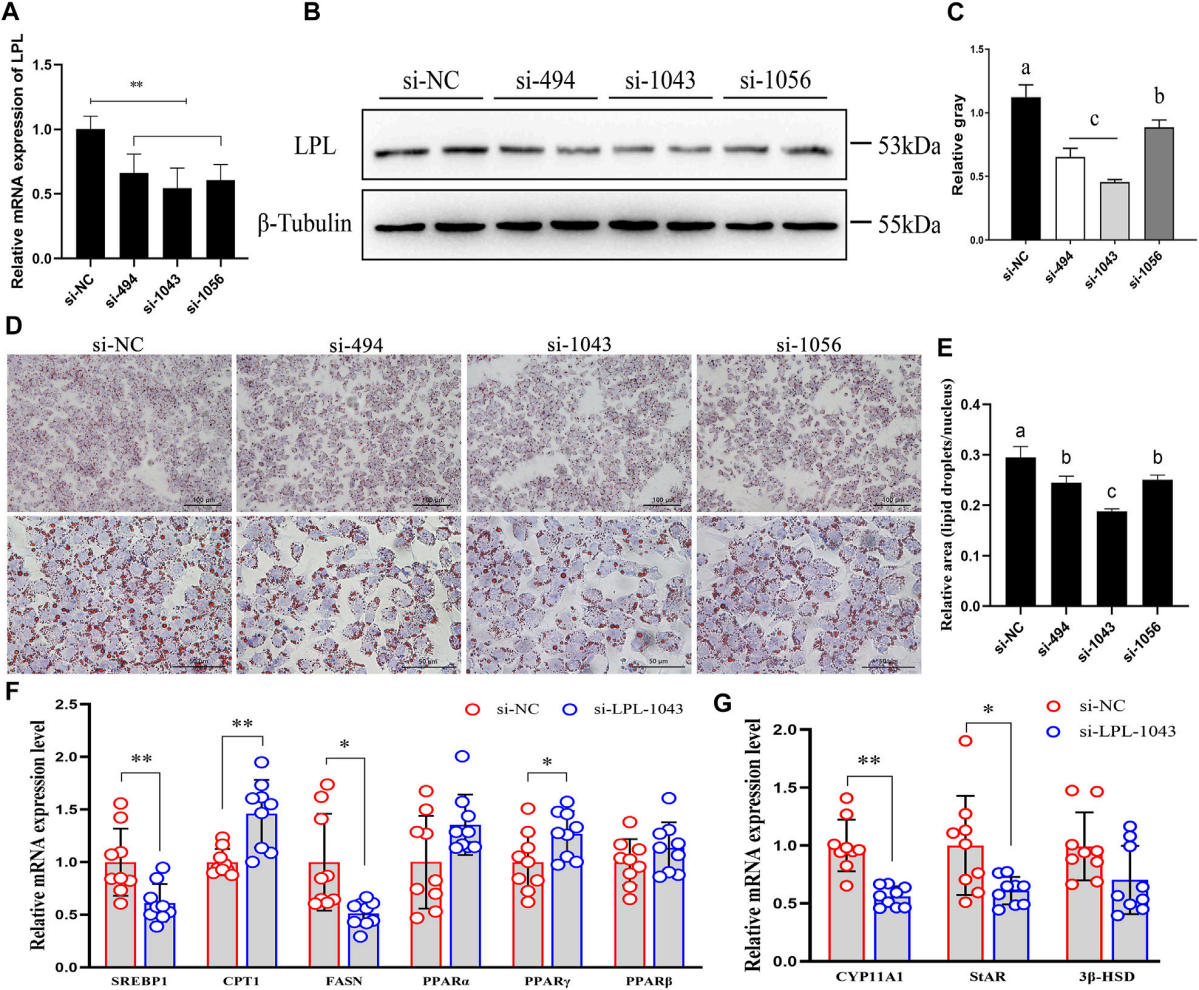
LPL regulate granulosa cell lipid metabolism and steroidogenesis during sexual maturity. **(A–C)** Three small RNA interference (si-494, si-1043, and si-1056) were used to knockdown the mRNA and protein expression of LPL. **(D,E)** After transfection of siRNAs, lipid droplets (LDs) accumulation in granulosa cells (GCs) was detected by oil red O staining (magnified 200× and 600×). **(F,G)** LPL knockdown regulated the mRNA expression of lipid metabolism- and steroidogenesis-related genes. Data are presented as mean ± standard error (SE); *p < 0.05 and **p < 0.01. Different lowercase letters indicate significant differences among the groups (*p* < 0.05).

Three small RNA interference (si-494, si-1043, and si-1056) were used to knockdown the expression of LPL. Compared to the negative control (si-NC), si-RNAs significantly decreased the mRNA and protein expression of LPL (Figures 8A–C). Moreover, after LPL interference, the lipid droplets accumulation in the GCs was decreased (Figures 8D,E), the mRNA expression levels of the lipid metabolism-related genes such as sterol regulatory element binding protein-1 (SREBP-1) (*p* < 0.01) and fatty acid synthase (FASN) (*p* < 0.05) were significantly decreased, whereas the expression levels of carnitine palmitoyltransferase 1 (CPT-1) (*p* < 0.01) and perixisome proliferation activated receptor gamma (PPARγ) (*p* < 0.05) were significantly increased, and no significant effect was detected in the expression levels of PPARα and PPARβ (*p* > 0.05) (Figure 8F). Moreover, the expression levels of steroidogenesis-related genes including recombinant cytochrome P450 11A1 (CYP11A1) (*p* < 0.01) and steroidogenic acute regulatory (StAR) (*p* < 0.05) were significantly decreased, whereas 3β-hydroxysteroid dehydrogenase (3β-HSD) was not significantly affected (*p* > 0.05) (Figure 8G). These results revealed that upregulation of LPL in chicken’s ovary during sexual maturity may play a vital role on GC lipid metabolism and steroidogenesis.

## Discussion

Sexual maturity in poultry is an essential factor that influences the female reproductive performance and egg production. In addition, a major important indicator of sexually matured laying breeder hens is age at first egg (AFE) (Kang et al., 2012). Sexual maturity in breeder hens indicate the commencement of egg laying, since it has significant effects on ovarian follicle development (Shit et al., 2014). There is high concentration of estrogen in the sexually matured breeder hens, which regulate the synthesis of yolk precursors in the liver via vitellogenesis and deposition of these yolk precursors into the oocyte of the ovary (Schneider et al., 1998), as well as an increase in the levels of gonadal steroid hormone and the promotion of follicular growth through receptor-mediated mechanism (McElroy et al., 2004).

Previous studies have reported on several microRNAs (Kang et al., 2013) and candidate genes (Guo et al., 2018) associated with sexual maturation of chicken’s ovary, however, studies on the functional proteins and metabolite profiles during ovarian development and folliculogenesis have not yet been elucidated. Therefore, in this study, we found significant changes in the protein and metabolite profile between sexually matured (D139) and immatured (D125) chicken ovaries. We found that hen egg protein 21 kDa (HEP21) was one of the DEPs between sexually matured and immatured chicken ovaries and was significantly enriched in reproduction (GO terms). This result was consistent with the study by Chen et al. (2020) which reported HEP21 as a candidate gene responsible for sexual maturation in chickens (Chen B. et al., 2020). In the present study, we found that chaperonin-containing tailless complex polypeptide 1 subunit 6A (CCT6A) and steroid receptor coactivator (SRC) were differentially expressed during sexual maturity in laying hens, and was related to the developmental process. Kang et al. identified differentially expressed genes in the ovary of sexually matured chickens at different ages, and found that CCT6A expression level was consistent with egg production performance, indicating that CCT6A may play an important role in the process of sexual maturation of hens (Kang et al., 2012). Liao et al. (2002) indicated that SRC played important roles in sexual maturation and reproductive function in female mice (Liao et al., 2002). Forkhead box L2 (FOXL2), a forkhead transcription factor essential for proper reproductive function in females, is preferentially expressed in the ovary (Uhlenhaut and Treier, 2006; Georges et al., 2014). FOXL2 is a highly conserved gene that is required for granulosa cell function and is also involved in ovary development (Uhlenhaut and Treier, 2006). In the present study, we found that the DEPs FOXL2 in sexually matured and immatured chicken ovaries was significantly enriched in the developmental process, growth, and reproduction (GO terms).

Compared to the protein abundance in the immatured chicken ovaries, the DEPs in the sexually matured ovaries were significantly enriched in the glycerolipid metabolism, fat digestion and absorption, and folate biosynthesis. The DMs were significantly enriched in glycerophospholipid metabolism, PPAR signaling pathway, ferroptosis, biosynthesis of amino acids, and biosynthesis of unsaturated fatty acids. It was reported that glycerolipid metabolism defines the follicular fluid of infertility patients (Batushansky et al., 2020), glycerolipid and glycerophospholipid metabolism were related to polycystic ovary syndrome (PCOS) patients (Liu et al., 2019), ovarian transcriptome in female Portunus trituberculatus revealed the regulation of fat digestion and absorption and PPAR signaling pathway on the gonadal development (Zhou et al., 2019), and PPAR signaling pathway was involved in ovarian follicle development (Dupont et al., 2012). Folate was reported to improve the symptom and infertility of patients with PCOS (Szczuko et al., 2016; Regidor et al., 2018). Zhu et al. showed that in fishes, there is more active amino acids, lipid metabolism, and energy dynamics in response to high energy input during ovary development (Zhu et al., 2020). Moreover, unsaturated fatty acids have an influence on cytoplasmic maturation of oocytes from prepubertal gilts (Pawlak et al., 2012).

In avian species, lipids and especially triglycerides may be stored in the growing oocytes, which play important role in vitellogenesis, as well as embryo development. Triglycerides are synthesized mainly by the liver and is transported by lipoproteins (apoB and apo-VLDL-II in matured laying hens), whereas the VLDL (the assembly of triglycerides and lipoproteins) transports the triglycerides into the oocyte of the chicken’s ovary (Hermier, 1997). Lipoprotein lipase (LPL) is a key enzyme responsible for catalyzing lipid metabolism and balance, and the extrahepatic rate-limiting enzyme in the hydrolysis of circulating triacylglycerides (Enerbäck and Gimble, 1993). The transfer of triglycerides in the form of VLDL into the ovarian tissue involves their catabolism by LPL. LPL catalyzes the hydrolysis of triglycerides to fatty acids (FAs) and glycerol (Hermier, 1997). LPL is highly expressed in the ovary of chickens (Benson et al., 1975; Schneider et al., 1990) and in fishes (Ryu et al., 2013; Akhavan et al., 2020), were predominantly expressed in the granulosa cells of the ovarian follicles. The activity of the granulosa cell LPL begins to increase as more lipids are deposited into the follicles. Granulosa cell LPL provides the follicular tissues with the requisite enzymatic system to hydrolyze very low-density lipoprotein triglyceride and generate free FAs for uptake and biosynthesis of neutral lipids by the growing oocytes (Benson et al., 1975; Ryu et al., 2013). The results in this study showed that lipoprotein lipase (LPL) was upregulated in the sexually matured chicken ovaries and was significantly enriched in the glycerolipid metabolism pathway. We validated the proteome sequencing data and the results showed that the expression of LPL in the sexually matured chicken ovaries were significantly increased as compared with the immatured chicken ovaries. LPL was found to be highly expressed and active in the ovary of the European sea bass during gonadal development and play crucial roles in steroidogenesis and transportation of neutral lipids into the oocytes (José Ibáñez et al., 2008). Ovarian LPL is directly involved in the uptake of lipids into the ovary of eel. Ovarian LPL transcript abundance increased as oogenesis increase, and it increases rapidly during midvitellogenesis, corresponding to pronounced increase in the ovarian lipid deposits and LPL activity (Divers et al., 2010).

In addition, inhibition of LPL caused a reduction in the lipid droplets (LDs) and the mRNA expression of lipid metabolism-related genes such as SREBP-1 and FASN. SREBP-1 and FASN are key enzymes in *de novo* synthesis of fatty acid (Horton et al., 2002; Wang et al., 2004). LDs, stored as neutral lipids, are responsible for several physiological processes including follicle development which serve as a source of energy for oocyte maturation (Sturmey et al., 2006; Silva et al., 2011). As the ovarian follicle grows, LDs contents gradually increases the follicular GCs (Gao et al., 2019). We found in this study that the mRNA expression of steroidogenesis-related genes such as CYP11A1 and StAR were significantly decreased. CYP11A1 and StAR are key genes that promote the synthesis of steroid hormones (Sewer and Li, 2008). These results were consistent with the previous study in goose GCs that showed that FASN-mediated lipid metabolism regulated steroidogenesis through the transcription of StAR and CYP11A1 (Chen X. et al., 2020). The results obtained in this study revealed that the upregulation of LPL in the chicken ovary during sexual maturity may play a vital role on GC lipid metabolism and steroidogenesis.

## Conclusion

In summary, the present study was carried out to investigate the integration of proteomics and metabolomics analysis of chicken ovary during sexual maturity. Results indicated that PPAR signaling pathway and glycerolipid metabolism were significantly enriched pathways. Lipoprotein lipase (LPL) as one of the differentially expressed proteins was found to be upregulated in the sexually matured chicken ovaries and significantly enriched in the glycerolipid metabolism pathway, which may partially interpret the steroidogenesis and lipid reserves for oocyte maturation in the ovarian follicle development during sexually maturity in chickens. In addition, knockdown of LPL decreased the content lipid droplets (LDs) of GCs, as well as the mRNA expression of the lipid metabolism-related gene SREBP-1 and FASN, and steroidogenesis-related gene CYP11A1 and StAR. Generally, our results revealed that the upregulation of LPL in the chicken ovary during sexual maturity promoted the lipid metabolism and steroidogenesis of the GC, which provided a theoretical basis for further studies on the mechanisms of lipid metabolism in avian GCs during chicken sexual maturity.

## Data Availability

The data supporting the conclusions of this article will be made available by the authors, without undue reservation.
