# Supplementary material for: Integrated Proteomic and Metabolomic Analyses of Chicken Ovary Revealed the Crucial Role of Lipoprotein Lipase on Lipid Metabolism and Steroidogenesis During Sexual Maturity
Source: Front Physiol. 2022 Apr 29;13:885030. doi: 10.3389/fphys.2022.885030 (PMC9099287; doi:10.3389/fphys.2022.885030)
Supplement: Supplementary file 1 [file Table1.DOCX]

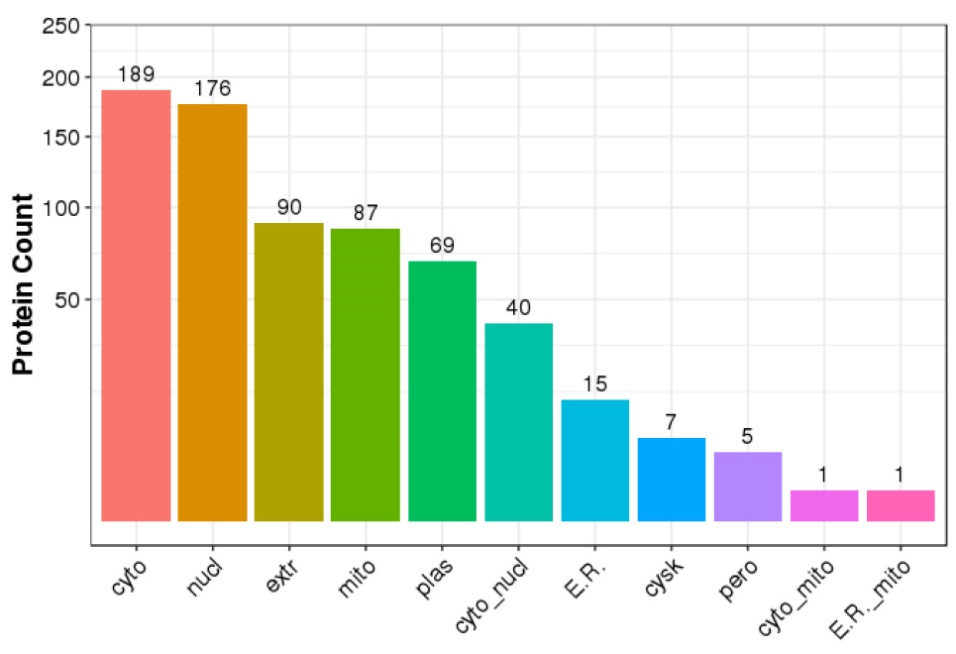


**Figure S1.** Subcellular localization of differentially expressed proteins (DEPs).


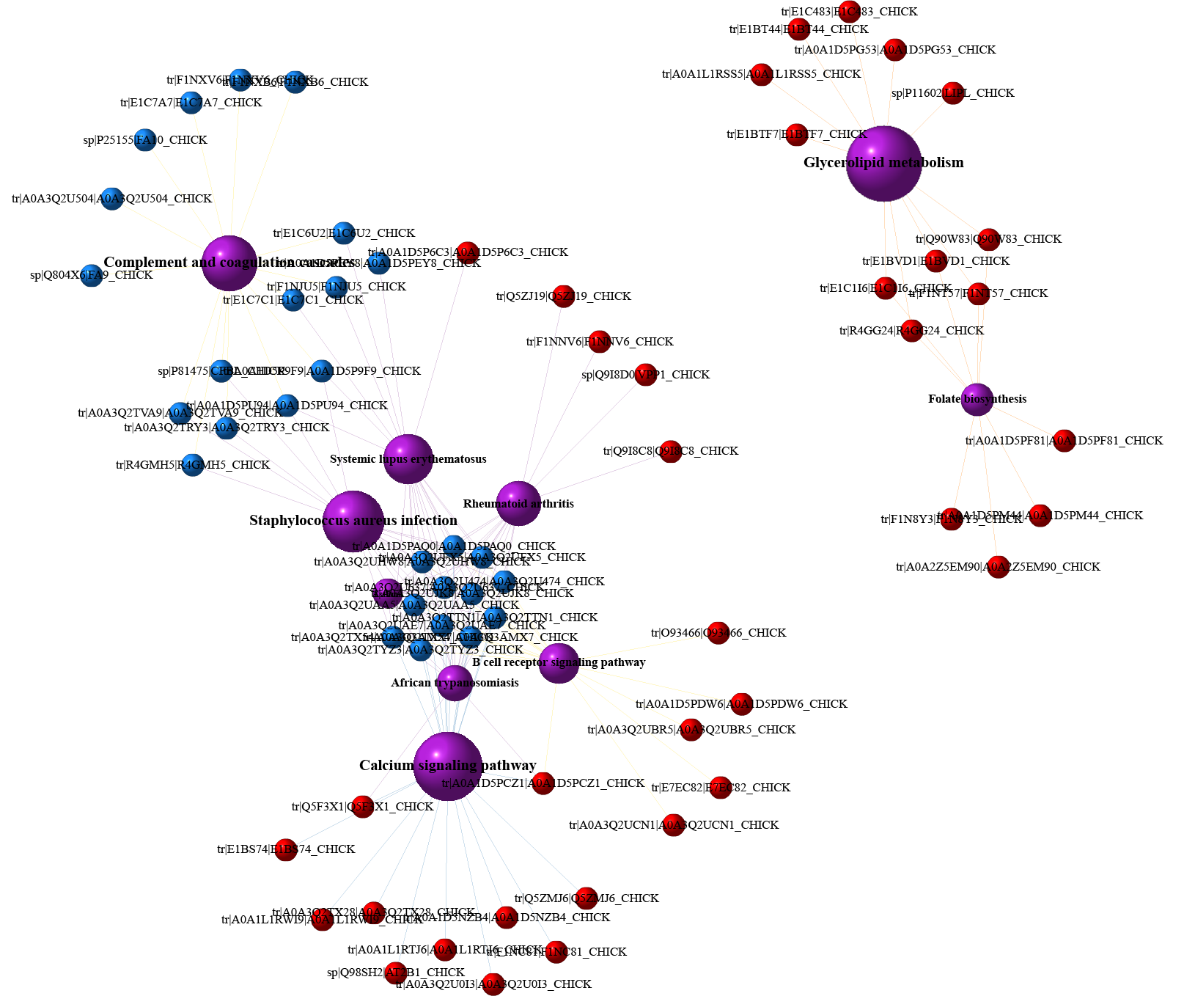


**Figure S2.** Pathway relationship networks of differentially expressed proteins (DEPs). Purple denote pathways; Red and green mean up- and down-regulation, respectively.


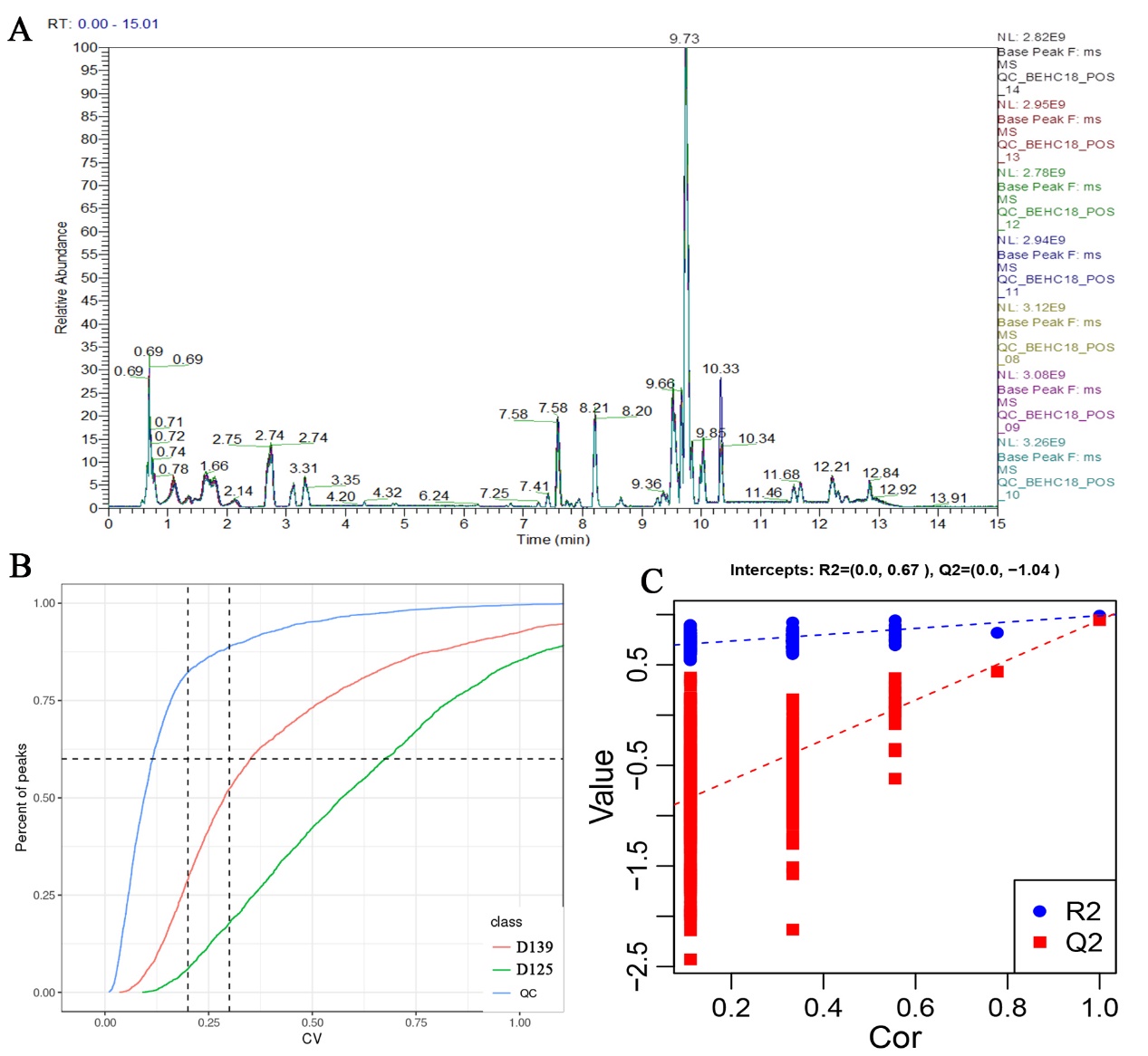


**Figure S3.** Characterization of differential metabolites (DMs) identified by LC-MS/MS positive ion mode. (A) The base peak chromatogram of all quality control (QC) samples. (B) The coefficient of variation (CV) of all QC samples. (C) Partial least squares method-discriminant analysis (PLS-DA) model were performed response permutation testing.


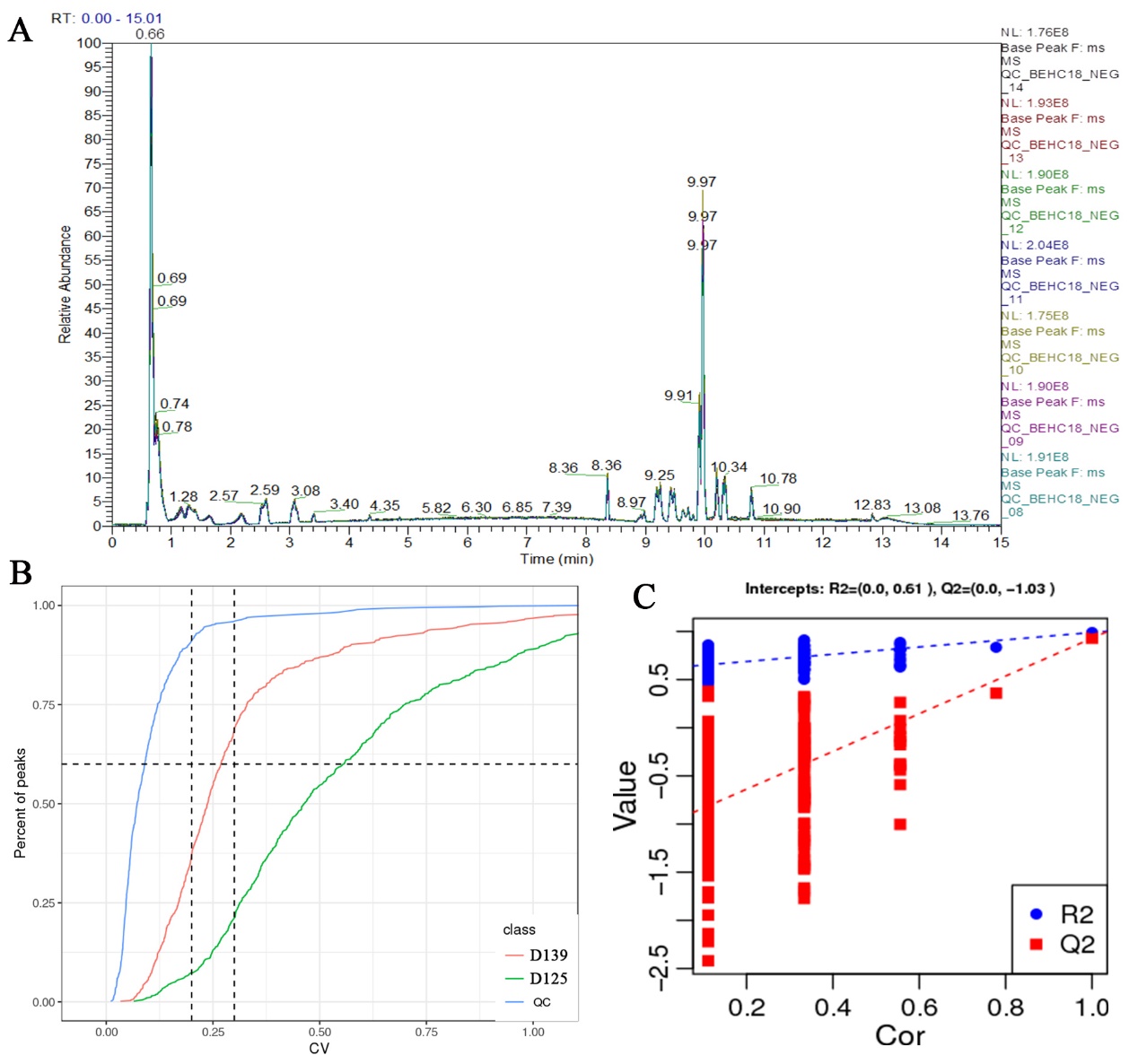


**Figure S4.** Characterization of differential metabolites (DMs) identified by LC-MS/MS negative ion mode. (A) The base peak chromatogram of all quality control (QC) samples. (B) The coefficient of variation (CV) of all QC samples. (C) Partial least squares method-discriminant analysis (PLS-DA) model were performed response permutation testing.
