# Supplementary material for: Integrated Proteomic and Metabolomic Analyses of Chicken Ovary Revealed the Crucial Role of Lipoprotein Lipase on Lipid Metabolism and Steroidogenesis During Sexual Maturity
Source: Front Physiol. 2022 Apr 29;13:885030. doi: 10.3389/fphys.2022.885030 (PMC9099287; doi:10.3389/fphys.2022.885030)
Supplement: Supplementary file 2 [file Table2.DOCX]

**Supplementary Table 1.** Up or down regulation of differential proteins in GO function classification.

| Ontoloty | GO_term | Protein_ID | log2  Foldchange | Up/  Down |
| --- | --- | --- | --- | --- |
| biological_process | behavior | tr|A0A1D5NZB4|A0A1D5NZB4_CHICK | 1.671702 | up |
| biological_process | behavior | tr|A0A1D5PIT4|A0A1D5PIT4_CHICK | -1.97988 | down |
| biological_process | behavior | tr|A0A1D5PTE8|A0A1D5PTE8_CHICK | 1.960486 | up |
| biological_process | behavior | tr|A0A3Q2U8I0|A0A3Q2U8I0_CHICK | 1.050068 | up |
| biological_process | behavior | tr|F1NT80|F1NT80_CHICK | 1.404176 | up |
| biological_process | behavior | tr|F1NTQ2|F1NTQ2_CHICK | 1.236275 | up |
| biological_process | biological adhesion | sp|P00523|SRC_CHICK | 1.599352 | up |
| biological_process | biological adhesion | sp|P42292|CD166_CHICK | 1.297212 | up |
| biological_process | biological adhesion | tr|A0A1D5PQC1|A0A1D5PQC1_CHICK | 1.667257 | up |
| biological_process | biological adhesion | tr|A0A1D5PWD7|A0A1D5PWD7_CHICK | 1.676687 | up |
| biological_process | biological adhesion | tr|A0A3Q2UCN1|A0A3Q2UCN1_CHICK | 1.92272 | up |
| biological_process | biological adhesion | tr|E1BTI7|E1BTI7_CHICK | 1.813027 | up |
| biological_process | biological adhesion | tr|E1C2F2|E1C2F2_CHICK | 1.546061 | up |
| biological_process | biological adhesion | tr|E7EC82|E7EC82_CHICK | 1.586027 | up |
| biological_process | biological adhesion | tr|O93466|O93466_CHICK | 1.669881 | up |
| biological_process | biological adhesion | tr|Q5F3X1|Q5F3X1_CHICK | 1.19047 | up |
| biological_process | biological adhesion | tr|Q5ZL65|Q5ZL65_CHICK | 1.695268 | up |
| biological_process | biological adhesion | tr|Q9PUJ4|Q9PUJ4_CHICK | 1.664957 | up |
| biological_process | biological regulation | sp|O42395|CNBP_CHICK | 1.365125 | up |
| biological_process | biological regulation | sp|O93436|STAM2_CHICK | 2.376336 | up |
| biological_process | biological regulation | sp|O93602|ATF2_CHICK | 2.071233 | up |
| biological_process | biological regulation | sp|P00523|SRC_CHICK | 1.599352 | up |
| biological_process | biological regulation | sp|P02197|MYG_CHICK | 2.885059 | up |
| biological_process | biological regulation | sp|P07090|CALB2_CHICK | 1.954973 | up |
| biological_process | biological regulation | sp|P25155|FA10_CHICK | -3.77432 | down |
| biological_process | biological regulation | sp|P28318|M126_CHICK | 3.846633 | up |
| biological_process | biological regulation | sp|P52162|MAX_CHICK | 1.334512 | up |
| biological_process | biological regulation | sp|P81475|CFBL_CHICK | -1.75388 | down |
| biological_process | biological regulation | sp|Q05423|FABP7_CHICK | 1.9874 | up |
| biological_process | biological regulation | sp|Q5F3K4|WDR48_CHICK | 1.71564 | up |
| biological_process | biological regulation | sp|Q5ZI08|SPT5H_CHICK | 1.235361 | up |
| biological_process | biological regulation | sp|Q5ZIB9|ANM7_CHICK | 1.168118 | up |
| biological_process | biological regulation | sp|Q5ZII9|TFP11_CHICK | 1.172107 | up |
| biological_process | biological regulation | sp|Q5ZIK2|PDZ11_CHICK | 1.259402 | up |
| biological_process | biological regulation | sp|Q5ZIP4|XRN2_CHICK | 1.286156 | up |
| biological_process | biological regulation | sp|Q5ZJ00|EM55_CHICK | 1.664901 | up |
| biological_process | biological regulation | sp|Q5ZJH9|DKC1_CHICK | 1.436701 | up |
| biological_process | biological regulation | sp|Q5ZJW6|KBRS2_CHICK | 1.256362 | up |
| biological_process | biological regulation | sp|Q5ZK05|TOLIP_CHICK | 1.088317 | up |
| biological_process | biological regulation | sp|Q5ZK92|SPAST_CHICK | 1.665787 | up |
| biological_process | biological regulation | sp|Q5ZL36|ZFY27_CHICK | 1.948763 | up |
| biological_process | biological regulation | sp|Q5ZM60|CCPG1_CHICK | 1.624972 | up |
| biological_process | biological regulation | sp|Q5ZM83|MIRO2_CHICK | 1.659999 | up |
| biological_process | biological regulation | sp|Q5ZMG0|DOPD_CHICK | 2.121364 | up |
| biological_process | biological regulation | sp|Q5ZML0|BABA2_CHICK | 1.144731 | up |
| biological_process | biological regulation | sp|Q804X6|FA9_CHICK | -5.71386 | down |
| biological_process | biological regulation | sp|Q8AXL1|SAT1_CHICK | 1.12819 | up |
| biological_process | biological regulation | sp|Q90733|COT2_CHICK | 1.595685 | up |
| biological_process | biological regulation | sp|Q9I8D0|VPP1_CHICK | 1.736096 | up |
| biological_process | biological regulation | sp|Q9IAM7|MRE11_CHICK | 1.337957 | up |
| biological_process | biological regulation | tr|A0A1D5NUD3|A0A1D5NUD3_CHICK | 1.402549 | up |
| biological_process | biological regulation | tr|A0A1D5NUD9|A0A1D5NUD9_CHICK | 1.157212 | up |
| biological_process | biological regulation | tr|A0A1D5NUU0|A0A1D5NUU0_CHICK | 1.000304 | up |
| biological_process | biological regulation | tr|A0A1D5NUV0|A0A1D5NUV0_CHICK | 2.157037 | up |
| biological_process | biological regulation | tr|A0A1D5NWV6|A0A1D5NWV6_CHICK | 1.553336 | up |
| biological_process | biological regulation | tr|A0A1D5NZB4|A0A1D5NZB4_CHICK | 1.671702 | up |
| biological_process | biological regulation | tr|A0A1D5NZS7|A0A1D5NZS7_CHICK | 1.185694 | up |
| biological_process | biological regulation | tr|A0A1D5P047|A0A1D5P047_CHICK | 1.659689 | up |
| biological_process | biological regulation | tr|A0A1D5P5X4|A0A1D5P5X4_CHICK | 1.559711 | up |
| biological_process | biological regulation | tr|A0A1D5P6C3|A0A1D5P6C3_CHICK | 2.100747 | up |
| biological_process | biological regulation | tr|A0A1D5P822|A0A1D5P822_CHICK | 1.478805 | up |
| biological_process | biological regulation | tr|A0A1D5P8H7|A0A1D5P8H7_CHICK | 1.530513 | up |
| biological_process | biological regulation | tr|A0A1D5P997|A0A1D5P997_CHICK | 1.563709 | up |
| biological_process | biological regulation | tr|A0A1D5P9G2|A0A1D5P9G2_CHICK | 1.768438 | up |
| biological_process | biological regulation | tr|A0A1D5PA08|A0A1D5PA08_CHICK | 1.946165 | up |
| biological_process | biological regulation | tr|A0A1D5PCZ1|A0A1D5PCZ1_CHICK | 1.542638 | up |
| biological_process | biological regulation | tr|A0A1D5PFS2|A0A1D5PFS2_CHICK | 1.463999 | up |
| biological_process | biological regulation | tr|A0A1D5PIB1|A0A1D5PIB1_CHICK | 2.10824 | up |
| biological_process | biological regulation | tr|A0A1D5PIT4|A0A1D5PIT4_CHICK | -1.97988 | down |
| biological_process | biological regulation | tr|A0A1D5PK72|A0A1D5PK72_CHICK | 2.032922 | up |
| biological_process | biological regulation | tr|A0A1D5PKI2|A0A1D5PKI2_CHICK | 1.392336 | up |
| biological_process | biological regulation | tr|A0A1D5PQ66|A0A1D5PQ66_CHICK | 1.664552 | up |
| biological_process | biological regulation | tr|A0A1D5PQD0|A0A1D5PQD0_CHICK | 1.314273 | up |
| biological_process | biological regulation | tr|A0A1D5PQG6|A0A1D5PQG6_CHICK | 2.091788 | up |
| biological_process | biological regulation | tr|A0A1D5PR34|A0A1D5PR34_CHICK | 1.370909 | up |
| biological_process | biological regulation | tr|A0A1D5PRI4|A0A1D5PRI4_CHICK | 1.28486 | up |
| biological_process | biological regulation | tr|A0A1D5PRI6|A0A1D5PRI6_CHICK | 1.580404 | up |
| biological_process | biological regulation | tr|A0A1D5PTE8|A0A1D5PTE8_CHICK | 1.960486 | up |
| biological_process | biological regulation | tr|A0A1D5PUJ5|A0A1D5PUJ5_CHICK | 1.266991 | up |
| biological_process | biological regulation | tr|A0A1D5PWD7|A0A1D5PWD7_CHICK | 1.676687 | up |
| biological_process | biological regulation | tr|A0A1D5PWT4|A0A1D5PWT4_CHICK | 1.101328 | up |
| biological_process | biological regulation | tr|A0A1D5PXN4|A0A1D5PXN4_CHICK | -1.85723 | down |
| biological_process | biological regulation | tr|A0A1D5PYB7|A0A1D5PYB7_CHICK | 1.489522 | up |
| biological_process | biological regulation | tr|A0A1L1RWR0|A0A1L1RWR0_CHICK | -2.84844 | down |
| biological_process | biological regulation | tr|A0A3Q2TUF8|A0A3Q2TUF8_CHICK | 1.02023 | up |
| biological_process | biological regulation | tr|A0A3Q2TVU0|A0A3Q2TVU0_CHICK | 1.280793 | up |
| biological_process | biological regulation | tr|A0A3Q2TY77|A0A3Q2TY77_CHICK | 1.82929 | up |
| biological_process | biological regulation | tr|A0A3Q2TYP7|A0A3Q2TYP7_CHICK | 2.159057 | up |
| biological_process | biological regulation | tr|A0A3Q2TZM7|A0A3Q2TZM7_CHICK | 1.419267 | up |
| biological_process | biological regulation | tr|A0A3Q2U504|A0A3Q2U504_CHICK | -2.35777 | down |
| biological_process | biological regulation | tr|A0A3Q2U8I0|A0A3Q2U8I0_CHICK | 1.050068 | up |
| biological_process | biological regulation | tr|A0A3Q2UBJ7|A0A3Q2UBJ7_CHICK | 1.945958 | up |
| biological_process | biological regulation | tr|A0A3Q2UCN1|A0A3Q2UCN1_CHICK | 1.92272 | up |
| biological_process | biological regulation | tr|A0A3Q2UD87|A0A3Q2UD87_CHICK | 1.205498 | up |
| biological_process | biological regulation | tr|A0A3Q2UM13|A0A3Q2UM13_CHICK | 3.560203 | up |
| biological_process | biological regulation | tr|A0A3Q2UPF3|A0A3Q2UPF3_CHICK | 1.295517 | up |
| biological_process | biological regulation | tr|A0A3Q3AB84|A0A3Q3AB84_CHICK | 1.170717 | up |
| biological_process | biological regulation | tr|A0A3Q3ALC8|A0A3Q3ALC8_CHICK | 2.188996 | up |
| biological_process | biological regulation | tr|A0A3S5ZPH2|A0A3S5ZPH2_CHICK | 1.677715 | up |
| biological_process | biological regulation | tr|A8VIF4|A8VIF4_CHICK | 1.024165 | up |
| biological_process | biological regulation | tr|D2D3P4|D2D3P4_CHICK | 2.146381 | up |
| biological_process | biological regulation | tr|E1BQC2|E1BQC2_CHICK | -2.52084 | down |
| biological_process | biological regulation | tr|E1BQI0|E1BQI0_CHICK | 1.067708 | up |
| biological_process | biological regulation | tr|E1BQW2|E1BQW2_CHICK | 1.823966 | up |
| biological_process | biological regulation | tr|E1BRQ9|E1BRQ9_CHICK | 1.034807 | up |
| biological_process | biological regulation | tr|E1BRR6|E1BRR6_CHICK | 1.332531 | up |
| biological_process | biological regulation | tr|E1BS94|E1BS94_CHICK | 1.70074 | up |
| biological_process | biological regulation | tr|E1BSH7|E1BSH7_CHICK | 1.527514 | up |
| biological_process | biological regulation | tr|E1BTE2|E1BTE2_CHICK | -2.67874 | down |
| biological_process | biological regulation | tr|E1BTV1|E1BTV1_CHICK | 2.001762 | up |
| biological_process | biological regulation | tr|E1BU27|E1BU27_CHICK | 3.035848 | up |
| biological_process | biological regulation | tr|E1BU89|E1BU89_CHICK | 1.303565 | up |
| biological_process | biological regulation | tr|E1BUJ1|E1BUJ1_CHICK | 2.709557 | up |
| biological_process | biological regulation | tr|E1BUX5|E1BUX5_CHICK | 1.099122 | up |
| biological_process | biological regulation | tr|E1BVQ3|E1BVQ3_CHICK | 1.650579 | up |
| biological_process | biological regulation | tr|E1BW27|E1BW27_CHICK | 1.938051 | up |
| biological_process | biological regulation | tr|E1BWB7|E1BWB7_CHICK | 1.446045 | up |
| biological_process | biological regulation | tr|E1BX21|E1BX21_CHICK | 1.42634 | up |
| biological_process | biological regulation | tr|E1BXS1|E1BXS1_CHICK | 1.514054 | up |
| biological_process | biological regulation | tr|E1BY40|E1BY40_CHICK | 1.129106 | up |
| biological_process | biological regulation | tr|E1BYQ3|E1BYQ3_CHICK | 1.202088 | up |
| biological_process | biological regulation | tr|E1BZ79|E1BZ79_CHICK | 2.388844 | up |
| biological_process | biological regulation | tr|E1C312|E1C312_CHICK | 1.811403 | up |
| biological_process | biological regulation | tr|E1C396|E1C396_CHICK | 1.08258 | up |
| biological_process | biological regulation | tr|E1C3U6|E1C3U6_CHICK | 1.144268 | up |
| biological_process | biological regulation | tr|E1C592|E1C592_CHICK | 1.565005 | up |
| biological_process | biological regulation | tr|E1C688|E1C688_CHICK | 1.775755 | up |
| biological_process | biological regulation | tr|E1C6S7|E1C6S7_CHICK | 1.1895 | up |
| biological_process | biological regulation | tr|E1C7H6|E1C7H6_CHICK | -1.05537 | down |
| biological_process | biological regulation | tr|E1C7P7|E1C7P7_CHICK | 2.341246 | up |
| biological_process | biological regulation | tr|E1C8U1|E1C8U1_CHICK | 1.411305 | up |
| biological_process | biological regulation | tr|E7EC82|E7EC82_CHICK | 1.586027 | up |
| biological_process | biological regulation | tr|F1N8V5|F1N8V5_CHICK | 2.344853 | up |
| biological_process | biological regulation | tr|F1N977|F1N977_CHICK | 1.720618 | up |
| biological_process | biological regulation | tr|F1N9S3|F1N9S3_CHICK | 1.256683 | up |
| biological_process | biological regulation | tr|F1N9U0|F1N9U0_CHICK | 1.038918 | up |
| biological_process | biological regulation | tr|F1N9Y3|F1N9Y3_CHICK | 1.33841 | up |
| biological_process | biological regulation | tr|F1NAS0|F1NAS0_CHICK | 1.779766 | up |
| biological_process | biological regulation | tr|F1NBX1|F1NBX1_CHICK | 1.439614 | up |
| biological_process | biological regulation | tr|F1NDH2|F1NDH2_CHICK | -1.95135 | down |
| biological_process | biological regulation | tr|F1NIQ3|F1NIQ3_CHICK | 1.483149 | up |
| biological_process | biological regulation | tr|F1NIY3|F1NIY3_CHICK | 1.568578 | up |
| biological_process | biological regulation | tr|F1NJU5|F1NJU5_CHICK | -1.49981 | down |
| biological_process | biological regulation | tr|F1NMJ9|F1NMJ9_CHICK | -2.4895 | down |
| biological_process | biological regulation | tr|F1NPS5|F1NPS5_CHICK | 3.319197 | up |
| biological_process | biological regulation | tr|F1NQ61|F1NQ61_CHICK | 1.080306 | up |
| biological_process | biological regulation | tr|F1NR86|F1NR86_CHICK | 1.817533 | up |
| biological_process | biological regulation | tr|F1NRZ4|F1NRZ4_CHICK | 1.14911 | up |
| biological_process | biological regulation | tr|F1NSF2|F1NSF2_CHICK | 1.708711 | up |
| biological_process | biological regulation | tr|F1NSI7|F1NSI7_CHICK | 1.77407 | up |
| biological_process | biological regulation | tr|F1NSQ4|F1NSQ4_CHICK | 1.21442 | up |
| biological_process | biological regulation | tr|F1NT58|F1NT58_CHICK | 1.701877 | up |
| biological_process | biological regulation | tr|F1NT80|F1NT80_CHICK | 1.404176 | up |
| biological_process | biological regulation | tr|F1NTQ2|F1NTQ2_CHICK | 1.236275 | up |
| biological_process | biological regulation | tr|F1NXV6|F1NXV6_CHICK | -4.87705 | down |
| biological_process | biological regulation | tr|F1P099|F1P099_CHICK | 1.399388 | up |
| biological_process | biological regulation | tr|F1P151|F1P151_CHICK | 2.603153 | up |
| biological_process | biological regulation | tr|F1P394|F1P394_CHICK | 1.286828 | up |
| biological_process | biological regulation | tr|F1P5A3|F1P5A3_CHICK | 2.127136 | up |
| biological_process | biological regulation | tr|F1P5X6|F1P5X6_CHICK | 1.295882 | up |
| biological_process | biological regulation | tr|F6YX81|F6YX81_CHICK | 1.194552 | up |
| biological_process | biological regulation | tr|F7BYG6|F7BYG6_CHICK | 3.125856 | up |
| biological_process | biological regulation | tr|H9L023|H9L023_CHICK | 1.212184 | up |
| biological_process | biological regulation | tr|I1SV11|I1SV11_CHICK | 1.313667 | up |
| biological_process | biological regulation | tr|O93466|O93466_CHICK | 1.669881 | up |
| biological_process | biological regulation | tr|Q2PUH1|Q2PUH1_CHICK | 1.468226 | up |
| biological_process | biological regulation | tr|Q4ADJ6|Q4ADJ6_CHICK | -4.04825 | down |
| biological_process | biological regulation | tr|Q5DWF6|Q5DWF6_CHICK | 1.289716 | up |
| biological_process | biological regulation | tr|Q5F3R8|Q5F3R8_CHICK | 2.332553 | up |
| biological_process | biological regulation | tr|Q5F3X1|Q5F3X1_CHICK | 1.19047 | up |
| biological_process | biological regulation | tr|Q5ZIL3|Q5ZIL3_CHICK | 1.863958 | up |
| biological_process | biological regulation | tr|Q5ZIZ3|Q5ZIZ3_CHICK | 1.248317 | up |
| biological_process | biological regulation | tr|Q5ZJ19|Q5ZJ19_CHICK | 2.470646 | up |
| biological_process | biological regulation | tr|Q5ZJ96|Q5ZJ96_CHICK | 1.306532 | up |
| biological_process | biological regulation | tr|Q5ZJA8|Q5ZJA8_CHICK | 1.093474 | up |
| biological_process | biological regulation | tr|Q5ZJQ4|Q5ZJQ4_CHICK | 1.678561 | up |
| biological_process | biological regulation | tr|Q5ZKI3|Q5ZKI3_CHICK | 1.326993 | up |
| biological_process | biological regulation | tr|Q5ZL65|Q5ZL65_CHICK | 1.695268 | up |
| biological_process | biological regulation | tr|Q5ZLI0|Q5ZLI0_CHICK | 1.097879 | up |
| biological_process | biological regulation | tr|Q5ZMA7|Q5ZMA7_CHICK | 1.172026 | up |
| biological_process | biological regulation | tr|Q5ZMG8|Q5ZMG8_CHICK | 1.771296 | up |
| biological_process | biological regulation | tr|Q5ZMJ6|Q5ZMJ6_CHICK | 1.845871 | up |
| biological_process | biological regulation | tr|Q5ZMP2|Q5ZMP2_CHICK | 1.165624 | up |
| biological_process | biological regulation | tr|Q7ZTS9|Q7ZTS9_CHICK | 1.923559 | up |
| biological_process | biological regulation | tr|Q9PUJ4|Q9PUJ4_CHICK | 1.664957 | up |
| biological_process | biological regulation | tr|Q9PVL6|Q9PVL6_CHICK | 1.71949 | up |
| biological_process | biological regulation | tr|R4GI86|R4GI86_CHICK | 2.078653 | up |
| biological_process | biological regulation | tr|R4GMH5|R4GMH5_CHICK | -2.32193 | down |
| biological_process | cell aggregation | sp|P42292|CD166_CHICK | 1.297212 | up |
| biological_process | cell killing | tr|A0A3Q2UCN1|A0A3Q2UCN1_CHICK | 1.92272 | up |
| biological_process | cell killing | tr|D2D3P4|D2D3P4_CHICK | 2.146381 | up |
| biological_process | cell killing | tr|F1NXV6|F1NXV6_CHICK | -4.87705 | down |
| biological_process | cell killing | tr|Q5ZMP2|Q5ZMP2_CHICK | 1.165624 | up |
| biological_process | cell proliferation | sp|O42395|CNBP_CHICK | 1.365125 | up |
| biological_process | cell proliferation | sp|P00523|SRC_CHICK | 1.599352 | up |
| biological_process | cell proliferation | sp|Q05423|FABP7_CHICK | 1.9874 | up |
| biological_process | cell proliferation | sp|Q5F3K4|WDR48_CHICK | 1.71564 | up |
| biological_process | cell proliferation | sp|Q8AXL1|SAT1_CHICK | 1.12819 | up |
| biological_process | cell proliferation | sp|Q90733|COT2_CHICK | 1.595685 | up |
| biological_process | cell proliferation | sp|Q9IAM7|MRE11_CHICK | 1.337957 | up |
| biological_process | cell proliferation | tr|A0A1D5NUV0|A0A1D5NUV0_CHICK | 2.157037 | up |
| biological_process | cell proliferation | tr|A0A1D5NZS7|A0A1D5NZS7_CHICK | 1.185694 | up |
| biological_process | cell proliferation | tr|A0A1D5P047|A0A1D5P047_CHICK | 1.659689 | up |
| biological_process | cell proliferation | tr|A0A1D5PA08|A0A1D5PA08_CHICK | 1.946165 | up |
| biological_process | cell proliferation | tr|A0A1D5PIT4|A0A1D5PIT4_CHICK | -1.97988 | down |
| biological_process | cell proliferation | tr|A0A1D5PQD0|A0A1D5PQD0_CHICK | 1.314273 | up |
| biological_process | cell proliferation | tr|A0A3Q2UCN1|A0A3Q2UCN1_CHICK | 1.92272 | up |
| biological_process | cell proliferation | tr|E1BSH7|E1BSH7_CHICK | 1.527514 | up |
| biological_process | cell proliferation | tr|E1BTE2|E1BTE2_CHICK | -2.67874 | down |
| biological_process | cell proliferation | tr|E1C3U6|E1C3U6_CHICK | 1.144268 | up |
| biological_process | cell proliferation | tr|F1N9S3|F1N9S3_CHICK | 1.256683 | up |
| biological_process | cell proliferation | tr|F1NMJ9|F1NMJ9_CHICK | -2.4895 | down |
| biological_process | cell proliferation | tr|F1NXV6|F1NXV6_CHICK | -4.87705 | down |
| biological_process | cell proliferation | tr|Q5F3X1|Q5F3X1_CHICK | 1.19047 | up |
| biological_process | cell proliferation | tr|Q5ZJQ4|Q5ZJQ4_CHICK | 1.678561 | up |
| biological_process | cell proliferation | tr|Q5ZL65|Q5ZL65_CHICK | 1.695268 | up |
| biological_process | cell proliferation | tr|Q9PUJ4|Q9PUJ4_CHICK | 1.664957 | up |
| biological_process | cellular component organization or biogenesis | sp|P00523|SRC_CHICK | 1.599352 | up |
| biological_process | cellular component organization or biogenesis | sp|P42292|CD166_CHICK | 1.297212 | up |
| biological_process | cellular component organization or biogenesis | sp|Q5ZHN3|WIPI2_CHICK | 1.61465 | up |
| biological_process | cellular component organization or biogenesis | sp|Q5ZI74|DHX30_CHICK | 1.265146 | up |
| biological_process | cellular component organization or biogenesis | sp|Q5ZIB9|ANM7_CHICK | 1.168118 | up |
| biological_process | cellular component organization or biogenesis | sp|Q5ZII9|TFP11_CHICK | 1.172107 | up |
| biological_process | cellular component organization or biogenesis | sp|Q5ZIK0|P4K2B_CHICK | 2.036191 | up |
| biological_process | cellular component organization or biogenesis | sp|Q5ZJB7|CHMP7_CHICK | 1.815716 | up |
| biological_process | cellular component organization or biogenesis | sp|Q5ZJH9|DKC1_CHICK | 1.436701 | up |
| biological_process | cellular component organization or biogenesis | sp|Q5ZK92|SPAST_CHICK | 1.665787 | up |
| biological_process | cellular component organization or biogenesis | sp|Q5ZKV4|NUBP2_CHICK | 1.740378 | up |
| biological_process | cellular component organization or biogenesis | sp|Q5ZL36|ZFY27_CHICK | 1.948763 | up |
| biological_process | cellular component organization or biogenesis | sp|Q5ZLD4|TMM11_CHICK | 1.73923 | up |
| biological_process | cellular component organization or biogenesis | sp|Q5ZM83|MIRO2_CHICK | 1.659999 | up |
| biological_process | cellular component organization or biogenesis | sp|Q5ZML0|BABA2_CHICK | 1.144731 | up |
| biological_process | cellular component organization or biogenesis | sp|Q9I8D0|VPP1_CHICK | 1.736096 | up |
| biological_process | cellular component organization or biogenesis | sp|Q9IAM7|MRE11_CHICK | 1.337957 | up |
| biological_process | cellular component organization or biogenesis | tr|A0A1D5NU82|A0A1D5NU82_CHICK | 1.12312 | up |
| biological_process | cellular component organization or biogenesis | tr|A0A1D5NUD9|A0A1D5NUD9_CHICK | 1.157212 | up |
| biological_process | cellular component organization or biogenesis | tr|A0A1D5NZB9|A0A1D5NZB9_CHICK | 1.567222 | up |
| biological_process | cellular component organization or biogenesis | tr|A0A1D5NZS7|A0A1D5NZS7_CHICK | 1.185694 | up |
| biological_process | cellular component organization or biogenesis | tr|A0A1D5NZZ9|A0A1D5NZZ9_CHICK | 2.286092 | up |
| biological_process | cellular component organization or biogenesis | tr|A0A1D5P047|A0A1D5P047_CHICK | 1.659689 | up |
| biological_process | cellular component organization or biogenesis | tr|A0A1D5P607|A0A1D5P607_CHICK | 1.51904 | up |
| biological_process | cellular component organization or biogenesis | tr|A0A1D5P6C3|A0A1D5P6C3_CHICK | 2.100747 | up |
| biological_process | cellular component organization or biogenesis | tr|A0A1D5P7E3|A0A1D5P7E3_CHICK | 1.017643 | up |
| biological_process | cellular component organization or biogenesis | tr|A0A1D5P8H7|A0A1D5P8H7_CHICK | 1.530513 | up |
| biological_process | cellular component organization or biogenesis | tr|A0A1D5PGV4|A0A1D5PGV4_CHICK | 1.524887 | up |
| biological_process | cellular component organization or biogenesis | tr|A0A1D5PQD0|A0A1D5PQD0_CHICK | 1.314273 | up |
| biological_process | cellular component organization or biogenesis | tr|A0A1D5PQG6|A0A1D5PQG6_CHICK | 2.091788 | up |
| biological_process | cellular component organization or biogenesis | tr|A0A1D5PR34|A0A1D5PR34_CHICK | 1.370909 | up |
| biological_process | cellular component organization or biogenesis | tr|A0A1D5PRI4|A0A1D5PRI4_CHICK | 1.28486 | up |
| biological_process | cellular component organization or biogenesis | tr|A0A1D5PTE8|A0A1D5PTE8_CHICK | 1.960486 | up |
| biological_process | cellular component organization or biogenesis | tr|A0A1D5PUJ5|A0A1D5PUJ5_CHICK | 1.266991 | up |
| biological_process | cellular component organization or biogenesis | tr|A0A1D5PWD7|A0A1D5PWD7_CHICK | 1.676687 | up |
| biological_process | cellular component organization or biogenesis | tr|A0A1D5PYU0|A0A1D5PYU0_CHICK | 1.138573 | up |
| biological_process | cellular component organization or biogenesis | tr|A0A1D5PZL3|A0A1D5PZL3_CHICK | 1.786305 | up |
| biological_process | cellular component organization or biogenesis | tr|A0A1L1RSS5|A0A1L1RSS5_CHICK | 1.143155 | up |
| biological_process | cellular component organization or biogenesis | tr|A0A3Q2TYP7|A0A3Q2TYP7_CHICK | 2.159057 | up |
| biological_process | cellular component organization or biogenesis | tr|A0A3Q2TZM7|A0A3Q2TZM7_CHICK | 1.419267 | up |
| biological_process | cellular component organization or biogenesis | tr|A0A3Q2U3C4|A0A3Q2U3C4_CHICK | 1.094483 | up |
| biological_process | cellular component organization or biogenesis | tr|A0A3Q2U853|A0A3Q2U853_CHICK | 1.530605 | up |
| biological_process | cellular component organization or biogenesis | tr|A0A3Q2UCN1|A0A3Q2UCN1_CHICK | 1.92272 | up |
| biological_process | cellular component organization or biogenesis | tr|A0A3Q2UD87|A0A3Q2UD87_CHICK | 1.205498 | up |
| biological_process | cellular component organization or biogenesis | tr|A0A3Q2UPF3|A0A3Q2UPF3_CHICK | 1.295517 | up |
| biological_process | cellular component organization or biogenesis | tr|A0A3Q3AB84|A0A3Q3AB84_CHICK | 1.170717 | up |
| biological_process | cellular component organization or biogenesis | tr|A0A3Q3ALC8|A0A3Q3ALC8_CHICK | 2.188996 | up |
| biological_process | cellular component organization or biogenesis | tr|A8VIF4|A8VIF4_CHICK | 1.024165 | up |
| biological_process | cellular component organization or biogenesis | tr|D2D3P4|D2D3P4_CHICK | 2.146381 | up |
| biological_process | cellular component organization or biogenesis | tr|E1BRQ9|E1BRQ9_CHICK | 1.034807 | up |
| biological_process | cellular component organization or biogenesis | tr|E1BSH7|E1BSH7_CHICK | 1.527514 | up |
| biological_process | cellular component organization or biogenesis | tr|E1BT94|E1BT94_CHICK | 1.49966 | up |
| biological_process | cellular component organization or biogenesis | tr|E1BTE2|E1BTE2_CHICK | -2.67874 | down |
| biological_process | cellular component organization or biogenesis | tr|E1BTV1|E1BTV1_CHICK | 2.001762 | up |
| biological_process | cellular component organization or biogenesis | tr|E1BUX5|E1BUX5_CHICK | 1.099122 | up |
| biological_process | cellular component organization or biogenesis | tr|E1BVQ3|E1BVQ3_CHICK | 1.650579 | up |
| biological_process | cellular component organization or biogenesis | tr|E1BW27|E1BW27_CHICK | 1.938051 | up |
| biological_process | cellular component organization or biogenesis | tr|E1BX21|E1BX21_CHICK | 1.42634 | up |
| biological_process | cellular component organization or biogenesis | tr|E1BXY8|E1BXY8_CHICK | 1.106549 | up |
| biological_process | cellular component organization or biogenesis | tr|E1BY40|E1BY40_CHICK | 1.129106 | up |
| biological_process | cellular component organization or biogenesis | tr|E1BYQ3|E1BYQ3_CHICK | 1.202088 | up |
| biological_process | cellular component organization or biogenesis | tr|E1C312|E1C312_CHICK | 1.811403 | up |
| biological_process | cellular component organization or biogenesis | tr|E1C396|E1C396_CHICK | 1.08258 | up |
| biological_process | cellular component organization or biogenesis | tr|E1C3U6|E1C3U6_CHICK | 1.144268 | up |
| biological_process | cellular component organization or biogenesis | tr|E1C8A2|E1C8A2_CHICK | 1.307606 | up |
| biological_process | cellular component organization or biogenesis | tr|E1C8U1|E1C8U1_CHICK | 1.411305 | up |
| biological_process | cellular component organization or biogenesis | tr|E7EC82|E7EC82_CHICK | 1.586027 | up |
| biological_process | cellular component organization or biogenesis | tr|F1N832|F1N832_CHICK | 1.427435 | up |
| biological_process | cellular component organization or biogenesis | tr|F1N8V5|F1N8V5_CHICK | 2.344853 | up |
| biological_process | cellular component organization or biogenesis | tr|F1N9S3|F1N9S3_CHICK | 1.256683 | up |
| biological_process | cellular component organization or biogenesis | tr|F1N9U0|F1N9U0_CHICK | 1.038918 | up |
| biological_process | cellular component organization or biogenesis | tr|F1NDH2|F1NDH2_CHICK | -1.95135 | down |
| biological_process | cellular component organization or biogenesis | tr|F1NIQ3|F1NIQ3_CHICK | 1.483149 | up |
| biological_process | cellular component organization or biogenesis | tr|F1NMU0|F1NMU0_CHICK | 2.467729 | up |
| biological_process | cellular component organization or biogenesis | tr|F1NPS5|F1NPS5_CHICK | 3.319197 | up |
| biological_process | cellular component organization or biogenesis | tr|F1NSI7|F1NSI7_CHICK | 1.77407 | up |
| biological_process | cellular component organization or biogenesis | tr|F1NSQ4|F1NSQ4_CHICK | 1.21442 | up |
| biological_process | cellular component organization or biogenesis | tr|F1NT80|F1NT80_CHICK | 1.404176 | up |
| biological_process | cellular component organization or biogenesis | tr|F1NTQ2|F1NTQ2_CHICK | 1.236275 | up |
| biological_process | cellular component organization or biogenesis | tr|F1NUY0|F1NUY0_CHICK | 1.570572 | up |
| biological_process | cellular component organization or biogenesis | tr|F1NXV6|F1NXV6_CHICK | -4.87705 | down |
| biological_process | cellular component organization or biogenesis | tr|F1NYG7|F1NYG7_CHICK | 1.537967 | up |
| biological_process | cellular component organization or biogenesis | tr|F1P394|F1P394_CHICK | 1.286828 | up |
| biological_process | cellular component organization or biogenesis | tr|F1P5A3|F1P5A3_CHICK | 2.127136 | up |
| biological_process | cellular component organization or biogenesis | tr|F6R228|F6R228_CHICK | 1.426396 | up |
| biological_process | cellular component organization or biogenesis | tr|H9L0M7|H9L0M7_CHICK | 1.164684 | up |
| biological_process | cellular component organization or biogenesis | tr|I1SV11|I1SV11_CHICK | 1.313667 | up |
| biological_process | cellular component organization or biogenesis | tr|O93466|O93466_CHICK | 1.669881 | up |
| biological_process | cellular component organization or biogenesis | tr|Q5F351|Q5F351_CHICK | 1.342446 | up |
| biological_process | cellular component organization or biogenesis | tr|Q5F3R8|Q5F3R8_CHICK | 2.332553 | up |
| biological_process | cellular component organization or biogenesis | tr|Q5F3X1|Q5F3X1_CHICK | 1.19047 | up |
| biological_process | cellular component organization or biogenesis | tr|Q5ZIL3|Q5ZIL3_CHICK | 1.863958 | up |
| biological_process | cellular component organization or biogenesis | tr|Q5ZIZ3|Q5ZIZ3_CHICK | 1.248317 | up |
| biological_process | cellular component organization or biogenesis | tr|Q5ZJA8|Q5ZJA8_CHICK | 1.093474 | up |
| biological_process | cellular component organization or biogenesis | tr|Q5ZJQ4|Q5ZJQ4_CHICK | 1.678561 | up |
| biological_process | cellular component organization or biogenesis | tr|Q5ZKI3|Q5ZKI3_CHICK | 1.326993 | up |
| biological_process | cellular component organization or biogenesis | tr|Q5ZKY5|Q5ZKY5_CHICK | 1.620297 | up |
| biological_process | cellular component organization or biogenesis | tr|Q5ZL65|Q5ZL65_CHICK | 1.695268 | up |
| biological_process | cellular component organization or biogenesis | tr|Q5ZMA7|Q5ZMA7_CHICK | 1.172026 | up |
| biological_process | cellular component organization or biogenesis | tr|Q5ZMJ6|Q5ZMJ6_CHICK | 1.845871 | up |
| biological_process | cellular component organization or biogenesis | tr|Q5ZMP2|Q5ZMP2_CHICK | 1.165624 | up |
| biological_process | cellular component organization or biogenesis | tr|Q9PUJ4|Q9PUJ4_CHICK | 1.664957 | up |
| biological_process | cellular component organization or biogenesis | tr|R4GHH2|R4GHH2_CHICK | 1.778292 | up |
| biological_process | cellular process | sp|O42395|CNBP_CHICK | 1.365125 | up |
| biological_process | cellular process | sp|O93436|STAM2_CHICK | 2.376336 | up |
| biological_process | cellular process | sp|O93602|ATF2_CHICK | 2.071233 | up |
| biological_process | cellular process | sp|P00337|LDHB_CHICK | 2.131478 | up |
| biological_process | cellular process | sp|P00523|SRC_CHICK | 1.599352 | up |
| biological_process | cellular process | sp|P00940|TPIS_CHICK | 2.162937 | up |
| biological_process | cellular process | sp|P02197|MYG_CHICK | 2.885059 | up |
| biological_process | cellular process | sp|P07090|CALB2_CHICK | 1.954973 | up |
| biological_process | cellular process | sp|P17923|NFIA_CHICK | 1.346459 | up |
| biological_process | cellular process | sp|P18936|NU1M_CHICK | 2.034658 | up |
| biological_process | cellular process | sp|P25155|FA10_CHICK | -3.77432 | down |
| biological_process | cellular process | sp|P42292|CD166_CHICK | 1.297212 | up |
| biological_process | cellular process | sp|P52162|MAX_CHICK | 1.334512 | up |
| biological_process | cellular process | sp|Q05423|FABP7_CHICK | 1.9874 | up |
| biological_process | cellular process | sp|Q5F3K4|WDR48_CHICK | 1.71564 | up |
| biological_process | cellular process | sp|Q5F3Z3|UB2V2_CHICK | 2.366484 | up |
| biological_process | cellular process | sp|Q5F480|ITPK1_CHICK | 1.066359 | up |
| biological_process | cellular process | sp|Q5ZHN3|WIPI2_CHICK | 1.61465 | up |
| biological_process | cellular process | sp|Q5ZHT1|ACD11_CHICK | 1.653324 | up |
| biological_process | cellular process | sp|Q5ZI08|SPT5H_CHICK | 1.235361 | up |
| biological_process | cellular process | sp|Q5ZI74|DHX30_CHICK | 1.265146 | up |
| biological_process | cellular process | sp|Q5ZIB9|ANM7_CHICK | 1.168118 | up |
| biological_process | cellular process | sp|Q5ZII9|TFP11_CHICK | 1.172107 | up |
| biological_process | cellular process | sp|Q5ZIK0|P4K2B_CHICK | 2.036191 | up |
| biological_process | cellular process | sp|Q5ZIK2|PDZ11_CHICK | 1.259402 | up |
| biological_process | cellular process | sp|Q5ZIP4|XRN2_CHICK | 1.286156 | up |
| biological_process | cellular process | sp|Q5ZJ00|EM55_CHICK | 1.664901 | up |
| biological_process | cellular process | sp|Q5ZJ08|SYYC_CHICK | 1.23041 | up |
| biological_process | cellular process | sp|Q5ZJA9|BORC5_CHICK | 1.655713 | up |
| biological_process | cellular process | sp|Q5ZJB7|CHMP7_CHICK | 1.815716 | up |
| biological_process | cellular process | sp|Q5ZJH9|DKC1_CHICK | 1.436701 | up |
| biological_process | cellular process | sp|Q5ZJW6|KBRS2_CHICK | 1.256362 | up |
| biological_process | cellular process | sp|Q5ZK05|TOLIP_CHICK | 1.088317 | up |
| biological_process | cellular process | sp|Q5ZK92|SPAST_CHICK | 1.665787 | up |
| biological_process | cellular process | sp|Q5ZKV4|NUBP2_CHICK | 1.740378 | up |
| biological_process | cellular process | sp|Q5ZL36|ZFY27_CHICK | 1.948763 | up |
| biological_process | cellular process | sp|Q5ZLD4|TMM11_CHICK | 1.73923 | up |
| biological_process | cellular process | sp|Q5ZLV2|PPIL3_CHICK | 1.29867 | up |
| biological_process | cellular process | sp|Q5ZM60|CCPG1_CHICK | 1.624972 | up |
| biological_process | cellular process | sp|Q5ZM83|MIRO2_CHICK | 1.659999 | up |
| biological_process | cellular process | sp|Q5ZMG0|DOPD_CHICK | 2.121364 | up |
| biological_process | cellular process | sp|Q5ZML0|BABA2_CHICK | 1.144731 | up |
| biological_process | cellular process | sp|Q8AXL1|SAT1_CHICK | 1.12819 | up |
| biological_process | cellular process | sp|Q90733|COT2_CHICK | 1.595685 | up |
| biological_process | cellular process | sp|Q9I8D0|VPP1_CHICK | 1.736096 | up |
| biological_process | cellular process | sp|Q9IAM7|MRE11_CHICK | 1.337957 | up |
| biological_process | cellular process | tr|A0A1D5NT70|A0A1D5NT70_CHICK | 1.819215 | up |
| biological_process | cellular process | tr|A0A1D5NU82|A0A1D5NU82_CHICK | 1.12312 | up |
| biological_process | cellular process | tr|A0A1D5NUD3|A0A1D5NUD3_CHICK | 1.402549 | up |
| biological_process | cellular process | tr|A0A1D5NUD9|A0A1D5NUD9_CHICK | 1.157212 | up |
| biological_process | cellular process | tr|A0A1D5NUK5|A0A1D5NUK5_CHICK | 2.534566 | up |
| biological_process | cellular process | tr|A0A1D5NUU0|A0A1D5NUU0_CHICK | 1.000304 | up |
| biological_process | cellular process | tr|A0A1D5NUV0|A0A1D5NUV0_CHICK | 2.157037 | up |
| biological_process | cellular process | tr|A0A1D5NWV6|A0A1D5NWV6_CHICK | 1.553336 | up |
| biological_process | cellular process | tr|A0A1D5NXG4|A0A1D5NXG4_CHICK | 1.245324 | up |
| biological_process | cellular process | tr|A0A1D5NXJ2|A0A1D5NXJ2_CHICK | 1.224878 | up |
| biological_process | cellular process | tr|A0A1D5NZB4|A0A1D5NZB4_CHICK | 1.671702 | up |
| biological_process | cellular process | tr|A0A1D5NZB9|A0A1D5NZB9_CHICK | 1.567222 | up |
| biological_process | cellular process | tr|A0A1D5NZS7|A0A1D5NZS7_CHICK | 1.185694 | up |
| biological_process | cellular process | tr|A0A1D5NZZ9|A0A1D5NZZ9_CHICK | 2.286092 | up |
| biological_process | cellular process | tr|A0A1D5P047|A0A1D5P047_CHICK | 1.659689 | up |
| biological_process | cellular process | tr|A0A1D5P1D5|A0A1D5P1D5_CHICK | 2.084726 | up |
| biological_process | cellular process | tr|A0A1D5P5X4|A0A1D5P5X4_CHICK | 1.559711 | up |
| biological_process | cellular process | tr|A0A1D5P607|A0A1D5P607_CHICK | 1.51904 | up |
| biological_process | cellular process | tr|A0A1D5P6C3|A0A1D5P6C3_CHICK | 2.100747 | up |
| biological_process | cellular process | tr|A0A1D5P6L7|A0A1D5P6L7_CHICK | 1.520092 | up |
| biological_process | cellular process | tr|A0A1D5P6M8|A0A1D5P6M8_CHICK | 1.82889 | up |
| biological_process | cellular process | tr|A0A1D5P6S3|A0A1D5P6S3_CHICK | 1.183172 | up |
| biological_process | cellular process | tr|A0A1D5P6Y6|A0A1D5P6Y6_CHICK | 1.32218 | up |
| biological_process | cellular process | tr|A0A1D5P7C5|A0A1D5P7C5_CHICK | 1.273318 | up |
| biological_process | cellular process | tr|A0A1D5P7E3|A0A1D5P7E3_CHICK | 1.017643 | up |
| biological_process | cellular process | tr|A0A1D5P822|A0A1D5P822_CHICK | 1.478805 | up |
| biological_process | cellular process | tr|A0A1D5P8H7|A0A1D5P8H7_CHICK | 1.530513 | up |
| biological_process | cellular process | tr|A0A1D5P997|A0A1D5P997_CHICK | 1.563709 | up |
| biological_process | cellular process | tr|A0A1D5P9G2|A0A1D5P9G2_CHICK | 1.768438 | up |
| biological_process | cellular process | tr|A0A1D5PA08|A0A1D5PA08_CHICK | 1.946165 | up |
| biological_process | cellular process | tr|A0A1D5PAM1|A0A1D5PAM1_CHICK | 2.577291 | up |
| biological_process | cellular process | tr|A0A1D5PC23|A0A1D5PC23_CHICK | 2.344267 | up |
| biological_process | cellular process | tr|A0A1D5PCZ1|A0A1D5PCZ1_CHICK | 1.542638 | up |
| biological_process | cellular process | tr|A0A1D5PFS2|A0A1D5PFS2_CHICK | 1.463999 | up |
| biological_process | cellular process | tr|A0A1D5PFX4|A0A1D5PFX4_CHICK | 1.061266 | up |
| biological_process | cellular process | tr|A0A1D5PG53|A0A1D5PG53_CHICK | 2.149242 | up |
| biological_process | cellular process | tr|A0A1D5PGV4|A0A1D5PGV4_CHICK | 1.524887 | up |
| biological_process | cellular process | tr|A0A1D5PHE2|A0A1D5PHE2_CHICK | 2.577604 | up |
| biological_process | cellular process | tr|A0A1D5PIB1|A0A1D5PIB1_CHICK | 2.10824 | up |
| biological_process | cellular process | tr|A0A1D5PIT4|A0A1D5PIT4_CHICK | -1.97988 | down |
| biological_process | cellular process | tr|A0A1D5PK72|A0A1D5PK72_CHICK | 2.032922 | up |
| biological_process | cellular process | tr|A0A1D5PM26|A0A1D5PM26_CHICK | 2.591272 | up |
| biological_process | cellular process | tr|A0A1D5PMH0|A0A1D5PMH0_CHICK | 1.197966 | up |
| biological_process | cellular process | tr|A0A1D5PQ66|A0A1D5PQ66_CHICK | 1.664552 | up |
| biological_process | cellular process | tr|A0A1D5PQD0|A0A1D5PQD0_CHICK | 1.314273 | up |
| biological_process | cellular process | tr|A0A1D5PQG6|A0A1D5PQG6_CHICK | 2.091788 | up |
| biological_process | cellular process | tr|A0A1D5PR34|A0A1D5PR34_CHICK | 1.370909 | up |
| biological_process | cellular process | tr|A0A1D5PRI4|A0A1D5PRI4_CHICK | 1.28486 | up |
| biological_process | cellular process | tr|A0A1D5PRI6|A0A1D5PRI6_CHICK | 1.580404 | up |
| biological_process | cellular process | tr|A0A1D5PTE8|A0A1D5PTE8_CHICK | 1.960486 | up |
| biological_process | cellular process | tr|A0A1D5PUJ5|A0A1D5PUJ5_CHICK | 1.266991 | up |
| biological_process | cellular process | tr|A0A1D5PWD7|A0A1D5PWD7_CHICK | 1.676687 | up |
| biological_process | cellular process | tr|A0A1D5PWT4|A0A1D5PWT4_CHICK | 1.101328 | up |
| biological_process | cellular process | tr|A0A1D5PXN4|A0A1D5PXN4_CHICK | -1.85723 | down |
| biological_process | cellular process | tr|A0A1D5PYB7|A0A1D5PYB7_CHICK | 1.489522 | up |
| biological_process | cellular process | tr|A0A1D5PYU0|A0A1D5PYU0_CHICK | 1.138573 | up |
| biological_process | cellular process | tr|A0A1D5PZL3|A0A1D5PZL3_CHICK | 1.786305 | up |
| biological_process | cellular process | tr|A0A1D5Q008|A0A1D5Q008_CHICK | 2.962255 | up |
| biological_process | cellular process | tr|A0A1L1RIY5|A0A1L1RIY5_CHICK | 751.4932 | up |
| biological_process | cellular process | tr|A0A1L1RL50|A0A1L1RL50_CHICK | 1.173616 | up |
| biological_process | cellular process | tr|A0A1L1RLU2|A0A1L1RLU2_CHICK | 1.324648 | up |
| biological_process | cellular process | tr|A0A1L1RPD1|A0A1L1RPD1_CHICK | 1.396669 | up |
| biological_process | cellular process | tr|A0A1L1RSS5|A0A1L1RSS5_CHICK | 1.143155 | up |
| biological_process | cellular process | tr|A0A1L1RWR0|A0A1L1RWR0_CHICK | -2.84844 | down |
| biological_process | cellular process | tr|A0A1L1RXA3|A0A1L1RXA3_CHICK | 1.394002 | up |
| biological_process | cellular process | tr|A0A286QZR1|A0A286QZR1_CHICK | 1.043669 | up |
| biological_process | cellular process | tr|A0A3Q2TUF8|A0A3Q2TUF8_CHICK | 1.02023 | up |
| biological_process | cellular process | tr|A0A3Q2TVU0|A0A3Q2TVU0_CHICK | 1.280793 | up |
| biological_process | cellular process | tr|A0A3Q2TVV0|A0A3Q2TVV0_CHICK | 1.145618 | up |
| biological_process | cellular process | tr|A0A3Q2TY77|A0A3Q2TY77_CHICK | 1.82929 | up |
| biological_process | cellular process | tr|A0A3Q2TYP7|A0A3Q2TYP7_CHICK | 2.159057 | up |
| biological_process | cellular process | tr|A0A3Q2TZM7|A0A3Q2TZM7_CHICK | 1.419267 | up |
| biological_process | cellular process | tr|A0A3Q2U3C4|A0A3Q2U3C4_CHICK | 1.094483 | up |
| biological_process | cellular process | tr|A0A3Q2U853|A0A3Q2U853_CHICK | 1.530605 | up |
| biological_process | cellular process | tr|A0A3Q2UB58|A0A3Q2UB58_CHICK | 2.070855 | up |
| biological_process | cellular process | tr|A0A3Q2UBJ7|A0A3Q2UBJ7_CHICK | 1.945958 | up |
| biological_process | cellular process | tr|A0A3Q2UCG6|A0A3Q2UCG6_CHICK | 1.033099 | up |
| biological_process | cellular process | tr|A0A3Q2UCN1|A0A3Q2UCN1_CHICK | 1.92272 | up |
| biological_process | cellular process | tr|A0A3Q2UD87|A0A3Q2UD87_CHICK | 1.205498 | up |
| biological_process | cellular process | tr|A0A3Q2UK74|A0A3Q2UK74_CHICK | 2.254776 | up |
| biological_process | cellular process | tr|A0A3Q2UPF3|A0A3Q2UPF3_CHICK | 1.295517 | up |
| biological_process | cellular process | tr|A0A3Q3AB84|A0A3Q3AB84_CHICK | 1.170717 | up |
| biological_process | cellular process | tr|A0A3Q3ALC8|A0A3Q3ALC8_CHICK | 2.188996 | up |
| biological_process | cellular process | tr|A0A3Q3APG2|A0A3Q3APG2_CHICK | 1.640987 | up |
| biological_process | cellular process | tr|A0A3S5ZPH2|A0A3S5ZPH2_CHICK | 1.677715 | up |
| biological_process | cellular process | tr|A8VIF4|A8VIF4_CHICK | 1.024165 | up |
| biological_process | cellular process | tr|B3VE14|B3VE14_CHICK | -1.63112 | down |
| biological_process | cellular process | tr|D2D3P4|D2D3P4_CHICK | 2.146381 | up |
| biological_process | cellular process | tr|E1BQC2|E1BQC2_CHICK | -2.52084 | down |
| biological_process | cellular process | tr|E1BQI0|E1BQI0_CHICK | 1.067708 | up |
| biological_process | cellular process | tr|E1BQU2|E1BQU2_CHICK | 1.629018 | up |
| biological_process | cellular process | tr|E1BQW2|E1BQW2_CHICK | 1.823966 | up |
| biological_process | cellular process | tr|E1BRQ9|E1BRQ9_CHICK | 1.034807 | up |
| biological_process | cellular process | tr|E1BRT3|E1BRT3_CHICK | 2.214419 | up |
| biological_process | cellular process | tr|E1BSH7|E1BSH7_CHICK | 1.527514 | up |
| biological_process | cellular process | tr|E1BT44|E1BT44_CHICK | 1.432225 | up |
| biological_process | cellular process | tr|E1BT94|E1BT94_CHICK | 1.49966 | up |
| biological_process | cellular process | tr|E1BTE2|E1BTE2_CHICK | -2.67874 | down |
| biological_process | cellular process | tr|E1BTS3|E1BTS3_CHICK | 1.090627 | up |
| biological_process | cellular process | tr|E1BTT8|E1BTT8_CHICK | 1.708105 | up |
| biological_process | cellular process | tr|E1BTV1|E1BTV1_CHICK | 2.001762 | up |
| biological_process | cellular process | tr|E1BU27|E1BU27_CHICK | 3.035848 | up |
| biological_process | cellular process | tr|E1BU89|E1BU89_CHICK | 1.303565 | up |
| biological_process | cellular process | tr|E1BUJ1|E1BUJ1_CHICK | 2.709557 | up |
| biological_process | cellular process | tr|E1BUX5|E1BUX5_CHICK | 1.099122 | up |
| biological_process | cellular process | tr|E1BV97|E1BV97_CHICK | 2.036483 | up |
| biological_process | cellular process | tr|E1BVC8|E1BVC8_CHICK | 2.179244 | up |
| biological_process | cellular process | tr|E1BVQ3|E1BVQ3_CHICK | 1.650579 | up |
| biological_process | cellular process | tr|E1BW27|E1BW27_CHICK | 1.938051 | up |
| biological_process | cellular process | tr|E1BWB7|E1BWB7_CHICK | 1.446045 | up |
| biological_process | cellular process | tr|E1BWG7|E1BWG7_CHICK | 2.421508 | up |
| biological_process | cellular process | tr|E1BX21|E1BX21_CHICK | 1.42634 | up |
| biological_process | cellular process | tr|E1BXC2|E1BXC2_CHICK | 1.767478 | up |
| biological_process | cellular process | tr|E1BXS1|E1BXS1_CHICK | 1.514054 | up |
| biological_process | cellular process | tr|E1BXY8|E1BXY8_CHICK | 1.106549 | up |
| biological_process | cellular process | tr|E1BY22|E1BY22_CHICK | 1.367672 | up |
| biological_process | cellular process | tr|E1BY40|E1BY40_CHICK | 1.129106 | up |
| biological_process | cellular process | tr|E1BYQ3|E1BYQ3_CHICK | 1.202088 | up |
| biological_process | cellular process | tr|E1BZ79|E1BZ79_CHICK | 2.388844 | up |
| biological_process | cellular process | tr|E1C1T2|E1C1T2_CHICK | 1.594659 | up |
| biological_process | cellular process | tr|E1C1Y5|E1C1Y5_CHICK | 1.135922 | up |
| biological_process | cellular process | tr|E1C229|E1C229_CHICK | 1.473036 | up |
| biological_process | cellular process | tr|E1C2E5|E1C2E5_CHICK | 1.182299 | up |
| biological_process | cellular process | tr|E1C310|E1C310_CHICK | 1.545191 | up |
| biological_process | cellular process | tr|E1C312|E1C312_CHICK | 1.811403 | up |
| biological_process | cellular process | tr|E1C396|E1C396_CHICK | 1.08258 | up |
| biological_process | cellular process | tr|E1C3U6|E1C3U6_CHICK | 1.144268 | up |
| biological_process | cellular process | tr|E1C688|E1C688_CHICK | 1.775755 | up |
| biological_process | cellular process | tr|E1C6S7|E1C6S7_CHICK | 1.1895 | up |
| biological_process | cellular process | tr|E1C7H6|E1C7H6_CHICK | -1.05537 | down |
| biological_process | cellular process | tr|E1C7P7|E1C7P7_CHICK | 2.341246 | up |
| biological_process | cellular process | tr|E1C8A2|E1C8A2_CHICK | 1.307606 | up |
| biological_process | cellular process | tr|E1C8U1|E1C8U1_CHICK | 1.411305 | up |
| biological_process | cellular process | tr|E1C9H5|E1C9H5_CHICK | 1.195027 | up |
| biological_process | cellular process | tr|E7EC82|E7EC82_CHICK | 1.586027 | up |
| biological_process | cellular process | tr|F1N832|F1N832_CHICK | 1.427435 | up |
| biological_process | cellular process | tr|F1N8V5|F1N8V5_CHICK | 2.344853 | up |
| biological_process | cellular process | tr|F1N9S3|F1N9S3_CHICK | 1.256683 | up |
| biological_process | cellular process | tr|F1N9U0|F1N9U0_CHICK | 1.038918 | up |
| biological_process | cellular process | tr|F1N9Y3|F1N9Y3_CHICK | 1.33841 | up |
| biological_process | cellular process | tr|F1NBX1|F1NBX1_CHICK | 1.439614 | up |
| biological_process | cellular process | tr|F1NDH2|F1NDH2_CHICK | -1.95135 | down |
| biological_process | cellular process | tr|F1NE88|F1NE88_CHICK | 1.362851 | up |
| biological_process | cellular process | tr|F1NEQ3|F1NEQ3_CHICK | 1.139952 | up |
| biological_process | cellular process | tr|F1NF87|F1NF87_CHICK | 1.015859 | up |
| biological_process | cellular process | tr|F1NIQ3|F1NIQ3_CHICK | 1.483149 | up |
| biological_process | cellular process | tr|F1NIY3|F1NIY3_CHICK | 1.568578 | up |
| biological_process | cellular process | tr|F1NLM0|F1NLM0_CHICK | 1.884615 | up |
| biological_process | cellular process | tr|F1NMJ9|F1NMJ9_CHICK | -2.4895 | down |
| biological_process | cellular process | tr|F1NMM9|F1NMM9_CHICK | 1.449446 | up |
| biological_process | cellular process | tr|F1NMQ3|F1NMQ3_CHICK | 1.885262 | up |
| biological_process | cellular process | tr|F1NMU0|F1NMU0_CHICK | 2.467729 | up |
| biological_process | cellular process | tr|F1NN63|F1NN63_CHICK | 1.72615 | up |
| biological_process | cellular process | tr|F1NPS5|F1NPS5_CHICK | 3.319197 | up |
| biological_process | cellular process | tr|F1NQ61|F1NQ61_CHICK | 1.080306 | up |
| biological_process | cellular process | tr|F1NR86|F1NR86_CHICK | 1.817533 | up |
| biological_process | cellular process | tr|F1NRZ4|F1NRZ4_CHICK | 1.14911 | up |
| biological_process | cellular process | tr|F1NSF2|F1NSF2_CHICK | 1.708711 | up |
| biological_process | cellular process | tr|F1NSI7|F1NSI7_CHICK | 1.77407 | up |
| biological_process | cellular process | tr|F1NSQ4|F1NSQ4_CHICK | 1.21442 | up |
| biological_process | cellular process | tr|F1NT58|F1NT58_CHICK | 1.701877 | up |
| biological_process | cellular process | tr|F1NT80|F1NT80_CHICK | 1.404176 | up |
| biological_process | cellular process | tr|F1NTQ2|F1NTQ2_CHICK | 1.236275 | up |
| biological_process | cellular process | tr|F1NUY0|F1NUY0_CHICK | 1.570572 | up |
| biological_process | cellular process | tr|F1NV66|F1NV66_CHICK | 1.865555 | up |
| biological_process | cellular process | tr|F1NXV6|F1NXV6_CHICK | -4.87705 | down |
| biological_process | cellular process | tr|F1NYG7|F1NYG7_CHICK | 1.537967 | up |
| biological_process | cellular process | tr|F1P099|F1P099_CHICK | 1.399388 | up |
| biological_process | cellular process | tr|F1P151|F1P151_CHICK | 2.603153 | up |
| biological_process | cellular process | tr|F1P2D2|F1P2D2_CHICK | 1.35416 | up |
| biological_process | cellular process | tr|F1P2G6|F1P2G6_CHICK | 1.470473 | up |
| biological_process | cellular process | tr|F1P372|F1P372_CHICK | 1.525043 | up |
| biological_process | cellular process | tr|F1P394|F1P394_CHICK | 1.286828 | up |
| biological_process | cellular process | tr|F1P526|F1P526_CHICK | 1.506479 | up |
| biological_process | cellular process | tr|F1P5A3|F1P5A3_CHICK | 2.127136 | up |
| biological_process | cellular process | tr|F1P5J7|F1P5J7_CHICK | 1.075835 | up |
| biological_process | cellular process | tr|F1P5X6|F1P5X6_CHICK | 1.295882 | up |
| biological_process | cellular process | tr|F6R228|F6R228_CHICK | 1.426396 | up |
| biological_process | cellular process | tr|F6YX81|F6YX81_CHICK | 1.194552 | up |
| biological_process | cellular process | tr|F7BFS9|F7BFS9_CHICK | 1.641443 | up |
| biological_process | cellular process | tr|H9L023|H9L023_CHICK | 1.212184 | up |
| biological_process | cellular process | tr|H9L0M7|H9L0M7_CHICK | 1.164684 | up |
| biological_process | cellular process | tr|I1SV11|I1SV11_CHICK | 1.313667 | up |
| biological_process | cellular process | tr|O93466|O93466_CHICK | 1.669881 | up |
| biological_process | cellular process | tr|Q2PUH1|Q2PUH1_CHICK | 1.468226 | up |
| biological_process | cellular process | tr|Q4ADJ6|Q4ADJ6_CHICK | -4.04825 | down |
| biological_process | cellular process | tr|Q5DWF6|Q5DWF6_CHICK | 1.289716 | up |
| biological_process | cellular process | tr|Q5F351|Q5F351_CHICK | 1.342446 | up |
| biological_process | cellular process | tr|Q5F3R8|Q5F3R8_CHICK | 2.332553 | up |
| biological_process | cellular process | tr|Q5F3X1|Q5F3X1_CHICK | 1.19047 | up |
| biological_process | cellular process | tr|Q5ZHP2|Q5ZHP2_CHICK | 2.412922 | up |
| biological_process | cellular process | tr|Q5ZIL3|Q5ZIL3_CHICK | 1.863958 | up |
| biological_process | cellular process | tr|Q5ZIZ3|Q5ZIZ3_CHICK | 1.248317 | up |
| biological_process | cellular process | tr|Q5ZJ19|Q5ZJ19_CHICK | 2.470646 | up |
| biological_process | cellular process | tr|Q5ZJ96|Q5ZJ96_CHICK | 1.306532 | up |
| biological_process | cellular process | tr|Q5ZJA8|Q5ZJA8_CHICK | 1.093474 | up |
| biological_process | cellular process | tr|Q5ZJQ4|Q5ZJQ4_CHICK | 1.678561 | up |
| biological_process | cellular process | tr|Q5ZJW2|Q5ZJW2_CHICK | 1.71086 | up |
| biological_process | cellular process | tr|Q5ZKI3|Q5ZKI3_CHICK | 1.326993 | up |
| biological_process | cellular process | tr|Q5ZKY5|Q5ZKY5_CHICK | 1.620297 | up |
| biological_process | cellular process | tr|Q5ZL65|Q5ZL65_CHICK | 1.695268 | up |
| biological_process | cellular process | tr|Q5ZLI0|Q5ZLI0_CHICK | 1.097879 | up |
| biological_process | cellular process | tr|Q5ZMA7|Q5ZMA7_CHICK | 1.172026 | up |
| biological_process | cellular process | tr|Q5ZMG8|Q5ZMG8_CHICK | 1.771296 | up |
| biological_process | cellular process | tr|Q5ZMJ6|Q5ZMJ6_CHICK | 1.845871 | up |
| biological_process | cellular process | tr|Q5ZMP2|Q5ZMP2_CHICK | 1.165624 | up |
| biological_process | cellular process | tr|Q7ZTS9|Q7ZTS9_CHICK | 1.923559 | up |
| biological_process | cellular process | tr|Q9DDD4|Q9DDD4_CHICK | -1.44831 | down |
| biological_process | cellular process | tr|Q9PUJ4|Q9PUJ4_CHICK | 1.664957 | up |
| biological_process | cellular process | tr|Q9PVL6|Q9PVL6_CHICK | 1.71949 | up |
| biological_process | cellular process | tr|R4GHH2|R4GHH2_CHICK | 1.778292 | up |
| biological_process | cellular process | tr|R4GI86|R4GI86_CHICK | 2.078653 | up |
| biological_process | cellular process | tr|V9GW30|V9GW30_CHICK | 1.560922 | up |
| biological_process | detoxification | tr|A0A1D5NT70|A0A1D5NT70_CHICK | 1.819215 | up |
| biological_process | detoxification | tr|E1BXC2|E1BXC2_CHICK | 1.767478 | up |
| biological_process | developmental process | sp|P00523|SRC_CHICK | 1.599352 | up |
| biological_process | developmental process | sp|P02197|MYG_CHICK | 2.885059 | up |
| biological_process | developmental process | sp|P42292|CD166_CHICK | 1.297212 | up |
| biological_process | developmental process | sp|Q05423|FABP7_CHICK | 1.9874 | up |
| biological_process | developmental process | sp|Q5F3K4|WDR48_CHICK | 1.71564 | up |
| biological_process | developmental process | sp|Q5ZIB9|ANM7_CHICK | 1.168118 | up |
| biological_process | developmental process | sp|Q5ZK05|TOLIP_CHICK | 1.088317 | up |
| biological_process | developmental process | sp|Q5ZK92|SPAST_CHICK | 1.665787 | up |
| biological_process | developmental process | sp|Q5ZL36|ZFY27_CHICK | 1.948763 | up |
| biological_process | developmental process | sp|Q8AXL1|SAT1_CHICK | 1.12819 | up |
| biological_process | developmental process | sp|Q90733|COT2_CHICK | 1.595685 | up |
| biological_process | developmental process | tr|A0A1D5NUV0|A0A1D5NUV0_CHICK | 2.157037 | up |
| biological_process | developmental process | tr|A0A1D5NXG4|A0A1D5NXG4_CHICK | 1.245324 | up |
| biological_process | developmental process | tr|A0A1D5NZS7|A0A1D5NZS7_CHICK | 1.185694 | up |
| biological_process | developmental process | tr|A0A1D5P6C3|A0A1D5P6C3_CHICK | 2.100747 | up |
| biological_process | developmental process | tr|A0A1D5P822|A0A1D5P822_CHICK | 1.478805 | up |
| biological_process | developmental process | tr|A0A1D5PA08|A0A1D5PA08_CHICK | 1.946165 | up |
| biological_process | developmental process | tr|A0A1D5PIB1|A0A1D5PIB1_CHICK | 2.10824 | up |
| biological_process | developmental process | tr|A0A1D5PQC1|A0A1D5PQC1_CHICK | 1.667257 | up |
| biological_process | developmental process | tr|A0A1D5PQD0|A0A1D5PQD0_CHICK | 1.314273 | up |
| biological_process | developmental process | tr|A0A1D5PRI6|A0A1D5PRI6_CHICK | 1.580404 | up |
| biological_process | developmental process | tr|A0A1D5PWD7|A0A1D5PWD7_CHICK | 1.676687 | up |
| biological_process | developmental process | tr|A0A1L1RPB1|A0A1L1RPB1_CHICK | 1.864507 | up |
| biological_process | developmental process | tr|A0A3Q2TVV0|A0A3Q2TVV0_CHICK | 1.145618 | up |
| biological_process | developmental process | tr|A0A3Q2UCN1|A0A3Q2UCN1_CHICK | 1.92272 | up |
| biological_process | developmental process | tr|A0A3Q2UD87|A0A3Q2UD87_CHICK | 1.205498 | up |
| biological_process | developmental process | tr|A0A3Q2UPF3|A0A3Q2UPF3_CHICK | 1.295517 | up |
| biological_process | developmental process | tr|A0A3Q3ALC8|A0A3Q3ALC8_CHICK | 2.188996 | up |
| biological_process | developmental process | tr|A8VIF4|A8VIF4_CHICK | 1.024165 | up |
| biological_process | developmental process | tr|D2D3P4|D2D3P4_CHICK | 2.146381 | up |
| biological_process | developmental process | tr|E1BQU2|E1BQU2_CHICK | 1.629018 | up |
| biological_process | developmental process | tr|E1BSH7|E1BSH7_CHICK | 1.527514 | up |
| biological_process | developmental process | tr|E1BTE2|E1BTE2_CHICK | -2.67874 | down |
| biological_process | developmental process | tr|E1BWG7|E1BWG7_CHICK | 2.421508 | up |
| biological_process | developmental process | tr|E1BX21|E1BX21_CHICK | 1.42634 | up |
| biological_process | developmental process | tr|E1BY22|E1BY22_CHICK | 1.367672 | up |
| biological_process | developmental process | tr|E1BY40|E1BY40_CHICK | 1.129106 | up |
| biological_process | developmental process | tr|E1BYQ3|E1BYQ3_CHICK | 1.202088 | up |
| biological_process | developmental process | tr|E1C310|E1C310_CHICK | 1.545191 | up |
| biological_process | developmental process | tr|E1C3U6|E1C3U6_CHICK | 1.144268 | up |
| biological_process | developmental process | tr|E1C688|E1C688_CHICK | 1.775755 | up |
| biological_process | developmental process | tr|E1C7H6|E1C7H6_CHICK | -1.05537 | down |
| biological_process | developmental process | tr|E7EC82|E7EC82_CHICK | 1.586027 | up |
| biological_process | developmental process | tr|F1N9Y3|F1N9Y3_CHICK | 1.33841 | up |
| biological_process | developmental process | tr|F1NDH2|F1NDH2_CHICK | -1.95135 | down |
| biological_process | developmental process | tr|F1NIY3|F1NIY3_CHICK | 1.568578 | up |
| biological_process | developmental process | tr|F1NMJ9|F1NMJ9_CHICK | -2.4895 | down |
| biological_process | developmental process | tr|F1NRZ4|F1NRZ4_CHICK | 1.14911 | up |
| biological_process | developmental process | tr|F1NSF2|F1NSF2_CHICK | 1.708711 | up |
| biological_process | developmental process | tr|F1NTQ2|F1NTQ2_CHICK | 1.236275 | up |
| biological_process | developmental process | tr|F1NXV6|F1NXV6_CHICK | -4.87705 | down |
| biological_process | developmental process | tr|F7BFS9|F7BFS9_CHICK | 1.641443 | up |
| biological_process | developmental process | tr|I1SV11|I1SV11_CHICK | 1.313667 | up |
| biological_process | developmental process | tr|O93466|O93466_CHICK | 1.669881 | up |
| biological_process | developmental process | tr|Q2PUH1|Q2PUH1_CHICK | 1.468226 | up |
| biological_process | developmental process | tr|Q5DWF6|Q5DWF6_CHICK | 1.289716 | up |
| biological_process | developmental process | tr|Q5F3X1|Q5F3X1_CHICK | 1.19047 | up |
| biological_process | developmental process | tr|Q5ZKI3|Q5ZKI3_CHICK | 1.326993 | up |
| biological_process | developmental process | tr|Q9DDD4|Q9DDD4_CHICK | -1.44831 | down |
| biological_process | developmental process | tr|Q9PUJ4|Q9PUJ4_CHICK | 1.664957 | up |
| biological_process | growth | sp|P42292|CD166_CHICK | 1.297212 | up |
| biological_process | growth | sp|Q5F3K4|WDR48_CHICK | 1.71564 | up |
| biological_process | growth | sp|Q5ZIP4|XRN2_CHICK | 1.286156 | up |
| biological_process | growth | sp|Q5ZL36|ZFY27_CHICK | 1.948763 | up |
| biological_process | growth | tr|A0A1D5PA08|A0A1D5PA08_CHICK | 1.946165 | up |
| biological_process | growth | tr|A0A1D5PQG6|A0A1D5PQG6_CHICK | 2.091788 | up |
| biological_process | growth | tr|A0A3Q2TVV0|A0A3Q2TVV0_CHICK | 1.145618 | up |
| biological_process | growth | tr|F1NXV6|F1NXV6_CHICK | -4.87705 | down |
| biological_process | growth | tr|I1SV11|I1SV11_CHICK | 1.313667 | up |
| biological_process | growth | tr|Q5ZJQ4|Q5ZJQ4_CHICK | 1.678561 | up |
| biological_process | immune system process | sp|P00523|SRC_CHICK | 1.599352 | up |
| biological_process | immune system process | sp|P02197|MYG_CHICK | 2.885059 | up |
| biological_process | immune system process | sp|P04210|LV1_CHICK | -2.82825 | down |
| biological_process | immune system process | sp|P28318|M126_CHICK | 3.846633 | up |
| biological_process | immune system process | sp|P42292|CD166_CHICK | 1.297212 | up |
| biological_process | immune system process | sp|P81475|CFBL_CHICK | -1.75388 | down |
| biological_process | immune system process | sp|Q5ZJ00|EM55_CHICK | 1.664901 | up |
| biological_process | immune system process | sp|Q5ZK05|TOLIP_CHICK | 1.088317 | up |
| biological_process | immune system process | tr|A0A1D5NUV0|A0A1D5NUV0_CHICK | 2.157037 | up |
| biological_process | immune system process | tr|A0A1D5P453|A0A1D5P453_CHICK | 1.553695 | up |
| biological_process | immune system process | tr|A0A1D5PA08|A0A1D5PA08_CHICK | 1.946165 | up |
| biological_process | immune system process | tr|A0A1D5PCZ1|A0A1D5PCZ1_CHICK | 1.542638 | up |
| biological_process | immune system process | tr|A0A1D5PWD7|A0A1D5PWD7_CHICK | 1.676687 | up |
| biological_process | immune system process | tr|A0A3Q2TYP7|A0A3Q2TYP7_CHICK | 2.159057 | up |
| biological_process | immune system process | tr|A0A3Q2UCN1|A0A3Q2UCN1_CHICK | 1.92272 | up |
| biological_process | immune system process | tr|D2D3P4|D2D3P4_CHICK | 2.146381 | up |
| biological_process | immune system process | tr|E1BQC2|E1BQC2_CHICK | -2.52084 | down |
| biological_process | immune system process | tr|E1BQU2|E1BQU2_CHICK | 1.629018 | up |
| biological_process | immune system process | tr|E1BTI7|E1BTI7_CHICK | 1.813027 | up |
| biological_process | immune system process | tr|E1BX21|E1BX21_CHICK | 1.42634 | up |
| biological_process | immune system process | tr|E1C592|E1C592_CHICK | 1.565005 | up |
| biological_process | immune system process | tr|E1C6U2|E1C6U2_CHICK | -1.17102 | down |
| biological_process | immune system process | tr|E1C7A7|E1C7A7_CHICK | -1.02532 | down |
| biological_process | immune system process | tr|E1C7C1|E1C7C1_CHICK | -1.84307 | down |
| biological_process | immune system process | tr|E7EC82|E7EC82_CHICK | 1.586027 | up |
| biological_process | immune system process | tr|F1NJU5|F1NJU5_CHICK | -1.49981 | down |
| biological_process | immune system process | tr|F1NMJ9|F1NMJ9_CHICK | -2.4895 | down |
| biological_process | immune system process | tr|F1NRZ4|F1NRZ4_CHICK | 1.14911 | up |
| biological_process | immune system process | tr|F1NXV6|F1NXV6_CHICK | -4.87705 | down |
| biological_process | immune system process | tr|Q4ADJ6|Q4ADJ6_CHICK | -4.04825 | down |
| biological_process | immune system process | tr|Q5DWF6|Q5DWF6_CHICK | 1.289716 | up |
| biological_process | immune system process | tr|Q5ZIL3|Q5ZIL3_CHICK | 1.863958 | up |
| biological_process | immune system process | tr|Q5ZL65|Q5ZL65_CHICK | 1.695268 | up |
| biological_process | immune system process | tr|Q5ZMP2|Q5ZMP2_CHICK | 1.165624 | up |
| biological_process | immune system process | tr|Q9PUJ4|Q9PUJ4_CHICK | 1.664957 | up |
| biological_process | localization | sp|O93436|STAM2_CHICK | 2.376336 | up |
| biological_process | localization | sp|P00523|SRC_CHICK | 1.599352 | up |
| biological_process | localization | sp|P19121|ALBU_CHICK | -1.69614 | down |
| biological_process | localization | sp|Q5ZHN3|WIPI2_CHICK | 1.61465 | up |
| biological_process | localization | sp|Q5ZIK2|PDZ11_CHICK | 1.259402 | up |
| biological_process | localization | sp|Q5ZJ00|EM55_CHICK | 1.664901 | up |
| biological_process | localization | sp|Q5ZJA9|BORC5_CHICK | 1.655713 | up |
| biological_process | localization | sp|Q5ZJB7|CHMP7_CHICK | 1.815716 | up |
| biological_process | localization | sp|Q5ZK05|TOLIP_CHICK | 1.088317 | up |
| biological_process | localization | sp|Q5ZK92|SPAST_CHICK | 1.665787 | up |
| biological_process | localization | sp|Q5ZKV9|VPS50_CHICK | 1.324318 | up |
| biological_process | localization | sp|Q5ZL36|ZFY27_CHICK | 1.948763 | up |
| biological_process | localization | sp|Q5ZLT0|XPO7_CHICK | 1.469766 | up |
| biological_process | localization | sp|Q5ZM83|MIRO2_CHICK | 1.659999 | up |
| biological_process | localization | sp|Q90733|COT2_CHICK | 1.595685 | up |
| biological_process | localization | sp|Q9I8D0|VPP1_CHICK | 1.736096 | up |
| biological_process | localization | tr|A0A1D5NTC8|A0A1D5NTC8_CHICK | 1.184237 | up |
| biological_process | localization | tr|A0A1D5NUV0|A0A1D5NUV0_CHICK | 2.157037 | up |
| biological_process | localization | tr|A0A1D5NVT0|A0A1D5NVT0_CHICK | 1.421036 | up |
| biological_process | localization | tr|A0A1D5NW68|A0A1D5NW68_CHICK | -2.66571 | down |
| biological_process | localization | tr|A0A1D5NWV6|A0A1D5NWV6_CHICK | 1.553336 | up |
| biological_process | localization | tr|A0A1D5NX04|A0A1D5NX04_CHICK | 2.142264 | up |
| biological_process | localization | tr|A0A1D5NXG4|A0A1D5NXG4_CHICK | 1.245324 | up |
| biological_process | localization | tr|A0A1D5NXR2|A0A1D5NXR2_CHICK | 1.37138 | up |
| biological_process | localization | tr|A0A1D5P2W5|A0A1D5P2W5_CHICK | 1.407792 | up |
| biological_process | localization | tr|A0A1D5P453|A0A1D5P453_CHICK | 1.553695 | up |
| biological_process | localization | tr|A0A1D5P5X4|A0A1D5P5X4_CHICK | 1.559711 | up |
| biological_process | localization | tr|A0A1D5P8H7|A0A1D5P8H7_CHICK | 1.530513 | up |
| biological_process | localization | tr|A0A1D5P997|A0A1D5P997_CHICK | 1.563709 | up |
| biological_process | localization | tr|A0A1D5PCG3|A0A1D5PCG3_CHICK | 1.321695 | up |
| biological_process | localization | tr|A0A1D5PEU0|A0A1D5PEU0_CHICK | 1.077518 | up |
| biological_process | localization | tr|A0A1D5PFS2|A0A1D5PFS2_CHICK | 1.463999 | up |
| biological_process | localization | tr|A0A1D5PGC8|A0A1D5PGC8_CHICK | 1.343827 | up |
| biological_process | localization | tr|A0A1D5PIB1|A0A1D5PIB1_CHICK | 2.10824 | up |
| biological_process | localization | tr|A0A1D5PK48|A0A1D5PK48_CHICK | -5.8308 | down |
| biological_process | localization | tr|A0A1D5PMH0|A0A1D5PMH0_CHICK | 1.197966 | up |
| biological_process | localization | tr|A0A1D5PQD0|A0A1D5PQD0_CHICK | 1.314273 | up |
| biological_process | localization | tr|A0A1D5PQG6|A0A1D5PQG6_CHICK | 2.091788 | up |
| biological_process | localization | tr|A0A1D5PQQ6|A0A1D5PQQ6_CHICK | 1.432587 | up |
| biological_process | localization | tr|A0A1D5PRI6|A0A1D5PRI6_CHICK | 1.580404 | up |
| biological_process | localization | tr|A0A1D5PWD7|A0A1D5PWD7_CHICK | 1.676687 | up |
| biological_process | localization | tr|A0A1D5PZ78|A0A1D5PZ78_CHICK | 1.936124 | up |
| biological_process | localization | tr|A0A1D5PZF2|A0A1D5PZF2_CHICK | 1.668934 | up |
| biological_process | localization | tr|A0A1D5PZL3|A0A1D5PZL3_CHICK | 1.786305 | up |
| biological_process | localization | tr|A0A1L1RLU2|A0A1L1RLU2_CHICK | 1.324648 | up |
| biological_process | localization | tr|A0A1L1RSS5|A0A1L1RSS5_CHICK | 1.143155 | up |
| biological_process | localization | tr|A0A1L1RWR0|A0A1L1RWR0_CHICK | -2.84844 | down |
| biological_process | localization | tr|A0A3Q2TUF8|A0A3Q2TUF8_CHICK | 1.02023 | up |
| biological_process | localization | tr|A0A3Q2TVV0|A0A3Q2TVV0_CHICK | 1.145618 | up |
| biological_process | localization | tr|A0A3Q2TY77|A0A3Q2TY77_CHICK | 1.82929 | up |
| biological_process | localization | tr|A0A3Q2TYP7|A0A3Q2TYP7_CHICK | 2.159057 | up |
| biological_process | localization | tr|A0A3Q2TZM7|A0A3Q2TZM7_CHICK | 1.419267 | up |
| biological_process | localization | tr|A0A3Q2UCN1|A0A3Q2UCN1_CHICK | 1.92272 | up |
| biological_process | localization | tr|A0A3Q2UD87|A0A3Q2UD87_CHICK | 1.205498 | up |
| biological_process | localization | tr|A0A3Q2UM13|A0A3Q2UM13_CHICK | 3.560203 | up |
| biological_process | localization | tr|A0A3Q3AB84|A0A3Q3AB84_CHICK | 1.170717 | up |
| biological_process | localization | tr|A0A3Q3AQ10|A0A3Q3AQ10_CHICK | 1.000417 | up |
| biological_process | localization | tr|A0A3S5ZPH2|A0A3S5ZPH2_CHICK | 1.677715 | up |
| biological_process | localization | tr|A8VIF4|A8VIF4_CHICK | 1.024165 | up |
| biological_process | localization | tr|D2D3P4|D2D3P4_CHICK | 2.146381 | up |
| biological_process | localization | tr|E1BQC2|E1BQC2_CHICK | -2.52084 | down |
| biological_process | localization | tr|E1BRQ9|E1BRQ9_CHICK | 1.034807 | up |
| biological_process | localization | tr|E1BRR6|E1BRR6_CHICK | 1.332531 | up |
| biological_process | localization | tr|E1BSH7|E1BSH7_CHICK | 1.527514 | up |
| biological_process | localization | tr|E1BTV1|E1BTV1_CHICK | 2.001762 | up |
| biological_process | localization | tr|E1BUX5|E1BUX5_CHICK | 1.099122 | up |
| biological_process | localization | tr|E1BVC8|E1BVC8_CHICK | 2.179244 | up |
| biological_process | localization | tr|E1BVQ3|E1BVQ3_CHICK | 1.650579 | up |
| biological_process | localization | tr|E1BW27|E1BW27_CHICK | 1.938051 | up |
| biological_process | localization | tr|E1BX21|E1BX21_CHICK | 1.42634 | up |
| biological_process | localization | tr|E1BXY8|E1BXY8_CHICK | 1.106549 | up |
| biological_process | localization | tr|E1BY40|E1BY40_CHICK | 1.129106 | up |
| biological_process | localization | tr|E1BYQ3|E1BYQ3_CHICK | 1.202088 | up |
| biological_process | localization | tr|E1C310|E1C310_CHICK | 1.545191 | up |
| biological_process | localization | tr|E1C396|E1C396_CHICK | 1.08258 | up |
| biological_process | localization | tr|E1C593|E1C593_CHICK | 1.922793 | up |
| biological_process | localization | tr|E1C7P7|E1C7P7_CHICK | 2.341246 | up |
| biological_process | localization | tr|E1C8A2|E1C8A2_CHICK | 1.307606 | up |
| biological_process | localization | tr|E1C8U1|E1C8U1_CHICK | 1.411305 | up |
| biological_process | localization | tr|F1N8T0|F1N8T0_CHICK | 1.514696 | up |
| biological_process | localization | tr|F1N8V5|F1N8V5_CHICK | 2.344853 | up |
| biological_process | localization | tr|F1N977|F1N977_CHICK | 1.720618 | up |
| biological_process | localization | tr|F1N9S3|F1N9S3_CHICK | 1.256683 | up |
| biological_process | localization | tr|F1N9U0|F1N9U0_CHICK | 1.038918 | up |
| biological_process | localization | tr|F1NDH2|F1NDH2_CHICK | -1.95135 | down |
| biological_process | localization | tr|F1NF87|F1NF87_CHICK | 1.015859 | up |
| biological_process | localization | tr|F1NMJ9|F1NMJ9_CHICK | -2.4895 | down |
| biological_process | localization | tr|F1NPS5|F1NPS5_CHICK | 3.319197 | up |
| biological_process | localization | tr|F1NQ61|F1NQ61_CHICK | 1.080306 | up |
| biological_process | localization | tr|F1NQP5|F1NQP5_CHICK | 2.898371 | up |
| biological_process | localization | tr|F1NSI7|F1NSI7_CHICK | 1.77407 | up |
| biological_process | localization | tr|F1NSQ4|F1NSQ4_CHICK | 1.21442 | up |
| biological_process | localization | tr|F1NT80|F1NT80_CHICK | 1.404176 | up |
| biological_process | localization | tr|F1NTQ2|F1NTQ2_CHICK | 1.236275 | up |
| biological_process | localization | tr|F1NXV6|F1NXV6_CHICK | -4.87705 | down |
| biological_process | localization | tr|F1P099|F1P099_CHICK | 1.399388 | up |
| biological_process | localization | tr|F1P151|F1P151_CHICK | 2.603153 | up |
| biological_process | localization | tr|F1P204|F1P204_CHICK | 2.129437 | up |
| biological_process | localization | tr|F7BYG6|F7BYG6_CHICK | 3.125856 | up |
| biological_process | localization | tr|H9L0M7|H9L0M7_CHICK | 1.164684 | up |
| biological_process | localization | tr|I1SV11|I1SV11_CHICK | 1.313667 | up |
| biological_process | localization | tr|Q4ADJ6|Q4ADJ6_CHICK | -4.04825 | down |
| biological_process | localization | tr|Q5F3R8|Q5F3R8_CHICK | 2.332553 | up |
| biological_process | localization | tr|Q5F3X1|Q5F3X1_CHICK | 1.19047 | up |
| biological_process | localization | tr|Q5F4B6|Q5F4B6_CHICK | 1.904086 | up |
| biological_process | localization | tr|Q5ZJ19|Q5ZJ19_CHICK | 2.470646 | up |
| biological_process | localization | tr|Q5ZJA8|Q5ZJA8_CHICK | 1.093474 | up |
| biological_process | localization | tr|Q5ZJQ4|Q5ZJQ4_CHICK | 1.678561 | up |
| biological_process | localization | tr|Q5ZL65|Q5ZL65_CHICK | 1.695268 | up |
| biological_process | localization | tr|Q5ZLF6|Q5ZLF6_CHICK | 1.17654 | up |
| biological_process | localization | tr|Q5ZMA7|Q5ZMA7_CHICK | 1.172026 | up |
| biological_process | localization | tr|Q5ZMJ6|Q5ZMJ6_CHICK | 1.845871 | up |
| biological_process | localization | tr|Q5ZMP2|Q5ZMP2_CHICK | 1.165624 | up |
| biological_process | localization | tr|Q9I8C8|Q9I8C8_CHICK | 1.2712 | up |
| biological_process | localization | tr|Q9PUJ4|Q9PUJ4_CHICK | 1.664957 | up |
| biological_process | localization | tr|Q9PVL6|Q9PVL6_CHICK | 1.71949 | up |
| biological_process | localization | tr|R4GHH2|R4GHH2_CHICK | 1.778292 | up |
| biological_process | locomotion | sp|P00523|SRC_CHICK | 1.599352 | up |
| biological_process | locomotion | sp|P42292|CD166_CHICK | 1.297212 | up |
| biological_process | locomotion | sp|Q5ZJ00|EM55_CHICK | 1.664901 | up |
| biological_process | locomotion | sp|Q90733|COT2_CHICK | 1.595685 | up |
| biological_process | locomotion | tr|A0A1D5NUV0|A0A1D5NUV0_CHICK | 2.157037 | up |
| biological_process | locomotion | tr|A0A1D5NZS7|A0A1D5NZS7_CHICK | 1.185694 | up |
| biological_process | locomotion | tr|A0A1D5PWD7|A0A1D5PWD7_CHICK | 1.676687 | up |
| biological_process | locomotion | tr|E1BYQ3|E1BYQ3_CHICK | 1.202088 | up |
| biological_process | locomotion | tr|E1C7P7|E1C7P7_CHICK | 2.341246 | up |
| biological_process | locomotion | tr|F1NDH2|F1NDH2_CHICK | -1.95135 | down |
| biological_process | locomotion | tr|F1NMJ9|F1NMJ9_CHICK | -2.4895 | down |
| biological_process | locomotion | tr|F1NTQ2|F1NTQ2_CHICK | 1.236275 | up |
| biological_process | locomotion | tr|F1P099|F1P099_CHICK | 1.399388 | up |
| biological_process | locomotion | tr|Q5F3X1|Q5F3X1_CHICK | 1.19047 | up |
| biological_process | locomotion | tr|Q5ZMA7|Q5ZMA7_CHICK | 1.172026 | up |
| biological_process | locomotion | tr|Q9PUJ4|Q9PUJ4_CHICK | 1.664957 | up |
| biological_process | metabolic process | sp|O42395|CNBP_CHICK | 1.365125 | up |
| biological_process | metabolic process | sp|O93602|ATF2_CHICK | 2.071233 | up |
| biological_process | metabolic process | sp|P00337|LDHB_CHICK | 2.131478 | up |
| biological_process | metabolic process | sp|P00523|SRC_CHICK | 1.599352 | up |
| biological_process | metabolic process | sp|P00940|TPIS_CHICK | 2.162937 | up |
| biological_process | metabolic process | sp|P02659|APOV1_CHICK | -11.5091 | down |
| biological_process | metabolic process | sp|P17923|NFIA_CHICK | 1.346459 | up |
| biological_process | metabolic process | sp|P18936|NU1M_CHICK | 2.034658 | up |
| biological_process | metabolic process | sp|P20136|GSTM2_CHICK | 2.226935 | up |
| biological_process | metabolic process | sp|P52162|MAX_CHICK | 1.334512 | up |
| biological_process | metabolic process | sp|P81475|CFBL_CHICK | -1.75388 | down |
| biological_process | metabolic process | sp|Q5F3K4|WDR48_CHICK | 1.71564 | up |
| biological_process | metabolic process | sp|Q5F3Z3|UB2V2_CHICK | 2.366484 | up |
| biological_process | metabolic process | sp|Q5F480|ITPK1_CHICK | 1.066359 | up |
| biological_process | metabolic process | sp|Q5ZHN3|WIPI2_CHICK | 1.61465 | up |
| biological_process | metabolic process | sp|Q5ZHT1|ACD11_CHICK | 1.653324 | up |
| biological_process | metabolic process | sp|Q5ZI08|SPT5H_CHICK | 1.235361 | up |
| biological_process | metabolic process | sp|Q5ZI74|DHX30_CHICK | 1.265146 | up |
| biological_process | metabolic process | sp|Q5ZIB9|ANM7_CHICK | 1.168118 | up |
| biological_process | metabolic process | sp|Q5ZIF1|APMAP_CHICK | 1.008442 | up |
| biological_process | metabolic process | sp|Q5ZII9|TFP11_CHICK | 1.172107 | up |
| biological_process | metabolic process | sp|Q5ZIK0|P4K2B_CHICK | 2.036191 | up |
| biological_process | metabolic process | sp|Q5ZIP4|XRN2_CHICK | 1.286156 | up |
| biological_process | metabolic process | sp|Q5ZJ08|SYYC_CHICK | 1.23041 | up |
| biological_process | metabolic process | sp|Q5ZJH9|DKC1_CHICK | 1.436701 | up |
| biological_process | metabolic process | sp|Q5ZK05|TOLIP_CHICK | 1.088317 | up |
| biological_process | metabolic process | sp|Q5ZKV4|NUBP2_CHICK | 1.740378 | up |
| biological_process | metabolic process | sp|Q5ZMG0|DOPD_CHICK | 2.121364 | up |
| biological_process | metabolic process | sp|Q5ZML0|BABA2_CHICK | 1.144731 | up |
| biological_process | metabolic process | sp|Q804X6|FA9_CHICK | -5.71386 | down |
| biological_process | metabolic process | sp|Q8AXL1|SAT1_CHICK | 1.12819 | up |
| biological_process | metabolic process | sp|Q90733|COT2_CHICK | 1.595685 | up |
| biological_process | metabolic process | sp|Q9I8D0|VPP1_CHICK | 1.736096 | up |
| biological_process | metabolic process | sp|Q9IAM7|MRE11_CHICK | 1.337957 | up |
| biological_process | metabolic process | tr|A0A1D5NT70|A0A1D5NT70_CHICK | 1.819215 | up |
| biological_process | metabolic process | tr|A0A1D5NTT2|A0A1D5NTT2_CHICK | 2.201769 | up |
| biological_process | metabolic process | tr|A0A1D5NUD3|A0A1D5NUD3_CHICK | 1.402549 | up |
| biological_process | metabolic process | tr|A0A1D5NUD9|A0A1D5NUD9_CHICK | 1.157212 | up |
| biological_process | metabolic process | tr|A0A1D5NUK5|A0A1D5NUK5_CHICK | 2.534566 | up |
| biological_process | metabolic process | tr|A0A1D5NUV0|A0A1D5NUV0_CHICK | 2.157037 | up |
| biological_process | metabolic process | tr|A0A1D5NZB9|A0A1D5NZB9_CHICK | 1.567222 | up |
| biological_process | metabolic process | tr|A0A1D5NZS7|A0A1D5NZS7_CHICK | 1.185694 | up |
| biological_process | metabolic process | tr|A0A1D5P047|A0A1D5P047_CHICK | 1.659689 | up |
| biological_process | metabolic process | tr|A0A1D5P1D5|A0A1D5P1D5_CHICK | 2.084726 | up |
| biological_process | metabolic process | tr|A0A1D5P4B1|A0A1D5P4B1_CHICK | 1.156059 | up |
| biological_process | metabolic process | tr|A0A1D5P607|A0A1D5P607_CHICK | 1.51904 | up |
| biological_process | metabolic process | tr|A0A1D5P6C3|A0A1D5P6C3_CHICK | 2.100747 | up |
| biological_process | metabolic process | tr|A0A1D5P6L7|A0A1D5P6L7_CHICK | 1.520092 | up |
| biological_process | metabolic process | tr|A0A1D5P6M8|A0A1D5P6M8_CHICK | 1.82889 | up |
| biological_process | metabolic process | tr|A0A1D5P6S3|A0A1D5P6S3_CHICK | 1.183172 | up |
| biological_process | metabolic process | tr|A0A1D5P6Y6|A0A1D5P6Y6_CHICK | 1.32218 | up |
| biological_process | metabolic process | tr|A0A1D5P7C5|A0A1D5P7C5_CHICK | 1.273318 | up |
| biological_process | metabolic process | tr|A0A1D5P8H7|A0A1D5P8H7_CHICK | 1.530513 | up |
| biological_process | metabolic process | tr|A0A1D5PA08|A0A1D5PA08_CHICK | 1.946165 | up |
| biological_process | metabolic process | tr|A0A1D5PAM1|A0A1D5PAM1_CHICK | 2.577291 | up |
| biological_process | metabolic process | tr|A0A1D5PC23|A0A1D5PC23_CHICK | 2.344267 | up |
| biological_process | metabolic process | tr|A0A1D5PCZ1|A0A1D5PCZ1_CHICK | 1.542638 | up |
| biological_process | metabolic process | tr|A0A1D5PFS2|A0A1D5PFS2_CHICK | 1.463999 | up |
| biological_process | metabolic process | tr|A0A1D5PFX4|A0A1D5PFX4_CHICK | 1.061266 | up |
| biological_process | metabolic process | tr|A0A1D5PG53|A0A1D5PG53_CHICK | 2.149242 | up |
| biological_process | metabolic process | tr|A0A1D5PGB1|A0A1D5PGB1_CHICK | 1.43532 | up |
| biological_process | metabolic process | tr|A0A1D5PHE2|A0A1D5PHE2_CHICK | 2.577604 | up |
| biological_process | metabolic process | tr|A0A1D5PIB1|A0A1D5PIB1_CHICK | 2.10824 | up |
| biological_process | metabolic process | tr|A0A1D5PIT4|A0A1D5PIT4_CHICK | -1.97988 | down |
| biological_process | metabolic process | tr|A0A1D5PK48|A0A1D5PK48_CHICK | -5.8308 | down |
| biological_process | metabolic process | tr|A0A1D5PQ66|A0A1D5PQ66_CHICK | 1.664552 | up |
| biological_process | metabolic process | tr|A0A1D5PR34|A0A1D5PR34_CHICK | 1.370909 | up |
| biological_process | metabolic process | tr|A0A1D5PRI4|A0A1D5PRI4_CHICK | 1.28486 | up |
| biological_process | metabolic process | tr|A0A1D5PRI6|A0A1D5PRI6_CHICK | 1.580404 | up |
| biological_process | metabolic process | tr|A0A1D5PTW3|A0A1D5PTW3_CHICK | 1.808599 | up |
| biological_process | metabolic process | tr|A0A1D5PWD7|A0A1D5PWD7_CHICK | 1.676687 | up |
| biological_process | metabolic process | tr|A0A1D5PWT4|A0A1D5PWT4_CHICK | 1.101328 | up |
| biological_process | metabolic process | tr|A0A1D5PYB7|A0A1D5PYB7_CHICK | 1.489522 | up |
| biological_process | metabolic process | tr|A0A1D5Q008|A0A1D5Q008_CHICK | 2.962255 | up |
| biological_process | metabolic process | tr|A0A1L1RPD1|A0A1L1RPD1_CHICK | 1.396669 | up |
| biological_process | metabolic process | tr|A0A1L1RSS5|A0A1L1RSS5_CHICK | 1.143155 | up |
| biological_process | metabolic process | tr|A0A1L1RWR0|A0A1L1RWR0_CHICK | -2.84844 | down |
| biological_process | metabolic process | tr|A0A1L1RXA3|A0A1L1RXA3_CHICK | 1.394002 | up |
| biological_process | metabolic process | tr|A0A286QZR1|A0A286QZR1_CHICK | 1.043669 | up |
| biological_process | metabolic process | tr|A0A3Q2TS24|A0A3Q2TS24_CHICK | 1.398629 | up |
| biological_process | metabolic process | tr|A0A3Q2TVU0|A0A3Q2TVU0_CHICK | 1.280793 | up |
| biological_process | metabolic process | tr|A0A3Q2TVV0|A0A3Q2TVV0_CHICK | 1.145618 | up |
| biological_process | metabolic process | tr|A0A3Q2TZM7|A0A3Q2TZM7_CHICK | 1.419267 | up |
| biological_process | metabolic process | tr|A0A3Q2U8I0|A0A3Q2U8I0_CHICK | 1.050068 | up |
| biological_process | metabolic process | tr|A0A3Q2UB58|A0A3Q2UB58_CHICK | 2.070855 | up |
| biological_process | metabolic process | tr|A0A3Q2UBJ7|A0A3Q2UBJ7_CHICK | 1.945958 | up |
| biological_process | metabolic process | tr|A0A3Q2UCG6|A0A3Q2UCG6_CHICK | 1.033099 | up |
| biological_process | metabolic process | tr|A0A3Q2UCN1|A0A3Q2UCN1_CHICK | 1.92272 | up |
| biological_process | metabolic process | tr|A0A3Q2UD87|A0A3Q2UD87_CHICK | 1.205498 | up |
| biological_process | metabolic process | tr|A0A3Q2UK74|A0A3Q2UK74_CHICK | 2.254776 | up |
| biological_process | metabolic process | tr|A0A3Q3ALC8|A0A3Q3ALC8_CHICK | 2.188996 | up |
| biological_process | metabolic process | tr|A0A3Q3APG2|A0A3Q3APG2_CHICK | 1.640987 | up |
| biological_process | metabolic process | tr|A8VIF4|A8VIF4_CHICK | 1.024165 | up |
| biological_process | metabolic process | tr|B3VE14|B3VE14_CHICK | -1.63112 | down |
| biological_process | metabolic process | tr|D2D3P4|D2D3P4_CHICK | 2.146381 | up |
| biological_process | metabolic process | tr|E1BQI0|E1BQI0_CHICK | 1.067708 | up |
| biological_process | metabolic process | tr|E1BQU2|E1BQU2_CHICK | 1.629018 | up |
| biological_process | metabolic process | tr|E1BQW2|E1BQW2_CHICK | 1.823966 | up |
| biological_process | metabolic process | tr|E1BRQ9|E1BRQ9_CHICK | 1.034807 | up |
| biological_process | metabolic process | tr|E1BRT3|E1BRT3_CHICK | 2.214419 | up |
| biological_process | metabolic process | tr|E1BS94|E1BS94_CHICK | 1.70074 | up |
| biological_process | metabolic process | tr|E1BSH7|E1BSH7_CHICK | 1.527514 | up |
| biological_process | metabolic process | tr|E1BT44|E1BT44_CHICK | 1.432225 | up |
| biological_process | metabolic process | tr|E1BT94|E1BT94_CHICK | 1.49966 | up |
| biological_process | metabolic process | tr|E1BTS3|E1BTS3_CHICK | 1.090627 | up |
| biological_process | metabolic process | tr|E1BTT8|E1BTT8_CHICK | 1.708105 | up |
| biological_process | metabolic process | tr|E1BU27|E1BU27_CHICK | 3.035848 | up |
| biological_process | metabolic process | tr|E1BU89|E1BU89_CHICK | 1.303565 | up |
| biological_process | metabolic process | tr|E1BUJ1|E1BUJ1_CHICK | 2.709557 | up |
| biological_process | metabolic process | tr|E1BV97|E1BV97_CHICK | 2.036483 | up |
| biological_process | metabolic process | tr|E1BVQ3|E1BVQ3_CHICK | 1.650579 | up |
| biological_process | metabolic process | tr|E1BWG7|E1BWG7_CHICK | 2.421508 | up |
| biological_process | metabolic process | tr|E1BX21|E1BX21_CHICK | 1.42634 | up |
| biological_process | metabolic process | tr|E1BX85|E1BX85_CHICK | 2.029343 | up |
| biological_process | metabolic process | tr|E1BXC2|E1BXC2_CHICK | 1.767478 | up |
| biological_process | metabolic process | tr|E1BY22|E1BY22_CHICK | 1.367672 | up |
| biological_process | metabolic process | tr|E1BYQ3|E1BYQ3_CHICK | 1.202088 | up |
| biological_process | metabolic process | tr|E1BZ79|E1BZ79_CHICK | 2.388844 | up |
| biological_process | metabolic process | tr|E1C1T2|E1C1T2_CHICK | 1.594659 | up |
| biological_process | metabolic process | tr|E1C1Y5|E1C1Y5_CHICK | 1.135922 | up |
| biological_process | metabolic process | tr|E1C229|E1C229_CHICK | 1.473036 | up |
| biological_process | metabolic process | tr|E1C2E5|E1C2E5_CHICK | 1.182299 | up |
| biological_process | metabolic process | tr|E1C312|E1C312_CHICK | 1.811403 | up |
| biological_process | metabolic process | tr|E1C396|E1C396_CHICK | 1.08258 | up |
| biological_process | metabolic process | tr|E1C3U6|E1C3U6_CHICK | 1.144268 | up |
| biological_process | metabolic process | tr|E1C688|E1C688_CHICK | 1.775755 | up |
| biological_process | metabolic process | tr|E1C6S7|E1C6S7_CHICK | 1.1895 | up |
| biological_process | metabolic process | tr|E1C7P7|E1C7P7_CHICK | 2.341246 | up |
| biological_process | metabolic process | tr|E1C8U1|E1C8U1_CHICK | 1.411305 | up |
| biological_process | metabolic process | tr|E1C9H5|E1C9H5_CHICK | 1.195027 | up |
| biological_process | metabolic process | tr|E7EC82|E7EC82_CHICK | 1.586027 | up |
| biological_process | metabolic process | tr|F1N832|F1N832_CHICK | 1.427435 | up |
| biological_process | metabolic process | tr|F1N9S3|F1N9S3_CHICK | 1.256683 | up |
| biological_process | metabolic process | tr|F1N9U0|F1N9U0_CHICK | 1.038918 | up |
| biological_process | metabolic process | tr|F1N9Y3|F1N9Y3_CHICK | 1.33841 | up |
| biological_process | metabolic process | tr|F1NBX1|F1NBX1_CHICK | 1.439614 | up |
| biological_process | metabolic process | tr|F1NDH2|F1NDH2_CHICK | -1.95135 | down |
| biological_process | metabolic process | tr|F1NE88|F1NE88_CHICK | 1.362851 | up |
| biological_process | metabolic process | tr|F1NEQ3|F1NEQ3_CHICK | 1.139952 | up |
| biological_process | metabolic process | tr|F1NIQ3|F1NIQ3_CHICK | 1.483149 | up |
| biological_process | metabolic process | tr|F1NIY3|F1NIY3_CHICK | 1.568578 | up |
| biological_process | metabolic process | tr|F1NJU5|F1NJU5_CHICK | -1.49981 | down |
| biological_process | metabolic process | tr|F1NLM0|F1NLM0_CHICK | 1.884615 | up |
| biological_process | metabolic process | tr|F1NMJ9|F1NMJ9_CHICK | -2.4895 | down |
| biological_process | metabolic process | tr|F1NMM9|F1NMM9_CHICK | 1.449446 | up |
| biological_process | metabolic process | tr|F1NMQ3|F1NMQ3_CHICK | 1.885262 | up |
| biological_process | metabolic process | tr|F1NMU0|F1NMU0_CHICK | 2.467729 | up |
| biological_process | metabolic process | tr|F1NN63|F1NN63_CHICK | 1.72615 | up |
| biological_process | metabolic process | tr|F1NPS5|F1NPS5_CHICK | 3.319197 | up |
| biological_process | metabolic process | tr|F1NQ61|F1NQ61_CHICK | 1.080306 | up |
| biological_process | metabolic process | tr|F1NR86|F1NR86_CHICK | 1.817533 | up |
| biological_process | metabolic process | tr|F1NRZ4|F1NRZ4_CHICK | 1.14911 | up |
| biological_process | metabolic process | tr|F1NSF2|F1NSF2_CHICK | 1.708711 | up |
| biological_process | metabolic process | tr|F1NT58|F1NT58_CHICK | 1.701877 | up |
| biological_process | metabolic process | tr|F1NT80|F1NT80_CHICK | 1.404176 | up |
| biological_process | metabolic process | tr|F1NTQ2|F1NTQ2_CHICK | 1.236275 | up |
| biological_process | metabolic process | tr|F1NUY9|F1NUY9_CHICK | 1.592258 | up |
| biological_process | metabolic process | tr|F1NV66|F1NV66_CHICK | 1.865555 | up |
| biological_process | metabolic process | tr|F1NXV6|F1NXV6_CHICK | -4.87705 | down |
| biological_process | metabolic process | tr|F1NXZ7|F1NXZ7_CHICK | 1.050248 | up |
| biological_process | metabolic process | tr|F1NYG7|F1NYG7_CHICK | 1.537967 | up |
| biological_process | metabolic process | tr|F1P099|F1P099_CHICK | 1.399388 | up |
| biological_process | metabolic process | tr|F1P2D2|F1P2D2_CHICK | 1.35416 | up |
| biological_process | metabolic process | tr|F1P2G6|F1P2G6_CHICK | 1.470473 | up |
| biological_process | metabolic process | tr|F1P372|F1P372_CHICK | 1.525043 | up |
| biological_process | metabolic process | tr|F1P394|F1P394_CHICK | 1.286828 | up |
| biological_process | metabolic process | tr|F1P526|F1P526_CHICK | 1.506479 | up |
| biological_process | metabolic process | tr|F1P5A3|F1P5A3_CHICK | 2.127136 | up |
| biological_process | metabolic process | tr|F1P5J7|F1P5J7_CHICK | 1.075835 | up |
| biological_process | metabolic process | tr|F1P5S5|F1P5S5_CHICK | 1.528046 | up |
| biological_process | metabolic process | tr|F1P5X6|F1P5X6_CHICK | 1.295882 | up |
| biological_process | metabolic process | tr|F6R228|F6R228_CHICK | 1.426396 | up |
| biological_process | metabolic process | tr|F7BFS9|F7BFS9_CHICK | 1.641443 | up |
| biological_process | metabolic process | tr|I1SV11|I1SV11_CHICK | 1.313667 | up |
| biological_process | metabolic process | tr|Q2PUH1|Q2PUH1_CHICK | 1.468226 | up |
| biological_process | metabolic process | tr|Q5DWF6|Q5DWF6_CHICK | 1.289716 | up |
| biological_process | metabolic process | tr|Q5F351|Q5F351_CHICK | 1.342446 | up |
| biological_process | metabolic process | tr|Q5F3R8|Q5F3R8_CHICK | 2.332553 | up |
| biological_process | metabolic process | tr|Q5F3X1|Q5F3X1_CHICK | 1.19047 | up |
| biological_process | metabolic process | tr|Q5F420|Q5F420_CHICK | 1.387816 | up |
| biological_process | metabolic process | tr|Q5F4B6|Q5F4B6_CHICK | 1.904086 | up |
| biological_process | metabolic process | tr|Q5ZHP2|Q5ZHP2_CHICK | 2.412922 | up |
| biological_process | metabolic process | tr|Q5ZIL3|Q5ZIL3_CHICK | 1.863958 | up |
| biological_process | metabolic process | tr|Q5ZIZ3|Q5ZIZ3_CHICK | 1.248317 | up |
| biological_process | metabolic process | tr|Q5ZJ96|Q5ZJ96_CHICK | 1.306532 | up |
| biological_process | metabolic process | tr|Q5ZJQ4|Q5ZJQ4_CHICK | 1.678561 | up |
| biological_process | metabolic process | tr|Q5ZJW2|Q5ZJW2_CHICK | 1.71086 | up |
| biological_process | metabolic process | tr|Q5ZKI3|Q5ZKI3_CHICK | 1.326993 | up |
| biological_process | metabolic process | tr|Q5ZLI0|Q5ZLI0_CHICK | 1.097879 | up |
| biological_process | metabolic process | tr|Q5ZMA7|Q5ZMA7_CHICK | 1.172026 | up |
| biological_process | metabolic process | tr|Q5ZMJ6|Q5ZMJ6_CHICK | 1.845871 | up |
| biological_process | metabolic process | tr|Q7ZTS9|Q7ZTS9_CHICK | 1.923559 | up |
| biological_process | metabolic process | tr|R4GI86|R4GI86_CHICK | 2.078653 | up |
| biological_process | metabolic process | tr|V9GW30|V9GW30_CHICK | 1.560922 | up |
| biological_process | multi-organism process | sp|Q5F3K4|WDR48_CHICK | 1.71564 | up |
| biological_process | multi-organism process | sp|Q5ZHN3|WIPI2_CHICK | 1.61465 | up |
| biological_process | multi-organism process | sp|Q5ZIB9|ANM7_CHICK | 1.168118 | up |
| biological_process | multi-organism process | sp|Q5ZIP4|XRN2_CHICK | 1.286156 | up |
| biological_process | multi-organism process | sp|Q90733|COT2_CHICK | 1.595685 | up |
| biological_process | multi-organism process | tr|A0A1D5NXG4|A0A1D5NXG4_CHICK | 1.245324 | up |
| biological_process | multi-organism process | tr|A0A1D5P453|A0A1D5P453_CHICK | 1.553695 | up |
| biological_process | multi-organism process | tr|A0A1D5PFS2|A0A1D5PFS2_CHICK | 1.463999 | up |
| biological_process | multi-organism process | tr|A0A1D5PIT4|A0A1D5PIT4_CHICK | -1.97988 | down |
| biological_process | multi-organism process | tr|E1BQC2|E1BQC2_CHICK | -2.52084 | down |
| biological_process | multi-organism process | tr|E1BQU2|E1BQU2_CHICK | 1.629018 | up |
| biological_process | multi-organism process | tr|E1BS94|E1BS94_CHICK | 1.70074 | up |
| biological_process | multi-organism process | tr|E1BTV1|E1BTV1_CHICK | 2.001762 | up |
| biological_process | multi-organism process | tr|E1BX21|E1BX21_CHICK | 1.42634 | up |
| biological_process | multi-organism process | tr|E1C592|E1C592_CHICK | 1.565005 | up |
| biological_process | multi-organism process | tr|E1C688|E1C688_CHICK | 1.775755 | up |
| biological_process | multi-organism process | tr|E7EC82|E7EC82_CHICK | 1.586027 | up |
| biological_process | multi-organism process | tr|F1NAS0|F1NAS0_CHICK | 1.779766 | up |
| biological_process | multi-organism process | tr|F1NIY3|F1NIY3_CHICK | 1.568578 | up |
| biological_process | multi-organism process | tr|F1NTQ2|F1NTQ2_CHICK | 1.236275 | up |
| biological_process | multi-organism process | tr|F1NXV6|F1NXV6_CHICK | -4.87705 | down |
| biological_process | multi-organism process | tr|I1SV11|I1SV11_CHICK | 1.313667 | up |
| biological_process | multi-organism process | tr|Q4ADJ6|Q4ADJ6_CHICK | -4.04825 | down |
| biological_process | multi-organism process | tr|Q5F3X1|Q5F3X1_CHICK | 1.19047 | up |
| biological_process | multi-organism process | tr|Q5ZL65|Q5ZL65_CHICK | 1.695268 | up |
| biological_process | multicellular organismal process | sp|P00523|SRC_CHICK | 1.599352 | up |
| biological_process | multicellular organismal process | sp|P02197|MYG_CHICK | 2.885059 | up |
| biological_process | multicellular organismal process | sp|P25155|FA10_CHICK | -3.77432 | down |
| biological_process | multicellular organismal process | sp|P42292|CD166_CHICK | 1.297212 | up |
| biological_process | multicellular organismal process | sp|Q05423|FABP7_CHICK | 1.9874 | up |
| biological_process | multicellular organismal process | sp|Q5F3K4|WDR48_CHICK | 1.71564 | up |
| biological_process | multicellular organismal process | sp|Q5ZIB9|ANM7_CHICK | 1.168118 | up |
| biological_process | multicellular organismal process | sp|Q5ZIP4|XRN2_CHICK | 1.286156 | up |
| biological_process | multicellular organismal process | sp|Q5ZK92|SPAST_CHICK | 1.665787 | up |
| biological_process | multicellular organismal process | sp|Q5ZL36|ZFY27_CHICK | 1.948763 | up |
| biological_process | multicellular organismal process | sp|Q804X6|FA9_CHICK | -5.71386 | down |
| biological_process | multicellular organismal process | sp|Q8AXL1|SAT1_CHICK | 1.12819 | up |
| biological_process | multicellular organismal process | sp|Q90733|COT2_CHICK | 1.595685 | up |
| biological_process | multicellular organismal process | tr|A0A1D5NUV0|A0A1D5NUV0_CHICK | 2.157037 | up |
| biological_process | multicellular organismal process | tr|A0A1D5NZB4|A0A1D5NZB4_CHICK | 1.671702 | up |
| biological_process | multicellular organismal process | tr|A0A1D5NZS7|A0A1D5NZS7_CHICK | 1.185694 | up |
| biological_process | multicellular organismal process | tr|A0A1D5P6C3|A0A1D5P6C3_CHICK | 2.100747 | up |
| biological_process | multicellular organismal process | tr|A0A1D5PA08|A0A1D5PA08_CHICK | 1.946165 | up |
| biological_process | multicellular organismal process | tr|A0A1D5PIB1|A0A1D5PIB1_CHICK | 2.10824 | up |
| biological_process | multicellular organismal process | tr|A0A1D5PIT4|A0A1D5PIT4_CHICK | -1.97988 | down |
| biological_process | multicellular organismal process | tr|A0A1D5PQC1|A0A1D5PQC1_CHICK | 1.667257 | up |
| biological_process | multicellular organismal process | tr|A0A1D5PQD0|A0A1D5PQD0_CHICK | 1.314273 | up |
| biological_process | multicellular organismal process | tr|A0A1D5PTE8|A0A1D5PTE8_CHICK | 1.960486 | up |
| biological_process | multicellular organismal process | tr|A0A1D5PWD7|A0A1D5PWD7_CHICK | 1.676687 | up |
| biological_process | multicellular organismal process | tr|A0A1L1RPB1|A0A1L1RPB1_CHICK | 1.864507 | up |
| biological_process | multicellular organismal process | tr|A0A3Q2TUF8|A0A3Q2TUF8_CHICK | 1.02023 | up |
| biological_process | multicellular organismal process | tr|A0A3Q2TVV0|A0A3Q2TVV0_CHICK | 1.145618 | up |
| biological_process | multicellular organismal process | tr|A0A3Q2U504|A0A3Q2U504_CHICK | -2.35777 | down |
| biological_process | multicellular organismal process | tr|A0A3Q2U8I0|A0A3Q2U8I0_CHICK | 1.050068 | up |
| biological_process | multicellular organismal process | tr|A0A3Q2UBJ7|A0A3Q2UBJ7_CHICK | 1.945958 | up |
| biological_process | multicellular organismal process | tr|A0A3Q2UCN1|A0A3Q2UCN1_CHICK | 1.92272 | up |
| biological_process | multicellular organismal process | tr|A0A3Q2UD87|A0A3Q2UD87_CHICK | 1.205498 | up |
| biological_process | multicellular organismal process | tr|A0A3Q2UPF3|A0A3Q2UPF3_CHICK | 1.295517 | up |
| biological_process | multicellular organismal process | tr|A0A3Q3ALC8|A0A3Q3ALC8_CHICK | 2.188996 | up |
| biological_process | multicellular organismal process | tr|A8VIF4|A8VIF4_CHICK | 1.024165 | up |
| biological_process | multicellular organismal process | tr|D2D3P4|D2D3P4_CHICK | 2.146381 | up |
| biological_process | multicellular organismal process | tr|E1BQU2|E1BQU2_CHICK | 1.629018 | up |
| biological_process | multicellular organismal process | tr|E1BSH7|E1BSH7_CHICK | 1.527514 | up |
| biological_process | multicellular organismal process | tr|E1BTE2|E1BTE2_CHICK | -2.67874 | down |
| biological_process | multicellular organismal process | tr|E1BU27|E1BU27_CHICK | 3.035848 | up |
| biological_process | multicellular organismal process | tr|E1BVQ3|E1BVQ3_CHICK | 1.650579 | up |
| biological_process | multicellular organismal process | tr|E1BX21|E1BX21_CHICK | 1.42634 | up |
| biological_process | multicellular organismal process | tr|E1BY22|E1BY22_CHICK | 1.367672 | up |
| biological_process | multicellular organismal process | tr|E1BY40|E1BY40_CHICK | 1.129106 | up |
| biological_process | multicellular organismal process | tr|E1BYQ3|E1BYQ3_CHICK | 1.202088 | up |
| biological_process | multicellular organismal process | tr|E1C310|E1C310_CHICK | 1.545191 | up |
| biological_process | multicellular organismal process | tr|E1C3U6|E1C3U6_CHICK | 1.144268 | up |
| biological_process | multicellular organismal process | tr|E1C688|E1C688_CHICK | 1.775755 | up |
| biological_process | multicellular organismal process | tr|E1C7H6|E1C7H6_CHICK | -1.05537 | down |
| biological_process | multicellular organismal process | tr|E7EC82|E7EC82_CHICK | 1.586027 | up |
| biological_process | multicellular organismal process | tr|F1N9Y3|F1N9Y3_CHICK | 1.33841 | up |
| biological_process | multicellular organismal process | tr|F1NDH2|F1NDH2_CHICK | -1.95135 | down |
| biological_process | multicellular organismal process | tr|F1NIY3|F1NIY3_CHICK | 1.568578 | up |
| biological_process | multicellular organismal process | tr|F1NMJ9|F1NMJ9_CHICK | -2.4895 | down |
| biological_process | multicellular organismal process | tr|F1NRZ4|F1NRZ4_CHICK | 1.14911 | up |
| biological_process | multicellular organismal process | tr|F1NT80|F1NT80_CHICK | 1.404176 | up |
| biological_process | multicellular organismal process | tr|F1NTQ2|F1NTQ2_CHICK | 1.236275 | up |
| biological_process | multicellular organismal process | tr|F1NXV6|F1NXV6_CHICK | -4.87705 | down |
| biological_process | multicellular organismal process | tr|F1P099|F1P099_CHICK | 1.399388 | up |
| biological_process | multicellular organismal process | tr|F7BFS9|F7BFS9_CHICK | 1.641443 | up |
| biological_process | multicellular organismal process | tr|I1SV11|I1SV11_CHICK | 1.313667 | up |
| biological_process | multicellular organismal process | tr|O93466|O93466_CHICK | 1.669881 | up |
| biological_process | multicellular organismal process | tr|Q2PUH1|Q2PUH1_CHICK | 1.468226 | up |
| biological_process | multicellular organismal process | tr|Q5DWF6|Q5DWF6_CHICK | 1.289716 | up |
| biological_process | multicellular organismal process | tr|Q5F3X1|Q5F3X1_CHICK | 1.19047 | up |
| biological_process | multicellular organismal process | tr|Q5ZKI3|Q5ZKI3_CHICK | 1.326993 | up |
| biological_process | multicellular organismal process | tr|Q9DDD4|Q9DDD4_CHICK | -1.44831 | down |
| biological_process | multicellular organismal process | tr|Q9PUJ4|Q9PUJ4_CHICK | 1.664957 | up |
| biological_process | multicellular organismal process | tr|R4GMH5|R4GMH5_CHICK | -2.32193 | down |
| biological_process | negative regulation of biological process | sp|P00523|SRC_CHICK | 1.599352 | up |
| biological_process | negative regulation of biological process | sp|Q05423|FABP7_CHICK | 1.9874 | up |
| biological_process | negative regulation of biological process | sp|Q5ZI08|SPT5H_CHICK | 1.235361 | up |
| biological_process | negative regulation of biological process | sp|Q5ZII9|TFP11_CHICK | 1.172107 | up |
| biological_process | negative regulation of biological process | sp|Q5ZML0|BABA2_CHICK | 1.144731 | up |
| biological_process | negative regulation of biological process | sp|Q90733|COT2_CHICK | 1.595685 | up |
| biological_process | negative regulation of biological process | sp|Q9IAM7|MRE11_CHICK | 1.337957 | up |
| biological_process | negative regulation of biological process | tr|A0A1D5NUD9|A0A1D5NUD9_CHICK | 1.157212 | up |
| biological_process | negative regulation of biological process | tr|A0A1D5NUV0|A0A1D5NUV0_CHICK | 2.157037 | up |
| biological_process | negative regulation of biological process | tr|A0A1D5NZS7|A0A1D5NZS7_CHICK | 1.185694 | up |
| biological_process | negative regulation of biological process | tr|A0A1D5P047|A0A1D5P047_CHICK | 1.659689 | up |
| biological_process | negative regulation of biological process | tr|A0A1D5PA08|A0A1D5PA08_CHICK | 1.946165 | up |
| biological_process | negative regulation of biological process | tr|A0A1D5PFS2|A0A1D5PFS2_CHICK | 1.463999 | up |
| biological_process | negative regulation of biological process | tr|A0A1D5PIT4|A0A1D5PIT4_CHICK | -1.97988 | down |
| biological_process | negative regulation of biological process | tr|A0A1D5PQD0|A0A1D5PQD0_CHICK | 1.314273 | up |
| biological_process | negative regulation of biological process | tr|A0A1D5PR34|A0A1D5PR34_CHICK | 1.370909 | up |
| biological_process | negative regulation of biological process | tr|A0A1D5PTE8|A0A1D5PTE8_CHICK | 1.960486 | up |
| biological_process | negative regulation of biological process | tr|A0A1D5PUJ5|A0A1D5PUJ5_CHICK | 1.266991 | up |
| biological_process | negative regulation of biological process | tr|A0A1D5PYB7|A0A1D5PYB7_CHICK | 1.489522 | up |
| biological_process | negative regulation of biological process | tr|A0A3Q2TVU0|A0A3Q2TVU0_CHICK | 1.280793 | up |
| biological_process | negative regulation of biological process | tr|A0A3Q2TZM7|A0A3Q2TZM7_CHICK | 1.419267 | up |
| biological_process | negative regulation of biological process | tr|A0A3Q2U504|A0A3Q2U504_CHICK | -2.35777 | down |
| biological_process | negative regulation of biological process | tr|A0A3Q2UBJ7|A0A3Q2UBJ7_CHICK | 1.945958 | up |
| biological_process | negative regulation of biological process | tr|A0A3Q2UCN1|A0A3Q2UCN1_CHICK | 1.92272 | up |
| biological_process | negative regulation of biological process | tr|A0A3Q3ALC8|A0A3Q3ALC8_CHICK | 2.188996 | up |
| biological_process | negative regulation of biological process | tr|A8VIF4|A8VIF4_CHICK | 1.024165 | up |
| biological_process | negative regulation of biological process | tr|E1BSH7|E1BSH7_CHICK | 1.527514 | up |
| biological_process | negative regulation of biological process | tr|E1BU89|E1BU89_CHICK | 1.303565 | up |
| biological_process | negative regulation of biological process | tr|E1BX21|E1BX21_CHICK | 1.42634 | up |
| biological_process | negative regulation of biological process | tr|E1BY40|E1BY40_CHICK | 1.129106 | up |
| biological_process | negative regulation of biological process | tr|E1BYQ3|E1BYQ3_CHICK | 1.202088 | up |
| biological_process | negative regulation of biological process | tr|E1BZ79|E1BZ79_CHICK | 2.388844 | up |
| biological_process | negative regulation of biological process | tr|E1C396|E1C396_CHICK | 1.08258 | up |
| biological_process | negative regulation of biological process | tr|E1C3U6|E1C3U6_CHICK | 1.144268 | up |
| biological_process | negative regulation of biological process | tr|E1C688|E1C688_CHICK | 1.775755 | up |
| biological_process | negative regulation of biological process | tr|E1C6S7|E1C6S7_CHICK | 1.1895 | up |
| biological_process | negative regulation of biological process | tr|E1C7H6|E1C7H6_CHICK | -1.05537 | down |
| biological_process | negative regulation of biological process | tr|E1C7P7|E1C7P7_CHICK | 2.341246 | up |
| biological_process | negative regulation of biological process | tr|E7EC82|E7EC82_CHICK | 1.586027 | up |
| biological_process | negative regulation of biological process | tr|F1N9Y3|F1N9Y3_CHICK | 1.33841 | up |
| biological_process | negative regulation of biological process | tr|F1NDH2|F1NDH2_CHICK | -1.95135 | down |
| biological_process | negative regulation of biological process | tr|F1NIQ3|F1NIQ3_CHICK | 1.483149 | up |
| biological_process | negative regulation of biological process | tr|F1NIY3|F1NIY3_CHICK | 1.568578 | up |
| biological_process | negative regulation of biological process | tr|F1NPS5|F1NPS5_CHICK | 3.319197 | up |
| biological_process | negative regulation of biological process | tr|F1NR86|F1NR86_CHICK | 1.817533 | up |
| biological_process | negative regulation of biological process | tr|F1NRZ4|F1NRZ4_CHICK | 1.14911 | up |
| biological_process | negative regulation of biological process | tr|F1NSF2|F1NSF2_CHICK | 1.708711 | up |
| biological_process | negative regulation of biological process | tr|F1NT80|F1NT80_CHICK | 1.404176 | up |
| biological_process | negative regulation of biological process | tr|F1NXV6|F1NXV6_CHICK | -4.87705 | down |
| biological_process | negative regulation of biological process | tr|F1P394|F1P394_CHICK | 1.286828 | up |
| biological_process | negative regulation of biological process | tr|F1P5A3|F1P5A3_CHICK | 2.127136 | up |
| biological_process | negative regulation of biological process | tr|I1SV11|I1SV11_CHICK | 1.313667 | up |
| biological_process | negative regulation of biological process | tr|Q2PUH1|Q2PUH1_CHICK | 1.468226 | up |
| biological_process | negative regulation of biological process | tr|Q5DWF6|Q5DWF6_CHICK | 1.289716 | up |
| biological_process | negative regulation of biological process | tr|Q5F3X1|Q5F3X1_CHICK | 1.19047 | up |
| biological_process | negative regulation of biological process | tr|Q5ZJQ4|Q5ZJQ4_CHICK | 1.678561 | up |
| biological_process | negative regulation of biological process | tr|Q5ZKI3|Q5ZKI3_CHICK | 1.326993 | up |
| biological_process | negative regulation of biological process | tr|Q5ZMA7|Q5ZMA7_CHICK | 1.172026 | up |
| biological_process | negative regulation of biological process | tr|Q5ZMJ6|Q5ZMJ6_CHICK | 1.845871 | up |
| biological_process | negative regulation of biological process | tr|Q9PUJ4|Q9PUJ4_CHICK | 1.664957 | up |
| biological_process | negative regulation of biological process | tr|R4GMH5|R4GMH5_CHICK | -2.32193 | down |
| biological_process | pigmentation | tr|D2D3P4|D2D3P4_CHICK | 2.146381 | up |
| biological_process | pigmentation | tr|Q5F3R8|Q5F3R8_CHICK | 2.332553 | up |
| biological_process | positive regulation of biological process | sp|O42395|CNBP_CHICK | 1.365125 | up |
| biological_process | positive regulation of biological process | sp|P25155|FA10_CHICK | -3.77432 | down |
| biological_process | positive regulation of biological process | sp|P81475|CFBL_CHICK | -1.75388 | down |
| biological_process | positive regulation of biological process | sp|Q5F3K4|WDR48_CHICK | 1.71564 | up |
| biological_process | positive regulation of biological process | sp|Q5ZI08|SPT5H_CHICK | 1.235361 | up |
| biological_process | positive regulation of biological process | sp|Q5ZK92|SPAST_CHICK | 1.665787 | up |
| biological_process | positive regulation of biological process | sp|Q5ZL36|ZFY27_CHICK | 1.948763 | up |
| biological_process | positive regulation of biological process | sp|Q5ZMG0|DOPD_CHICK | 2.121364 | up |
| biological_process | positive regulation of biological process | sp|Q5ZML0|BABA2_CHICK | 1.144731 | up |
| biological_process | positive regulation of biological process | sp|Q90733|COT2_CHICK | 1.595685 | up |
| biological_process | positive regulation of biological process | sp|Q9IAM7|MRE11_CHICK | 1.337957 | up |
| biological_process | positive regulation of biological process | tr|A0A1D5NUD9|A0A1D5NUD9_CHICK | 1.157212 | up |
| biological_process | positive regulation of biological process | tr|A0A1D5NUV0|A0A1D5NUV0_CHICK | 2.157037 | up |
| biological_process | positive regulation of biological process | tr|A0A1D5P6C3|A0A1D5P6C3_CHICK | 2.100747 | up |
| biological_process | positive regulation of biological process | tr|A0A1D5PA08|A0A1D5PA08_CHICK | 1.946165 | up |
| biological_process | positive regulation of biological process | tr|A0A1D5PCZ1|A0A1D5PCZ1_CHICK | 1.542638 | up |
| biological_process | positive regulation of biological process | tr|A0A1D5PFS2|A0A1D5PFS2_CHICK | 1.463999 | up |
| biological_process | positive regulation of biological process | tr|A0A1D5PRI4|A0A1D5PRI4_CHICK | 1.28486 | up |
| biological_process | positive regulation of biological process | tr|A0A1D5PTE8|A0A1D5PTE8_CHICK | 1.960486 | up |
| biological_process | positive regulation of biological process | tr|A0A1D5PWD7|A0A1D5PWD7_CHICK | 1.676687 | up |
| biological_process | positive regulation of biological process | tr|A0A3Q2TY77|A0A3Q2TY77_CHICK | 1.82929 | up |
| biological_process | positive regulation of biological process | tr|A0A3Q2TZM7|A0A3Q2TZM7_CHICK | 1.419267 | up |
| biological_process | positive regulation of biological process | tr|A0A3Q2UCN1|A0A3Q2UCN1_CHICK | 1.92272 | up |
| biological_process | positive regulation of biological process | tr|A0A3Q2UPF3|A0A3Q2UPF3_CHICK | 1.295517 | up |
| biological_process | positive regulation of biological process | tr|A0A3Q3ALC8|A0A3Q3ALC8_CHICK | 2.188996 | up |
| biological_process | positive regulation of biological process | tr|D2D3P4|D2D3P4_CHICK | 2.146381 | up |
| biological_process | positive regulation of biological process | tr|E1BQI0|E1BQI0_CHICK | 1.067708 | up |
| biological_process | positive regulation of biological process | tr|E1BQW2|E1BQW2_CHICK | 1.823966 | up |
| biological_process | positive regulation of biological process | tr|E1BSH7|E1BSH7_CHICK | 1.527514 | up |
| biological_process | positive regulation of biological process | tr|E1BTV1|E1BTV1_CHICK | 2.001762 | up |
| biological_process | positive regulation of biological process | tr|E1BVQ3|E1BVQ3_CHICK | 1.650579 | up |
| biological_process | positive regulation of biological process | tr|E1BW27|E1BW27_CHICK | 1.938051 | up |
| biological_process | positive regulation of biological process | tr|E1BX21|E1BX21_CHICK | 1.42634 | up |
| biological_process | positive regulation of biological process | tr|E1BYQ3|E1BYQ3_CHICK | 1.202088 | up |
| biological_process | positive regulation of biological process | tr|E1C396|E1C396_CHICK | 1.08258 | up |
| biological_process | positive regulation of biological process | tr|E1C7H6|E1C7H6_CHICK | -1.05537 | down |
| biological_process | positive regulation of biological process | tr|E1C7P7|E1C7P7_CHICK | 2.341246 | up |
| biological_process | positive regulation of biological process | tr|E7EC82|E7EC82_CHICK | 1.586027 | up |
| biological_process | positive regulation of biological process | tr|F1N9S3|F1N9S3_CHICK | 1.256683 | up |
| biological_process | positive regulation of biological process | tr|F1N9U0|F1N9U0_CHICK | 1.038918 | up |
| biological_process | positive regulation of biological process | tr|F1N9Y3|F1N9Y3_CHICK | 1.33841 | up |
| biological_process | positive regulation of biological process | tr|F1NDH2|F1NDH2_CHICK | -1.95135 | down |
| biological_process | positive regulation of biological process | tr|F1NIY3|F1NIY3_CHICK | 1.568578 | up |
| biological_process | positive regulation of biological process | tr|F1NJU5|F1NJU5_CHICK | -1.49981 | down |
| biological_process | positive regulation of biological process | tr|F1NMJ9|F1NMJ9_CHICK | -2.4895 | down |
| biological_process | positive regulation of biological process | tr|F1NRZ4|F1NRZ4_CHICK | 1.14911 | up |
| biological_process | positive regulation of biological process | tr|F1NSF2|F1NSF2_CHICK | 1.708711 | up |
| biological_process | positive regulation of biological process | tr|F1NT58|F1NT58_CHICK | 1.701877 | up |
| biological_process | positive regulation of biological process | tr|F1NT80|F1NT80_CHICK | 1.404176 | up |
| biological_process | positive regulation of biological process | tr|F1NTQ2|F1NTQ2_CHICK | 1.236275 | up |
| biological_process | positive regulation of biological process | tr|F1NXV6|F1NXV6_CHICK | -4.87705 | down |
| biological_process | positive regulation of biological process | tr|I1SV11|I1SV11_CHICK | 1.313667 | up |
| biological_process | positive regulation of biological process | tr|O93466|O93466_CHICK | 1.669881 | up |
| biological_process | positive regulation of biological process | tr|Q2PUH1|Q2PUH1_CHICK | 1.468226 | up |
| biological_process | positive regulation of biological process | tr|Q5F3X1|Q5F3X1_CHICK | 1.19047 | up |
| biological_process | positive regulation of biological process | tr|Q5ZIL3|Q5ZIL3_CHICK | 1.863958 | up |
| biological_process | positive regulation of biological process | tr|Q5ZIZ3|Q5ZIZ3_CHICK | 1.248317 | up |
| biological_process | positive regulation of biological process | tr|Q5ZJ19|Q5ZJ19_CHICK | 2.470646 | up |
| biological_process | positive regulation of biological process | tr|Q5ZJ96|Q5ZJ96_CHICK | 1.306532 | up |
| biological_process | positive regulation of biological process | tr|Q5ZJQ4|Q5ZJQ4_CHICK | 1.678561 | up |
| biological_process | positive regulation of biological process | tr|Q5ZKI3|Q5ZKI3_CHICK | 1.326993 | up |
| biological_process | positive regulation of biological process | tr|Q5ZL65|Q5ZL65_CHICK | 1.695268 | up |
| biological_process | positive regulation of biological process | tr|Q5ZMP2|Q5ZMP2_CHICK | 1.165624 | up |
| biological_process | positive regulation of biological process | tr|Q7ZTS9|Q7ZTS9_CHICK | 1.923559 | up |
| biological_process | positive regulation of biological process | tr|Q9PUJ4|Q9PUJ4_CHICK | 1.664957 | up |
| biological_process | positive regulation of biological process | tr|Q9PVL6|Q9PVL6_CHICK | 1.71949 | up |
| biological_process | positive regulation of biological process | tr|R4GI86|R4GI86_CHICK | 2.078653 | up |
| biological_process | presynaptic process involved in chemical synaptic transmission | sp|Q5ZIK2|PDZ11_CHICK | 1.259402 | up |
| biological_process | presynaptic process involved in chemical synaptic transmission | tr|A0A3Q2TY77|A0A3Q2TY77_CHICK | 1.82929 | up |
| biological_process | regulation of biological process | sp|O42395|CNBP_CHICK | 1.365125 | up |
| biological_process | regulation of biological process | sp|O93436|STAM2_CHICK | 2.376336 | up |
| biological_process | regulation of biological process | sp|O93602|ATF2_CHICK | 2.071233 | up |
| biological_process | regulation of biological process | sp|P00523|SRC_CHICK | 1.599352 | up |
| biological_process | regulation of biological process | sp|P25155|FA10_CHICK | -3.77432 | down |
| biological_process | regulation of biological process | sp|P28318|M126_CHICK | 3.846633 | up |
| biological_process | regulation of biological process | sp|P52162|MAX_CHICK | 1.334512 | up |
| biological_process | regulation of biological process | sp|P81475|CFBL_CHICK | -1.75388 | down |
| biological_process | regulation of biological process | sp|Q05423|FABP7_CHICK | 1.9874 | up |
| biological_process | regulation of biological process | sp|Q5F3K4|WDR48_CHICK | 1.71564 | up |
| biological_process | regulation of biological process | sp|Q5ZI08|SPT5H_CHICK | 1.235361 | up |
| biological_process | regulation of biological process | sp|Q5ZIB9|ANM7_CHICK | 1.168118 | up |
| biological_process | regulation of biological process | sp|Q5ZII9|TFP11_CHICK | 1.172107 | up |
| biological_process | regulation of biological process | sp|Q5ZIP4|XRN2_CHICK | 1.286156 | up |
| biological_process | regulation of biological process | sp|Q5ZJ00|EM55_CHICK | 1.664901 | up |
| biological_process | regulation of biological process | sp|Q5ZJW6|KBRS2_CHICK | 1.256362 | up |
| biological_process | regulation of biological process | sp|Q5ZK05|TOLIP_CHICK | 1.088317 | up |
| biological_process | regulation of biological process | sp|Q5ZK92|SPAST_CHICK | 1.665787 | up |
| biological_process | regulation of biological process | sp|Q5ZL36|ZFY27_CHICK | 1.948763 | up |
| biological_process | regulation of biological process | sp|Q5ZM60|CCPG1_CHICK | 1.624972 | up |
| biological_process | regulation of biological process | sp|Q5ZM83|MIRO2_CHICK | 1.659999 | up |
| biological_process | regulation of biological process | sp|Q5ZMG0|DOPD_CHICK | 2.121364 | up |
| biological_process | regulation of biological process | sp|Q5ZML0|BABA2_CHICK | 1.144731 | up |
| biological_process | regulation of biological process | sp|Q8AXL1|SAT1_CHICK | 1.12819 | up |
| biological_process | regulation of biological process | sp|Q90733|COT2_CHICK | 1.595685 | up |
| biological_process | regulation of biological process | sp|Q9IAM7|MRE11_CHICK | 1.337957 | up |
| biological_process | regulation of biological process | tr|A0A1D5NUD3|A0A1D5NUD3_CHICK | 1.402549 | up |
| biological_process | regulation of biological process | tr|A0A1D5NUD9|A0A1D5NUD9_CHICK | 1.157212 | up |
| biological_process | regulation of biological process | tr|A0A1D5NUU0|A0A1D5NUU0_CHICK | 1.000304 | up |
| biological_process | regulation of biological process | tr|A0A1D5NUV0|A0A1D5NUV0_CHICK | 2.157037 | up |
| biological_process | regulation of biological process | tr|A0A1D5NWV6|A0A1D5NWV6_CHICK | 1.553336 | up |
| biological_process | regulation of biological process | tr|A0A1D5NZB4|A0A1D5NZB4_CHICK | 1.671702 | up |
| biological_process | regulation of biological process | tr|A0A1D5NZS7|A0A1D5NZS7_CHICK | 1.185694 | up |
| biological_process | regulation of biological process | tr|A0A1D5P047|A0A1D5P047_CHICK | 1.659689 | up |
| biological_process | regulation of biological process | tr|A0A1D5P6C3|A0A1D5P6C3_CHICK | 2.100747 | up |
| biological_process | regulation of biological process | tr|A0A1D5P822|A0A1D5P822_CHICK | 1.478805 | up |
| biological_process | regulation of biological process | tr|A0A1D5P8H7|A0A1D5P8H7_CHICK | 1.530513 | up |
| biological_process | regulation of biological process | tr|A0A1D5P997|A0A1D5P997_CHICK | 1.563709 | up |
| biological_process | regulation of biological process | tr|A0A1D5P9G2|A0A1D5P9G2_CHICK | 1.768438 | up |
| biological_process | regulation of biological process | tr|A0A1D5PA08|A0A1D5PA08_CHICK | 1.946165 | up |
| biological_process | regulation of biological process | tr|A0A1D5PCZ1|A0A1D5PCZ1_CHICK | 1.542638 | up |
| biological_process | regulation of biological process | tr|A0A1D5PFS2|A0A1D5PFS2_CHICK | 1.463999 | up |
| biological_process | regulation of biological process | tr|A0A1D5PIB1|A0A1D5PIB1_CHICK | 2.10824 | up |
| biological_process | regulation of biological process | tr|A0A1D5PIT4|A0A1D5PIT4_CHICK | -1.97988 | down |
| biological_process | regulation of biological process | tr|A0A1D5PK72|A0A1D5PK72_CHICK | 2.032922 | up |
| biological_process | regulation of biological process | tr|A0A1D5PQ66|A0A1D5PQ66_CHICK | 1.664552 | up |
| biological_process | regulation of biological process | tr|A0A1D5PQD0|A0A1D5PQD0_CHICK | 1.314273 | up |
| biological_process | regulation of biological process | tr|A0A1D5PQG6|A0A1D5PQG6_CHICK | 2.091788 | up |
| biological_process | regulation of biological process | tr|A0A1D5PR34|A0A1D5PR34_CHICK | 1.370909 | up |
| biological_process | regulation of biological process | tr|A0A1D5PRI4|A0A1D5PRI4_CHICK | 1.28486 | up |
| biological_process | regulation of biological process | tr|A0A1D5PRI6|A0A1D5PRI6_CHICK | 1.580404 | up |
| biological_process | regulation of biological process | tr|A0A1D5PTE8|A0A1D5PTE8_CHICK | 1.960486 | up |
| biological_process | regulation of biological process | tr|A0A1D5PUJ5|A0A1D5PUJ5_CHICK | 1.266991 | up |
| biological_process | regulation of biological process | tr|A0A1D5PWD7|A0A1D5PWD7_CHICK | 1.676687 | up |
| biological_process | regulation of biological process | tr|A0A1D5PWT4|A0A1D5PWT4_CHICK | 1.101328 | up |
| biological_process | regulation of biological process | tr|A0A1D5PXN4|A0A1D5PXN4_CHICK | -1.85723 | down |
| biological_process | regulation of biological process | tr|A0A1D5PYB7|A0A1D5PYB7_CHICK | 1.489522 | up |
| biological_process | regulation of biological process | tr|A0A3Q2TUF8|A0A3Q2TUF8_CHICK | 1.02023 | up |
| biological_process | regulation of biological process | tr|A0A3Q2TVU0|A0A3Q2TVU0_CHICK | 1.280793 | up |
| biological_process | regulation of biological process | tr|A0A3Q2TY77|A0A3Q2TY77_CHICK | 1.82929 | up |
| biological_process | regulation of biological process | tr|A0A3Q2TYP7|A0A3Q2TYP7_CHICK | 2.159057 | up |
| biological_process | regulation of biological process | tr|A0A3Q2TZM7|A0A3Q2TZM7_CHICK | 1.419267 | up |
| biological_process | regulation of biological process | tr|A0A3Q2U504|A0A3Q2U504_CHICK | -2.35777 | down |
| biological_process | regulation of biological process | tr|A0A3Q2U8I0|A0A3Q2U8I0_CHICK | 1.050068 | up |
| biological_process | regulation of biological process | tr|A0A3Q2UBJ7|A0A3Q2UBJ7_CHICK | 1.945958 | up |
| biological_process | regulation of biological process | tr|A0A3Q2UCN1|A0A3Q2UCN1_CHICK | 1.92272 | up |
| biological_process | regulation of biological process | tr|A0A3Q2UD87|A0A3Q2UD87_CHICK | 1.205498 | up |
| biological_process | regulation of biological process | tr|A0A3Q2UPF3|A0A3Q2UPF3_CHICK | 1.295517 | up |
| biological_process | regulation of biological process | tr|A0A3Q3AB84|A0A3Q3AB84_CHICK | 1.170717 | up |
| biological_process | regulation of biological process | tr|A0A3Q3ALC8|A0A3Q3ALC8_CHICK | 2.188996 | up |
| biological_process | regulation of biological process | tr|A8VIF4|A8VIF4_CHICK | 1.024165 | up |
| biological_process | regulation of biological process | tr|D2D3P4|D2D3P4_CHICK | 2.146381 | up |
| biological_process | regulation of biological process | tr|E1BQI0|E1BQI0_CHICK | 1.067708 | up |
| biological_process | regulation of biological process | tr|E1BQW2|E1BQW2_CHICK | 1.823966 | up |
| biological_process | regulation of biological process | tr|E1BRQ9|E1BRQ9_CHICK | 1.034807 | up |
| biological_process | regulation of biological process | tr|E1BS94|E1BS94_CHICK | 1.70074 | up |
| biological_process | regulation of biological process | tr|E1BSH7|E1BSH7_CHICK | 1.527514 | up |
| biological_process | regulation of biological process | tr|E1BTE2|E1BTE2_CHICK | -2.67874 | down |
| biological_process | regulation of biological process | tr|E1BTV1|E1BTV1_CHICK | 2.001762 | up |
| biological_process | regulation of biological process | tr|E1BU89|E1BU89_CHICK | 1.303565 | up |
| biological_process | regulation of biological process | tr|E1BUJ1|E1BUJ1_CHICK | 2.709557 | up |
| biological_process | regulation of biological process | tr|E1BUX5|E1BUX5_CHICK | 1.099122 | up |
| biological_process | regulation of biological process | tr|E1BVQ3|E1BVQ3_CHICK | 1.650579 | up |
| biological_process | regulation of biological process | tr|E1BW27|E1BW27_CHICK | 1.938051 | up |
| biological_process | regulation of biological process | tr|E1BWB7|E1BWB7_CHICK | 1.446045 | up |
| biological_process | regulation of biological process | tr|E1BX21|E1BX21_CHICK | 1.42634 | up |
| biological_process | regulation of biological process | tr|E1BXS1|E1BXS1_CHICK | 1.514054 | up |
| biological_process | regulation of biological process | tr|E1BY40|E1BY40_CHICK | 1.129106 | up |
| biological_process | regulation of biological process | tr|E1BYQ3|E1BYQ3_CHICK | 1.202088 | up |
| biological_process | regulation of biological process | tr|E1BZ79|E1BZ79_CHICK | 2.388844 | up |
| biological_process | regulation of biological process | tr|E1C312|E1C312_CHICK | 1.811403 | up |
| biological_process | regulation of biological process | tr|E1C396|E1C396_CHICK | 1.08258 | up |
| biological_process | regulation of biological process | tr|E1C3U6|E1C3U6_CHICK | 1.144268 | up |
| biological_process | regulation of biological process | tr|E1C592|E1C592_CHICK | 1.565005 | up |
| biological_process | regulation of biological process | tr|E1C688|E1C688_CHICK | 1.775755 | up |
| biological_process | regulation of biological process | tr|E1C6S7|E1C6S7_CHICK | 1.1895 | up |
| biological_process | regulation of biological process | tr|E1C7H6|E1C7H6_CHICK | -1.05537 | down |
| biological_process | regulation of biological process | tr|E1C7P7|E1C7P7_CHICK | 2.341246 | up |
| biological_process | regulation of biological process | tr|E1C8U1|E1C8U1_CHICK | 1.411305 | up |
| biological_process | regulation of biological process | tr|E7EC82|E7EC82_CHICK | 1.586027 | up |
| biological_process | regulation of biological process | tr|F1N8V5|F1N8V5_CHICK | 2.344853 | up |
| biological_process | regulation of biological process | tr|F1N9S3|F1N9S3_CHICK | 1.256683 | up |
| biological_process | regulation of biological process | tr|F1N9U0|F1N9U0_CHICK | 1.038918 | up |
| biological_process | regulation of biological process | tr|F1N9Y3|F1N9Y3_CHICK | 1.33841 | up |
| biological_process | regulation of biological process | tr|F1NBX1|F1NBX1_CHICK | 1.439614 | up |
| biological_process | regulation of biological process | tr|F1NDH2|F1NDH2_CHICK | -1.95135 | down |
| biological_process | regulation of biological process | tr|F1NIQ3|F1NIQ3_CHICK | 1.483149 | up |
| biological_process | regulation of biological process | tr|F1NIY3|F1NIY3_CHICK | 1.568578 | up |
| biological_process | regulation of biological process | tr|F1NJU5|F1NJU5_CHICK | -1.49981 | down |
| biological_process | regulation of biological process | tr|F1NMJ9|F1NMJ9_CHICK | -2.4895 | down |
| biological_process | regulation of biological process | tr|F1NPS5|F1NPS5_CHICK | 3.319197 | up |
| biological_process | regulation of biological process | tr|F1NQ61|F1NQ61_CHICK | 1.080306 | up |
| biological_process | regulation of biological process | tr|F1NR86|F1NR86_CHICK | 1.817533 | up |
| biological_process | regulation of biological process | tr|F1NRZ4|F1NRZ4_CHICK | 1.14911 | up |
| biological_process | regulation of biological process | tr|F1NSF2|F1NSF2_CHICK | 1.708711 | up |
| biological_process | regulation of biological process | tr|F1NSI7|F1NSI7_CHICK | 1.77407 | up |
| biological_process | regulation of biological process | tr|F1NSQ4|F1NSQ4_CHICK | 1.21442 | up |
| biological_process | regulation of biological process | tr|F1NT58|F1NT58_CHICK | 1.701877 | up |
| biological_process | regulation of biological process | tr|F1NT80|F1NT80_CHICK | 1.404176 | up |
| biological_process | regulation of biological process | tr|F1NTQ2|F1NTQ2_CHICK | 1.236275 | up |
| biological_process | regulation of biological process | tr|F1NXV6|F1NXV6_CHICK | -4.87705 | down |
| biological_process | regulation of biological process | tr|F1P099|F1P099_CHICK | 1.399388 | up |
| biological_process | regulation of biological process | tr|F1P151|F1P151_CHICK | 2.603153 | up |
| biological_process | regulation of biological process | tr|F1P394|F1P394_CHICK | 1.286828 | up |
| biological_process | regulation of biological process | tr|F1P5A3|F1P5A3_CHICK | 2.127136 | up |
| biological_process | regulation of biological process | tr|F1P5X6|F1P5X6_CHICK | 1.295882 | up |
| biological_process | regulation of biological process | tr|F6YX81|F6YX81_CHICK | 1.194552 | up |
| biological_process | regulation of biological process | tr|H9L023|H9L023_CHICK | 1.212184 | up |
| biological_process | regulation of biological process | tr|I1SV11|I1SV11_CHICK | 1.313667 | up |
| biological_process | regulation of biological process | tr|O93466|O93466_CHICK | 1.669881 | up |
| biological_process | regulation of biological process | tr|Q2PUH1|Q2PUH1_CHICK | 1.468226 | up |
| biological_process | regulation of biological process | tr|Q5DWF6|Q5DWF6_CHICK | 1.289716 | up |
| biological_process | regulation of biological process | tr|Q5F3R8|Q5F3R8_CHICK | 2.332553 | up |
| biological_process | regulation of biological process | tr|Q5F3X1|Q5F3X1_CHICK | 1.19047 | up |
| biological_process | regulation of biological process | tr|Q5ZIL3|Q5ZIL3_CHICK | 1.863958 | up |
| biological_process | regulation of biological process | tr|Q5ZIZ3|Q5ZIZ3_CHICK | 1.248317 | up |
| biological_process | regulation of biological process | tr|Q5ZJ19|Q5ZJ19_CHICK | 2.470646 | up |
| biological_process | regulation of biological process | tr|Q5ZJ96|Q5ZJ96_CHICK | 1.306532 | up |
| biological_process | regulation of biological process | tr|Q5ZJA8|Q5ZJA8_CHICK | 1.093474 | up |
| biological_process | regulation of biological process | tr|Q5ZJQ4|Q5ZJQ4_CHICK | 1.678561 | up |
| biological_process | regulation of biological process | tr|Q5ZKI3|Q5ZKI3_CHICK | 1.326993 | up |
| biological_process | regulation of biological process | tr|Q5ZL65|Q5ZL65_CHICK | 1.695268 | up |
| biological_process | regulation of biological process | tr|Q5ZLI0|Q5ZLI0_CHICK | 1.097879 | up |
| biological_process | regulation of biological process | tr|Q5ZMA7|Q5ZMA7_CHICK | 1.172026 | up |
| biological_process | regulation of biological process | tr|Q5ZMG8|Q5ZMG8_CHICK | 1.771296 | up |
| biological_process | regulation of biological process | tr|Q5ZMJ6|Q5ZMJ6_CHICK | 1.845871 | up |
| biological_process | regulation of biological process | tr|Q5ZMP2|Q5ZMP2_CHICK | 1.165624 | up |
| biological_process | regulation of biological process | tr|Q7ZTS9|Q7ZTS9_CHICK | 1.923559 | up |
| biological_process | regulation of biological process | tr|Q9PUJ4|Q9PUJ4_CHICK | 1.664957 | up |
| biological_process | regulation of biological process | tr|Q9PVL6|Q9PVL6_CHICK | 1.71949 | up |
| biological_process | regulation of biological process | tr|R4GI86|R4GI86_CHICK | 2.078653 | up |
| biological_process | regulation of biological process | tr|R4GMH5|R4GMH5_CHICK | -2.32193 | down |
| biological_process | reproduction | sp|Q5F3K4|WDR48_CHICK | 1.71564 | up |
| biological_process | reproduction | sp|Q5ZIB9|ANM7_CHICK | 1.168118 | up |
| biological_process | reproduction | sp|Q5ZIP4|XRN2_CHICK | 1.286156 | up |
| biological_process | reproduction | sp|Q90733|COT2_CHICK | 1.595685 | up |
| biological_process | reproduction | sp|Q9IAM7|MRE11_CHICK | 1.337957 | up |
| biological_process | reproduction | tr|A0A1D5NU82|A0A1D5NU82_CHICK | 1.12312 | up |
| biological_process | reproduction | tr|A0A1D5NXG4|A0A1D5NXG4_CHICK | 1.245324 | up |
| biological_process | reproduction | tr|A0A1D5PA08|A0A1D5PA08_CHICK | 1.946165 | up |
| biological_process | reproduction | tr|A0A1D5PIT4|A0A1D5PIT4_CHICK | -1.97988 | down |
| biological_process | reproduction | tr|A0A3Q2UD87|A0A3Q2UD87_CHICK | 1.205498 | up |
| biological_process | reproduction | tr|E1BQU2|E1BQU2_CHICK | 1.629018 | up |
| biological_process | reproduction | tr|E1BTE2|E1BTE2_CHICK | -2.67874 | down |
| biological_process | reproduction | tr|E1C688|E1C688_CHICK | 1.775755 | up |
| biological_process | reproduction | tr|F1NTQ2|F1NTQ2_CHICK | 1.236275 | up |
| biological_process | reproduction | tr|I1SV11|I1SV11_CHICK | 1.313667 | up |
| biological_process | reproduction | tr|Q5ZKY5|Q5ZKY5_CHICK | 1.620297 | up |
| biological_process | reproductive process | sp|Q5F3K4|WDR48_CHICK | 1.71564 | up |
| biological_process | reproductive process | sp|Q5ZIB9|ANM7_CHICK | 1.168118 | up |
| biological_process | reproductive process | sp|Q5ZIP4|XRN2_CHICK | 1.286156 | up |
| biological_process | reproductive process | sp|Q90733|COT2_CHICK | 1.595685 | up |
| biological_process | reproductive process | sp|Q9IAM7|MRE11_CHICK | 1.337957 | up |
| biological_process | reproductive process | tr|A0A1D5NU82|A0A1D5NU82_CHICK | 1.12312 | up |
| biological_process | reproductive process | tr|A0A1D5NXG4|A0A1D5NXG4_CHICK | 1.245324 | up |
| biological_process | reproductive process | tr|A0A1D5PA08|A0A1D5PA08_CHICK | 1.946165 | up |
| biological_process | reproductive process | tr|A0A1D5PIT4|A0A1D5PIT4_CHICK | -1.97988 | down |
| biological_process | reproductive process | tr|A0A3Q2UD87|A0A3Q2UD87_CHICK | 1.205498 | up |
| biological_process | reproductive process | tr|E1BQU2|E1BQU2_CHICK | 1.629018 | up |
| biological_process | reproductive process | tr|E1BTE2|E1BTE2_CHICK | -2.67874 | down |
| biological_process | reproductive process | tr|E1C688|E1C688_CHICK | 1.775755 | up |
| biological_process | reproductive process | tr|F1NTQ2|F1NTQ2_CHICK | 1.236275 | up |
| biological_process | reproductive process | tr|I1SV11|I1SV11_CHICK | 1.313667 | up |
| biological_process | reproductive process | tr|Q5ZKY5|Q5ZKY5_CHICK | 1.620297 | up |
| biological_process | response to stimulus | sp|O93436|STAM2_CHICK | 2.376336 | up |
| biological_process | response to stimulus | sp|O93602|ATF2_CHICK | 2.071233 | up |
| biological_process | response to stimulus | sp|P00523|SRC_CHICK | 1.599352 | up |
| biological_process | response to stimulus | sp|P02197|MYG_CHICK | 2.885059 | up |
| biological_process | response to stimulus | sp|P04210|LV1_CHICK | -2.82825 | down |
| biological_process | response to stimulus | sp|P25155|FA10_CHICK | -3.77432 | down |
| biological_process | response to stimulus | sp|P28318|M126_CHICK | 3.846633 | up |
| biological_process | response to stimulus | sp|P42292|CD166_CHICK | 1.297212 | up |
| biological_process | response to stimulus | sp|P81475|CFBL_CHICK | -1.75388 | down |
| biological_process | response to stimulus | sp|Q05423|FABP7_CHICK | 1.9874 | up |
| biological_process | response to stimulus | sp|Q5F3K4|WDR48_CHICK | 1.71564 | up |
| biological_process | response to stimulus | sp|Q5F3Z3|UB2V2_CHICK | 2.366484 | up |
| biological_process | response to stimulus | sp|Q5ZHN3|WIPI2_CHICK | 1.61465 | up |
| biological_process | response to stimulus | sp|Q5ZJ00|EM55_CHICK | 1.664901 | up |
| biological_process | response to stimulus | sp|Q5ZJW6|KBRS2_CHICK | 1.256362 | up |
| biological_process | response to stimulus | sp|Q5ZK05|TOLIP_CHICK | 1.088317 | up |
| biological_process | response to stimulus | sp|Q5ZL36|ZFY27_CHICK | 1.948763 | up |
| biological_process | response to stimulus | sp|Q5ZM83|MIRO2_CHICK | 1.659999 | up |
| biological_process | response to stimulus | sp|Q5ZMG0|DOPD_CHICK | 2.121364 | up |
| biological_process | response to stimulus | sp|Q5ZML0|BABA2_CHICK | 1.144731 | up |
| biological_process | response to stimulus | sp|Q804X6|FA9_CHICK | -5.71386 | down |
| biological_process | response to stimulus | sp|Q9IAM7|MRE11_CHICK | 1.337957 | up |
| biological_process | response to stimulus | tr|A0A1D5NT70|A0A1D5NT70_CHICK | 1.819215 | up |
| biological_process | response to stimulus | tr|A0A1D5NUD3|A0A1D5NUD3_CHICK | 1.402549 | up |
| biological_process | response to stimulus | tr|A0A1D5NUD9|A0A1D5NUD9_CHICK | 1.157212 | up |
| biological_process | response to stimulus | tr|A0A1D5NUU0|A0A1D5NUU0_CHICK | 1.000304 | up |
| biological_process | response to stimulus | tr|A0A1D5NUV0|A0A1D5NUV0_CHICK | 2.157037 | up |
| biological_process | response to stimulus | tr|A0A1D5NWV6|A0A1D5NWV6_CHICK | 1.553336 | up |
| biological_process | response to stimulus | tr|A0A1D5NZB4|A0A1D5NZB4_CHICK | 1.671702 | up |
| biological_process | response to stimulus | tr|A0A1D5NZS7|A0A1D5NZS7_CHICK | 1.185694 | up |
| biological_process | response to stimulus | tr|A0A1D5P453|A0A1D5P453_CHICK | 1.553695 | up |
| biological_process | response to stimulus | tr|A0A1D5P9G2|A0A1D5P9G2_CHICK | 1.768438 | up |
| biological_process | response to stimulus | tr|A0A1D5PA08|A0A1D5PA08_CHICK | 1.946165 | up |
| biological_process | response to stimulus | tr|A0A1D5PAM1|A0A1D5PAM1_CHICK | 2.577291 | up |
| biological_process | response to stimulus | tr|A0A1D5PCZ1|A0A1D5PCZ1_CHICK | 1.542638 | up |
| biological_process | response to stimulus | tr|A0A1D5PFS2|A0A1D5PFS2_CHICK | 1.463999 | up |
| biological_process | response to stimulus | tr|A0A1D5PIB1|A0A1D5PIB1_CHICK | 2.10824 | up |
| biological_process | response to stimulus | tr|A0A1D5PK72|A0A1D5PK72_CHICK | 2.032922 | up |
| biological_process | response to stimulus | tr|A0A1D5PQD0|A0A1D5PQD0_CHICK | 1.314273 | up |
| biological_process | response to stimulus | tr|A0A1D5PRI4|A0A1D5PRI4_CHICK | 1.28486 | up |
| biological_process | response to stimulus | tr|A0A1D5PRI6|A0A1D5PRI6_CHICK | 1.580404 | up |
| biological_process | response to stimulus | tr|A0A1D5PWD7|A0A1D5PWD7_CHICK | 1.676687 | up |
| biological_process | response to stimulus | tr|A0A1D5PXN4|A0A1D5PXN4_CHICK | -1.85723 | down |
| biological_process | response to stimulus | tr|A0A3Q2TUF8|A0A3Q2TUF8_CHICK | 1.02023 | up |
| biological_process | response to stimulus | tr|A0A3Q2TVV0|A0A3Q2TVV0_CHICK | 1.145618 | up |
| biological_process | response to stimulus | tr|A0A3Q2U504|A0A3Q2U504_CHICK | -2.35777 | down |
| biological_process | response to stimulus | tr|A0A3Q2UCN1|A0A3Q2UCN1_CHICK | 1.92272 | up |
| biological_process | response to stimulus | tr|A0A3Q3AIE0|A0A3Q3AIE0_CHICK | 1.346321 | up |
| biological_process | response to stimulus | tr|A0A3Q3ALC8|A0A3Q3ALC8_CHICK | 2.188996 | up |
| biological_process | response to stimulus | tr|A8VIF4|A8VIF4_CHICK | 1.024165 | up |
| biological_process | response to stimulus | tr|D2D3P4|D2D3P4_CHICK | 2.146381 | up |
| biological_process | response to stimulus | tr|E1BQC2|E1BQC2_CHICK | -2.52084 | down |
| biological_process | response to stimulus | tr|E1BS94|E1BS94_CHICK | 1.70074 | up |
| biological_process | response to stimulus | tr|E1BSH7|E1BSH7_CHICK | 1.527514 | up |
| biological_process | response to stimulus | tr|E1BTI7|E1BTI7_CHICK | 1.813027 | up |
| biological_process | response to stimulus | tr|E1BU89|E1BU89_CHICK | 1.303565 | up |
| biological_process | response to stimulus | tr|E1BUJ1|E1BUJ1_CHICK | 2.709557 | up |
| biological_process | response to stimulus | tr|E1BVQ3|E1BVQ3_CHICK | 1.650579 | up |
| biological_process | response to stimulus | tr|E1BW27|E1BW27_CHICK | 1.938051 | up |
| biological_process | response to stimulus | tr|E1BX21|E1BX21_CHICK | 1.42634 | up |
| biological_process | response to stimulus | tr|E1BXC2|E1BXC2_CHICK | 1.767478 | up |
| biological_process | response to stimulus | tr|E1BYQ3|E1BYQ3_CHICK | 1.202088 | up |
| biological_process | response to stimulus | tr|E1BZ79|E1BZ79_CHICK | 2.388844 | up |
| biological_process | response to stimulus | tr|E1C396|E1C396_CHICK | 1.08258 | up |
| biological_process | response to stimulus | tr|E1C3U6|E1C3U6_CHICK | 1.144268 | up |
| biological_process | response to stimulus | tr|E1C592|E1C592_CHICK | 1.565005 | up |
| biological_process | response to stimulus | tr|E1C688|E1C688_CHICK | 1.775755 | up |
| biological_process | response to stimulus | tr|E1C6U2|E1C6U2_CHICK | -1.17102 | down |
| biological_process | response to stimulus | tr|E1C7A7|E1C7A7_CHICK | -1.02532 | down |
| biological_process | response to stimulus | tr|E1C7C1|E1C7C1_CHICK | -1.84307 | down |
| biological_process | response to stimulus | tr|E1C7P7|E1C7P7_CHICK | 2.341246 | up |
| biological_process | response to stimulus | tr|E7EC82|E7EC82_CHICK | 1.586027 | up |
| biological_process | response to stimulus | tr|F1N9Y3|F1N9Y3_CHICK | 1.33841 | up |
| biological_process | response to stimulus | tr|F1NBX1|F1NBX1_CHICK | 1.439614 | up |
| biological_process | response to stimulus | tr|F1NDH2|F1NDH2_CHICK | -1.95135 | down |
| biological_process | response to stimulus | tr|F1NIQ3|F1NIQ3_CHICK | 1.483149 | up |
| biological_process | response to stimulus | tr|F1NIY3|F1NIY3_CHICK | 1.568578 | up |
| biological_process | response to stimulus | tr|F1NJU5|F1NJU5_CHICK | -1.49981 | down |
| biological_process | response to stimulus | tr|F1NLM0|F1NLM0_CHICK | 1.884615 | up |
| biological_process | response to stimulus | tr|F1NMJ9|F1NMJ9_CHICK | -2.4895 | down |
| biological_process | response to stimulus | tr|F1NPS5|F1NPS5_CHICK | 3.319197 | up |
| biological_process | response to stimulus | tr|F1NR86|F1NR86_CHICK | 1.817533 | up |
| biological_process | response to stimulus | tr|F1NRZ4|F1NRZ4_CHICK | 1.14911 | up |
| biological_process | response to stimulus | tr|F1NSQ4|F1NSQ4_CHICK | 1.21442 | up |
| biological_process | response to stimulus | tr|F1NV66|F1NV66_CHICK | 1.865555 | up |
| biological_process | response to stimulus | tr|F1NXV6|F1NXV6_CHICK | -4.87705 | down |
| biological_process | response to stimulus | tr|F1P151|F1P151_CHICK | 2.603153 | up |
| biological_process | response to stimulus | tr|F1P394|F1P394_CHICK | 1.286828 | up |
| biological_process | response to stimulus | tr|F6YX81|F6YX81_CHICK | 1.194552 | up |
| biological_process | response to stimulus | tr|H9L023|H9L023_CHICK | 1.212184 | up |
| biological_process | response to stimulus | tr|O93466|O93466_CHICK | 1.669881 | up |
| biological_process | response to stimulus | tr|Q2PUH1|Q2PUH1_CHICK | 1.468226 | up |
| biological_process | response to stimulus | tr|Q4ADJ6|Q4ADJ6_CHICK | -4.04825 | down |
| biological_process | response to stimulus | tr|Q5DWF6|Q5DWF6_CHICK | 1.289716 | up |
| biological_process | response to stimulus | tr|Q5F351|Q5F351_CHICK | 1.342446 | up |
| biological_process | response to stimulus | tr|Q5F3R8|Q5F3R8_CHICK | 2.332553 | up |
| biological_process | response to stimulus | tr|Q5F3X1|Q5F3X1_CHICK | 1.19047 | up |
| biological_process | response to stimulus | tr|Q5ZIL3|Q5ZIL3_CHICK | 1.863958 | up |
| biological_process | response to stimulus | tr|Q5ZJ19|Q5ZJ19_CHICK | 2.470646 | up |
| biological_process | response to stimulus | tr|Q5ZJA8|Q5ZJA8_CHICK | 1.093474 | up |
| biological_process | response to stimulus | tr|Q5ZJQ4|Q5ZJQ4_CHICK | 1.678561 | up |
| biological_process | response to stimulus | tr|Q5ZKI3|Q5ZKI3_CHICK | 1.326993 | up |
| biological_process | response to stimulus | tr|Q5ZL65|Q5ZL65_CHICK | 1.695268 | up |
| biological_process | response to stimulus | tr|Q5ZMA7|Q5ZMA7_CHICK | 1.172026 | up |
| biological_process | response to stimulus | tr|Q5ZMG8|Q5ZMG8_CHICK | 1.771296 | up |
| biological_process | response to stimulus | tr|Q5ZMJ6|Q5ZMJ6_CHICK | 1.845871 | up |
| biological_process | response to stimulus | tr|Q5ZMP2|Q5ZMP2_CHICK | 1.165624 | up |
| biological_process | response to stimulus | tr|Q9DDD4|Q9DDD4_CHICK | -1.44831 | down |
| biological_process | response to stimulus | tr|Q9PUJ4|Q9PUJ4_CHICK | 1.664957 | up |
| biological_process | response to stimulus | tr|R4GI86|R4GI86_CHICK | 2.078653 | up |
| biological_process | response to stimulus | tr|R4GMH5|R4GMH5_CHICK | -2.32193 | down |
| biological_process | rhythmic process | tr|A0A1D5PIT4|A0A1D5PIT4_CHICK | -1.97988 | down |
| biological_process | rhythmic process | tr|E1BX21|E1BX21_CHICK | 1.42634 | up |
| biological_process | rhythmic process | tr|I1SV11|I1SV11_CHICK | 1.313667 | up |
| biological_process | rhythmic process | tr|Q2PUH1|Q2PUH1_CHICK | 1.468226 | up |
| biological_process | signaling | sp|O93436|STAM2_CHICK | 2.376336 | up |
| biological_process | signaling | sp|P00523|SRC_CHICK | 1.599352 | up |
| biological_process | signaling | sp|P25155|FA10_CHICK | -3.77432 | down |
| biological_process | signaling | sp|Q5ZIK2|PDZ11_CHICK | 1.259402 | up |
| biological_process | signaling | sp|Q5ZJW6|KBRS2_CHICK | 1.256362 | up |
| biological_process | signaling | sp|Q5ZK05|TOLIP_CHICK | 1.088317 | up |
| biological_process | signaling | sp|Q5ZL36|ZFY27_CHICK | 1.948763 | up |
| biological_process | signaling | sp|Q5ZM83|MIRO2_CHICK | 1.659999 | up |
| biological_process | signaling | tr|A0A1D5NUD3|A0A1D5NUD3_CHICK | 1.402549 | up |
| biological_process | signaling | tr|A0A1D5NUD9|A0A1D5NUD9_CHICK | 1.157212 | up |
| biological_process | signaling | tr|A0A1D5NUU0|A0A1D5NUU0_CHICK | 1.000304 | up |
| biological_process | signaling | tr|A0A1D5NUV0|A0A1D5NUV0_CHICK | 2.157037 | up |
| biological_process | signaling | tr|A0A1D5NWV6|A0A1D5NWV6_CHICK | 1.553336 | up |
| biological_process | signaling | tr|A0A1D5NZB4|A0A1D5NZB4_CHICK | 1.671702 | up |
| biological_process | signaling | tr|A0A1D5P9G2|A0A1D5P9G2_CHICK | 1.768438 | up |
| biological_process | signaling | tr|A0A1D5PA08|A0A1D5PA08_CHICK | 1.946165 | up |
| biological_process | signaling | tr|A0A1D5PCZ1|A0A1D5PCZ1_CHICK | 1.542638 | up |
| biological_process | signaling | tr|A0A1D5PFS2|A0A1D5PFS2_CHICK | 1.463999 | up |
| biological_process | signaling | tr|A0A1D5PIB1|A0A1D5PIB1_CHICK | 2.10824 | up |
| biological_process | signaling | tr|A0A1D5PK72|A0A1D5PK72_CHICK | 2.032922 | up |
| biological_process | signaling | tr|A0A1D5PQD0|A0A1D5PQD0_CHICK | 1.314273 | up |
| biological_process | signaling | tr|A0A1D5PRI6|A0A1D5PRI6_CHICK | 1.580404 | up |
| biological_process | signaling | tr|A0A1D5PWD7|A0A1D5PWD7_CHICK | 1.676687 | up |
| biological_process | signaling | tr|A0A1D5PXN4|A0A1D5PXN4_CHICK | -1.85723 | down |
| biological_process | signaling | tr|A0A3Q2TUF8|A0A3Q2TUF8_CHICK | 1.02023 | up |
| biological_process | signaling | tr|A0A3Q2TY77|A0A3Q2TY77_CHICK | 1.82929 | up |
| biological_process | signaling | tr|A0A3Q2UCN1|A0A3Q2UCN1_CHICK | 1.92272 | up |
| biological_process | signaling | tr|A0A3Q3ALC8|A0A3Q3ALC8_CHICK | 2.188996 | up |
| biological_process | signaling | tr|E1BSH7|E1BSH7_CHICK | 1.527514 | up |
| biological_process | signaling | tr|E1BU89|E1BU89_CHICK | 1.303565 | up |
| biological_process | signaling | tr|E1BVQ3|E1BVQ3_CHICK | 1.650579 | up |
| biological_process | signaling | tr|E1BW27|E1BW27_CHICK | 1.938051 | up |
| biological_process | signaling | tr|E1BYQ3|E1BYQ3_CHICK | 1.202088 | up |
| biological_process | signaling | tr|E1C396|E1C396_CHICK | 1.08258 | up |
| biological_process | signaling | tr|E1C3U6|E1C3U6_CHICK | 1.144268 | up |
| biological_process | signaling | tr|E1C688|E1C688_CHICK | 1.775755 | up |
| biological_process | signaling | tr|E1C7P7|E1C7P7_CHICK | 2.341246 | up |
| biological_process | signaling | tr|E7EC82|E7EC82_CHICK | 1.586027 | up |
| biological_process | signaling | tr|F1N9Y3|F1N9Y3_CHICK | 1.33841 | up |
| biological_process | signaling | tr|F1NBX1|F1NBX1_CHICK | 1.439614 | up |
| biological_process | signaling | tr|F1NDH2|F1NDH2_CHICK | -1.95135 | down |
| biological_process | signaling | tr|F1NIY3|F1NIY3_CHICK | 1.568578 | up |
| biological_process | signaling | tr|F1NMJ9|F1NMJ9_CHICK | -2.4895 | down |
| biological_process | signaling | tr|F1NRZ4|F1NRZ4_CHICK | 1.14911 | up |
| biological_process | signaling | tr|F1NSQ4|F1NSQ4_CHICK | 1.21442 | up |
| biological_process | signaling | tr|F1NT80|F1NT80_CHICK | 1.404176 | up |
| biological_process | signaling | tr|F1NXV6|F1NXV6_CHICK | -4.87705 | down |
| biological_process | signaling | tr|F1P151|F1P151_CHICK | 2.603153 | up |
| biological_process | signaling | tr|F6YX81|F6YX81_CHICK | 1.194552 | up |
| biological_process | signaling | tr|H9L023|H9L023_CHICK | 1.212184 | up |
| biological_process | signaling | tr|I1SV11|I1SV11_CHICK | 1.313667 | up |
| biological_process | signaling | tr|O93466|O93466_CHICK | 1.669881 | up |
| biological_process | signaling | tr|Q2PUH1|Q2PUH1_CHICK | 1.468226 | up |
| biological_process | signaling | tr|Q5F3R8|Q5F3R8_CHICK | 2.332553 | up |
| biological_process | signaling | tr|Q5F3X1|Q5F3X1_CHICK | 1.19047 | up |
| biological_process | signaling | tr|Q5ZIL3|Q5ZIL3_CHICK | 1.863958 | up |
| biological_process | signaling | tr|Q5ZJ19|Q5ZJ19_CHICK | 2.470646 | up |
| biological_process | signaling | tr|Q5ZJQ4|Q5ZJQ4_CHICK | 1.678561 | up |
| biological_process | signaling | tr|Q5ZKI3|Q5ZKI3_CHICK | 1.326993 | up |
| biological_process | signaling | tr|Q5ZMG8|Q5ZMG8_CHICK | 1.771296 | up |
| biological_process | signaling | tr|Q9PUJ4|Q9PUJ4_CHICK | 1.664957 | up |
| biological_process | signaling | tr|R4GI86|R4GI86_CHICK | 2.078653 | up |
| cellular_component | cell | sp|O42395|CNBP_CHICK | 1.365125 | up |
| cellular_component | cell | sp|O93436|STAM2_CHICK | 2.376336 | up |
| cellular_component | cell | sp|O93602|ATF2_CHICK | 2.071233 | up |
| cellular_component | cell | sp|P00337|LDHB_CHICK | 2.131478 | up |
| cellular_component | cell | sp|P00523|SRC_CHICK | 1.599352 | up |
| cellular_component | cell | sp|P00940|TPIS_CHICK | 2.162937 | up |
| cellular_component | cell | sp|P07090|CALB2_CHICK | 1.954973 | up |
| cellular_component | cell | sp|P17923|NFIA_CHICK | 1.346459 | up |
| cellular_component | cell | sp|P18936|NU1M_CHICK | 2.034658 | up |
| cellular_component | cell | sp|P20136|GSTM2_CHICK | 2.226935 | up |
| cellular_component | cell | sp|P41366|VMO1_CHICK | -5.73258 | down |
| cellular_component | cell | sp|P42292|CD166_CHICK | 1.297212 | up |
| cellular_component | cell | sp|P52162|MAX_CHICK | 1.334512 | up |
| cellular_component | cell | sp|Q05423|FABP7_CHICK | 1.9874 | up |
| cellular_component | cell | sp|Q5F3K4|WDR48_CHICK | 1.71564 | up |
| cellular_component | cell | sp|Q5F3Z3|UB2V2_CHICK | 2.366484 | up |
| cellular_component | cell | sp|Q5F480|ITPK1_CHICK | 1.066359 | up |
| cellular_component | cell | sp|Q5ZHN3|WIPI2_CHICK | 1.61465 | up |
| cellular_component | cell | sp|Q5ZHT1|ACD11_CHICK | 1.653324 | up |
| cellular_component | cell | sp|Q5ZI08|SPT5H_CHICK | 1.235361 | up |
| cellular_component | cell | sp|Q5ZI74|DHX30_CHICK | 1.265146 | up |
| cellular_component | cell | sp|Q5ZIB9|ANM7_CHICK | 1.168118 | up |
| cellular_component | cell | sp|Q5ZID0|NMRL1_CHICK | 2.429369 | up |
| cellular_component | cell | sp|Q5ZIF1|APMAP_CHICK | 1.008442 | up |
| cellular_component | cell | sp|Q5ZII9|TFP11_CHICK | 1.172107 | up |
| cellular_component | cell | sp|Q5ZIK0|P4K2B_CHICK | 2.036191 | up |
| cellular_component | cell | sp|Q5ZIK2|PDZ11_CHICK | 1.259402 | up |
| cellular_component | cell | sp|Q5ZIP4|XRN2_CHICK | 1.286156 | up |
| cellular_component | cell | sp|Q5ZJ00|EM55_CHICK | 1.664901 | up |
| cellular_component | cell | sp|Q5ZJ08|SYYC_CHICK | 1.23041 | up |
| cellular_component | cell | sp|Q5ZJA9|BORC5_CHICK | 1.655713 | up |
| cellular_component | cell | sp|Q5ZJB7|CHMP7_CHICK | 1.815716 | up |
| cellular_component | cell | sp|Q5ZJH9|DKC1_CHICK | 1.436701 | up |
| cellular_component | cell | sp|Q5ZJJ8|UBCP1_CHICK | 2.077308 | up |
| cellular_component | cell | sp|Q5ZJW6|KBRS2_CHICK | 1.256362 | up |
| cellular_component | cell | sp|Q5ZK05|TOLIP_CHICK | 1.088317 | up |
| cellular_component | cell | sp|Q5ZK92|SPAST_CHICK | 1.665787 | up |
| cellular_component | cell | sp|Q5ZKA3|CWC22_CHICK | 1.734 | up |
| cellular_component | cell | sp|Q5ZKV4|NUBP2_CHICK | 1.740378 | up |
| cellular_component | cell | sp|Q5ZKV9|VPS50_CHICK | 1.324318 | up |
| cellular_component | cell | sp|Q5ZL36|ZFY27_CHICK | 1.948763 | up |
| cellular_component | cell | sp|Q5ZLD4|TMM11_CHICK | 1.73923 | up |
| cellular_component | cell | sp|Q5ZLT0|XPO7_CHICK | 1.469766 | up |
| cellular_component | cell | sp|Q5ZLV2|PPIL3_CHICK | 1.29867 | up |
| cellular_component | cell | sp|Q5ZM83|MIRO2_CHICK | 1.659999 | up |
| cellular_component | cell | sp|Q5ZMG0|DOPD_CHICK | 2.121364 | up |
| cellular_component | cell | sp|Q5ZML0|BABA2_CHICK | 1.144731 | up |
| cellular_component | cell | sp|Q8AXL1|SAT1_CHICK | 1.12819 | up |
| cellular_component | cell | sp|Q90733|COT2_CHICK | 1.595685 | up |
| cellular_component | cell | sp|Q9I8D0|VPP1_CHICK | 1.736096 | up |
| cellular_component | cell | sp|Q9IAM7|MRE11_CHICK | 1.337957 | up |
| cellular_component | cell | tr|A0A1D5NT70|A0A1D5NT70_CHICK | 1.819215 | up |
| cellular_component | cell | tr|A0A1D5NTC8|A0A1D5NTC8_CHICK | 1.184237 | up |
| cellular_component | cell | tr|A0A1D5NTR1|A0A1D5NTR1_CHICK | 1.213885 | up |
| cellular_component | cell | tr|A0A1D5NU82|A0A1D5NU82_CHICK | 1.12312 | up |
| cellular_component | cell | tr|A0A1D5NUD3|A0A1D5NUD3_CHICK | 1.402549 | up |
| cellular_component | cell | tr|A0A1D5NUD9|A0A1D5NUD9_CHICK | 1.157212 | up |
| cellular_component | cell | tr|A0A1D5NUK5|A0A1D5NUK5_CHICK | 2.534566 | up |
| cellular_component | cell | tr|A0A1D5NUV0|A0A1D5NUV0_CHICK | 2.157037 | up |
| cellular_component | cell | tr|A0A1D5NVT0|A0A1D5NVT0_CHICK | 1.421036 | up |
| cellular_component | cell | tr|A0A1D5NWJ7|A0A1D5NWJ7_CHICK | 1.195763 | up |
| cellular_component | cell | tr|A0A1D5NWV6|A0A1D5NWV6_CHICK | 1.553336 | up |
| cellular_component | cell | tr|A0A1D5NXG4|A0A1D5NXG4_CHICK | 1.245324 | up |
| cellular_component | cell | tr|A0A1D5NXR2|A0A1D5NXR2_CHICK | 1.37138 | up |
| cellular_component | cell | tr|A0A1D5NXS8|A0A1D5NXS8_CHICK | 1.203865 | up |
| cellular_component | cell | tr|A0A1D5NZB4|A0A1D5NZB4_CHICK | 1.671702 | up |
| cellular_component | cell | tr|A0A1D5NZS7|A0A1D5NZS7_CHICK | 1.185694 | up |
| cellular_component | cell | tr|A0A1D5P047|A0A1D5P047_CHICK | 1.659689 | up |
| cellular_component | cell | tr|A0A1D5P1D5|A0A1D5P1D5_CHICK | 2.084726 | up |
| cellular_component | cell | tr|A0A1D5P453|A0A1D5P453_CHICK | 1.553695 | up |
| cellular_component | cell | tr|A0A1D5P4B1|A0A1D5P4B1_CHICK | 1.156059 | up |
| cellular_component | cell | tr|A0A1D5P5X4|A0A1D5P5X4_CHICK | 1.559711 | up |
| cellular_component | cell | tr|A0A1D5P607|A0A1D5P607_CHICK | 1.51904 | up |
| cellular_component | cell | tr|A0A1D5P6C3|A0A1D5P6C3_CHICK | 2.100747 | up |
| cellular_component | cell | tr|A0A1D5P6L7|A0A1D5P6L7_CHICK | 1.520092 | up |
| cellular_component | cell | tr|A0A1D5P6M8|A0A1D5P6M8_CHICK | 1.82889 | up |
| cellular_component | cell | tr|A0A1D5P6S3|A0A1D5P6S3_CHICK | 1.183172 | up |
| cellular_component | cell | tr|A0A1D5P6Y6|A0A1D5P6Y6_CHICK | 1.32218 | up |
| cellular_component | cell | tr|A0A1D5P7C5|A0A1D5P7C5_CHICK | 1.273318 | up |
| cellular_component | cell | tr|A0A1D5P7E3|A0A1D5P7E3_CHICK | 1.017643 | up |
| cellular_component | cell | tr|A0A1D5P7Q4|A0A1D5P7Q4_CHICK | 1.066394 | up |
| cellular_component | cell | tr|A0A1D5P822|A0A1D5P822_CHICK | 1.478805 | up |
| cellular_component | cell | tr|A0A1D5P8H7|A0A1D5P8H7_CHICK | 1.530513 | up |
| cellular_component | cell | tr|A0A1D5P8V8|A0A1D5P8V8_CHICK | 1.608897 | up |
| cellular_component | cell | tr|A0A1D5P997|A0A1D5P997_CHICK | 1.563709 | up |
| cellular_component | cell | tr|A0A1D5P9G2|A0A1D5P9G2_CHICK | 1.768438 | up |
| cellular_component | cell | tr|A0A1D5PA08|A0A1D5PA08_CHICK | 1.946165 | up |
| cellular_component | cell | tr|A0A1D5PAM1|A0A1D5PAM1_CHICK | 2.577291 | up |
| cellular_component | cell | tr|A0A1D5PC23|A0A1D5PC23_CHICK | 2.344267 | up |
| cellular_component | cell | tr|A0A1D5PCZ1|A0A1D5PCZ1_CHICK | 1.542638 | up |
| cellular_component | cell | tr|A0A1D5PDV1|A0A1D5PDV1_CHICK | 1.384898 | up |
| cellular_component | cell | tr|A0A1D5PEE5|A0A1D5PEE5_CHICK | 1.102471 | up |
| cellular_component | cell | tr|A0A1D5PF37|A0A1D5PF37_CHICK | 1.238601 | up |
| cellular_component | cell | tr|A0A1D5PFS2|A0A1D5PFS2_CHICK | 1.463999 | up |
| cellular_component | cell | tr|A0A1D5PGB1|A0A1D5PGB1_CHICK | 1.43532 | up |
| cellular_component | cell | tr|A0A1D5PIB1|A0A1D5PIB1_CHICK | 2.10824 | up |
| cellular_component | cell | tr|A0A1D5PJB9|A0A1D5PJB9_CHICK | 1.548287 | up |
| cellular_component | cell | tr|A0A1D5PKQ7|A0A1D5PKQ7_CHICK | -1.34664 | down |
| cellular_component | cell | tr|A0A1D5PM87|A0A1D5PM87_CHICK | 1.108975 | up |
| cellular_component | cell | tr|A0A1D5PMR8|A0A1D5PMR8_CHICK | 1.213883 | up |
| cellular_component | cell | tr|A0A1D5PQ66|A0A1D5PQ66_CHICK | 1.664552 | up |
| cellular_component | cell | tr|A0A1D5PQC1|A0A1D5PQC1_CHICK | 1.667257 | up |
| cellular_component | cell | tr|A0A1D5PQD0|A0A1D5PQD0_CHICK | 1.314273 | up |
| cellular_component | cell | tr|A0A1D5PQG6|A0A1D5PQG6_CHICK | 2.091788 | up |
| cellular_component | cell | tr|A0A1D5PQQ6|A0A1D5PQQ6_CHICK | 1.432587 | up |
| cellular_component | cell | tr|A0A1D5PR34|A0A1D5PR34_CHICK | 1.370909 | up |
| cellular_component | cell | tr|A0A1D5PRI4|A0A1D5PRI4_CHICK | 1.28486 | up |
| cellular_component | cell | tr|A0A1D5PRI6|A0A1D5PRI6_CHICK | 1.580404 | up |
| cellular_component | cell | tr|A0A1D5PTE8|A0A1D5PTE8_CHICK | 1.960486 | up |
| cellular_component | cell | tr|A0A1D5PTW3|A0A1D5PTW3_CHICK | 1.808599 | up |
| cellular_component | cell | tr|A0A1D5PUE2|A0A1D5PUE2_CHICK | 2.043282 | up |
| cellular_component | cell | tr|A0A1D5PUJ5|A0A1D5PUJ5_CHICK | 1.266991 | up |
| cellular_component | cell | tr|A0A1D5PWD7|A0A1D5PWD7_CHICK | 1.676687 | up |
| cellular_component | cell | tr|A0A1D5PWT4|A0A1D5PWT4_CHICK | 1.101328 | up |
| cellular_component | cell | tr|A0A1D5PXN4|A0A1D5PXN4_CHICK | -1.85723 | down |
| cellular_component | cell | tr|A0A1D5PYU0|A0A1D5PYU0_CHICK | 1.138573 | up |
| cellular_component | cell | tr|A0A1D5PZ78|A0A1D5PZ78_CHICK | 1.936124 | up |
| cellular_component | cell | tr|A0A1D5PZF2|A0A1D5PZF2_CHICK | 1.668934 | up |
| cellular_component | cell | tr|A0A1D5PZL3|A0A1D5PZL3_CHICK | 1.786305 | up |
| cellular_component | cell | tr|A0A1D6UPT6|A0A1D6UPT6_CHICK | 1.170381 | up |
| cellular_component | cell | tr|A0A1L1RIY5|A0A1L1RIY5_CHICK | 751.4932 | up |
| cellular_component | cell | tr|A0A1L1RL50|A0A1L1RL50_CHICK | 1.173616 | up |
| cellular_component | cell | tr|A0A1L1RPB1|A0A1L1RPB1_CHICK | 1.864507 | up |
| cellular_component | cell | tr|A0A1L1RPT9|A0A1L1RPT9_CHICK | 1.353636 | up |
| cellular_component | cell | tr|A0A1L1RSG1|A0A1L1RSG1_CHICK | 1.889712 | up |
| cellular_component | cell | tr|A0A1L1RSS5|A0A1L1RSS5_CHICK | 1.143155 | up |
| cellular_component | cell | tr|A0A1L1RXA3|A0A1L1RXA3_CHICK | 1.394002 | up |
| cellular_component | cell | tr|A0A3Q2TRS4|A0A3Q2TRS4_CHICK | 1.770416 | up |
| cellular_component | cell | tr|A0A3Q2TUF8|A0A3Q2TUF8_CHICK | 1.02023 | up |
| cellular_component | cell | tr|A0A3Q2TUN2|A0A3Q2TUN2_CHICK | 1.036105 | up |
| cellular_component | cell | tr|A0A3Q2TVU0|A0A3Q2TVU0_CHICK | 1.280793 | up |
| cellular_component | cell | tr|A0A3Q2TVV0|A0A3Q2TVV0_CHICK | 1.145618 | up |
| cellular_component | cell | tr|A0A3Q2TY77|A0A3Q2TY77_CHICK | 1.82929 | up |
| cellular_component | cell | tr|A0A3Q2TYP7|A0A3Q2TYP7_CHICK | 2.159057 | up |
| cellular_component | cell | tr|A0A3Q2TZM7|A0A3Q2TZM7_CHICK | 1.419267 | up |
| cellular_component | cell | tr|A0A3Q2U067|A0A3Q2U067_CHICK | 1.508572 | up |
| cellular_component | cell | tr|A0A3Q2U3C4|A0A3Q2U3C4_CHICK | 1.094483 | up |
| cellular_component | cell | tr|A0A3Q2U853|A0A3Q2U853_CHICK | 1.530605 | up |
| cellular_component | cell | tr|A0A3Q2U8I0|A0A3Q2U8I0_CHICK | 1.050068 | up |
| cellular_component | cell | tr|A0A3Q2UB58|A0A3Q2UB58_CHICK | 2.070855 | up |
| cellular_component | cell | tr|A0A3Q2UBJ7|A0A3Q2UBJ7_CHICK | 1.945958 | up |
| cellular_component | cell | tr|A0A3Q2UCG6|A0A3Q2UCG6_CHICK | 1.033099 | up |
| cellular_component | cell | tr|A0A3Q2UCN1|A0A3Q2UCN1_CHICK | 1.92272 | up |
| cellular_component | cell | tr|A0A3Q2UCU2|A0A3Q2UCU2_CHICK | 1.557712 | up |
| cellular_component | cell | tr|A0A3Q2UD87|A0A3Q2UD87_CHICK | 1.205498 | up |
| cellular_component | cell | tr|A0A3Q2UM13|A0A3Q2UM13_CHICK | 3.560203 | up |
| cellular_component | cell | tr|A0A3Q2UPF3|A0A3Q2UPF3_CHICK | 1.295517 | up |
| cellular_component | cell | tr|A0A3Q3AB84|A0A3Q3AB84_CHICK | 1.170717 | up |
| cellular_component | cell | tr|A0A3Q3ALC8|A0A3Q3ALC8_CHICK | 2.188996 | up |
| cellular_component | cell | tr|A0A3S5ZPH2|A0A3S5ZPH2_CHICK | 1.677715 | up |
| cellular_component | cell | tr|A2T4N8|A2T4N8_CHICK | 1.116095 | up |
| cellular_component | cell | tr|A8VIF4|A8VIF4_CHICK | 1.024165 | up |
| cellular_component | cell | tr|D2D3P4|D2D3P4_CHICK | 2.146381 | up |
| cellular_component | cell | tr|E1BQC2|E1BQC2_CHICK | -2.52084 | down |
| cellular_component | cell | tr|E1BQI0|E1BQI0_CHICK | 1.067708 | up |
| cellular_component | cell | tr|E1BQU2|E1BQU2_CHICK | 1.629018 | up |
| cellular_component | cell | tr|E1BQW2|E1BQW2_CHICK | 1.823966 | up |
| cellular_component | cell | tr|E1BRQ9|E1BRQ9_CHICK | 1.034807 | up |
| cellular_component | cell | tr|E1BRR6|E1BRR6_CHICK | 1.332531 | up |
| cellular_component | cell | tr|E1BS31|E1BS31_CHICK | 1.448795 | up |
| cellular_component | cell | tr|E1BSH7|E1BSH7_CHICK | 1.527514 | up |
| cellular_component | cell | tr|E1BT44|E1BT44_CHICK | 1.432225 | up |
| cellular_component | cell | tr|E1BT94|E1BT94_CHICK | 1.49966 | up |
| cellular_component | cell | tr|E1BTE2|E1BTE2_CHICK | -2.67874 | down |
| cellular_component | cell | tr|E1BTI7|E1BTI7_CHICK | 1.813027 | up |
| cellular_component | cell | tr|E1BTS3|E1BTS3_CHICK | 1.090627 | up |
| cellular_component | cell | tr|E1BTT8|E1BTT8_CHICK | 1.708105 | up |
| cellular_component | cell | tr|E1BTV1|E1BTV1_CHICK | 2.001762 | up |
| cellular_component | cell | tr|E1BU27|E1BU27_CHICK | 3.035848 | up |
| cellular_component | cell | tr|E1BU89|E1BU89_CHICK | 1.303565 | up |
| cellular_component | cell | tr|E1BUJ1|E1BUJ1_CHICK | 2.709557 | up |
| cellular_component | cell | tr|E1BUX5|E1BUX5_CHICK | 1.099122 | up |
| cellular_component | cell | tr|E1BV97|E1BV97_CHICK | 2.036483 | up |
| cellular_component | cell | tr|E1BVC8|E1BVC8_CHICK | 2.179244 | up |
| cellular_component | cell | tr|E1BVQ3|E1BVQ3_CHICK | 1.650579 | up |
| cellular_component | cell | tr|E1BW27|E1BW27_CHICK | 1.938051 | up |
| cellular_component | cell | tr|E1BWB7|E1BWB7_CHICK | 1.446045 | up |
| cellular_component | cell | tr|E1BX21|E1BX21_CHICK | 1.42634 | up |
| cellular_component | cell | tr|E1BX85|E1BX85_CHICK | 2.029343 | up |
| cellular_component | cell | tr|E1BXC2|E1BXC2_CHICK | 1.767478 | up |
| cellular_component | cell | tr|E1BXS1|E1BXS1_CHICK | 1.514054 | up |
| cellular_component | cell | tr|E1BXT9|E1BXT9_CHICK | 1.600608 | up |
| cellular_component | cell | tr|E1BXY8|E1BXY8_CHICK | 1.106549 | up |
| cellular_component | cell | tr|E1BY22|E1BY22_CHICK | 1.367672 | up |
| cellular_component | cell | tr|E1BY40|E1BY40_CHICK | 1.129106 | up |
| cellular_component | cell | tr|E1BYQ3|E1BYQ3_CHICK | 1.202088 | up |
| cellular_component | cell | tr|E1BZ79|E1BZ79_CHICK | 2.388844 | up |
| cellular_component | cell | tr|E1C1T2|E1C1T2_CHICK | 1.594659 | up |
| cellular_component | cell | tr|E1C1Y5|E1C1Y5_CHICK | 1.135922 | up |
| cellular_component | cell | tr|E1C229|E1C229_CHICK | 1.473036 | up |
| cellular_component | cell | tr|E1C2E5|E1C2E5_CHICK | 1.182299 | up |
| cellular_component | cell | tr|E1C2F2|E1C2F2_CHICK | 1.546061 | up |
| cellular_component | cell | tr|E1C310|E1C310_CHICK | 1.545191 | up |
| cellular_component | cell | tr|E1C312|E1C312_CHICK | 1.811403 | up |
| cellular_component | cell | tr|E1C396|E1C396_CHICK | 1.08258 | up |
| cellular_component | cell | tr|E1C3U6|E1C3U6_CHICK | 1.144268 | up |
| cellular_component | cell | tr|E1C593|E1C593_CHICK | 1.922793 | up |
| cellular_component | cell | tr|E1C688|E1C688_CHICK | 1.775755 | up |
| cellular_component | cell | tr|E1C6S7|E1C6S7_CHICK | 1.1895 | up |
| cellular_component | cell | tr|E1C6U2|E1C6U2_CHICK | -1.17102 | down |
| cellular_component | cell | tr|E1C6V0|E1C6V0_CHICK | 1.577098 | up |
| cellular_component | cell | tr|E1C7C1|E1C7C1_CHICK | -1.84307 | down |
| cellular_component | cell | tr|E1C7J8|E1C7J8_CHICK | 1.184879 | up |
| cellular_component | cell | tr|E1C7P7|E1C7P7_CHICK | 2.341246 | up |
| cellular_component | cell | tr|E1C8A2|E1C8A2_CHICK | 1.307606 | up |
| cellular_component | cell | tr|E1C8U1|E1C8U1_CHICK | 1.411305 | up |
| cellular_component | cell | tr|E1C9H5|E1C9H5_CHICK | 1.195027 | up |
| cellular_component | cell | tr|E7EC82|E7EC82_CHICK | 1.586027 | up |
| cellular_component | cell | tr|F1N832|F1N832_CHICK | 1.427435 | up |
| cellular_component | cell | tr|F1N8T0|F1N8T0_CHICK | 1.514696 | up |
| cellular_component | cell | tr|F1N8V5|F1N8V5_CHICK | 2.344853 | up |
| cellular_component | cell | tr|F1N977|F1N977_CHICK | 1.720618 | up |
| cellular_component | cell | tr|F1N9S3|F1N9S3_CHICK | 1.256683 | up |
| cellular_component | cell | tr|F1N9U0|F1N9U0_CHICK | 1.038918 | up |
| cellular_component | cell | tr|F1N9Y3|F1N9Y3_CHICK | 1.33841 | up |
| cellular_component | cell | tr|F1NBX1|F1NBX1_CHICK | 1.439614 | up |
| cellular_component | cell | tr|F1NBY5|F1NBY5_CHICK | 1.813459 | up |
| cellular_component | cell | tr|F1NEA1|F1NEA1_CHICK | 1.395721 | up |
| cellular_component | cell | tr|F1NEQ3|F1NEQ3_CHICK | 1.139952 | up |
| cellular_component | cell | tr|F1NF87|F1NF87_CHICK | 1.015859 | up |
| cellular_component | cell | tr|F1NH23|F1NH23_CHICK | 1.850288 | up |
| cellular_component | cell | tr|F1NIK3|F1NIK3_CHICK | 1.065249 | up |
| cellular_component | cell | tr|F1NIQ3|F1NIQ3_CHICK | 1.483149 | up |
| cellular_component | cell | tr|F1NIY3|F1NIY3_CHICK | 1.568578 | up |
| cellular_component | cell | tr|F1NJK4|F1NJK4_CHICK | 1.788595 | up |
| cellular_component | cell | tr|F1NJU5|F1NJU5_CHICK | -1.49981 | down |
| cellular_component | cell | tr|F1NKY6|F1NKY6_CHICK | 1.171068 | up |
| cellular_component | cell | tr|F1NMJ9|F1NMJ9_CHICK | -2.4895 | down |
| cellular_component | cell | tr|F1NMU0|F1NMU0_CHICK | 2.467729 | up |
| cellular_component | cell | tr|F1NN63|F1NN63_CHICK | 1.72615 | up |
| cellular_component | cell | tr|F1NPS5|F1NPS5_CHICK | 3.319197 | up |
| cellular_component | cell | tr|F1NQ61|F1NQ61_CHICK | 1.080306 | up |
| cellular_component | cell | tr|F1NRZ4|F1NRZ4_CHICK | 1.14911 | up |
| cellular_component | cell | tr|F1NSD6|F1NSD6_CHICK | 1.689363 | up |
| cellular_component | cell | tr|F1NSF2|F1NSF2_CHICK | 1.708711 | up |
| cellular_component | cell | tr|F1NSI7|F1NSI7_CHICK | 1.77407 | up |
| cellular_component | cell | tr|F1NSQ4|F1NSQ4_CHICK | 1.21442 | up |
| cellular_component | cell | tr|F1NT58|F1NT58_CHICK | 1.701877 | up |
| cellular_component | cell | tr|F1NT80|F1NT80_CHICK | 1.404176 | up |
| cellular_component | cell | tr|F1NTQ2|F1NTQ2_CHICK | 1.236275 | up |
| cellular_component | cell | tr|F1NUY0|F1NUY0_CHICK | 1.570572 | up |
| cellular_component | cell | tr|F1NUY9|F1NUY9_CHICK | 1.592258 | up |
| cellular_component | cell | tr|F1NV66|F1NV66_CHICK | 1.865555 | up |
| cellular_component | cell | tr|F1NXV6|F1NXV6_CHICK | -4.87705 | down |
| cellular_component | cell | tr|F1NXZ7|F1NXZ7_CHICK | 1.050248 | up |
| cellular_component | cell | tr|F1NYG7|F1NYG7_CHICK | 1.537967 | up |
| cellular_component | cell | tr|F1P151|F1P151_CHICK | 2.603153 | up |
| cellular_component | cell | tr|F1P204|F1P204_CHICK | 2.129437 | up |
| cellular_component | cell | tr|F1P278|F1P278_CHICK | 1.88205 | up |
| cellular_component | cell | tr|F1P2G6|F1P2G6_CHICK | 1.470473 | up |
| cellular_component | cell | tr|F1P372|F1P372_CHICK | 1.525043 | up |
| cellular_component | cell | tr|F1P394|F1P394_CHICK | 1.286828 | up |
| cellular_component | cell | tr|F1P5A3|F1P5A3_CHICK | 2.127136 | up |
| cellular_component | cell | tr|F1P5B8|F1P5B8_CHICK | 1.715588 | up |
| cellular_component | cell | tr|F1P5J7|F1P5J7_CHICK | 1.075835 | up |
| cellular_component | cell | tr|F1P5X6|F1P5X6_CHICK | 1.295882 | up |
| cellular_component | cell | tr|F6R228|F6R228_CHICK | 1.426396 | up |
| cellular_component | cell | tr|F6YX81|F6YX81_CHICK | 1.194552 | up |
| cellular_component | cell | tr|F7BFS9|F7BFS9_CHICK | 1.641443 | up |
| cellular_component | cell | tr|F7BYG6|F7BYG6_CHICK | 3.125856 | up |
| cellular_component | cell | tr|H9L0M7|H9L0M7_CHICK | 1.164684 | up |
| cellular_component | cell | tr|I1SV11|I1SV11_CHICK | 1.313667 | up |
| cellular_component | cell | tr|M9NCD4|M9NCD4_CHICK | 1.22338 | up |
| cellular_component | cell | tr|O93466|O93466_CHICK | 1.669881 | up |
| cellular_component | cell | tr|Q2PUH1|Q2PUH1_CHICK | 1.468226 | up |
| cellular_component | cell | tr|Q4ADJ6|Q4ADJ6_CHICK | -4.04825 | down |
| cellular_component | cell | tr|Q5DWF6|Q5DWF6_CHICK | 1.289716 | up |
| cellular_component | cell | tr|Q5F351|Q5F351_CHICK | 1.342446 | up |
| cellular_component | cell | tr|Q5F3R8|Q5F3R8_CHICK | 2.332553 | up |
| cellular_component | cell | tr|Q5F3X1|Q5F3X1_CHICK | 1.19047 | up |
| cellular_component | cell | tr|Q5F4B6|Q5F4B6_CHICK | 1.904086 | up |
| cellular_component | cell | tr|Q5QRU7|Q5QRU7_CHICK | 2.017795 | up |
| cellular_component | cell | tr|Q5ZHP4|Q5ZHP4_CHICK | 2.101948 | up |
| cellular_component | cell | tr|Q5ZIL3|Q5ZIL3_CHICK | 1.863958 | up |
| cellular_component | cell | tr|Q5ZIZ3|Q5ZIZ3_CHICK | 1.248317 | up |
| cellular_component | cell | tr|Q5ZJ19|Q5ZJ19_CHICK | 2.470646 | up |
| cellular_component | cell | tr|Q5ZJ96|Q5ZJ96_CHICK | 1.306532 | up |
| cellular_component | cell | tr|Q5ZJA8|Q5ZJA8_CHICK | 1.093474 | up |
| cellular_component | cell | tr|Q5ZJQ4|Q5ZJQ4_CHICK | 1.678561 | up |
| cellular_component | cell | tr|Q5ZJW2|Q5ZJW2_CHICK | 1.71086 | up |
| cellular_component | cell | tr|Q5ZKI3|Q5ZKI3_CHICK | 1.326993 | up |
| cellular_component | cell | tr|Q5ZKY5|Q5ZKY5_CHICK | 1.620297 | up |
| cellular_component | cell | tr|Q5ZL65|Q5ZL65_CHICK | 1.695268 | up |
| cellular_component | cell | tr|Q5ZLI0|Q5ZLI0_CHICK | 1.097879 | up |
| cellular_component | cell | tr|Q5ZMA7|Q5ZMA7_CHICK | 1.172026 | up |
| cellular_component | cell | tr|Q5ZMG8|Q5ZMG8_CHICK | 1.771296 | up |
| cellular_component | cell | tr|Q5ZMJ6|Q5ZMJ6_CHICK | 1.845871 | up |
| cellular_component | cell | tr|Q5ZMP2|Q5ZMP2_CHICK | 1.165624 | up |
| cellular_component | cell | tr|Q9DDD4|Q9DDD4_CHICK | -1.44831 | down |
| cellular_component | cell | tr|Q9I882|Q9I882_CHICK | 2.21964 | up |
| cellular_component | cell | tr|Q9I8C8|Q9I8C8_CHICK | 1.2712 | up |
| cellular_component | cell | tr|Q9PUJ4|Q9PUJ4_CHICK | 1.664957 | up |
| cellular_component | cell | tr|Q9PVL6|Q9PVL6_CHICK | 1.71949 | up |
| cellular_component | cell | tr|R4GHH2|R4GHH2_CHICK | 1.778292 | up |
| cellular_component | cell | tr|R4GHY3|R4GHY3_CHICK | 1.375649 | up |
| cellular_component | cell | tr|R4GI86|R4GI86_CHICK | 2.078653 | up |
| cellular_component | cell | tr|R4GJH2|R4GJH2_CHICK | 1.058464 | up |
| cellular_component | cell | tr|R4GLW9|R4GLW9_CHICK | 1.120506 | up |
| cellular_component | cell junction | sp|P07090|CALB2_CHICK | 1.954973 | up |
| cellular_component | cell junction | sp|P42292|CD166_CHICK | 1.297212 | up |
| cellular_component | cell junction | sp|Q05423|FABP7_CHICK | 1.9874 | up |
| cellular_component | cell junction | sp|Q5ZIK2|PDZ11_CHICK | 1.259402 | up |
| cellular_component | cell junction | tr|A0A1D5PQG6|A0A1D5PQG6_CHICK | 2.091788 | up |
| cellular_component | cell junction | tr|A0A1D5PWD7|A0A1D5PWD7_CHICK | 1.676687 | up |
| cellular_component | cell junction | tr|A0A3Q2UCN1|A0A3Q2UCN1_CHICK | 1.92272 | up |
| cellular_component | cell junction | tr|A8VIF4|A8VIF4_CHICK | 1.024165 | up |
| cellular_component | cell junction | tr|E1BRR6|E1BRR6_CHICK | 1.332531 | up |
| cellular_component | cell junction | tr|E1C2F2|E1C2F2_CHICK | 1.546061 | up |
| cellular_component | cell junction | tr|E1C9A8|E1C9A8_CHICK | 1.484292 | up |
| cellular_component | cell junction | tr|F1NEA1|F1NEA1_CHICK | 1.395721 | up |
| cellular_component | cell junction | tr|F1NMJ9|F1NMJ9_CHICK | -2.4895 | down |
| cellular_component | cell junction | tr|Q5F3R8|Q5F3R8_CHICK | 2.332553 | up |
| cellular_component | cell junction | tr|Q5ZJ19|Q5ZJ19_CHICK | 2.470646 | up |
| cellular_component | cell junction | tr|Q9PUJ4|Q9PUJ4_CHICK | 1.664957 | up |
| cellular_component | cell part | sp|O42395|CNBP_CHICK | 1.365125 | up |
| cellular_component | cell part | sp|O93436|STAM2_CHICK | 2.376336 | up |
| cellular_component | cell part | sp|O93602|ATF2_CHICK | 2.071233 | up |
| cellular_component | cell part | sp|P00337|LDHB_CHICK | 2.131478 | up |
| cellular_component | cell part | sp|P00523|SRC_CHICK | 1.599352 | up |
| cellular_component | cell part | sp|P00940|TPIS_CHICK | 2.162937 | up |
| cellular_component | cell part | sp|P07090|CALB2_CHICK | 1.954973 | up |
| cellular_component | cell part | sp|P17923|NFIA_CHICK | 1.346459 | up |
| cellular_component | cell part | sp|P18936|NU1M_CHICK | 2.034658 | up |
| cellular_component | cell part | sp|P20136|GSTM2_CHICK | 2.226935 | up |
| cellular_component | cell part | sp|P42292|CD166_CHICK | 1.297212 | up |
| cellular_component | cell part | sp|P52162|MAX_CHICK | 1.334512 | up |
| cellular_component | cell part | sp|Q05423|FABP7_CHICK | 1.9874 | up |
| cellular_component | cell part | sp|Q5F3K4|WDR48_CHICK | 1.71564 | up |
| cellular_component | cell part | sp|Q5F3Z3|UB2V2_CHICK | 2.366484 | up |
| cellular_component | cell part | sp|Q5F480|ITPK1_CHICK | 1.066359 | up |
| cellular_component | cell part | sp|Q5ZHN3|WIPI2_CHICK | 1.61465 | up |
| cellular_component | cell part | sp|Q5ZHT1|ACD11_CHICK | 1.653324 | up |
| cellular_component | cell part | sp|Q5ZI08|SPT5H_CHICK | 1.235361 | up |
| cellular_component | cell part | sp|Q5ZI74|DHX30_CHICK | 1.265146 | up |
| cellular_component | cell part | sp|Q5ZIB9|ANM7_CHICK | 1.168118 | up |
| cellular_component | cell part | sp|Q5ZID0|NMRL1_CHICK | 2.429369 | up |
| cellular_component | cell part | sp|Q5ZIF1|APMAP_CHICK | 1.008442 | up |
| cellular_component | cell part | sp|Q5ZII9|TFP11_CHICK | 1.172107 | up |
| cellular_component | cell part | sp|Q5ZIK0|P4K2B_CHICK | 2.036191 | up |
| cellular_component | cell part | sp|Q5ZIK2|PDZ11_CHICK | 1.259402 | up |
| cellular_component | cell part | sp|Q5ZIP4|XRN2_CHICK | 1.286156 | up |
| cellular_component | cell part | sp|Q5ZJ00|EM55_CHICK | 1.664901 | up |
| cellular_component | cell part | sp|Q5ZJ08|SYYC_CHICK | 1.23041 | up |
| cellular_component | cell part | sp|Q5ZJA9|BORC5_CHICK | 1.655713 | up |
| cellular_component | cell part | sp|Q5ZJB7|CHMP7_CHICK | 1.815716 | up |
| cellular_component | cell part | sp|Q5ZJH9|DKC1_CHICK | 1.436701 | up |
| cellular_component | cell part | sp|Q5ZJJ8|UBCP1_CHICK | 2.077308 | up |
| cellular_component | cell part | sp|Q5ZJW6|KBRS2_CHICK | 1.256362 | up |
| cellular_component | cell part | sp|Q5ZK05|TOLIP_CHICK | 1.088317 | up |
| cellular_component | cell part | sp|Q5ZK92|SPAST_CHICK | 1.665787 | up |
| cellular_component | cell part | sp|Q5ZKA3|CWC22_CHICK | 1.734 | up |
| cellular_component | cell part | sp|Q5ZKV4|NUBP2_CHICK | 1.740378 | up |
| cellular_component | cell part | sp|Q5ZKV9|VPS50_CHICK | 1.324318 | up |
| cellular_component | cell part | sp|Q5ZL36|ZFY27_CHICK | 1.948763 | up |
| cellular_component | cell part | sp|Q5ZLD4|TMM11_CHICK | 1.73923 | up |
| cellular_component | cell part | sp|Q5ZLT0|XPO7_CHICK | 1.469766 | up |
| cellular_component | cell part | sp|Q5ZLV2|PPIL3_CHICK | 1.29867 | up |
| cellular_component | cell part | sp|Q5ZM83|MIRO2_CHICK | 1.659999 | up |
| cellular_component | cell part | sp|Q5ZMG0|DOPD_CHICK | 2.121364 | up |
| cellular_component | cell part | sp|Q5ZML0|BABA2_CHICK | 1.144731 | up |
| cellular_component | cell part | sp|Q8AXL1|SAT1_CHICK | 1.12819 | up |
| cellular_component | cell part | sp|Q90733|COT2_CHICK | 1.595685 | up |
| cellular_component | cell part | sp|Q9I8D0|VPP1_CHICK | 1.736096 | up |
| cellular_component | cell part | sp|Q9IAM7|MRE11_CHICK | 1.337957 | up |
| cellular_component | cell part | tr|A0A1D5NT70|A0A1D5NT70_CHICK | 1.819215 | up |
| cellular_component | cell part | tr|A0A1D5NTC8|A0A1D5NTC8_CHICK | 1.184237 | up |
| cellular_component | cell part | tr|A0A1D5NTR1|A0A1D5NTR1_CHICK | 1.213885 | up |
| cellular_component | cell part | tr|A0A1D5NU82|A0A1D5NU82_CHICK | 1.12312 | up |
| cellular_component | cell part | tr|A0A1D5NUD3|A0A1D5NUD3_CHICK | 1.402549 | up |
| cellular_component | cell part | tr|A0A1D5NUD9|A0A1D5NUD9_CHICK | 1.157212 | up |
| cellular_component | cell part | tr|A0A1D5NUK5|A0A1D5NUK5_CHICK | 2.534566 | up |
| cellular_component | cell part | tr|A0A1D5NUV0|A0A1D5NUV0_CHICK | 2.157037 | up |
| cellular_component | cell part | tr|A0A1D5NVT0|A0A1D5NVT0_CHICK | 1.421036 | up |
| cellular_component | cell part | tr|A0A1D5NWJ7|A0A1D5NWJ7_CHICK | 1.195763 | up |
| cellular_component | cell part | tr|A0A1D5NWV6|A0A1D5NWV6_CHICK | 1.553336 | up |
| cellular_component | cell part | tr|A0A1D5NXG4|A0A1D5NXG4_CHICK | 1.245324 | up |
| cellular_component | cell part | tr|A0A1D5NXR2|A0A1D5NXR2_CHICK | 1.37138 | up |
| cellular_component | cell part | tr|A0A1D5NXS8|A0A1D5NXS8_CHICK | 1.203865 | up |
| cellular_component | cell part | tr|A0A1D5NZB4|A0A1D5NZB4_CHICK | 1.671702 | up |
| cellular_component | cell part | tr|A0A1D5NZS7|A0A1D5NZS7_CHICK | 1.185694 | up |
| cellular_component | cell part | tr|A0A1D5P047|A0A1D5P047_CHICK | 1.659689 | up |
| cellular_component | cell part | tr|A0A1D5P1D5|A0A1D5P1D5_CHICK | 2.084726 | up |
| cellular_component | cell part | tr|A0A1D5P453|A0A1D5P453_CHICK | 1.553695 | up |
| cellular_component | cell part | tr|A0A1D5P4B1|A0A1D5P4B1_CHICK | 1.156059 | up |
| cellular_component | cell part | tr|A0A1D5P5X4|A0A1D5P5X4_CHICK | 1.559711 | up |
| cellular_component | cell part | tr|A0A1D5P607|A0A1D5P607_CHICK | 1.51904 | up |
| cellular_component | cell part | tr|A0A1D5P6C3|A0A1D5P6C3_CHICK | 2.100747 | up |
| cellular_component | cell part | tr|A0A1D5P6L7|A0A1D5P6L7_CHICK | 1.520092 | up |
| cellular_component | cell part | tr|A0A1D5P6M8|A0A1D5P6M8_CHICK | 1.82889 | up |
| cellular_component | cell part | tr|A0A1D5P6S3|A0A1D5P6S3_CHICK | 1.183172 | up |
| cellular_component | cell part | tr|A0A1D5P6Y6|A0A1D5P6Y6_CHICK | 1.32218 | up |
| cellular_component | cell part | tr|A0A1D5P7C5|A0A1D5P7C5_CHICK | 1.273318 | up |
| cellular_component | cell part | tr|A0A1D5P7E3|A0A1D5P7E3_CHICK | 1.017643 | up |
| cellular_component | cell part | tr|A0A1D5P7Q4|A0A1D5P7Q4_CHICK | 1.066394 | up |
| cellular_component | cell part | tr|A0A1D5P822|A0A1D5P822_CHICK | 1.478805 | up |
| cellular_component | cell part | tr|A0A1D5P8H7|A0A1D5P8H7_CHICK | 1.530513 | up |
| cellular_component | cell part | tr|A0A1D5P8V8|A0A1D5P8V8_CHICK | 1.608897 | up |
| cellular_component | cell part | tr|A0A1D5P997|A0A1D5P997_CHICK | 1.563709 | up |
| cellular_component | cell part | tr|A0A1D5P9G2|A0A1D5P9G2_CHICK | 1.768438 | up |
| cellular_component | cell part | tr|A0A1D5PA08|A0A1D5PA08_CHICK | 1.946165 | up |
| cellular_component | cell part | tr|A0A1D5PAM1|A0A1D5PAM1_CHICK | 2.577291 | up |
| cellular_component | cell part | tr|A0A1D5PC23|A0A1D5PC23_CHICK | 2.344267 | up |
| cellular_component | cell part | tr|A0A1D5PCZ1|A0A1D5PCZ1_CHICK | 1.542638 | up |
| cellular_component | cell part | tr|A0A1D5PDV1|A0A1D5PDV1_CHICK | 1.384898 | up |
| cellular_component | cell part | tr|A0A1D5PEE5|A0A1D5PEE5_CHICK | 1.102471 | up |
| cellular_component | cell part | tr|A0A1D5PF37|A0A1D5PF37_CHICK | 1.238601 | up |
| cellular_component | cell part | tr|A0A1D5PFS2|A0A1D5PFS2_CHICK | 1.463999 | up |
| cellular_component | cell part | tr|A0A1D5PGB1|A0A1D5PGB1_CHICK | 1.43532 | up |
| cellular_component | cell part | tr|A0A1D5PIB1|A0A1D5PIB1_CHICK | 2.10824 | up |
| cellular_component | cell part | tr|A0A1D5PJB9|A0A1D5PJB9_CHICK | 1.548287 | up |
| cellular_component | cell part | tr|A0A1D5PKQ7|A0A1D5PKQ7_CHICK | -1.34664 | down |
| cellular_component | cell part | tr|A0A1D5PM87|A0A1D5PM87_CHICK | 1.108975 | up |
| cellular_component | cell part | tr|A0A1D5PMR8|A0A1D5PMR8_CHICK | 1.213883 | up |
| cellular_component | cell part | tr|A0A1D5PQ66|A0A1D5PQ66_CHICK | 1.664552 | up |
| cellular_component | cell part | tr|A0A1D5PQC1|A0A1D5PQC1_CHICK | 1.667257 | up |
| cellular_component | cell part | tr|A0A1D5PQD0|A0A1D5PQD0_CHICK | 1.314273 | up |
| cellular_component | cell part | tr|A0A1D5PQG6|A0A1D5PQG6_CHICK | 2.091788 | up |
| cellular_component | cell part | tr|A0A1D5PQQ6|A0A1D5PQQ6_CHICK | 1.432587 | up |
| cellular_component | cell part | tr|A0A1D5PR34|A0A1D5PR34_CHICK | 1.370909 | up |
| cellular_component | cell part | tr|A0A1D5PRI4|A0A1D5PRI4_CHICK | 1.28486 | up |
| cellular_component | cell part | tr|A0A1D5PRI6|A0A1D5PRI6_CHICK | 1.580404 | up |
| cellular_component | cell part | tr|A0A1D5PTE8|A0A1D5PTE8_CHICK | 1.960486 | up |
| cellular_component | cell part | tr|A0A1D5PTW3|A0A1D5PTW3_CHICK | 1.808599 | up |
| cellular_component | cell part | tr|A0A1D5PUE2|A0A1D5PUE2_CHICK | 2.043282 | up |
| cellular_component | cell part | tr|A0A1D5PUJ5|A0A1D5PUJ5_CHICK | 1.266991 | up |
| cellular_component | cell part | tr|A0A1D5PWD7|A0A1D5PWD7_CHICK | 1.676687 | up |
| cellular_component | cell part | tr|A0A1D5PXN4|A0A1D5PXN4_CHICK | -1.85723 | down |
| cellular_component | cell part | tr|A0A1D5PYU0|A0A1D5PYU0_CHICK | 1.138573 | up |
| cellular_component | cell part | tr|A0A1D5PZ78|A0A1D5PZ78_CHICK | 1.936124 | up |
| cellular_component | cell part | tr|A0A1D5PZF2|A0A1D5PZF2_CHICK | 1.668934 | up |
| cellular_component | cell part | tr|A0A1D5PZL3|A0A1D5PZL3_CHICK | 1.786305 | up |
| cellular_component | cell part | tr|A0A1D6UPT6|A0A1D6UPT6_CHICK | 1.170381 | up |
| cellular_component | cell part | tr|A0A1L1RIY5|A0A1L1RIY5_CHICK | 751.4932 | up |
| cellular_component | cell part | tr|A0A1L1RL50|A0A1L1RL50_CHICK | 1.173616 | up |
| cellular_component | cell part | tr|A0A1L1RPB1|A0A1L1RPB1_CHICK | 1.864507 | up |
| cellular_component | cell part | tr|A0A1L1RPT9|A0A1L1RPT9_CHICK | 1.353636 | up |
| cellular_component | cell part | tr|A0A1L1RSG1|A0A1L1RSG1_CHICK | 1.889712 | up |
| cellular_component | cell part | tr|A0A1L1RSS5|A0A1L1RSS5_CHICK | 1.143155 | up |
| cellular_component | cell part | tr|A0A1L1RXA3|A0A1L1RXA3_CHICK | 1.394002 | up |
| cellular_component | cell part | tr|A0A3Q2TRS4|A0A3Q2TRS4_CHICK | 1.770416 | up |
| cellular_component | cell part | tr|A0A3Q2TUF8|A0A3Q2TUF8_CHICK | 1.02023 | up |
| cellular_component | cell part | tr|A0A3Q2TUN2|A0A3Q2TUN2_CHICK | 1.036105 | up |
| cellular_component | cell part | tr|A0A3Q2TVU0|A0A3Q2TVU0_CHICK | 1.280793 | up |
| cellular_component | cell part | tr|A0A3Q2TVV0|A0A3Q2TVV0_CHICK | 1.145618 | up |
| cellular_component | cell part | tr|A0A3Q2TY77|A0A3Q2TY77_CHICK | 1.82929 | up |
| cellular_component | cell part | tr|A0A3Q2TYP7|A0A3Q2TYP7_CHICK | 2.159057 | up |
| cellular_component | cell part | tr|A0A3Q2TZM7|A0A3Q2TZM7_CHICK | 1.419267 | up |
| cellular_component | cell part | tr|A0A3Q2U067|A0A3Q2U067_CHICK | 1.508572 | up |
| cellular_component | cell part | tr|A0A3Q2U3C4|A0A3Q2U3C4_CHICK | 1.094483 | up |
| cellular_component | cell part | tr|A0A3Q2U853|A0A3Q2U853_CHICK | 1.530605 | up |
| cellular_component | cell part | tr|A0A3Q2U8I0|A0A3Q2U8I0_CHICK | 1.050068 | up |
| cellular_component | cell part | tr|A0A3Q2UB58|A0A3Q2UB58_CHICK | 2.070855 | up |
| cellular_component | cell part | tr|A0A3Q2UBJ7|A0A3Q2UBJ7_CHICK | 1.945958 | up |
| cellular_component | cell part | tr|A0A3Q2UCG6|A0A3Q2UCG6_CHICK | 1.033099 | up |
| cellular_component | cell part | tr|A0A3Q2UCN1|A0A3Q2UCN1_CHICK | 1.92272 | up |
| cellular_component | cell part | tr|A0A3Q2UCU2|A0A3Q2UCU2_CHICK | 1.557712 | up |
| cellular_component | cell part | tr|A0A3Q2UD87|A0A3Q2UD87_CHICK | 1.205498 | up |
| cellular_component | cell part | tr|A0A3Q2UM13|A0A3Q2UM13_CHICK | 3.560203 | up |
| cellular_component | cell part | tr|A0A3Q2UPF3|A0A3Q2UPF3_CHICK | 1.295517 | up |
| cellular_component | cell part | tr|A0A3Q3AB84|A0A3Q3AB84_CHICK | 1.170717 | up |
| cellular_component | cell part | tr|A0A3Q3ALC8|A0A3Q3ALC8_CHICK | 2.188996 | up |
| cellular_component | cell part | tr|A2T4N8|A2T4N8_CHICK | 1.116095 | up |
| cellular_component | cell part | tr|A8VIF4|A8VIF4_CHICK | 1.024165 | up |
| cellular_component | cell part | tr|D2D3P4|D2D3P4_CHICK | 2.146381 | up |
| cellular_component | cell part | tr|E1BQI0|E1BQI0_CHICK | 1.067708 | up |
| cellular_component | cell part | tr|E1BQU2|E1BQU2_CHICK | 1.629018 | up |
| cellular_component | cell part | tr|E1BQW2|E1BQW2_CHICK | 1.823966 | up |
| cellular_component | cell part | tr|E1BRQ9|E1BRQ9_CHICK | 1.034807 | up |
| cellular_component | cell part | tr|E1BRR6|E1BRR6_CHICK | 1.332531 | up |
| cellular_component | cell part | tr|E1BS31|E1BS31_CHICK | 1.448795 | up |
| cellular_component | cell part | tr|E1BSH7|E1BSH7_CHICK | 1.527514 | up |
| cellular_component | cell part | tr|E1BT44|E1BT44_CHICK | 1.432225 | up |
| cellular_component | cell part | tr|E1BT94|E1BT94_CHICK | 1.49966 | up |
| cellular_component | cell part | tr|E1BTE2|E1BTE2_CHICK | -2.67874 | down |
| cellular_component | cell part | tr|E1BTI7|E1BTI7_CHICK | 1.813027 | up |
| cellular_component | cell part | tr|E1BTS3|E1BTS3_CHICK | 1.090627 | up |
| cellular_component | cell part | tr|E1BTT8|E1BTT8_CHICK | 1.708105 | up |
| cellular_component | cell part | tr|E1BTV1|E1BTV1_CHICK | 2.001762 | up |
| cellular_component | cell part | tr|E1BU27|E1BU27_CHICK | 3.035848 | up |
| cellular_component | cell part | tr|E1BU89|E1BU89_CHICK | 1.303565 | up |
| cellular_component | cell part | tr|E1BUJ1|E1BUJ1_CHICK | 2.709557 | up |
| cellular_component | cell part | tr|E1BUX5|E1BUX5_CHICK | 1.099122 | up |
| cellular_component | cell part | tr|E1BV97|E1BV97_CHICK | 2.036483 | up |
| cellular_component | cell part | tr|E1BVC8|E1BVC8_CHICK | 2.179244 | up |
| cellular_component | cell part | tr|E1BVQ3|E1BVQ3_CHICK | 1.650579 | up |
| cellular_component | cell part | tr|E1BW27|E1BW27_CHICK | 1.938051 | up |
| cellular_component | cell part | tr|E1BWB7|E1BWB7_CHICK | 1.446045 | up |
| cellular_component | cell part | tr|E1BX21|E1BX21_CHICK | 1.42634 | up |
| cellular_component | cell part | tr|E1BX85|E1BX85_CHICK | 2.029343 | up |
| cellular_component | cell part | tr|E1BXC2|E1BXC2_CHICK | 1.767478 | up |
| cellular_component | cell part | tr|E1BXS1|E1BXS1_CHICK | 1.514054 | up |
| cellular_component | cell part | tr|E1BXT9|E1BXT9_CHICK | 1.600608 | up |
| cellular_component | cell part | tr|E1BXY8|E1BXY8_CHICK | 1.106549 | up |
| cellular_component | cell part | tr|E1BY22|E1BY22_CHICK | 1.367672 | up |
| cellular_component | cell part | tr|E1BY40|E1BY40_CHICK | 1.129106 | up |
| cellular_component | cell part | tr|E1BYQ3|E1BYQ3_CHICK | 1.202088 | up |
| cellular_component | cell part | tr|E1BZ79|E1BZ79_CHICK | 2.388844 | up |
| cellular_component | cell part | tr|E1C1T2|E1C1T2_CHICK | 1.594659 | up |
| cellular_component | cell part | tr|E1C1Y5|E1C1Y5_CHICK | 1.135922 | up |
| cellular_component | cell part | tr|E1C229|E1C229_CHICK | 1.473036 | up |
| cellular_component | cell part | tr|E1C2E5|E1C2E5_CHICK | 1.182299 | up |
| cellular_component | cell part | tr|E1C2F2|E1C2F2_CHICK | 1.546061 | up |
| cellular_component | cell part | tr|E1C310|E1C310_CHICK | 1.545191 | up |
| cellular_component | cell part | tr|E1C312|E1C312_CHICK | 1.811403 | up |
| cellular_component | cell part | tr|E1C396|E1C396_CHICK | 1.08258 | up |
| cellular_component | cell part | tr|E1C3U6|E1C3U6_CHICK | 1.144268 | up |
| cellular_component | cell part | tr|E1C593|E1C593_CHICK | 1.922793 | up |
| cellular_component | cell part | tr|E1C688|E1C688_CHICK | 1.775755 | up |
| cellular_component | cell part | tr|E1C6S7|E1C6S7_CHICK | 1.1895 | up |
| cellular_component | cell part | tr|E1C6U2|E1C6U2_CHICK | -1.17102 | down |
| cellular_component | cell part | tr|E1C6V0|E1C6V0_CHICK | 1.577098 | up |
| cellular_component | cell part | tr|E1C7C1|E1C7C1_CHICK | -1.84307 | down |
| cellular_component | cell part | tr|E1C7J8|E1C7J8_CHICK | 1.184879 | up |
| cellular_component | cell part | tr|E1C7P7|E1C7P7_CHICK | 2.341246 | up |
| cellular_component | cell part | tr|E1C8A2|E1C8A2_CHICK | 1.307606 | up |
| cellular_component | cell part | tr|E1C8U1|E1C8U1_CHICK | 1.411305 | up |
| cellular_component | cell part | tr|E1C9H5|E1C9H5_CHICK | 1.195027 | up |
| cellular_component | cell part | tr|E7EC82|E7EC82_CHICK | 1.586027 | up |
| cellular_component | cell part | tr|F1N832|F1N832_CHICK | 1.427435 | up |
| cellular_component | cell part | tr|F1N8T0|F1N8T0_CHICK | 1.514696 | up |
| cellular_component | cell part | tr|F1N8V5|F1N8V5_CHICK | 2.344853 | up |
| cellular_component | cell part | tr|F1N977|F1N977_CHICK | 1.720618 | up |
| cellular_component | cell part | tr|F1N9S3|F1N9S3_CHICK | 1.256683 | up |
| cellular_component | cell part | tr|F1N9U0|F1N9U0_CHICK | 1.038918 | up |
| cellular_component | cell part | tr|F1N9Y3|F1N9Y3_CHICK | 1.33841 | up |
| cellular_component | cell part | tr|F1NBX1|F1NBX1_CHICK | 1.439614 | up |
| cellular_component | cell part | tr|F1NBY5|F1NBY5_CHICK | 1.813459 | up |
| cellular_component | cell part | tr|F1NEA1|F1NEA1_CHICK | 1.395721 | up |
| cellular_component | cell part | tr|F1NEQ3|F1NEQ3_CHICK | 1.139952 | up |
| cellular_component | cell part | tr|F1NF87|F1NF87_CHICK | 1.015859 | up |
| cellular_component | cell part | tr|F1NH23|F1NH23_CHICK | 1.850288 | up |
| cellular_component | cell part | tr|F1NIK3|F1NIK3_CHICK | 1.065249 | up |
| cellular_component | cell part | tr|F1NIQ3|F1NIQ3_CHICK | 1.483149 | up |
| cellular_component | cell part | tr|F1NIY3|F1NIY3_CHICK | 1.568578 | up |
| cellular_component | cell part | tr|F1NJK4|F1NJK4_CHICK | 1.788595 | up |
| cellular_component | cell part | tr|F1NJU5|F1NJU5_CHICK | -1.49981 | down |
| cellular_component | cell part | tr|F1NKY6|F1NKY6_CHICK | 1.171068 | up |
| cellular_component | cell part | tr|F1NMJ9|F1NMJ9_CHICK | -2.4895 | down |
| cellular_component | cell part | tr|F1NMU0|F1NMU0_CHICK | 2.467729 | up |
| cellular_component | cell part | tr|F1NN63|F1NN63_CHICK | 1.72615 | up |
| cellular_component | cell part | tr|F1NPS5|F1NPS5_CHICK | 3.319197 | up |
| cellular_component | cell part | tr|F1NQ61|F1NQ61_CHICK | 1.080306 | up |
| cellular_component | cell part | tr|F1NRZ4|F1NRZ4_CHICK | 1.14911 | up |
| cellular_component | cell part | tr|F1NSD6|F1NSD6_CHICK | 1.689363 | up |
| cellular_component | cell part | tr|F1NSF2|F1NSF2_CHICK | 1.708711 | up |
| cellular_component | cell part | tr|F1NSI7|F1NSI7_CHICK | 1.77407 | up |
| cellular_component | cell part | tr|F1NSQ4|F1NSQ4_CHICK | 1.21442 | up |
| cellular_component | cell part | tr|F1NT58|F1NT58_CHICK | 1.701877 | up |
| cellular_component | cell part | tr|F1NT80|F1NT80_CHICK | 1.404176 | up |
| cellular_component | cell part | tr|F1NTQ2|F1NTQ2_CHICK | 1.236275 | up |
| cellular_component | cell part | tr|F1NUY0|F1NUY0_CHICK | 1.570572 | up |
| cellular_component | cell part | tr|F1NUY9|F1NUY9_CHICK | 1.592258 | up |
| cellular_component | cell part | tr|F1NV66|F1NV66_CHICK | 1.865555 | up |
| cellular_component | cell part | tr|F1NXV6|F1NXV6_CHICK | -4.87705 | down |
| cellular_component | cell part | tr|F1NXZ7|F1NXZ7_CHICK | 1.050248 | up |
| cellular_component | cell part | tr|F1NYG7|F1NYG7_CHICK | 1.537967 | up |
| cellular_component | cell part | tr|F1P151|F1P151_CHICK | 2.603153 | up |
| cellular_component | cell part | tr|F1P204|F1P204_CHICK | 2.129437 | up |
| cellular_component | cell part | tr|F1P278|F1P278_CHICK | 1.88205 | up |
| cellular_component | cell part | tr|F1P2G6|F1P2G6_CHICK | 1.470473 | up |
| cellular_component | cell part | tr|F1P372|F1P372_CHICK | 1.525043 | up |
| cellular_component | cell part | tr|F1P394|F1P394_CHICK | 1.286828 | up |
| cellular_component | cell part | tr|F1P5A3|F1P5A3_CHICK | 2.127136 | up |
| cellular_component | cell part | tr|F1P5B8|F1P5B8_CHICK | 1.715588 | up |
| cellular_component | cell part | tr|F1P5J7|F1P5J7_CHICK | 1.075835 | up |
| cellular_component | cell part | tr|F1P5X6|F1P5X6_CHICK | 1.295882 | up |
| cellular_component | cell part | tr|F6R228|F6R228_CHICK | 1.426396 | up |
| cellular_component | cell part | tr|F6YX81|F6YX81_CHICK | 1.194552 | up |
| cellular_component | cell part | tr|F7BFS9|F7BFS9_CHICK | 1.641443 | up |
| cellular_component | cell part | tr|F7BYG6|F7BYG6_CHICK | 3.125856 | up |
| cellular_component | cell part | tr|H9L0M7|H9L0M7_CHICK | 1.164684 | up |
| cellular_component | cell part | tr|I1SV11|I1SV11_CHICK | 1.313667 | up |
| cellular_component | cell part | tr|M9NCD4|M9NCD4_CHICK | 1.22338 | up |
| cellular_component | cell part | tr|O93466|O93466_CHICK | 1.669881 | up |
| cellular_component | cell part | tr|Q2PUH1|Q2PUH1_CHICK | 1.468226 | up |
| cellular_component | cell part | tr|Q5DWF6|Q5DWF6_CHICK | 1.289716 | up |
| cellular_component | cell part | tr|Q5F351|Q5F351_CHICK | 1.342446 | up |
| cellular_component | cell part | tr|Q5F3R8|Q5F3R8_CHICK | 2.332553 | up |
| cellular_component | cell part | tr|Q5F3X1|Q5F3X1_CHICK | 1.19047 | up |
| cellular_component | cell part | tr|Q5F4B6|Q5F4B6_CHICK | 1.904086 | up |
| cellular_component | cell part | tr|Q5QRU7|Q5QRU7_CHICK | 2.017795 | up |
| cellular_component | cell part | tr|Q5ZHP4|Q5ZHP4_CHICK | 2.101948 | up |
| cellular_component | cell part | tr|Q5ZIL3|Q5ZIL3_CHICK | 1.863958 | up |
| cellular_component | cell part | tr|Q5ZIZ3|Q5ZIZ3_CHICK | 1.248317 | up |
| cellular_component | cell part | tr|Q5ZJ19|Q5ZJ19_CHICK | 2.470646 | up |
| cellular_component | cell part | tr|Q5ZJ96|Q5ZJ96_CHICK | 1.306532 | up |
| cellular_component | cell part | tr|Q5ZJA8|Q5ZJA8_CHICK | 1.093474 | up |
| cellular_component | cell part | tr|Q5ZJQ4|Q5ZJQ4_CHICK | 1.678561 | up |
| cellular_component | cell part | tr|Q5ZJW2|Q5ZJW2_CHICK | 1.71086 | up |
| cellular_component | cell part | tr|Q5ZKI3|Q5ZKI3_CHICK | 1.326993 | up |
| cellular_component | cell part | tr|Q5ZKY5|Q5ZKY5_CHICK | 1.620297 | up |
| cellular_component | cell part | tr|Q5ZL65|Q5ZL65_CHICK | 1.695268 | up |
| cellular_component | cell part | tr|Q5ZLI0|Q5ZLI0_CHICK | 1.097879 | up |
| cellular_component | cell part | tr|Q5ZMA7|Q5ZMA7_CHICK | 1.172026 | up |
| cellular_component | cell part | tr|Q5ZMG8|Q5ZMG8_CHICK | 1.771296 | up |
| cellular_component | cell part | tr|Q5ZMJ6|Q5ZMJ6_CHICK | 1.845871 | up |
| cellular_component | cell part | tr|Q5ZMP2|Q5ZMP2_CHICK | 1.165624 | up |
| cellular_component | cell part | tr|Q9DDD4|Q9DDD4_CHICK | -1.44831 | down |
| cellular_component | cell part | tr|Q9I882|Q9I882_CHICK | 2.21964 | up |
| cellular_component | cell part | tr|Q9I8C8|Q9I8C8_CHICK | 1.2712 | up |
| cellular_component | cell part | tr|Q9PUJ4|Q9PUJ4_CHICK | 1.664957 | up |
| cellular_component | cell part | tr|Q9PVL6|Q9PVL6_CHICK | 1.71949 | up |
| cellular_component | cell part | tr|R4GHH2|R4GHH2_CHICK | 1.778292 | up |
| cellular_component | cell part | tr|R4GHY3|R4GHY3_CHICK | 1.375649 | up |
| cellular_component | cell part | tr|R4GI86|R4GI86_CHICK | 2.078653 | up |
| cellular_component | cell part | tr|R4GJH2|R4GJH2_CHICK | 1.058464 | up |
| cellular_component | cell part | tr|R4GLW9|R4GLW9_CHICK | 1.120506 | up |
| cellular_component | extracellular region | sp|P02197|MYG_CHICK | 2.885059 | up |
| cellular_component | extracellular region | sp|P02659|APOV1_CHICK | -11.5091 | down |
| cellular_component | extracellular region | sp|P04210|LV1_CHICK | -2.82825 | down |
| cellular_component | extracellular region | sp|P19121|ALBU_CHICK | -1.69614 | down |
| cellular_component | extracellular region | sp|P25155|FA10_CHICK | -3.77432 | down |
| cellular_component | extracellular region | sp|P41366|VMO1_CHICK | -5.73258 | down |
| cellular_component | extracellular region | sp|P42292|CD166_CHICK | 1.297212 | up |
| cellular_component | extracellular region | sp|P81475|CFBL_CHICK | -1.75388 | down |
| cellular_component | extracellular region | sp|Q5F3Z3|UB2V2_CHICK | 2.366484 | up |
| cellular_component | extracellular region | sp|Q5ZIF1|APMAP_CHICK | 1.008442 | up |
| cellular_component | extracellular region | sp|Q5ZII9|TFP11_CHICK | 1.172107 | up |
| cellular_component | extracellular region | sp|Q5ZK05|TOLIP_CHICK | 1.088317 | up |
| cellular_component | extracellular region | sp|Q5ZK92|SPAST_CHICK | 1.665787 | up |
| cellular_component | extracellular region | sp|Q5ZKV9|VPS50_CHICK | 1.324318 | up |
| cellular_component | extracellular region | sp|Q5ZMG0|DOPD_CHICK | 2.121364 | up |
| cellular_component | extracellular region | sp|Q804X6|FA9_CHICK | -5.71386 | down |
| cellular_component | extracellular region | tr|A0A140JXP0|A0A140JXP0_CHICK | -4.52589 | down |
| cellular_component | extracellular region | tr|A0A1D5NUV0|A0A1D5NUV0_CHICK | 2.157037 | up |
| cellular_component | extracellular region | tr|A0A1D5NW68|A0A1D5NW68_CHICK | -2.66571 | down |
| cellular_component | extracellular region | tr|A0A1D5P6M8|A0A1D5P6M8_CHICK | 1.82889 | up |
| cellular_component | extracellular region | tr|A0A1D5PC23|A0A1D5PC23_CHICK | 2.344267 | up |
| cellular_component | extracellular region | tr|A0A1D5PHH6|A0A1D5PHH6_CHICK | -2.20613 | down |
| cellular_component | extracellular region | tr|A0A1D5PIT4|A0A1D5PIT4_CHICK | -1.97988 | down |
| cellular_component | extracellular region | tr|A0A1D5PK48|A0A1D5PK48_CHICK | -5.8308 | down |
| cellular_component | extracellular region | tr|A0A1D5PRI6|A0A1D5PRI6_CHICK | 1.580404 | up |
| cellular_component | extracellular region | tr|A0A1D5PU94|A0A1D5PU94_CHICK | -1.37297 | down |
| cellular_component | extracellular region | tr|A0A1D5PWD7|A0A1D5PWD7_CHICK | 1.676687 | up |
| cellular_component | extracellular region | tr|A0A1L1RRQ8|A0A1L1RRQ8_CHICK | -1.76977 | down |
| cellular_component | extracellular region | tr|A0A1L1RWR0|A0A1L1RWR0_CHICK | -2.84844 | down |
| cellular_component | extracellular region | tr|A0A1L1RZ25|A0A1L1RZ25_CHICK | 1.75764 | up |
| cellular_component | extracellular region | tr|A0A3Q2TTT6|A0A3Q2TTT6_CHICK | 1.855872 | up |
| cellular_component | extracellular region | tr|A0A3Q2TUF8|A0A3Q2TUF8_CHICK | 1.02023 | up |
| cellular_component | extracellular region | tr|A0A3Q2TVV0|A0A3Q2TVV0_CHICK | 1.145618 | up |
| cellular_component | extracellular region | tr|A0A3Q2TY77|A0A3Q2TY77_CHICK | 1.82929 | up |
| cellular_component | extracellular region | tr|A0A3Q2TYP7|A0A3Q2TYP7_CHICK | 2.159057 | up |
| cellular_component | extracellular region | tr|A0A3Q2TZM7|A0A3Q2TZM7_CHICK | 1.419267 | up |
| cellular_component | extracellular region | tr|A0A3Q2U3C4|A0A3Q2U3C4_CHICK | 1.094483 | up |
| cellular_component | extracellular region | tr|A0A3Q2U504|A0A3Q2U504_CHICK | -2.35777 | down |
| cellular_component | extracellular region | tr|A0A3Q2UCN1|A0A3Q2UCN1_CHICK | 1.92272 | up |
| cellular_component | extracellular region | tr|A0A3Q2UK74|A0A3Q2UK74_CHICK | 2.254776 | up |
| cellular_component | extracellular region | tr|A0A3Q2UPF3|A0A3Q2UPF3_CHICK | 1.295517 | up |
| cellular_component | extracellular region | tr|A0A3Q3AB84|A0A3Q3AB84_CHICK | 1.170717 | up |
| cellular_component | extracellular region | tr|A0A3Q3AN52|A0A3Q3AN52_CHICK | 1.965147 | up |
| cellular_component | extracellular region | tr|A8VIF4|A8VIF4_CHICK | 1.024165 | up |
| cellular_component | extracellular region | tr|B3VE14|B3VE14_CHICK | -1.63112 | down |
| cellular_component | extracellular region | tr|D2D3P4|D2D3P4_CHICK | 2.146381 | up |
| cellular_component | extracellular region | tr|E1BQC2|E1BQC2_CHICK | -2.52084 | down |
| cellular_component | extracellular region | tr|E1BRR6|E1BRR6_CHICK | 1.332531 | up |
| cellular_component | extracellular region | tr|E1BS31|E1BS31_CHICK | 1.448795 | up |
| cellular_component | extracellular region | tr|E1BS94|E1BS94_CHICK | 1.70074 | up |
| cellular_component | extracellular region | tr|E1BT44|E1BT44_CHICK | 1.432225 | up |
| cellular_component | extracellular region | tr|E1BTE2|E1BTE2_CHICK | -2.67874 | down |
| cellular_component | extracellular region | tr|E1BTI7|E1BTI7_CHICK | 1.813027 | up |
| cellular_component | extracellular region | tr|E1BTV1|E1BTV1_CHICK | 2.001762 | up |
| cellular_component | extracellular region | tr|E1BU89|E1BU89_CHICK | 1.303565 | up |
| cellular_component | extracellular region | tr|E1BW27|E1BW27_CHICK | 1.938051 | up |
| cellular_component | extracellular region | tr|E1BWG7|E1BWG7_CHICK | 2.421508 | up |
| cellular_component | extracellular region | tr|E1BXC2|E1BXC2_CHICK | 1.767478 | up |
| cellular_component | extracellular region | tr|E1BYQ3|E1BYQ3_CHICK | 1.202088 | up |
| cellular_component | extracellular region | tr|E1BZ79|E1BZ79_CHICK | 2.388844 | up |
| cellular_component | extracellular region | tr|E1C396|E1C396_CHICK | 1.08258 | up |
| cellular_component | extracellular region | tr|E1C688|E1C688_CHICK | 1.775755 | up |
| cellular_component | extracellular region | tr|E1C7C1|E1C7C1_CHICK | -1.84307 | down |
| cellular_component | extracellular region | tr|E1C7H6|E1C7H6_CHICK | -1.05537 | down |
| cellular_component | extracellular region | tr|E1C7P7|E1C7P7_CHICK | 2.341246 | up |
| cellular_component | extracellular region | tr|E1C8A2|E1C8A2_CHICK | 1.307606 | up |
| cellular_component | extracellular region | tr|F1NBX1|F1NBX1_CHICK | 1.439614 | up |
| cellular_component | extracellular region | tr|F1NCM5|F1NCM5_CHICK | 2.577712 | up |
| cellular_component | extracellular region | tr|F1NDH2|F1NDH2_CHICK | -1.95135 | down |
| cellular_component | extracellular region | tr|F1NJU5|F1NJU5_CHICK | -1.49981 | down |
| cellular_component | extracellular region | tr|F1NPS5|F1NPS5_CHICK | 3.319197 | up |
| cellular_component | extracellular region | tr|F1NTQ2|F1NTQ2_CHICK | 1.236275 | up |
| cellular_component | extracellular region | tr|F1NUY0|F1NUY0_CHICK | 1.570572 | up |
| cellular_component | extracellular region | tr|F1NXV6|F1NXV6_CHICK | -4.87705 | down |
| cellular_component | extracellular region | tr|F1P099|F1P099_CHICK | 1.399388 | up |
| cellular_component | extracellular region | tr|F1P278|F1P278_CHICK | 1.88205 | up |
| cellular_component | extracellular region | tr|F1P2D2|F1P2D2_CHICK | 1.35416 | up |
| cellular_component | extracellular region | tr|F1P5B8|F1P5B8_CHICK | 1.715588 | up |
| cellular_component | extracellular region | tr|I1SV11|I1SV11_CHICK | 1.313667 | up |
| cellular_component | extracellular region | tr|O93466|O93466_CHICK | 1.669881 | up |
| cellular_component | extracellular region | tr|Q4ADJ6|Q4ADJ6_CHICK | -4.04825 | down |
| cellular_component | extracellular region | tr|Q5F3R8|Q5F3R8_CHICK | 2.332553 | up |
| cellular_component | extracellular region | tr|Q5F3X1|Q5F3X1_CHICK | 1.19047 | up |
| cellular_component | extracellular region | tr|Q5ZIL3|Q5ZIL3_CHICK | 1.863958 | up |
| cellular_component | extracellular region | tr|Q5ZJ19|Q5ZJ19_CHICK | 2.470646 | up |
| cellular_component | extracellular region | tr|Q5ZL65|Q5ZL65_CHICK | 1.695268 | up |
| cellular_component | extracellular region | tr|Q5ZMA7|Q5ZMA7_CHICK | 1.172026 | up |
| cellular_component | extracellular region | tr|Q5ZMP2|Q5ZMP2_CHICK | 1.165624 | up |
| cellular_component | extracellular region | tr|Q9DDD4|Q9DDD4_CHICK | -1.44831 | down |
| cellular_component | extracellular region | tr|Q9PVL6|Q9PVL6_CHICK | 1.71949 | up |
| cellular_component | extracellular region | tr|R4GHY3|R4GHY3_CHICK | 1.375649 | up |
| cellular_component | extracellular region | tr|R4GI86|R4GI86_CHICK | 2.078653 | up |
| cellular_component | extracellular region | tr|R4GMH5|R4GMH5_CHICK | -2.32193 | down |
| cellular_component | extracellular region part | sp|P02197|MYG_CHICK | 2.885059 | up |
| cellular_component | extracellular region part | sp|P02659|APOV1_CHICK | -11.5091 | down |
| cellular_component | extracellular region part | sp|P04210|LV1_CHICK | -2.82825 | down |
| cellular_component | extracellular region part | sp|P19121|ALBU_CHICK | -1.69614 | down |
| cellular_component | extracellular region part | sp|P41366|VMO1_CHICK | -5.73258 | down |
| cellular_component | extracellular region part | sp|P42292|CD166_CHICK | 1.297212 | up |
| cellular_component | extracellular region part | sp|Q5F3Z3|UB2V2_CHICK | 2.366484 | up |
| cellular_component | extracellular region part | sp|Q5ZIF1|APMAP_CHICK | 1.008442 | up |
| cellular_component | extracellular region part | sp|Q5ZII9|TFP11_CHICK | 1.172107 | up |
| cellular_component | extracellular region part | sp|Q5ZK05|TOLIP_CHICK | 1.088317 | up |
| cellular_component | extracellular region part | sp|Q5ZK92|SPAST_CHICK | 1.665787 | up |
| cellular_component | extracellular region part | sp|Q5ZKV9|VPS50_CHICK | 1.324318 | up |
| cellular_component | extracellular region part | sp|Q5ZMG0|DOPD_CHICK | 2.121364 | up |
| cellular_component | extracellular region part | sp|Q804X6|FA9_CHICK | -5.71386 | down |
| cellular_component | extracellular region part | tr|A0A1D5NUV0|A0A1D5NUV0_CHICK | 2.157037 | up |
| cellular_component | extracellular region part | tr|A0A1D5NW68|A0A1D5NW68_CHICK | -2.66571 | down |
| cellular_component | extracellular region part | tr|A0A1D5P6M8|A0A1D5P6M8_CHICK | 1.82889 | up |
| cellular_component | extracellular region part | tr|A0A1D5PC23|A0A1D5PC23_CHICK | 2.344267 | up |
| cellular_component | extracellular region part | tr|A0A1D5PRI6|A0A1D5PRI6_CHICK | 1.580404 | up |
| cellular_component | extracellular region part | tr|A0A1D5PU94|A0A1D5PU94_CHICK | -1.37297 | down |
| cellular_component | extracellular region part | tr|A0A1D5PWD7|A0A1D5PWD7_CHICK | 1.676687 | up |
| cellular_component | extracellular region part | tr|A0A1L1RRQ8|A0A1L1RRQ8_CHICK | -1.76977 | down |
| cellular_component | extracellular region part | tr|A0A1L1RZ25|A0A1L1RZ25_CHICK | 1.75764 | up |
| cellular_component | extracellular region part | tr|A0A3Q2TTT6|A0A3Q2TTT6_CHICK | 1.855872 | up |
| cellular_component | extracellular region part | tr|A0A3Q2TUF8|A0A3Q2TUF8_CHICK | 1.02023 | up |
| cellular_component | extracellular region part | tr|A0A3Q2TVV0|A0A3Q2TVV0_CHICK | 1.145618 | up |
| cellular_component | extracellular region part | tr|A0A3Q2TY77|A0A3Q2TY77_CHICK | 1.82929 | up |
| cellular_component | extracellular region part | tr|A0A3Q2TYP7|A0A3Q2TYP7_CHICK | 2.159057 | up |
| cellular_component | extracellular region part | tr|A0A3Q2TZM7|A0A3Q2TZM7_CHICK | 1.419267 | up |
| cellular_component | extracellular region part | tr|A0A3Q2U3C4|A0A3Q2U3C4_CHICK | 1.094483 | up |
| cellular_component | extracellular region part | tr|A0A3Q2U504|A0A3Q2U504_CHICK | -2.35777 | down |
| cellular_component | extracellular region part | tr|A0A3Q2UCN1|A0A3Q2UCN1_CHICK | 1.92272 | up |
| cellular_component | extracellular region part | tr|A0A3Q2UK74|A0A3Q2UK74_CHICK | 2.254776 | up |
| cellular_component | extracellular region part | tr|A0A3Q2UPF3|A0A3Q2UPF3_CHICK | 1.295517 | up |
| cellular_component | extracellular region part | tr|A0A3Q3AB84|A0A3Q3AB84_CHICK | 1.170717 | up |
| cellular_component | extracellular region part | tr|A0A3Q3AN52|A0A3Q3AN52_CHICK | 1.965147 | up |
| cellular_component | extracellular region part | tr|A8VIF4|A8VIF4_CHICK | 1.024165 | up |
| cellular_component | extracellular region part | tr|B3VE14|B3VE14_CHICK | -1.63112 | down |
| cellular_component | extracellular region part | tr|D2D3P4|D2D3P4_CHICK | 2.146381 | up |
| cellular_component | extracellular region part | tr|E1BQC2|E1BQC2_CHICK | -2.52084 | down |
| cellular_component | extracellular region part | tr|E1BRR6|E1BRR6_CHICK | 1.332531 | up |
| cellular_component | extracellular region part | tr|E1BS31|E1BS31_CHICK | 1.448795 | up |
| cellular_component | extracellular region part | tr|E1BS94|E1BS94_CHICK | 1.70074 | up |
| cellular_component | extracellular region part | tr|E1BT44|E1BT44_CHICK | 1.432225 | up |
| cellular_component | extracellular region part | tr|E1BTE2|E1BTE2_CHICK | -2.67874 | down |
| cellular_component | extracellular region part | tr|E1BTI7|E1BTI7_CHICK | 1.813027 | up |
| cellular_component | extracellular region part | tr|E1BTV1|E1BTV1_CHICK | 2.001762 | up |
| cellular_component | extracellular region part | tr|E1BU89|E1BU89_CHICK | 1.303565 | up |
| cellular_component | extracellular region part | tr|E1BW27|E1BW27_CHICK | 1.938051 | up |
| cellular_component | extracellular region part | tr|E1BWG7|E1BWG7_CHICK | 2.421508 | up |
| cellular_component | extracellular region part | tr|E1BXC2|E1BXC2_CHICK | 1.767478 | up |
| cellular_component | extracellular region part | tr|E1BYQ3|E1BYQ3_CHICK | 1.202088 | up |
| cellular_component | extracellular region part | tr|E1BZ79|E1BZ79_CHICK | 2.388844 | up |
| cellular_component | extracellular region part | tr|E1C396|E1C396_CHICK | 1.08258 | up |
| cellular_component | extracellular region part | tr|E1C688|E1C688_CHICK | 1.775755 | up |
| cellular_component | extracellular region part | tr|E1C7C1|E1C7C1_CHICK | -1.84307 | down |
| cellular_component | extracellular region part | tr|E1C7H6|E1C7H6_CHICK | -1.05537 | down |
| cellular_component | extracellular region part | tr|E1C7P7|E1C7P7_CHICK | 2.341246 | up |
| cellular_component | extracellular region part | tr|E1C8A2|E1C8A2_CHICK | 1.307606 | up |
| cellular_component | extracellular region part | tr|F1NCM5|F1NCM5_CHICK | 2.577712 | up |
| cellular_component | extracellular region part | tr|F1NDH2|F1NDH2_CHICK | -1.95135 | down |
| cellular_component | extracellular region part | tr|F1NJU5|F1NJU5_CHICK | -1.49981 | down |
| cellular_component | extracellular region part | tr|F1NPS5|F1NPS5_CHICK | 3.319197 | up |
| cellular_component | extracellular region part | tr|F1NTQ2|F1NTQ2_CHICK | 1.236275 | up |
| cellular_component | extracellular region part | tr|F1NUY0|F1NUY0_CHICK | 1.570572 | up |
| cellular_component | extracellular region part | tr|F1NXV6|F1NXV6_CHICK | -4.87705 | down |
| cellular_component | extracellular region part | tr|F1P099|F1P099_CHICK | 1.399388 | up |
| cellular_component | extracellular region part | tr|F1P278|F1P278_CHICK | 1.88205 | up |
| cellular_component | extracellular region part | tr|F1P2D2|F1P2D2_CHICK | 1.35416 | up |
| cellular_component | extracellular region part | tr|F1P5B8|F1P5B8_CHICK | 1.715588 | up |
| cellular_component | extracellular region part | tr|I1SV11|I1SV11_CHICK | 1.313667 | up |
| cellular_component | extracellular region part | tr|O93466|O93466_CHICK | 1.669881 | up |
| cellular_component | extracellular region part | tr|Q4ADJ6|Q4ADJ6_CHICK | -4.04825 | down |
| cellular_component | extracellular region part | tr|Q5F3R8|Q5F3R8_CHICK | 2.332553 | up |
| cellular_component | extracellular region part | tr|Q5F3X1|Q5F3X1_CHICK | 1.19047 | up |
| cellular_component | extracellular region part | tr|Q5ZIL3|Q5ZIL3_CHICK | 1.863958 | up |
| cellular_component | extracellular region part | tr|Q5ZJ19|Q5ZJ19_CHICK | 2.470646 | up |
| cellular_component | extracellular region part | tr|Q5ZL65|Q5ZL65_CHICK | 1.695268 | up |
| cellular_component | extracellular region part | tr|Q5ZMA7|Q5ZMA7_CHICK | 1.172026 | up |
| cellular_component | extracellular region part | tr|Q5ZMP2|Q5ZMP2_CHICK | 1.165624 | up |
| cellular_component | extracellular region part | tr|Q9DDD4|Q9DDD4_CHICK | -1.44831 | down |
| cellular_component | extracellular region part | tr|Q9PVL6|Q9PVL6_CHICK | 1.71949 | up |
| cellular_component | extracellular region part | tr|R4GHY3|R4GHY3_CHICK | 1.375649 | up |
| cellular_component | extracellular region part | tr|R4GI86|R4GI86_CHICK | 2.078653 | up |
| cellular_component | macromolecular complex | sp|P00523|SRC_CHICK | 1.599352 | up |
| cellular_component | macromolecular complex | sp|P02659|APOV1_CHICK | -11.5091 | down |
| cellular_component | macromolecular complex | sp|P18936|NU1M_CHICK | 2.034658 | up |
| cellular_component | macromolecular complex | sp|P42292|CD166_CHICK | 1.297212 | up |
| cellular_component | macromolecular complex | sp|P52162|MAX_CHICK | 1.334512 | up |
| cellular_component | macromolecular complex | sp|Q5F3Z3|UB2V2_CHICK | 2.366484 | up |
| cellular_component | macromolecular complex | sp|Q5ZHN3|WIPI2_CHICK | 1.61465 | up |
| cellular_component | macromolecular complex | sp|Q5ZI08|SPT5H_CHICK | 1.235361 | up |
| cellular_component | macromolecular complex | sp|Q5ZII9|TFP11_CHICK | 1.172107 | up |
| cellular_component | macromolecular complex | sp|Q5ZJ08|SYYC_CHICK | 1.23041 | up |
| cellular_component | macromolecular complex | sp|Q5ZJA9|BORC5_CHICK | 1.655713 | up |
| cellular_component | macromolecular complex | sp|Q5ZJB7|CHMP7_CHICK | 1.815716 | up |
| cellular_component | macromolecular complex | sp|Q5ZJH9|DKC1_CHICK | 1.436701 | up |
| cellular_component | macromolecular complex | sp|Q5ZKA3|CWC22_CHICK | 1.734 | up |
| cellular_component | macromolecular complex | sp|Q5ZKV9|VPS50_CHICK | 1.324318 | up |
| cellular_component | macromolecular complex | sp|Q5ZLT0|XPO7_CHICK | 1.469766 | up |
| cellular_component | macromolecular complex | sp|Q5ZLV2|PPIL3_CHICK | 1.29867 | up |
| cellular_component | macromolecular complex | sp|Q5ZML0|BABA2_CHICK | 1.144731 | up |
| cellular_component | macromolecular complex | sp|Q9I8D0|VPP1_CHICK | 1.736096 | up |
| cellular_component | macromolecular complex | sp|Q9IAM7|MRE11_CHICK | 1.337957 | up |
| cellular_component | macromolecular complex | tr|A0A1D5NU82|A0A1D5NU82_CHICK | 1.12312 | up |
| cellular_component | macromolecular complex | tr|A0A1D5P047|A0A1D5P047_CHICK | 1.659689 | up |
| cellular_component | macromolecular complex | tr|A0A1D5P1D5|A0A1D5P1D5_CHICK | 2.084726 | up |
| cellular_component | macromolecular complex | tr|A0A1D5P453|A0A1D5P453_CHICK | 1.553695 | up |
| cellular_component | macromolecular complex | tr|A0A1D5P607|A0A1D5P607_CHICK | 1.51904 | up |
| cellular_component | macromolecular complex | tr|A0A1D5P6L7|A0A1D5P6L7_CHICK | 1.520092 | up |
| cellular_component | macromolecular complex | tr|A0A1D5P7C5|A0A1D5P7C5_CHICK | 1.273318 | up |
| cellular_component | macromolecular complex | tr|A0A1D5P8H7|A0A1D5P8H7_CHICK | 1.530513 | up |
| cellular_component | macromolecular complex | tr|A0A1D5PA08|A0A1D5PA08_CHICK | 1.946165 | up |
| cellular_component | macromolecular complex | tr|A0A1D5PF37|A0A1D5PF37_CHICK | 1.238601 | up |
| cellular_component | macromolecular complex | tr|A0A1D5PFS2|A0A1D5PFS2_CHICK | 1.463999 | up |
| cellular_component | macromolecular complex | tr|A0A1D5PM26|A0A1D5PM26_CHICK | 2.591272 | up |
| cellular_component | macromolecular complex | tr|A0A1D5PMR8|A0A1D5PMR8_CHICK | 1.213883 | up |
| cellular_component | macromolecular complex | tr|A0A1D5PQ66|A0A1D5PQ66_CHICK | 1.664552 | up |
| cellular_component | macromolecular complex | tr|A0A1D5PQD0|A0A1D5PQD0_CHICK | 1.314273 | up |
| cellular_component | macromolecular complex | tr|A0A1D5PRI4|A0A1D5PRI4_CHICK | 1.28486 | up |
| cellular_component | macromolecular complex | tr|A0A1D5PUJ5|A0A1D5PUJ5_CHICK | 1.266991 | up |
| cellular_component | macromolecular complex | tr|A0A1D5PWD7|A0A1D5PWD7_CHICK | 1.676687 | up |
| cellular_component | macromolecular complex | tr|A0A1L1RL50|A0A1L1RL50_CHICK | 1.173616 | up |
| cellular_component | macromolecular complex | tr|A0A1L1RSG1|A0A1L1RSG1_CHICK | 1.889712 | up |
| cellular_component | macromolecular complex | tr|A0A1L1RSS5|A0A1L1RSS5_CHICK | 1.143155 | up |
| cellular_component | macromolecular complex | tr|A0A3Q2TUF8|A0A3Q2TUF8_CHICK | 1.02023 | up |
| cellular_component | macromolecular complex | tr|A0A3Q2TUN2|A0A3Q2TUN2_CHICK | 1.036105 | up |
| cellular_component | macromolecular complex | tr|A0A3Q2TVU0|A0A3Q2TVU0_CHICK | 1.280793 | up |
| cellular_component | macromolecular complex | tr|A0A3Q2UB58|A0A3Q2UB58_CHICK | 2.070855 | up |
| cellular_component | macromolecular complex | tr|A0A3Q2UCN1|A0A3Q2UCN1_CHICK | 1.92272 | up |
| cellular_component | macromolecular complex | tr|A0A3Q2UD87|A0A3Q2UD87_CHICK | 1.205498 | up |
| cellular_component | macromolecular complex | tr|A0A3Q3ALC8|A0A3Q3ALC8_CHICK | 2.188996 | up |
| cellular_component | macromolecular complex | tr|E1BQI0|E1BQI0_CHICK | 1.067708 | up |
| cellular_component | macromolecular complex | tr|E1BT94|E1BT94_CHICK | 1.49966 | up |
| cellular_component | macromolecular complex | tr|E1BUJ1|E1BUJ1_CHICK | 2.709557 | up |
| cellular_component | macromolecular complex | tr|E1BUX5|E1BUX5_CHICK | 1.099122 | up |
| cellular_component | macromolecular complex | tr|E1BV97|E1BV97_CHICK | 2.036483 | up |
| cellular_component | macromolecular complex | tr|E1BW27|E1BW27_CHICK | 1.938051 | up |
| cellular_component | macromolecular complex | tr|E1BX21|E1BX21_CHICK | 1.42634 | up |
| cellular_component | macromolecular complex | tr|E1BXY8|E1BXY8_CHICK | 1.106549 | up |
| cellular_component | macromolecular complex | tr|E1BYQ3|E1BYQ3_CHICK | 1.202088 | up |
| cellular_component | macromolecular complex | tr|E1C1Y5|E1C1Y5_CHICK | 1.135922 | up |
| cellular_component | macromolecular complex | tr|E1C2F2|E1C2F2_CHICK | 1.546061 | up |
| cellular_component | macromolecular complex | tr|E1C396|E1C396_CHICK | 1.08258 | up |
| cellular_component | macromolecular complex | tr|E1C3U6|E1C3U6_CHICK | 1.144268 | up |
| cellular_component | macromolecular complex | tr|E1C593|E1C593_CHICK | 1.922793 | up |
| cellular_component | macromolecular complex | tr|E1C6U2|E1C6U2_CHICK | -1.17102 | down |
| cellular_component | macromolecular complex | tr|E1C7C1|E1C7C1_CHICK | -1.84307 | down |
| cellular_component | macromolecular complex | tr|E1C7P7|E1C7P7_CHICK | 2.341246 | up |
| cellular_component | macromolecular complex | tr|E1C8A2|E1C8A2_CHICK | 1.307606 | up |
| cellular_component | macromolecular complex | tr|E1C8U1|E1C8U1_CHICK | 1.411305 | up |
| cellular_component | macromolecular complex | tr|E7EC82|E7EC82_CHICK | 1.586027 | up |
| cellular_component | macromolecular complex | tr|F1N832|F1N832_CHICK | 1.427435 | up |
| cellular_component | macromolecular complex | tr|F1N9S3|F1N9S3_CHICK | 1.256683 | up |
| cellular_component | macromolecular complex | tr|F1N9U0|F1N9U0_CHICK | 1.038918 | up |
| cellular_component | macromolecular complex | tr|F1NAS0|F1NAS0_CHICK | 1.779766 | up |
| cellular_component | macromolecular complex | tr|F1NF87|F1NF87_CHICK | 1.015859 | up |
| cellular_component | macromolecular complex | tr|F1NJU5|F1NJU5_CHICK | -1.49981 | down |
| cellular_component | macromolecular complex | tr|F1NMU0|F1NMU0_CHICK | 2.467729 | up |
| cellular_component | macromolecular complex | tr|F1NPS5|F1NPS5_CHICK | 3.319197 | up |
| cellular_component | macromolecular complex | tr|F1NV66|F1NV66_CHICK | 1.865555 | up |
| cellular_component | macromolecular complex | tr|F1P151|F1P151_CHICK | 2.603153 | up |
| cellular_component | macromolecular complex | tr|F1P2G6|F1P2G6_CHICK | 1.470473 | up |
| cellular_component | macromolecular complex | tr|F1P5B8|F1P5B8_CHICK | 1.715588 | up |
| cellular_component | macromolecular complex | tr|F1P5X6|F1P5X6_CHICK | 1.295882 | up |
| cellular_component | macromolecular complex | tr|F7BFS9|F7BFS9_CHICK | 1.641443 | up |
| cellular_component | macromolecular complex | tr|O93466|O93466_CHICK | 1.669881 | up |
| cellular_component | macromolecular complex | tr|Q5F351|Q5F351_CHICK | 1.342446 | up |
| cellular_component | macromolecular complex | tr|Q5F4B6|Q5F4B6_CHICK | 1.904086 | up |
| cellular_component | macromolecular complex | tr|Q5ZJ19|Q5ZJ19_CHICK | 2.470646 | up |
| cellular_component | macromolecular complex | tr|Q5ZJ96|Q5ZJ96_CHICK | 1.306532 | up |
| cellular_component | macromolecular complex | tr|Q5ZJA8|Q5ZJA8_CHICK | 1.093474 | up |
| cellular_component | macromolecular complex | tr|Q5ZJW2|Q5ZJW2_CHICK | 1.71086 | up |
| cellular_component | macromolecular complex | tr|Q5ZKI3|Q5ZKI3_CHICK | 1.326993 | up |
| cellular_component | macromolecular complex | tr|Q5ZKY5|Q5ZKY5_CHICK | 1.620297 | up |
| cellular_component | macromolecular complex | tr|Q5ZLI0|Q5ZLI0_CHICK | 1.097879 | up |
| cellular_component | macromolecular complex | tr|Q5ZMP2|Q5ZMP2_CHICK | 1.165624 | up |
| cellular_component | macromolecular complex | tr|Q9I8C8|Q9I8C8_CHICK | 1.2712 | up |
| cellular_component | macromolecular complex | tr|R4GHH2|R4GHH2_CHICK | 1.778292 | up |
| cellular_component | membrane | sp|O93436|STAM2_CHICK | 2.376336 | up |
| cellular_component | membrane | sp|O93602|ATF2_CHICK | 2.071233 | up |
| cellular_component | membrane | sp|P00523|SRC_CHICK | 1.599352 | up |
| cellular_component | membrane | sp|P18936|NU1M_CHICK | 2.034658 | up |
| cellular_component | membrane | sp|P42292|CD166_CHICK | 1.297212 | up |
| cellular_component | membrane | sp|Q5ZHN3|WIPI2_CHICK | 1.61465 | up |
| cellular_component | membrane | sp|Q5ZIF1|APMAP_CHICK | 1.008442 | up |
| cellular_component | membrane | sp|Q5ZIK0|P4K2B_CHICK | 2.036191 | up |
| cellular_component | membrane | sp|Q5ZIK2|PDZ11_CHICK | 1.259402 | up |
| cellular_component | membrane | sp|Q5ZIP4|XRN2_CHICK | 1.286156 | up |
| cellular_component | membrane | sp|Q5ZJ00|EM55_CHICK | 1.664901 | up |
| cellular_component | membrane | sp|Q5ZJA9|BORC5_CHICK | 1.655713 | up |
| cellular_component | membrane | sp|Q5ZJB7|CHMP7_CHICK | 1.815716 | up |
| cellular_component | membrane | sp|Q5ZJW6|KBRS2_CHICK | 1.256362 | up |
| cellular_component | membrane | sp|Q5ZK92|SPAST_CHICK | 1.665787 | up |
| cellular_component | membrane | sp|Q5ZKV9|VPS50_CHICK | 1.324318 | up |
| cellular_component | membrane | sp|Q5ZL36|ZFY27_CHICK | 1.948763 | up |
| cellular_component | membrane | sp|Q5ZLD4|TMM11_CHICK | 1.73923 | up |
| cellular_component | membrane | sp|Q5ZM60|CCPG1_CHICK | 1.624972 | up |
| cellular_component | membrane | sp|Q5ZM83|MIRO2_CHICK | 1.659999 | up |
| cellular_component | membrane | sp|Q98SH2|AT2B1_CHICK | 1.799719 | up |
| cellular_component | membrane | sp|Q9I8D0|VPP1_CHICK | 1.736096 | up |
| cellular_component | membrane | tr|A0A1D5NTT2|A0A1D5NTT2_CHICK | 2.201769 | up |
| cellular_component | membrane | tr|A0A1D5NVT0|A0A1D5NVT0_CHICK | 1.421036 | up |
| cellular_component | membrane | tr|A0A1D5NWJ7|A0A1D5NWJ7_CHICK | 1.195763 | up |
| cellular_component | membrane | tr|A0A1D5NXG4|A0A1D5NXG4_CHICK | 1.245324 | up |
| cellular_component | membrane | tr|A0A1D5NXR2|A0A1D5NXR2_CHICK | 1.37138 | up |
| cellular_component | membrane | tr|A0A1D5NZB4|A0A1D5NZB4_CHICK | 1.671702 | up |
| cellular_component | membrane | tr|A0A1D5P0N6|A0A1D5P0N6_CHICK | 1.727172 | up |
| cellular_component | membrane | tr|A0A1D5P2G7|A0A1D5P2G7_CHICK | 2.766527 | up |
| cellular_component | membrane | tr|A0A1D5P453|A0A1D5P453_CHICK | 1.553695 | up |
| cellular_component | membrane | tr|A0A1D5P4B1|A0A1D5P4B1_CHICK | 1.156059 | up |
| cellular_component | membrane | tr|A0A1D5P5X4|A0A1D5P5X4_CHICK | 1.559711 | up |
| cellular_component | membrane | tr|A0A1D5P6S3|A0A1D5P6S3_CHICK | 1.183172 | up |
| cellular_component | membrane | tr|A0A1D5P756|A0A1D5P756_CHICK | 1.203038 | up |
| cellular_component | membrane | tr|A0A1D5P822|A0A1D5P822_CHICK | 1.478805 | up |
| cellular_component | membrane | tr|A0A1D5P8H7|A0A1D5P8H7_CHICK | 1.530513 | up |
| cellular_component | membrane | tr|A0A1D5P997|A0A1D5P997_CHICK | 1.563709 | up |
| cellular_component | membrane | tr|A0A1D5P9E8|A0A1D5P9E8_CHICK | 1.511813 | up |
| cellular_component | membrane | tr|A0A1D5PCG3|A0A1D5PCG3_CHICK | 1.321695 | up |
| cellular_component | membrane | tr|A0A1D5PEE5|A0A1D5PEE5_CHICK | 1.102471 | up |
| cellular_component | membrane | tr|A0A1D5PFS2|A0A1D5PFS2_CHICK | 1.463999 | up |
| cellular_component | membrane | tr|A0A1D5PGC8|A0A1D5PGC8_CHICK | 1.343827 | up |
| cellular_component | membrane | tr|A0A1D5PL07|A0A1D5PL07_CHICK | 1.796091 | up |
| cellular_component | membrane | tr|A0A1D5PM44|A0A1D5PM44_CHICK | 1.054523 | up |
| cellular_component | membrane | tr|A0A1D5PP61|A0A1D5PP61_CHICK | 1.385936 | up |
| cellular_component | membrane | tr|A0A1D5PQC1|A0A1D5PQC1_CHICK | 1.667257 | up |
| cellular_component | membrane | tr|A0A1D5PQD0|A0A1D5PQD0_CHICK | 1.314273 | up |
| cellular_component | membrane | tr|A0A1D5PQG6|A0A1D5PQG6_CHICK | 2.091788 | up |
| cellular_component | membrane | tr|A0A1D5PQQ6|A0A1D5PQQ6_CHICK | 1.432587 | up |
| cellular_component | membrane | tr|A0A1D5PR34|A0A1D5PR34_CHICK | 1.370909 | up |
| cellular_component | membrane | tr|A0A1D5PRI4|A0A1D5PRI4_CHICK | 1.28486 | up |
| cellular_component | membrane | tr|A0A1D5PTW3|A0A1D5PTW3_CHICK | 1.808599 | up |
| cellular_component | membrane | tr|A0A1D5PUE2|A0A1D5PUE2_CHICK | 2.043282 | up |
| cellular_component | membrane | tr|A0A1D5PWD7|A0A1D5PWD7_CHICK | 1.676687 | up |
| cellular_component | membrane | tr|A0A1D5PYU0|A0A1D5PYU0_CHICK | 1.138573 | up |
| cellular_component | membrane | tr|A0A1D5PZ78|A0A1D5PZ78_CHICK | 1.936124 | up |
| cellular_component | membrane | tr|A0A1L1RL50|A0A1L1RL50_CHICK | 1.173616 | up |
| cellular_component | membrane | tr|A0A1L1RPD1|A0A1L1RPD1_CHICK | 1.396669 | up |
| cellular_component | membrane | tr|A0A1L1RPT9|A0A1L1RPT9_CHICK | 1.353636 | up |
| cellular_component | membrane | tr|A0A1L1RSG1|A0A1L1RSG1_CHICK | 1.889712 | up |
| cellular_component | membrane | tr|A0A1L1RSS5|A0A1L1RSS5_CHICK | 1.143155 | up |
| cellular_component | membrane | tr|A0A1L1RWP3|A0A1L1RWP3_CHICK | -4.54587 | down |
| cellular_component | membrane | tr|A0A1L1S0P1|A0A1L1S0P1_CHICK | -2.55147 | down |
| cellular_component | membrane | tr|A0A3Q2TTT6|A0A3Q2TTT6_CHICK | 1.855872 | up |
| cellular_component | membrane | tr|A0A3Q2TUF8|A0A3Q2TUF8_CHICK | 1.02023 | up |
| cellular_component | membrane | tr|A0A3Q2TUN2|A0A3Q2TUN2_CHICK | 1.036105 | up |
| cellular_component | membrane | tr|A0A3Q2TVV0|A0A3Q2TVV0_CHICK | 1.145618 | up |
| cellular_component | membrane | tr|A0A3Q2TY77|A0A3Q2TY77_CHICK | 1.82929 | up |
| cellular_component | membrane | tr|A0A3Q2TYP7|A0A3Q2TYP7_CHICK | 2.159057 | up |
| cellular_component | membrane | tr|A0A3Q2TZM7|A0A3Q2TZM7_CHICK | 1.419267 | up |
| cellular_component | membrane | tr|A0A3Q2U067|A0A3Q2U067_CHICK | 1.508572 | up |
| cellular_component | membrane | tr|A0A3Q2U3C4|A0A3Q2U3C4_CHICK | 1.094483 | up |
| cellular_component | membrane | tr|A0A3Q2UBH5|A0A3Q2UBH5_CHICK | 1.115044 | up |
| cellular_component | membrane | tr|A0A3Q2UCG6|A0A3Q2UCG6_CHICK | 1.033099 | up |
| cellular_component | membrane | tr|A0A3Q2UCN1|A0A3Q2UCN1_CHICK | 1.92272 | up |
| cellular_component | membrane | tr|A0A3Q2UCU2|A0A3Q2UCU2_CHICK | 1.557712 | up |
| cellular_component | membrane | tr|A0A3Q2UD87|A0A3Q2UD87_CHICK | 1.205498 | up |
| cellular_component | membrane | tr|A0A3Q2UM13|A0A3Q2UM13_CHICK | 3.560203 | up |
| cellular_component | membrane | tr|A0A3Q3AB84|A0A3Q3AB84_CHICK | 1.170717 | up |
| cellular_component | membrane | tr|A0A3Q3AN52|A0A3Q3AN52_CHICK | 1.965147 | up |
| cellular_component | membrane | tr|A0A3Q3AQ10|A0A3Q3AQ10_CHICK | 1.000417 | up |
| cellular_component | membrane | tr|A8VIF4|A8VIF4_CHICK | 1.024165 | up |
| cellular_component | membrane | tr|D2D3P4|D2D3P4_CHICK | 2.146381 | up |
| cellular_component | membrane | tr|E1BQI0|E1BQI0_CHICK | 1.067708 | up |
| cellular_component | membrane | tr|E1BRR6|E1BRR6_CHICK | 1.332531 | up |
| cellular_component | membrane | tr|E1BS31|E1BS31_CHICK | 1.448795 | up |
| cellular_component | membrane | tr|E1BSH7|E1BSH7_CHICK | 1.527514 | up |
| cellular_component | membrane | tr|E1BT94|E1BT94_CHICK | 1.49966 | up |
| cellular_component | membrane | tr|E1BTV1|E1BTV1_CHICK | 2.001762 | up |
| cellular_component | membrane | tr|E1BUX5|E1BUX5_CHICK | 1.099122 | up |
| cellular_component | membrane | tr|E1BVC8|E1BVC8_CHICK | 2.179244 | up |
| cellular_component | membrane | tr|E1BVQ3|E1BVQ3_CHICK | 1.650579 | up |
| cellular_component | membrane | tr|E1BW27|E1BW27_CHICK | 1.938051 | up |
| cellular_component | membrane | tr|E1BWW7|E1BWW7_CHICK | 1.556141 | up |
| cellular_component | membrane | tr|E1BXS1|E1BXS1_CHICK | 1.514054 | up |
| cellular_component | membrane | tr|E1BXT9|E1BXT9_CHICK | 1.600608 | up |
| cellular_component | membrane | tr|E1BXY8|E1BXY8_CHICK | 1.106549 | up |
| cellular_component | membrane | tr|E1BY40|E1BY40_CHICK | 1.129106 | up |
| cellular_component | membrane | tr|E1BYQ3|E1BYQ3_CHICK | 1.202088 | up |
| cellular_component | membrane | tr|E1BZ74|E1BZ74_CHICK | 1.217761 | up |
| cellular_component | membrane | tr|E1C2F2|E1C2F2_CHICK | 1.546061 | up |
| cellular_component | membrane | tr|E1C310|E1C310_CHICK | 1.545191 | up |
| cellular_component | membrane | tr|E1C3Q6|E1C3Q6_CHICK | 1.829017 | up |
| cellular_component | membrane | tr|E1C592|E1C592_CHICK | 1.565005 | up |
| cellular_component | membrane | tr|E1C688|E1C688_CHICK | 1.775755 | up |
| cellular_component | membrane | tr|E1C6U2|E1C6U2_CHICK | -1.17102 | down |
| cellular_component | membrane | tr|E1C7C1|E1C7C1_CHICK | -1.84307 | down |
| cellular_component | membrane | tr|E1C7J8|E1C7J8_CHICK | 1.184879 | up |
| cellular_component | membrane | tr|E1C7P7|E1C7P7_CHICK | 2.341246 | up |
| cellular_component | membrane | tr|E1C8A2|E1C8A2_CHICK | 1.307606 | up |
| cellular_component | membrane | tr|E1C8U1|E1C8U1_CHICK | 1.411305 | up |
| cellular_component | membrane | tr|E7EC82|E7EC82_CHICK | 1.586027 | up |
| cellular_component | membrane | tr|F1N9U0|F1N9U0_CHICK | 1.038918 | up |
| cellular_component | membrane | tr|F1NEA1|F1NEA1_CHICK | 1.395721 | up |
| cellular_component | membrane | tr|F1NEQ3|F1NEQ3_CHICK | 1.139952 | up |
| cellular_component | membrane | tr|F1NH23|F1NH23_CHICK | 1.850288 | up |
| cellular_component | membrane | tr|F1NIK3|F1NIK3_CHICK | 1.065249 | up |
| cellular_component | membrane | tr|F1NJU5|F1NJU5_CHICK | -1.49981 | down |
| cellular_component | membrane | tr|F1NKY6|F1NKY6_CHICK | 1.171068 | up |
| cellular_component | membrane | tr|F1NLV3|F1NLV3_CHICK | 2.001445 | up |
| cellular_component | membrane | tr|F1NMJ9|F1NMJ9_CHICK | -2.4895 | down |
| cellular_component | membrane | tr|F1NMU0|F1NMU0_CHICK | 2.467729 | up |
| cellular_component | membrane | tr|F1NPS5|F1NPS5_CHICK | 3.319197 | up |
| cellular_component | membrane | tr|F1NQ61|F1NQ61_CHICK | 1.080306 | up |
| cellular_component | membrane | tr|F1NSQ4|F1NSQ4_CHICK | 1.21442 | up |
| cellular_component | membrane | tr|F1NT80|F1NT80_CHICK | 1.404176 | up |
| cellular_component | membrane | tr|F1NTQ2|F1NTQ2_CHICK | 1.236275 | up |
| cellular_component | membrane | tr|F1NXV6|F1NXV6_CHICK | -4.87705 | down |
| cellular_component | membrane | tr|F1NXZ7|F1NXZ7_CHICK | 1.050248 | up |
| cellular_component | membrane | tr|F1NYG7|F1NYG7_CHICK | 1.537967 | up |
| cellular_component | membrane | tr|F1P151|F1P151_CHICK | 2.603153 | up |
| cellular_component | membrane | tr|F1P204|F1P204_CHICK | 2.129437 | up |
| cellular_component | membrane | tr|F1P2G6|F1P2G6_CHICK | 1.470473 | up |
| cellular_component | membrane | tr|F1P5B8|F1P5B8_CHICK | 1.715588 | up |
| cellular_component | membrane | tr|F1P5J7|F1P5J7_CHICK | 1.075835 | up |
| cellular_component | membrane | tr|F6YX81|F6YX81_CHICK | 1.194552 | up |
| cellular_component | membrane | tr|F7BYG6|F7BYG6_CHICK | 3.125856 | up |
| cellular_component | membrane | tr|H9L023|H9L023_CHICK | 1.212184 | up |
| cellular_component | membrane | tr|H9L0M7|H9L0M7_CHICK | 1.164684 | up |
| cellular_component | membrane | tr|O93466|O93466_CHICK | 1.669881 | up |
| cellular_component | membrane | tr|Q5F3R8|Q5F3R8_CHICK | 2.332553 | up |
| cellular_component | membrane | tr|Q5F3X1|Q5F3X1_CHICK | 1.19047 | up |
| cellular_component | membrane | tr|Q5F420|Q5F420_CHICK | 1.387816 | up |
| cellular_component | membrane | tr|Q5ZIZ3|Q5ZIZ3_CHICK | 1.248317 | up |
| cellular_component | membrane | tr|Q5ZJ19|Q5ZJ19_CHICK | 2.470646 | up |
| cellular_component | membrane | tr|Q5ZJ96|Q5ZJ96_CHICK | 1.306532 | up |
| cellular_component | membrane | tr|Q5ZJW2|Q5ZJW2_CHICK | 1.71086 | up |
| cellular_component | membrane | tr|Q5ZKI3|Q5ZKI3_CHICK | 1.326993 | up |
| cellular_component | membrane | tr|Q5ZKY5|Q5ZKY5_CHICK | 1.620297 | up |
| cellular_component | membrane | tr|Q5ZL65|Q5ZL65_CHICK | 1.695268 | up |
| cellular_component | membrane | tr|Q5ZLF6|Q5ZLF6_CHICK | 1.17654 | up |
| cellular_component | membrane | tr|Q5ZMA7|Q5ZMA7_CHICK | 1.172026 | up |
| cellular_component | membrane | tr|Q5ZMG8|Q5ZMG8_CHICK | 1.771296 | up |
| cellular_component | membrane | tr|Q5ZMJ6|Q5ZMJ6_CHICK | 1.845871 | up |
| cellular_component | membrane | tr|Q5ZMP2|Q5ZMP2_CHICK | 1.165624 | up |
| cellular_component | membrane | tr|Q9I8C8|Q9I8C8_CHICK | 1.2712 | up |
| cellular_component | membrane | tr|Q9PUJ4|Q9PUJ4_CHICK | 1.664957 | up |
| cellular_component | membrane | tr|Q9PVL6|Q9PVL6_CHICK | 1.71949 | up |
| cellular_component | membrane | tr|R4GJH2|R4GJH2_CHICK | 1.058464 | up |
| cellular_component | membrane | tr|R4GLW9|R4GLW9_CHICK | 1.120506 | up |
| cellular_component | membrane part | sp|P00523|SRC_CHICK | 1.599352 | up |
| cellular_component | membrane part | sp|P18936|NU1M_CHICK | 2.034658 | up |
| cellular_component | membrane part | sp|P42292|CD166_CHICK | 1.297212 | up |
| cellular_component | membrane part | sp|Q5ZHN3|WIPI2_CHICK | 1.61465 | up |
| cellular_component | membrane part | sp|Q5ZIF1|APMAP_CHICK | 1.008442 | up |
| cellular_component | membrane part | sp|Q5ZIK2|PDZ11_CHICK | 1.259402 | up |
| cellular_component | membrane part | sp|Q5ZJA9|BORC5_CHICK | 1.655713 | up |
| cellular_component | membrane part | sp|Q5ZJB7|CHMP7_CHICK | 1.815716 | up |
| cellular_component | membrane part | sp|Q5ZL36|ZFY27_CHICK | 1.948763 | up |
| cellular_component | membrane part | sp|Q5ZLD4|TMM11_CHICK | 1.73923 | up |
| cellular_component | membrane part | sp|Q5ZM60|CCPG1_CHICK | 1.624972 | up |
| cellular_component | membrane part | sp|Q5ZM83|MIRO2_CHICK | 1.659999 | up |
| cellular_component | membrane part | sp|Q98SH2|AT2B1_CHICK | 1.799719 | up |
| cellular_component | membrane part | sp|Q9I8D0|VPP1_CHICK | 1.736096 | up |
| cellular_component | membrane part | tr|A0A1D5NTT2|A0A1D5NTT2_CHICK | 2.201769 | up |
| cellular_component | membrane part | tr|A0A1D5NVT0|A0A1D5NVT0_CHICK | 1.421036 | up |
| cellular_component | membrane part | tr|A0A1D5NWJ7|A0A1D5NWJ7_CHICK | 1.195763 | up |
| cellular_component | membrane part | tr|A0A1D5NXG4|A0A1D5NXG4_CHICK | 1.245324 | up |
| cellular_component | membrane part | tr|A0A1D5NXR2|A0A1D5NXR2_CHICK | 1.37138 | up |
| cellular_component | membrane part | tr|A0A1D5NZB4|A0A1D5NZB4_CHICK | 1.671702 | up |
| cellular_component | membrane part | tr|A0A1D5P0N6|A0A1D5P0N6_CHICK | 1.727172 | up |
| cellular_component | membrane part | tr|A0A1D5P2G7|A0A1D5P2G7_CHICK | 2.766527 | up |
| cellular_component | membrane part | tr|A0A1D5P453|A0A1D5P453_CHICK | 1.553695 | up |
| cellular_component | membrane part | tr|A0A1D5P4B1|A0A1D5P4B1_CHICK | 1.156059 | up |
| cellular_component | membrane part | tr|A0A1D5P5X4|A0A1D5P5X4_CHICK | 1.559711 | up |
| cellular_component | membrane part | tr|A0A1D5P756|A0A1D5P756_CHICK | 1.203038 | up |
| cellular_component | membrane part | tr|A0A1D5P8H7|A0A1D5P8H7_CHICK | 1.530513 | up |
| cellular_component | membrane part | tr|A0A1D5P9E8|A0A1D5P9E8_CHICK | 1.511813 | up |
| cellular_component | membrane part | tr|A0A1D5PCG3|A0A1D5PCG3_CHICK | 1.321695 | up |
| cellular_component | membrane part | tr|A0A1D5PFS2|A0A1D5PFS2_CHICK | 1.463999 | up |
| cellular_component | membrane part | tr|A0A1D5PGC8|A0A1D5PGC8_CHICK | 1.343827 | up |
| cellular_component | membrane part | tr|A0A1D5PL07|A0A1D5PL07_CHICK | 1.796091 | up |
| cellular_component | membrane part | tr|A0A1D5PM44|A0A1D5PM44_CHICK | 1.054523 | up |
| cellular_component | membrane part | tr|A0A1D5PP61|A0A1D5PP61_CHICK | 1.385936 | up |
| cellular_component | membrane part | tr|A0A1D5PR34|A0A1D5PR34_CHICK | 1.370909 | up |
| cellular_component | membrane part | tr|A0A1D5PTW3|A0A1D5PTW3_CHICK | 1.808599 | up |
| cellular_component | membrane part | tr|A0A1D5PUE2|A0A1D5PUE2_CHICK | 2.043282 | up |
| cellular_component | membrane part | tr|A0A1D5PWD7|A0A1D5PWD7_CHICK | 1.676687 | up |
| cellular_component | membrane part | tr|A0A1D5PYU0|A0A1D5PYU0_CHICK | 1.138573 | up |
| cellular_component | membrane part | tr|A0A1L1RL50|A0A1L1RL50_CHICK | 1.173616 | up |
| cellular_component | membrane part | tr|A0A1L1RPD1|A0A1L1RPD1_CHICK | 1.396669 | up |
| cellular_component | membrane part | tr|A0A1L1RSG1|A0A1L1RSG1_CHICK | 1.889712 | up |
| cellular_component | membrane part | tr|A0A1L1RSS5|A0A1L1RSS5_CHICK | 1.143155 | up |
| cellular_component | membrane part | tr|A0A3Q2TUF8|A0A3Q2TUF8_CHICK | 1.02023 | up |
| cellular_component | membrane part | tr|A0A3Q2TUN2|A0A3Q2TUN2_CHICK | 1.036105 | up |
| cellular_component | membrane part | tr|A0A3Q2TVV0|A0A3Q2TVV0_CHICK | 1.145618 | up |
| cellular_component | membrane part | tr|A0A3Q2U067|A0A3Q2U067_CHICK | 1.508572 | up |
| cellular_component | membrane part | tr|A0A3Q2U3C4|A0A3Q2U3C4_CHICK | 1.094483 | up |
| cellular_component | membrane part | tr|A0A3Q2UBH5|A0A3Q2UBH5_CHICK | 1.115044 | up |
| cellular_component | membrane part | tr|A0A3Q2UCG6|A0A3Q2UCG6_CHICK | 1.033099 | up |
| cellular_component | membrane part | tr|A0A3Q2UCN1|A0A3Q2UCN1_CHICK | 1.92272 | up |
| cellular_component | membrane part | tr|A0A3Q2UCU2|A0A3Q2UCU2_CHICK | 1.557712 | up |
| cellular_component | membrane part | tr|A0A3Q2UM13|A0A3Q2UM13_CHICK | 3.560203 | up |
| cellular_component | membrane part | tr|A0A3Q3AB84|A0A3Q3AB84_CHICK | 1.170717 | up |
| cellular_component | membrane part | tr|A0A3Q3AN52|A0A3Q3AN52_CHICK | 1.965147 | up |
| cellular_component | membrane part | tr|A0A3Q3AQ10|A0A3Q3AQ10_CHICK | 1.000417 | up |
| cellular_component | membrane part | tr|E1BQI0|E1BQI0_CHICK | 1.067708 | up |
| cellular_component | membrane part | tr|E1BRR6|E1BRR6_CHICK | 1.332531 | up |
| cellular_component | membrane part | tr|E1BSH7|E1BSH7_CHICK | 1.527514 | up |
| cellular_component | membrane part | tr|E1BT94|E1BT94_CHICK | 1.49966 | up |
| cellular_component | membrane part | tr|E1BTV1|E1BTV1_CHICK | 2.001762 | up |
| cellular_component | membrane part | tr|E1BUX5|E1BUX5_CHICK | 1.099122 | up |
| cellular_component | membrane part | tr|E1BVC8|E1BVC8_CHICK | 2.179244 | up |
| cellular_component | membrane part | tr|E1BW27|E1BW27_CHICK | 1.938051 | up |
| cellular_component | membrane part | tr|E1BWW7|E1BWW7_CHICK | 1.556141 | up |
| cellular_component | membrane part | tr|E1BXS1|E1BXS1_CHICK | 1.514054 | up |
| cellular_component | membrane part | tr|E1BY40|E1BY40_CHICK | 1.129106 | up |
| cellular_component | membrane part | tr|E1BZ74|E1BZ74_CHICK | 1.217761 | up |
| cellular_component | membrane part | tr|E1C310|E1C310_CHICK | 1.545191 | up |
| cellular_component | membrane part | tr|E1C3Q6|E1C3Q6_CHICK | 1.829017 | up |
| cellular_component | membrane part | tr|E1C688|E1C688_CHICK | 1.775755 | up |
| cellular_component | membrane part | tr|E1C6U2|E1C6U2_CHICK | -1.17102 | down |
| cellular_component | membrane part | tr|E1C7C1|E1C7C1_CHICK | -1.84307 | down |
| cellular_component | membrane part | tr|E1C7P7|E1C7P7_CHICK | 2.341246 | up |
| cellular_component | membrane part | tr|E1C8A2|E1C8A2_CHICK | 1.307606 | up |
| cellular_component | membrane part | tr|E7EC82|E7EC82_CHICK | 1.586027 | up |
| cellular_component | membrane part | tr|F1NH23|F1NH23_CHICK | 1.850288 | up |
| cellular_component | membrane part | tr|F1NJU5|F1NJU5_CHICK | -1.49981 | down |
| cellular_component | membrane part | tr|F1NLV3|F1NLV3_CHICK | 2.001445 | up |
| cellular_component | membrane part | tr|F1NMJ9|F1NMJ9_CHICK | -2.4895 | down |
| cellular_component | membrane part | tr|F1NMU0|F1NMU0_CHICK | 2.467729 | up |
| cellular_component | membrane part | tr|F1NPS5|F1NPS5_CHICK | 3.319197 | up |
| cellular_component | membrane part | tr|F1NQ61|F1NQ61_CHICK | 1.080306 | up |
| cellular_component | membrane part | tr|F1NSQ4|F1NSQ4_CHICK | 1.21442 | up |
| cellular_component | membrane part | tr|F1NT80|F1NT80_CHICK | 1.404176 | up |
| cellular_component | membrane part | tr|F1NXV6|F1NXV6_CHICK | -4.87705 | down |
| cellular_component | membrane part | tr|F1NYG7|F1NYG7_CHICK | 1.537967 | up |
| cellular_component | membrane part | tr|F1P151|F1P151_CHICK | 2.603153 | up |
| cellular_component | membrane part | tr|F1P204|F1P204_CHICK | 2.129437 | up |
| cellular_component | membrane part | tr|F1P2G6|F1P2G6_CHICK | 1.470473 | up |
| cellular_component | membrane part | tr|F1P5B8|F1P5B8_CHICK | 1.715588 | up |
| cellular_component | membrane part | tr|F6YX81|F6YX81_CHICK | 1.194552 | up |
| cellular_component | membrane part | tr|H9L023|H9L023_CHICK | 1.212184 | up |
| cellular_component | membrane part | tr|Q5F3R8|Q5F3R8_CHICK | 2.332553 | up |
| cellular_component | membrane part | tr|Q5F420|Q5F420_CHICK | 1.387816 | up |
| cellular_component | membrane part | tr|Q5ZJ19|Q5ZJ19_CHICK | 2.470646 | up |
| cellular_component | membrane part | tr|Q5ZJ96|Q5ZJ96_CHICK | 1.306532 | up |
| cellular_component | membrane part | tr|Q5ZJW2|Q5ZJW2_CHICK | 1.71086 | up |
| cellular_component | membrane part | tr|Q5ZL65|Q5ZL65_CHICK | 1.695268 | up |
| cellular_component | membrane part | tr|Q5ZLF6|Q5ZLF6_CHICK | 1.17654 | up |
| cellular_component | membrane part | tr|Q5ZMA7|Q5ZMA7_CHICK | 1.172026 | up |
| cellular_component | membrane part | tr|Q5ZMJ6|Q5ZMJ6_CHICK | 1.845871 | up |
| cellular_component | membrane part | tr|Q5ZMP2|Q5ZMP2_CHICK | 1.165624 | up |
| cellular_component | membrane part | tr|Q9I8C8|Q9I8C8_CHICK | 1.2712 | up |
| cellular_component | membrane part | tr|Q9PUJ4|Q9PUJ4_CHICK | 1.664957 | up |
| cellular_component | membrane part | tr|Q9PVL6|Q9PVL6_CHICK | 1.71949 | up |
| cellular_component | membrane part | tr|R4GJH2|R4GJH2_CHICK | 1.058464 | up |
| cellular_component | membrane-enclosed lumen | sp|P52162|MAX_CHICK | 1.334512 | up |
| cellular_component | membrane-enclosed lumen | sp|Q05423|FABP7_CHICK | 1.9874 | up |
| cellular_component | membrane-enclosed lumen | sp|Q5F3Z3|UB2V2_CHICK | 2.366484 | up |
| cellular_component | membrane-enclosed lumen | sp|Q5ZI08|SPT5H_CHICK | 1.235361 | up |
| cellular_component | membrane-enclosed lumen | sp|Q5ZI74|DHX30_CHICK | 1.265146 | up |
| cellular_component | membrane-enclosed lumen | sp|Q5ZIB9|ANM7_CHICK | 1.168118 | up |
| cellular_component | membrane-enclosed lumen | sp|Q5ZII9|TFP11_CHICK | 1.172107 | up |
| cellular_component | membrane-enclosed lumen | sp|Q5ZIP4|XRN2_CHICK | 1.286156 | up |
| cellular_component | membrane-enclosed lumen | sp|Q5ZJ08|SYYC_CHICK | 1.23041 | up |
| cellular_component | membrane-enclosed lumen | sp|Q5ZJH9|DKC1_CHICK | 1.436701 | up |
| cellular_component | membrane-enclosed lumen | sp|Q5ZJJ8|UBCP1_CHICK | 2.077308 | up |
| cellular_component | membrane-enclosed lumen | sp|Q5ZK92|SPAST_CHICK | 1.665787 | up |
| cellular_component | membrane-enclosed lumen | sp|Q5ZKA3|CWC22_CHICK | 1.734 | up |
| cellular_component | membrane-enclosed lumen | sp|Q9IAM7|MRE11_CHICK | 1.337957 | up |
| cellular_component | membrane-enclosed lumen | tr|A0A1D5NU82|A0A1D5NU82_CHICK | 1.12312 | up |
| cellular_component | membrane-enclosed lumen | tr|A0A1D5NUD9|A0A1D5NUD9_CHICK | 1.157212 | up |
| cellular_component | membrane-enclosed lumen | tr|A0A1D5NZS7|A0A1D5NZS7_CHICK | 1.185694 | up |
| cellular_component | membrane-enclosed lumen | tr|A0A1D5P607|A0A1D5P607_CHICK | 1.51904 | up |
| cellular_component | membrane-enclosed lumen | tr|A0A1D5P6C3|A0A1D5P6C3_CHICK | 2.100747 | up |
| cellular_component | membrane-enclosed lumen | tr|A0A1D5P7C5|A0A1D5P7C5_CHICK | 1.273318 | up |
| cellular_component | membrane-enclosed lumen | tr|A0A1D5P8H7|A0A1D5P8H7_CHICK | 1.530513 | up |
| cellular_component | membrane-enclosed lumen | tr|A0A1D5PAM1|A0A1D5PAM1_CHICK | 2.577291 | up |
| cellular_component | membrane-enclosed lumen | tr|A0A1D5PEE5|A0A1D5PEE5_CHICK | 1.102471 | up |
| cellular_component | membrane-enclosed lumen | tr|A0A1D5PF37|A0A1D5PF37_CHICK | 1.238601 | up |
| cellular_component | membrane-enclosed lumen | tr|A0A1D5PKQ7|A0A1D5PKQ7_CHICK | -1.34664 | down |
| cellular_component | membrane-enclosed lumen | tr|A0A1D5PM87|A0A1D5PM87_CHICK | 1.108975 | up |
| cellular_component | membrane-enclosed lumen | tr|A0A1D5PMR8|A0A1D5PMR8_CHICK | 1.213883 | up |
| cellular_component | membrane-enclosed lumen | tr|A0A1D5PQD0|A0A1D5PQD0_CHICK | 1.314273 | up |
| cellular_component | membrane-enclosed lumen | tr|A0A1D5PRI4|A0A1D5PRI4_CHICK | 1.28486 | up |
| cellular_component | membrane-enclosed lumen | tr|A0A1D5PRI6|A0A1D5PRI6_CHICK | 1.580404 | up |
| cellular_component | membrane-enclosed lumen | tr|A0A1L1RPT9|A0A1L1RPT9_CHICK | 1.353636 | up |
| cellular_component | membrane-enclosed lumen | tr|A0A1L1RSS5|A0A1L1RSS5_CHICK | 1.143155 | up |
| cellular_component | membrane-enclosed lumen | tr|A0A3Q2TVU0|A0A3Q2TVU0_CHICK | 1.280793 | up |
| cellular_component | membrane-enclosed lumen | tr|A0A3Q2UB58|A0A3Q2UB58_CHICK | 2.070855 | up |
| cellular_component | membrane-enclosed lumen | tr|A0A3Q2UBJ7|A0A3Q2UBJ7_CHICK | 1.945958 | up |
| cellular_component | membrane-enclosed lumen | tr|A0A3Q2UCN1|A0A3Q2UCN1_CHICK | 1.92272 | up |
| cellular_component | membrane-enclosed lumen | tr|A0A3Q2UD87|A0A3Q2UD87_CHICK | 1.205498 | up |
| cellular_component | membrane-enclosed lumen | tr|A0A3Q3ALC8|A0A3Q3ALC8_CHICK | 2.188996 | up |
| cellular_component | membrane-enclosed lumen | tr|E1BRR6|E1BRR6_CHICK | 1.332531 | up |
| cellular_component | membrane-enclosed lumen | tr|E1BT94|E1BT94_CHICK | 1.49966 | up |
| cellular_component | membrane-enclosed lumen | tr|E1BUJ1|E1BUJ1_CHICK | 2.709557 | up |
| cellular_component | membrane-enclosed lumen | tr|E1BV97|E1BV97_CHICK | 2.036483 | up |
| cellular_component | membrane-enclosed lumen | tr|E1BX21|E1BX21_CHICK | 1.42634 | up |
| cellular_component | membrane-enclosed lumen | tr|E1BXY8|E1BXY8_CHICK | 1.106549 | up |
| cellular_component | membrane-enclosed lumen | tr|E1C1Y5|E1C1Y5_CHICK | 1.135922 | up |
| cellular_component | membrane-enclosed lumen | tr|E1C2F2|E1C2F2_CHICK | 1.546061 | up |
| cellular_component | membrane-enclosed lumen | tr|E1C3U6|E1C3U6_CHICK | 1.144268 | up |
| cellular_component | membrane-enclosed lumen | tr|E1C593|E1C593_CHICK | 1.922793 | up |
| cellular_component | membrane-enclosed lumen | tr|E1C688|E1C688_CHICK | 1.775755 | up |
| cellular_component | membrane-enclosed lumen | tr|E1C6S7|E1C6S7_CHICK | 1.1895 | up |
| cellular_component | membrane-enclosed lumen | tr|F1N832|F1N832_CHICK | 1.427435 | up |
| cellular_component | membrane-enclosed lumen | tr|F1N9U0|F1N9U0_CHICK | 1.038918 | up |
| cellular_component | membrane-enclosed lumen | tr|F1NEA1|F1NEA1_CHICK | 1.395721 | up |
| cellular_component | membrane-enclosed lumen | tr|F1NEQ3|F1NEQ3_CHICK | 1.139952 | up |
| cellular_component | membrane-enclosed lumen | tr|F1NIK3|F1NIK3_CHICK | 1.065249 | up |
| cellular_component | membrane-enclosed lumen | tr|F1NKY6|F1NKY6_CHICK | 1.171068 | up |
| cellular_component | membrane-enclosed lumen | tr|F1NPS5|F1NPS5_CHICK | 3.319197 | up |
| cellular_component | membrane-enclosed lumen | tr|F1NRZ4|F1NRZ4_CHICK | 1.14911 | up |
| cellular_component | membrane-enclosed lumen | tr|F1NXZ7|F1NXZ7_CHICK | 1.050248 | up |
| cellular_component | membrane-enclosed lumen | tr|F1P394|F1P394_CHICK | 1.286828 | up |
| cellular_component | membrane-enclosed lumen | tr|F1P5J7|F1P5J7_CHICK | 1.075835 | up |
| cellular_component | membrane-enclosed lumen | tr|F6R228|F6R228_CHICK | 1.426396 | up |
| cellular_component | membrane-enclosed lumen | tr|F7BFS9|F7BFS9_CHICK | 1.641443 | up |
| cellular_component | membrane-enclosed lumen | tr|Q2PUH1|Q2PUH1_CHICK | 1.468226 | up |
| cellular_component | membrane-enclosed lumen | tr|Q5DWF6|Q5DWF6_CHICK | 1.289716 | up |
| cellular_component | membrane-enclosed lumen | tr|Q5F4B6|Q5F4B6_CHICK | 1.904086 | up |
| cellular_component | membrane-enclosed lumen | tr|Q5QRU7|Q5QRU7_CHICK | 2.017795 | up |
| cellular_component | membrane-enclosed lumen | tr|Q5ZJA8|Q5ZJA8_CHICK | 1.093474 | up |
| cellular_component | membrane-enclosed lumen | tr|Q5ZKY5|Q5ZKY5_CHICK | 1.620297 | up |
| cellular_component | membrane-enclosed lumen | tr|Q5ZLI0|Q5ZLI0_CHICK | 1.097879 | up |
| cellular_component | membrane-enclosed lumen | tr|Q9DDD4|Q9DDD4_CHICK | -1.44831 | down |
| cellular_component | nucleoid | sp|Q5ZI74|DHX30_CHICK | 1.265146 | up |
| cellular_component | organelle | sp|O42395|CNBP_CHICK | 1.365125 | up |
| cellular_component | organelle | sp|O93436|STAM2_CHICK | 2.376336 | up |
| cellular_component | organelle | sp|O93602|ATF2_CHICK | 2.071233 | up |
| cellular_component | organelle | sp|P00523|SRC_CHICK | 1.599352 | up |
| cellular_component | organelle | sp|P02197|MYG_CHICK | 2.885059 | up |
| cellular_component | organelle | sp|P07090|CALB2_CHICK | 1.954973 | up |
| cellular_component | organelle | sp|P17923|NFIA_CHICK | 1.346459 | up |
| cellular_component | organelle | sp|P18936|NU1M_CHICK | 2.034658 | up |
| cellular_component | organelle | sp|P41366|VMO1_CHICK | -5.73258 | down |
| cellular_component | organelle | sp|P42292|CD166_CHICK | 1.297212 | up |
| cellular_component | organelle | sp|P52162|MAX_CHICK | 1.334512 | up |
| cellular_component | organelle | sp|Q05423|FABP7_CHICK | 1.9874 | up |
| cellular_component | organelle | sp|Q5F3K4|WDR48_CHICK | 1.71564 | up |
| cellular_component | organelle | sp|Q5F3Z3|UB2V2_CHICK | 2.366484 | up |
| cellular_component | organelle | sp|Q5ZHN3|WIPI2_CHICK | 1.61465 | up |
| cellular_component | organelle | sp|Q5ZHT1|ACD11_CHICK | 1.653324 | up |
| cellular_component | organelle | sp|Q5ZI08|SPT5H_CHICK | 1.235361 | up |
| cellular_component | organelle | sp|Q5ZI74|DHX30_CHICK | 1.265146 | up |
| cellular_component | organelle | sp|Q5ZIB9|ANM7_CHICK | 1.168118 | up |
| cellular_component | organelle | sp|Q5ZID0|NMRL1_CHICK | 2.429369 | up |
| cellular_component | organelle | sp|Q5ZIF1|APMAP_CHICK | 1.008442 | up |
| cellular_component | organelle | sp|Q5ZII9|TFP11_CHICK | 1.172107 | up |
| cellular_component | organelle | sp|Q5ZIK0|P4K2B_CHICK | 2.036191 | up |
| cellular_component | organelle | sp|Q5ZIP4|XRN2_CHICK | 1.286156 | up |
| cellular_component | organelle | sp|Q5ZJ00|EM55_CHICK | 1.664901 | up |
| cellular_component | organelle | sp|Q5ZJ08|SYYC_CHICK | 1.23041 | up |
| cellular_component | organelle | sp|Q5ZJA9|BORC5_CHICK | 1.655713 | up |
| cellular_component | organelle | sp|Q5ZJB7|CHMP7_CHICK | 1.815716 | up |
| cellular_component | organelle | sp|Q5ZJH9|DKC1_CHICK | 1.436701 | up |
| cellular_component | organelle | sp|Q5ZJJ8|UBCP1_CHICK | 2.077308 | up |
| cellular_component | organelle | sp|Q5ZK05|TOLIP_CHICK | 1.088317 | up |
| cellular_component | organelle | sp|Q5ZK92|SPAST_CHICK | 1.665787 | up |
| cellular_component | organelle | sp|Q5ZKA3|CWC22_CHICK | 1.734 | up |
| cellular_component | organelle | sp|Q5ZKV4|NUBP2_CHICK | 1.740378 | up |
| cellular_component | organelle | sp|Q5ZKV9|VPS50_CHICK | 1.324318 | up |
| cellular_component | organelle | sp|Q5ZL36|ZFY27_CHICK | 1.948763 | up |
| cellular_component | organelle | sp|Q5ZLD4|TMM11_CHICK | 1.73923 | up |
| cellular_component | organelle | sp|Q5ZLT0|XPO7_CHICK | 1.469766 | up |
| cellular_component | organelle | sp|Q5ZLV2|PPIL3_CHICK | 1.29867 | up |
| cellular_component | organelle | sp|Q5ZM83|MIRO2_CHICK | 1.659999 | up |
| cellular_component | organelle | sp|Q5ZML0|BABA2_CHICK | 1.144731 | up |
| cellular_component | organelle | sp|Q90733|COT2_CHICK | 1.595685 | up |
| cellular_component | organelle | sp|Q9I8D0|VPP1_CHICK | 1.736096 | up |
| cellular_component | organelle | sp|Q9IAM7|MRE11_CHICK | 1.337957 | up |
| cellular_component | organelle | tr|A0A1D5NU82|A0A1D5NU82_CHICK | 1.12312 | up |
| cellular_component | organelle | tr|A0A1D5NUD9|A0A1D5NUD9_CHICK | 1.157212 | up |
| cellular_component | organelle | tr|A0A1D5NUV0|A0A1D5NUV0_CHICK | 2.157037 | up |
| cellular_component | organelle | tr|A0A1D5NWJ7|A0A1D5NWJ7_CHICK | 1.195763 | up |
| cellular_component | organelle | tr|A0A1D5NXR2|A0A1D5NXR2_CHICK | 1.37138 | up |
| cellular_component | organelle | tr|A0A1D5NXS8|A0A1D5NXS8_CHICK | 1.203865 | up |
| cellular_component | organelle | tr|A0A1D5NZB4|A0A1D5NZB4_CHICK | 1.671702 | up |
| cellular_component | organelle | tr|A0A1D5NZS7|A0A1D5NZS7_CHICK | 1.185694 | up |
| cellular_component | organelle | tr|A0A1D5P047|A0A1D5P047_CHICK | 1.659689 | up |
| cellular_component | organelle | tr|A0A1D5P453|A0A1D5P453_CHICK | 1.553695 | up |
| cellular_component | organelle | tr|A0A1D5P4B1|A0A1D5P4B1_CHICK | 1.156059 | up |
| cellular_component | organelle | tr|A0A1D5P607|A0A1D5P607_CHICK | 1.51904 | up |
| cellular_component | organelle | tr|A0A1D5P6C3|A0A1D5P6C3_CHICK | 2.100747 | up |
| cellular_component | organelle | tr|A0A1D5P6M8|A0A1D5P6M8_CHICK | 1.82889 | up |
| cellular_component | organelle | tr|A0A1D5P6S3|A0A1D5P6S3_CHICK | 1.183172 | up |
| cellular_component | organelle | tr|A0A1D5P7C5|A0A1D5P7C5_CHICK | 1.273318 | up |
| cellular_component | organelle | tr|A0A1D5P7E3|A0A1D5P7E3_CHICK | 1.017643 | up |
| cellular_component | organelle | tr|A0A1D5P7Q4|A0A1D5P7Q4_CHICK | 1.066394 | up |
| cellular_component | organelle | tr|A0A1D5P822|A0A1D5P822_CHICK | 1.478805 | up |
| cellular_component | organelle | tr|A0A1D5P8H7|A0A1D5P8H7_CHICK | 1.530513 | up |
| cellular_component | organelle | tr|A0A1D5P8V8|A0A1D5P8V8_CHICK | 1.608897 | up |
| cellular_component | organelle | tr|A0A1D5P997|A0A1D5P997_CHICK | 1.563709 | up |
| cellular_component | organelle | tr|A0A1D5PAM1|A0A1D5PAM1_CHICK | 2.577291 | up |
| cellular_component | organelle | tr|A0A1D5PC23|A0A1D5PC23_CHICK | 2.344267 | up |
| cellular_component | organelle | tr|A0A1D5PDV1|A0A1D5PDV1_CHICK | 1.384898 | up |
| cellular_component | organelle | tr|A0A1D5PEE5|A0A1D5PEE5_CHICK | 1.102471 | up |
| cellular_component | organelle | tr|A0A1D5PF37|A0A1D5PF37_CHICK | 1.238601 | up |
| cellular_component | organelle | tr|A0A1D5PFS2|A0A1D5PFS2_CHICK | 1.463999 | up |
| cellular_component | organelle | tr|A0A1D5PIB1|A0A1D5PIB1_CHICK | 2.10824 | up |
| cellular_component | organelle | tr|A0A1D5PKQ7|A0A1D5PKQ7_CHICK | -1.34664 | down |
| cellular_component | organelle | tr|A0A1D5PM87|A0A1D5PM87_CHICK | 1.108975 | up |
| cellular_component | organelle | tr|A0A1D5PMR8|A0A1D5PMR8_CHICK | 1.213883 | up |
| cellular_component | organelle | tr|A0A1D5PQ66|A0A1D5PQ66_CHICK | 1.664552 | up |
| cellular_component | organelle | tr|A0A1D5PQC1|A0A1D5PQC1_CHICK | 1.667257 | up |
| cellular_component | organelle | tr|A0A1D5PQD0|A0A1D5PQD0_CHICK | 1.314273 | up |
| cellular_component | organelle | tr|A0A1D5PQG6|A0A1D5PQG6_CHICK | 2.091788 | up |
| cellular_component | organelle | tr|A0A1D5PQQ6|A0A1D5PQQ6_CHICK | 1.432587 | up |
| cellular_component | organelle | tr|A0A1D5PR34|A0A1D5PR34_CHICK | 1.370909 | up |
| cellular_component | organelle | tr|A0A1D5PRI4|A0A1D5PRI4_CHICK | 1.28486 | up |
| cellular_component | organelle | tr|A0A1D5PRI6|A0A1D5PRI6_CHICK | 1.580404 | up |
| cellular_component | organelle | tr|A0A1D5PTW3|A0A1D5PTW3_CHICK | 1.808599 | up |
| cellular_component | organelle | tr|A0A1D5PUE2|A0A1D5PUE2_CHICK | 2.043282 | up |
| cellular_component | organelle | tr|A0A1D5PUJ5|A0A1D5PUJ5_CHICK | 1.266991 | up |
| cellular_component | organelle | tr|A0A1D5PWD7|A0A1D5PWD7_CHICK | 1.676687 | up |
| cellular_component | organelle | tr|A0A1D5PXN4|A0A1D5PXN4_CHICK | -1.85723 | down |
| cellular_component | organelle | tr|A0A1D5PYU0|A0A1D5PYU0_CHICK | 1.138573 | up |
| cellular_component | organelle | tr|A0A1D6UPT6|A0A1D6UPT6_CHICK | 1.170381 | up |
| cellular_component | organelle | tr|A0A1L1RL50|A0A1L1RL50_CHICK | 1.173616 | up |
| cellular_component | organelle | tr|A0A1L1RPB1|A0A1L1RPB1_CHICK | 1.864507 | up |
| cellular_component | organelle | tr|A0A1L1RPT9|A0A1L1RPT9_CHICK | 1.353636 | up |
| cellular_component | organelle | tr|A0A1L1RSG1|A0A1L1RSG1_CHICK | 1.889712 | up |
| cellular_component | organelle | tr|A0A1L1RSS5|A0A1L1RSS5_CHICK | 1.143155 | up |
| cellular_component | organelle | tr|A0A1L1RZ25|A0A1L1RZ25_CHICK | 1.75764 | up |
| cellular_component | organelle | tr|A0A3Q2TTT6|A0A3Q2TTT6_CHICK | 1.855872 | up |
| cellular_component | organelle | tr|A0A3Q2TUF8|A0A3Q2TUF8_CHICK | 1.02023 | up |
| cellular_component | organelle | tr|A0A3Q2TUN2|A0A3Q2TUN2_CHICK | 1.036105 | up |
| cellular_component | organelle | tr|A0A3Q2TVU0|A0A3Q2TVU0_CHICK | 1.280793 | up |
| cellular_component | organelle | tr|A0A3Q2TVV0|A0A3Q2TVV0_CHICK | 1.145618 | up |
| cellular_component | organelle | tr|A0A3Q2TY77|A0A3Q2TY77_CHICK | 1.82929 | up |
| cellular_component | organelle | tr|A0A3Q2TYP7|A0A3Q2TYP7_CHICK | 2.159057 | up |
| cellular_component | organelle | tr|A0A3Q2TZM7|A0A3Q2TZM7_CHICK | 1.419267 | up |
| cellular_component | organelle | tr|A0A3Q2U067|A0A3Q2U067_CHICK | 1.508572 | up |
| cellular_component | organelle | tr|A0A3Q2U3C4|A0A3Q2U3C4_CHICK | 1.094483 | up |
| cellular_component | organelle | tr|A0A3Q2U504|A0A3Q2U504_CHICK | -2.35777 | down |
| cellular_component | organelle | tr|A0A3Q2U853|A0A3Q2U853_CHICK | 1.530605 | up |
| cellular_component | organelle | tr|A0A3Q2U8I0|A0A3Q2U8I0_CHICK | 1.050068 | up |
| cellular_component | organelle | tr|A0A3Q2UB58|A0A3Q2UB58_CHICK | 2.070855 | up |
| cellular_component | organelle | tr|A0A3Q2UBJ7|A0A3Q2UBJ7_CHICK | 1.945958 | up |
| cellular_component | organelle | tr|A0A3Q2UCG6|A0A3Q2UCG6_CHICK | 1.033099 | up |
| cellular_component | organelle | tr|A0A3Q2UCN1|A0A3Q2UCN1_CHICK | 1.92272 | up |
| cellular_component | organelle | tr|A0A3Q2UCU2|A0A3Q2UCU2_CHICK | 1.557712 | up |
| cellular_component | organelle | tr|A0A3Q2UD87|A0A3Q2UD87_CHICK | 1.205498 | up |
| cellular_component | organelle | tr|A0A3Q2UPF3|A0A3Q2UPF3_CHICK | 1.295517 | up |
| cellular_component | organelle | tr|A0A3Q3AB84|A0A3Q3AB84_CHICK | 1.170717 | up |
| cellular_component | organelle | tr|A0A3Q3ALC8|A0A3Q3ALC8_CHICK | 2.188996 | up |
| cellular_component | organelle | tr|A0A3Q3AN52|A0A3Q3AN52_CHICK | 1.965147 | up |
| cellular_component | organelle | tr|A8VIF4|A8VIF4_CHICK | 1.024165 | up |
| cellular_component | organelle | tr|B3VE14|B3VE14_CHICK | -1.63112 | down |
| cellular_component | organelle | tr|D2D3P4|D2D3P4_CHICK | 2.146381 | up |
| cellular_component | organelle | tr|E1BQI0|E1BQI0_CHICK | 1.067708 | up |
| cellular_component | organelle | tr|E1BQW2|E1BQW2_CHICK | 1.823966 | up |
| cellular_component | organelle | tr|E1BRQ9|E1BRQ9_CHICK | 1.034807 | up |
| cellular_component | organelle | tr|E1BRR6|E1BRR6_CHICK | 1.332531 | up |
| cellular_component | organelle | tr|E1BS31|E1BS31_CHICK | 1.448795 | up |
| cellular_component | organelle | tr|E1BS94|E1BS94_CHICK | 1.70074 | up |
| cellular_component | organelle | tr|E1BSH7|E1BSH7_CHICK | 1.527514 | up |
| cellular_component | organelle | tr|E1BT44|E1BT44_CHICK | 1.432225 | up |
| cellular_component | organelle | tr|E1BT94|E1BT94_CHICK | 1.49966 | up |
| cellular_component | organelle | tr|E1BTE2|E1BTE2_CHICK | -2.67874 | down |
| cellular_component | organelle | tr|E1BTI7|E1BTI7_CHICK | 1.813027 | up |
| cellular_component | organelle | tr|E1BTS3|E1BTS3_CHICK | 1.090627 | up |
| cellular_component | organelle | tr|E1BTV1|E1BTV1_CHICK | 2.001762 | up |
| cellular_component | organelle | tr|E1BU89|E1BU89_CHICK | 1.303565 | up |
| cellular_component | organelle | tr|E1BUJ1|E1BUJ1_CHICK | 2.709557 | up |
| cellular_component | organelle | tr|E1BUX5|E1BUX5_CHICK | 1.099122 | up |
| cellular_component | organelle | tr|E1BV97|E1BV97_CHICK | 2.036483 | up |
| cellular_component | organelle | tr|E1BVC8|E1BVC8_CHICK | 2.179244 | up |
| cellular_component | organelle | tr|E1BVQ3|E1BVQ3_CHICK | 1.650579 | up |
| cellular_component | organelle | tr|E1BW27|E1BW27_CHICK | 1.938051 | up |
| cellular_component | organelle | tr|E1BWB7|E1BWB7_CHICK | 1.446045 | up |
| cellular_component | organelle | tr|E1BWG7|E1BWG7_CHICK | 2.421508 | up |
| cellular_component | organelle | tr|E1BX21|E1BX21_CHICK | 1.42634 | up |
| cellular_component | organelle | tr|E1BXC2|E1BXC2_CHICK | 1.767478 | up |
| cellular_component | organelle | tr|E1BXS1|E1BXS1_CHICK | 1.514054 | up |
| cellular_component | organelle | tr|E1BXT9|E1BXT9_CHICK | 1.600608 | up |
| cellular_component | organelle | tr|E1BXY8|E1BXY8_CHICK | 1.106549 | up |
| cellular_component | organelle | tr|E1BY22|E1BY22_CHICK | 1.367672 | up |
| cellular_component | organelle | tr|E1BY40|E1BY40_CHICK | 1.129106 | up |
| cellular_component | organelle | tr|E1BYQ3|E1BYQ3_CHICK | 1.202088 | up |
| cellular_component | organelle | tr|E1BZ79|E1BZ79_CHICK | 2.388844 | up |
| cellular_component | organelle | tr|E1C1Y5|E1C1Y5_CHICK | 1.135922 | up |
| cellular_component | organelle | tr|E1C229|E1C229_CHICK | 1.473036 | up |
| cellular_component | organelle | tr|E1C2E5|E1C2E5_CHICK | 1.182299 | up |
| cellular_component | organelle | tr|E1C2F2|E1C2F2_CHICK | 1.546061 | up |
| cellular_component | organelle | tr|E1C310|E1C310_CHICK | 1.545191 | up |
| cellular_component | organelle | tr|E1C312|E1C312_CHICK | 1.811403 | up |
| cellular_component | organelle | tr|E1C396|E1C396_CHICK | 1.08258 | up |
| cellular_component | organelle | tr|E1C3U6|E1C3U6_CHICK | 1.144268 | up |
| cellular_component | organelle | tr|E1C593|E1C593_CHICK | 1.922793 | up |
| cellular_component | organelle | tr|E1C688|E1C688_CHICK | 1.775755 | up |
| cellular_component | organelle | tr|E1C6S7|E1C6S7_CHICK | 1.1895 | up |
| cellular_component | organelle | tr|E1C6V0|E1C6V0_CHICK | 1.577098 | up |
| cellular_component | organelle | tr|E1C7C1|E1C7C1_CHICK | -1.84307 | down |
| cellular_component | organelle | tr|E1C7J8|E1C7J8_CHICK | 1.184879 | up |
| cellular_component | organelle | tr|E1C7P7|E1C7P7_CHICK | 2.341246 | up |
| cellular_component | organelle | tr|E1C8A2|E1C8A2_CHICK | 1.307606 | up |
| cellular_component | organelle | tr|E1C8U1|E1C8U1_CHICK | 1.411305 | up |
| cellular_component | organelle | tr|E1C9H5|E1C9H5_CHICK | 1.195027 | up |
| cellular_component | organelle | tr|E7EC82|E7EC82_CHICK | 1.586027 | up |
| cellular_component | organelle | tr|F1N832|F1N832_CHICK | 1.427435 | up |
| cellular_component | organelle | tr|F1N977|F1N977_CHICK | 1.720618 | up |
| cellular_component | organelle | tr|F1N9U0|F1N9U0_CHICK | 1.038918 | up |
| cellular_component | organelle | tr|F1N9Y3|F1N9Y3_CHICK | 1.33841 | up |
| cellular_component | organelle | tr|F1NBX1|F1NBX1_CHICK | 1.439614 | up |
| cellular_component | organelle | tr|F1NBY5|F1NBY5_CHICK | 1.813459 | up |
| cellular_component | organelle | tr|F1NCM5|F1NCM5_CHICK | 2.577712 | up |
| cellular_component | organelle | tr|F1NDH2|F1NDH2_CHICK | -1.95135 | down |
| cellular_component | organelle | tr|F1NEA1|F1NEA1_CHICK | 1.395721 | up |
| cellular_component | organelle | tr|F1NEQ3|F1NEQ3_CHICK | 1.139952 | up |
| cellular_component | organelle | tr|F1NH23|F1NH23_CHICK | 1.850288 | up |
| cellular_component | organelle | tr|F1NIK3|F1NIK3_CHICK | 1.065249 | up |
| cellular_component | organelle | tr|F1NIQ3|F1NIQ3_CHICK | 1.483149 | up |
| cellular_component | organelle | tr|F1NIY3|F1NIY3_CHICK | 1.568578 | up |
| cellular_component | organelle | tr|F1NJK4|F1NJK4_CHICK | 1.788595 | up |
| cellular_component | organelle | tr|F1NJU5|F1NJU5_CHICK | -1.49981 | down |
| cellular_component | organelle | tr|F1NKY6|F1NKY6_CHICK | 1.171068 | up |
| cellular_component | organelle | tr|F1NMJ9|F1NMJ9_CHICK | -2.4895 | down |
| cellular_component | organelle | tr|F1NMU0|F1NMU0_CHICK | 2.467729 | up |
| cellular_component | organelle | tr|F1NPS5|F1NPS5_CHICK | 3.319197 | up |
| cellular_component | organelle | tr|F1NQ61|F1NQ61_CHICK | 1.080306 | up |
| cellular_component | organelle | tr|F1NRZ4|F1NRZ4_CHICK | 1.14911 | up |
| cellular_component | organelle | tr|F1NSD6|F1NSD6_CHICK | 1.689363 | up |
| cellular_component | organelle | tr|F1NSF2|F1NSF2_CHICK | 1.708711 | up |
| cellular_component | organelle | tr|F1NSQ4|F1NSQ4_CHICK | 1.21442 | up |
| cellular_component | organelle | tr|F1NT58|F1NT58_CHICK | 1.701877 | up |
| cellular_component | organelle | tr|F1NT80|F1NT80_CHICK | 1.404176 | up |
| cellular_component | organelle | tr|F1NTQ2|F1NTQ2_CHICK | 1.236275 | up |
| cellular_component | organelle | tr|F1NUY0|F1NUY0_CHICK | 1.570572 | up |
| cellular_component | organelle | tr|F1NUY9|F1NUY9_CHICK | 1.592258 | up |
| cellular_component | organelle | tr|F1NV66|F1NV66_CHICK | 1.865555 | up |
| cellular_component | organelle | tr|F1NXV6|F1NXV6_CHICK | -4.87705 | down |
| cellular_component | organelle | tr|F1NXZ7|F1NXZ7_CHICK | 1.050248 | up |
| cellular_component | organelle | tr|F1NYG7|F1NYG7_CHICK | 1.537967 | up |
| cellular_component | organelle | tr|F1P099|F1P099_CHICK | 1.399388 | up |
| cellular_component | organelle | tr|F1P151|F1P151_CHICK | 2.603153 | up |
| cellular_component | organelle | tr|F1P278|F1P278_CHICK | 1.88205 | up |
| cellular_component | organelle | tr|F1P2D2|F1P2D2_CHICK | 1.35416 | up |
| cellular_component | organelle | tr|F1P2G6|F1P2G6_CHICK | 1.470473 | up |
| cellular_component | organelle | tr|F1P394|F1P394_CHICK | 1.286828 | up |
| cellular_component | organelle | tr|F1P5A3|F1P5A3_CHICK | 2.127136 | up |
| cellular_component | organelle | tr|F1P5B8|F1P5B8_CHICK | 1.715588 | up |
| cellular_component | organelle | tr|F1P5J7|F1P5J7_CHICK | 1.075835 | up |
| cellular_component | organelle | tr|F6R228|F6R228_CHICK | 1.426396 | up |
| cellular_component | organelle | tr|F6YX81|F6YX81_CHICK | 1.194552 | up |
| cellular_component | organelle | tr|F7BFS9|F7BFS9_CHICK | 1.641443 | up |
| cellular_component | organelle | tr|F7BYG6|F7BYG6_CHICK | 3.125856 | up |
| cellular_component | organelle | tr|H9L0M7|H9L0M7_CHICK | 1.164684 | up |
| cellular_component | organelle | tr|I1SV11|I1SV11_CHICK | 1.313667 | up |
| cellular_component | organelle | tr|O93466|O93466_CHICK | 1.669881 | up |
| cellular_component | organelle | tr|Q2PUH1|Q2PUH1_CHICK | 1.468226 | up |
| cellular_component | organelle | tr|Q5DWF6|Q5DWF6_CHICK | 1.289716 | up |
| cellular_component | organelle | tr|Q5F351|Q5F351_CHICK | 1.342446 | up |
| cellular_component | organelle | tr|Q5F3R8|Q5F3R8_CHICK | 2.332553 | up |
| cellular_component | organelle | tr|Q5F3X1|Q5F3X1_CHICK | 1.19047 | up |
| cellular_component | organelle | tr|Q5F4B6|Q5F4B6_CHICK | 1.904086 | up |
| cellular_component | organelle | tr|Q5QRU7|Q5QRU7_CHICK | 2.017795 | up |
| cellular_component | organelle | tr|Q5ZHP4|Q5ZHP4_CHICK | 2.101948 | up |
| cellular_component | organelle | tr|Q5ZIL3|Q5ZIL3_CHICK | 1.863958 | up |
| cellular_component | organelle | tr|Q5ZIZ3|Q5ZIZ3_CHICK | 1.248317 | up |
| cellular_component | organelle | tr|Q5ZJ19|Q5ZJ19_CHICK | 2.470646 | up |
| cellular_component | organelle | tr|Q5ZJ96|Q5ZJ96_CHICK | 1.306532 | up |
| cellular_component | organelle | tr|Q5ZJA8|Q5ZJA8_CHICK | 1.093474 | up |
| cellular_component | organelle | tr|Q5ZJQ4|Q5ZJQ4_CHICK | 1.678561 | up |
| cellular_component | organelle | tr|Q5ZJW2|Q5ZJW2_CHICK | 1.71086 | up |
| cellular_component | organelle | tr|Q5ZKI3|Q5ZKI3_CHICK | 1.326993 | up |
| cellular_component | organelle | tr|Q5ZKY5|Q5ZKY5_CHICK | 1.620297 | up |
| cellular_component | organelle | tr|Q5ZL65|Q5ZL65_CHICK | 1.695268 | up |
| cellular_component | organelle | tr|Q5ZLI0|Q5ZLI0_CHICK | 1.097879 | up |
| cellular_component | organelle | tr|Q5ZMA7|Q5ZMA7_CHICK | 1.172026 | up |
| cellular_component | organelle | tr|Q5ZMJ6|Q5ZMJ6_CHICK | 1.845871 | up |
| cellular_component | organelle | tr|Q5ZMP2|Q5ZMP2_CHICK | 1.165624 | up |
| cellular_component | organelle | tr|Q9DDD4|Q9DDD4_CHICK | -1.44831 | down |
| cellular_component | organelle | tr|Q9I882|Q9I882_CHICK | 2.21964 | up |
| cellular_component | organelle | tr|Q9I8C8|Q9I8C8_CHICK | 1.2712 | up |
| cellular_component | organelle | tr|Q9PVL6|Q9PVL6_CHICK | 1.71949 | up |
| cellular_component | organelle | tr|R4GHH2|R4GHH2_CHICK | 1.778292 | up |
| cellular_component | organelle | tr|R4GHY3|R4GHY3_CHICK | 1.375649 | up |
| cellular_component | organelle | tr|R4GI86|R4GI86_CHICK | 2.078653 | up |
| cellular_component | organelle | tr|R4GJH2|R4GJH2_CHICK | 1.058464 | up |
| cellular_component | organelle | tr|R4GLW9|R4GLW9_CHICK | 1.120506 | up |
| cellular_component | organelle part | sp|O93436|STAM2_CHICK | 2.376336 | up |
| cellular_component | organelle part | sp|O93602|ATF2_CHICK | 2.071233 | up |
| cellular_component | organelle part | sp|P00523|SRC_CHICK | 1.599352 | up |
| cellular_component | organelle part | sp|P18936|NU1M_CHICK | 2.034658 | up |
| cellular_component | organelle part | sp|P52162|MAX_CHICK | 1.334512 | up |
| cellular_component | organelle part | sp|Q05423|FABP7_CHICK | 1.9874 | up |
| cellular_component | organelle part | sp|Q5F3Z3|UB2V2_CHICK | 2.366484 | up |
| cellular_component | organelle part | sp|Q5ZI08|SPT5H_CHICK | 1.235361 | up |
| cellular_component | organelle part | sp|Q5ZI74|DHX30_CHICK | 1.265146 | up |
| cellular_component | organelle part | sp|Q5ZIB9|ANM7_CHICK | 1.168118 | up |
| cellular_component | organelle part | sp|Q5ZII9|TFP11_CHICK | 1.172107 | up |
| cellular_component | organelle part | sp|Q5ZIK0|P4K2B_CHICK | 2.036191 | up |
| cellular_component | organelle part | sp|Q5ZIP4|XRN2_CHICK | 1.286156 | up |
| cellular_component | organelle part | sp|Q5ZJ00|EM55_CHICK | 1.664901 | up |
| cellular_component | organelle part | sp|Q5ZJ08|SYYC_CHICK | 1.23041 | up |
| cellular_component | organelle part | sp|Q5ZJA9|BORC5_CHICK | 1.655713 | up |
| cellular_component | organelle part | sp|Q5ZJB7|CHMP7_CHICK | 1.815716 | up |
| cellular_component | organelle part | sp|Q5ZJH9|DKC1_CHICK | 1.436701 | up |
| cellular_component | organelle part | sp|Q5ZJJ8|UBCP1_CHICK | 2.077308 | up |
| cellular_component | organelle part | sp|Q5ZK92|SPAST_CHICK | 1.665787 | up |
| cellular_component | organelle part | sp|Q5ZKA3|CWC22_CHICK | 1.734 | up |
| cellular_component | organelle part | sp|Q5ZKV4|NUBP2_CHICK | 1.740378 | up |
| cellular_component | organelle part | sp|Q5ZKV9|VPS50_CHICK | 1.324318 | up |
| cellular_component | organelle part | sp|Q5ZL36|ZFY27_CHICK | 1.948763 | up |
| cellular_component | organelle part | sp|Q5ZLD4|TMM11_CHICK | 1.73923 | up |
| cellular_component | organelle part | sp|Q5ZLT0|XPO7_CHICK | 1.469766 | up |
| cellular_component | organelle part | sp|Q5ZLV2|PPIL3_CHICK | 1.29867 | up |
| cellular_component | organelle part | sp|Q5ZM83|MIRO2_CHICK | 1.659999 | up |
| cellular_component | organelle part | sp|Q5ZML0|BABA2_CHICK | 1.144731 | up |
| cellular_component | organelle part | sp|Q9I8D0|VPP1_CHICK | 1.736096 | up |
| cellular_component | organelle part | sp|Q9IAM7|MRE11_CHICK | 1.337957 | up |
| cellular_component | organelle part | tr|A0A1D5NU82|A0A1D5NU82_CHICK | 1.12312 | up |
| cellular_component | organelle part | tr|A0A1D5NUD9|A0A1D5NUD9_CHICK | 1.157212 | up |
| cellular_component | organelle part | tr|A0A1D5NWJ7|A0A1D5NWJ7_CHICK | 1.195763 | up |
| cellular_component | organelle part | tr|A0A1D5NZB4|A0A1D5NZB4_CHICK | 1.671702 | up |
| cellular_component | organelle part | tr|A0A1D5NZS7|A0A1D5NZS7_CHICK | 1.185694 | up |
| cellular_component | organelle part | tr|A0A1D5P047|A0A1D5P047_CHICK | 1.659689 | up |
| cellular_component | organelle part | tr|A0A1D5P453|A0A1D5P453_CHICK | 1.553695 | up |
| cellular_component | organelle part | tr|A0A1D5P4B1|A0A1D5P4B1_CHICK | 1.156059 | up |
| cellular_component | organelle part | tr|A0A1D5P607|A0A1D5P607_CHICK | 1.51904 | up |
| cellular_component | organelle part | tr|A0A1D5P6C3|A0A1D5P6C3_CHICK | 2.100747 | up |
| cellular_component | organelle part | tr|A0A1D5P6M8|A0A1D5P6M8_CHICK | 1.82889 | up |
| cellular_component | organelle part | tr|A0A1D5P6S3|A0A1D5P6S3_CHICK | 1.183172 | up |
| cellular_component | organelle part | tr|A0A1D5P7C5|A0A1D5P7C5_CHICK | 1.273318 | up |
| cellular_component | organelle part | tr|A0A1D5P8H7|A0A1D5P8H7_CHICK | 1.530513 | up |
| cellular_component | organelle part | tr|A0A1D5P997|A0A1D5P997_CHICK | 1.563709 | up |
| cellular_component | organelle part | tr|A0A1D5PAM1|A0A1D5PAM1_CHICK | 2.577291 | up |
| cellular_component | organelle part | tr|A0A1D5PEE5|A0A1D5PEE5_CHICK | 1.102471 | up |
| cellular_component | organelle part | tr|A0A1D5PF37|A0A1D5PF37_CHICK | 1.238601 | up |
| cellular_component | organelle part | tr|A0A1D5PFS2|A0A1D5PFS2_CHICK | 1.463999 | up |
| cellular_component | organelle part | tr|A0A1D5PKQ7|A0A1D5PKQ7_CHICK | -1.34664 | down |
| cellular_component | organelle part | tr|A0A1D5PM87|A0A1D5PM87_CHICK | 1.108975 | up |
| cellular_component | organelle part | tr|A0A1D5PMR8|A0A1D5PMR8_CHICK | 1.213883 | up |
| cellular_component | organelle part | tr|A0A1D5PQ66|A0A1D5PQ66_CHICK | 1.664552 | up |
| cellular_component | organelle part | tr|A0A1D5PQD0|A0A1D5PQD0_CHICK | 1.314273 | up |
| cellular_component | organelle part | tr|A0A1D5PQQ6|A0A1D5PQQ6_CHICK | 1.432587 | up |
| cellular_component | organelle part | tr|A0A1D5PR34|A0A1D5PR34_CHICK | 1.370909 | up |
| cellular_component | organelle part | tr|A0A1D5PRI4|A0A1D5PRI4_CHICK | 1.28486 | up |
| cellular_component | organelle part | tr|A0A1D5PRI6|A0A1D5PRI6_CHICK | 1.580404 | up |
| cellular_component | organelle part | tr|A0A1D5PUJ5|A0A1D5PUJ5_CHICK | 1.266991 | up |
| cellular_component | organelle part | tr|A0A1D5PWD7|A0A1D5PWD7_CHICK | 1.676687 | up |
| cellular_component | organelle part | tr|A0A1D5PYU0|A0A1D5PYU0_CHICK | 1.138573 | up |
| cellular_component | organelle part | tr|A0A1L1RL50|A0A1L1RL50_CHICK | 1.173616 | up |
| cellular_component | organelle part | tr|A0A1L1RPT9|A0A1L1RPT9_CHICK | 1.353636 | up |
| cellular_component | organelle part | tr|A0A1L1RSG1|A0A1L1RSG1_CHICK | 1.889712 | up |
| cellular_component | organelle part | tr|A0A1L1RSS5|A0A1L1RSS5_CHICK | 1.143155 | up |
| cellular_component | organelle part | tr|A0A3Q2TUN2|A0A3Q2TUN2_CHICK | 1.036105 | up |
| cellular_component | organelle part | tr|A0A3Q2TVU0|A0A3Q2TVU0_CHICK | 1.280793 | up |
| cellular_component | organelle part | tr|A0A3Q2TVV0|A0A3Q2TVV0_CHICK | 1.145618 | up |
| cellular_component | organelle part | tr|A0A3Q2TY77|A0A3Q2TY77_CHICK | 1.82929 | up |
| cellular_component | organelle part | tr|A0A3Q2TYP7|A0A3Q2TYP7_CHICK | 2.159057 | up |
| cellular_component | organelle part | tr|A0A3Q2UB58|A0A3Q2UB58_CHICK | 2.070855 | up |
| cellular_component | organelle part | tr|A0A3Q2UBJ7|A0A3Q2UBJ7_CHICK | 1.945958 | up |
| cellular_component | organelle part | tr|A0A3Q2UCN1|A0A3Q2UCN1_CHICK | 1.92272 | up |
| cellular_component | organelle part | tr|A0A3Q2UD87|A0A3Q2UD87_CHICK | 1.205498 | up |
| cellular_component | organelle part | tr|A0A3Q3AB84|A0A3Q3AB84_CHICK | 1.170717 | up |
| cellular_component | organelle part | tr|A0A3Q3ALC8|A0A3Q3ALC8_CHICK | 2.188996 | up |
| cellular_component | organelle part | tr|A8VIF4|A8VIF4_CHICK | 1.024165 | up |
| cellular_component | organelle part | tr|D2D3P4|D2D3P4_CHICK | 2.146381 | up |
| cellular_component | organelle part | tr|E1BQI0|E1BQI0_CHICK | 1.067708 | up |
| cellular_component | organelle part | tr|E1BRR6|E1BRR6_CHICK | 1.332531 | up |
| cellular_component | organelle part | tr|E1BSH7|E1BSH7_CHICK | 1.527514 | up |
| cellular_component | organelle part | tr|E1BT94|E1BT94_CHICK | 1.49966 | up |
| cellular_component | organelle part | tr|E1BUJ1|E1BUJ1_CHICK | 2.709557 | up |
| cellular_component | organelle part | tr|E1BUX5|E1BUX5_CHICK | 1.099122 | up |
| cellular_component | organelle part | tr|E1BV97|E1BV97_CHICK | 2.036483 | up |
| cellular_component | organelle part | tr|E1BVC8|E1BVC8_CHICK | 2.179244 | up |
| cellular_component | organelle part | tr|E1BVQ3|E1BVQ3_CHICK | 1.650579 | up |
| cellular_component | organelle part | tr|E1BW27|E1BW27_CHICK | 1.938051 | up |
| cellular_component | organelle part | tr|E1BX21|E1BX21_CHICK | 1.42634 | up |
| cellular_component | organelle part | tr|E1BXT9|E1BXT9_CHICK | 1.600608 | up |
| cellular_component | organelle part | tr|E1BXY8|E1BXY8_CHICK | 1.106549 | up |
| cellular_component | organelle part | tr|E1BYQ3|E1BYQ3_CHICK | 1.202088 | up |
| cellular_component | organelle part | tr|E1C1Y5|E1C1Y5_CHICK | 1.135922 | up |
| cellular_component | organelle part | tr|E1C2F2|E1C2F2_CHICK | 1.546061 | up |
| cellular_component | organelle part | tr|E1C310|E1C310_CHICK | 1.545191 | up |
| cellular_component | organelle part | tr|E1C312|E1C312_CHICK | 1.811403 | up |
| cellular_component | organelle part | tr|E1C396|E1C396_CHICK | 1.08258 | up |
| cellular_component | organelle part | tr|E1C3U6|E1C3U6_CHICK | 1.144268 | up |
| cellular_component | organelle part | tr|E1C593|E1C593_CHICK | 1.922793 | up |
| cellular_component | organelle part | tr|E1C688|E1C688_CHICK | 1.775755 | up |
| cellular_component | organelle part | tr|E1C6S7|E1C6S7_CHICK | 1.1895 | up |
| cellular_component | organelle part | tr|E1C8A2|E1C8A2_CHICK | 1.307606 | up |
| cellular_component | organelle part | tr|E1C8U1|E1C8U1_CHICK | 1.411305 | up |
| cellular_component | organelle part | tr|E7EC82|E7EC82_CHICK | 1.586027 | up |
| cellular_component | organelle part | tr|F1N832|F1N832_CHICK | 1.427435 | up |
| cellular_component | organelle part | tr|F1N9U0|F1N9U0_CHICK | 1.038918 | up |
| cellular_component | organelle part | tr|F1NEA1|F1NEA1_CHICK | 1.395721 | up |
| cellular_component | organelle part | tr|F1NEQ3|F1NEQ3_CHICK | 1.139952 | up |
| cellular_component | organelle part | tr|F1NH23|F1NH23_CHICK | 1.850288 | up |
| cellular_component | organelle part | tr|F1NIK3|F1NIK3_CHICK | 1.065249 | up |
| cellular_component | organelle part | tr|F1NIQ3|F1NIQ3_CHICK | 1.483149 | up |
| cellular_component | organelle part | tr|F1NKY6|F1NKY6_CHICK | 1.171068 | up |
| cellular_component | organelle part | tr|F1NMU0|F1NMU0_CHICK | 2.467729 | up |
| cellular_component | organelle part | tr|F1NPS5|F1NPS5_CHICK | 3.319197 | up |
| cellular_component | organelle part | tr|F1NQ61|F1NQ61_CHICK | 1.080306 | up |
| cellular_component | organelle part | tr|F1NRZ4|F1NRZ4_CHICK | 1.14911 | up |
| cellular_component | organelle part | tr|F1NSD6|F1NSD6_CHICK | 1.689363 | up |
| cellular_component | organelle part | tr|F1NSQ4|F1NSQ4_CHICK | 1.21442 | up |
| cellular_component | organelle part | tr|F1NT80|F1NT80_CHICK | 1.404176 | up |
| cellular_component | organelle part | tr|F1NV66|F1NV66_CHICK | 1.865555 | up |
| cellular_component | organelle part | tr|F1NXZ7|F1NXZ7_CHICK | 1.050248 | up |
| cellular_component | organelle part | tr|F1P151|F1P151_CHICK | 2.603153 | up |
| cellular_component | organelle part | tr|F1P2G6|F1P2G6_CHICK | 1.470473 | up |
| cellular_component | organelle part | tr|F1P394|F1P394_CHICK | 1.286828 | up |
| cellular_component | organelle part | tr|F1P5A3|F1P5A3_CHICK | 2.127136 | up |
| cellular_component | organelle part | tr|F1P5B8|F1P5B8_CHICK | 1.715588 | up |
| cellular_component | organelle part | tr|F1P5J7|F1P5J7_CHICK | 1.075835 | up |
| cellular_component | organelle part | tr|F6R228|F6R228_CHICK | 1.426396 | up |
| cellular_component | organelle part | tr|F7BFS9|F7BFS9_CHICK | 1.641443 | up |
| cellular_component | organelle part | tr|H9L0M7|H9L0M7_CHICK | 1.164684 | up |
| cellular_component | organelle part | tr|O93466|O93466_CHICK | 1.669881 | up |
| cellular_component | organelle part | tr|Q2PUH1|Q2PUH1_CHICK | 1.468226 | up |
| cellular_component | organelle part | tr|Q5DWF6|Q5DWF6_CHICK | 1.289716 | up |
| cellular_component | organelle part | tr|Q5F351|Q5F351_CHICK | 1.342446 | up |
| cellular_component | organelle part | tr|Q5F3R8|Q5F3R8_CHICK | 2.332553 | up |
| cellular_component | organelle part | tr|Q5F4B6|Q5F4B6_CHICK | 1.904086 | up |
| cellular_component | organelle part | tr|Q5QRU7|Q5QRU7_CHICK | 2.017795 | up |
| cellular_component | organelle part | tr|Q5ZIZ3|Q5ZIZ3_CHICK | 1.248317 | up |
| cellular_component | organelle part | tr|Q5ZJ19|Q5ZJ19_CHICK | 2.470646 | up |
| cellular_component | organelle part | tr|Q5ZJ96|Q5ZJ96_CHICK | 1.306532 | up |
| cellular_component | organelle part | tr|Q5ZJA8|Q5ZJA8_CHICK | 1.093474 | up |
| cellular_component | organelle part | tr|Q5ZJW2|Q5ZJW2_CHICK | 1.71086 | up |
| cellular_component | organelle part | tr|Q5ZKY5|Q5ZKY5_CHICK | 1.620297 | up |
| cellular_component | organelle part | tr|Q5ZLI0|Q5ZLI0_CHICK | 1.097879 | up |
| cellular_component | organelle part | tr|Q5ZMJ6|Q5ZMJ6_CHICK | 1.845871 | up |
| cellular_component | organelle part | tr|Q5ZMP2|Q5ZMP2_CHICK | 1.165624 | up |
| cellular_component | organelle part | tr|Q9DDD4|Q9DDD4_CHICK | -1.44831 | down |
| cellular_component | organelle part | tr|Q9I8C8|Q9I8C8_CHICK | 1.2712 | up |
| cellular_component | organelle part | tr|R4GLW9|R4GLW9_CHICK | 1.120506 | up |
| cellular_component | supramolecular complex | sp|Q5ZK92|SPAST_CHICK | 1.665787 | up |
| cellular_component | supramolecular complex | tr|A0A1D5NU82|A0A1D5NU82_CHICK | 1.12312 | up |
| cellular_component | supramolecular complex | tr|A0A1D5P453|A0A1D5P453_CHICK | 1.553695 | up |
| cellular_component | supramolecular complex | tr|E1BYQ3|E1BYQ3_CHICK | 1.202088 | up |
| cellular_component | supramolecular complex | tr|E1C312|E1C312_CHICK | 1.811403 | up |
| cellular_component | supramolecular complex | tr|E7EC82|E7EC82_CHICK | 1.586027 | up |
| cellular_component | supramolecular complex | tr|F1NSD6|F1NSD6_CHICK | 1.689363 | up |
| cellular_component | supramolecular complex | tr|O93466|O93466_CHICK | 1.669881 | up |
| cellular_component | supramolecular complex | tr|Q5F351|Q5F351_CHICK | 1.342446 | up |
| cellular_component | supramolecular complex | tr|Q5ZKY5|Q5ZKY5_CHICK | 1.620297 | up |
| cellular_component | synapse | sp|P07090|CALB2_CHICK | 1.954973 | up |
| cellular_component | synapse | sp|Q5ZIK2|PDZ11_CHICK | 1.259402 | up |
| cellular_component | synapse | tr|A0A3Q2TY77|A0A3Q2TY77_CHICK | 1.82929 | up |
| cellular_component | synapse | tr|E1BVC8|E1BVC8_CHICK | 2.179244 | up |
| cellular_component | synapse | tr|F1NEA1|F1NEA1_CHICK | 1.395721 | up |
| cellular_component | synapse | tr|F1NT80|F1NT80_CHICK | 1.404176 | up |
| cellular_component | synapse | tr|Q5F3R8|Q5F3R8_CHICK | 2.332553 | up |
| cellular_component | synapse part | sp|Q5ZIK2|PDZ11_CHICK | 1.259402 | up |
| cellular_component | synapse part | tr|A0A3Q2TY77|A0A3Q2TY77_CHICK | 1.82929 | up |
| cellular_component | synapse part | tr|E1BVC8|E1BVC8_CHICK | 2.179244 | up |
| cellular_component | synapse part | tr|F1NEA1|F1NEA1_CHICK | 1.395721 | up |
| cellular_component | synapse part | tr|F1NT80|F1NT80_CHICK | 1.404176 | up |
| cellular_component | synapse part | tr|Q5F3R8|Q5F3R8_CHICK | 2.332553 | up |
| molecular_function | antioxidant activity | tr|A0A3Q3AIE0|A0A3Q3AIE0_CHICK | 1.346321 | up |
| molecular_function | binding | sp|O42395|CNBP_CHICK | 1.365125 | up |
| molecular_function | binding | sp|O93436|STAM2_CHICK | 2.376336 | up |
| molecular_function | binding | sp|O93602|ATF2_CHICK | 2.071233 | up |
| molecular_function | binding | sp|P00523|SRC_CHICK | 1.599352 | up |
| molecular_function | binding | sp|P02197|MYG_CHICK | 2.885059 | up |
| molecular_function | binding | sp|P07090|CALB2_CHICK | 1.954973 | up |
| molecular_function | binding | sp|P17923|NFIA_CHICK | 1.346459 | up |
| molecular_function | binding | sp|P25155|FA10_CHICK | -3.77432 | down |
| molecular_function | binding | sp|P28318|M126_CHICK | 3.846633 | up |
| molecular_function | binding | sp|P42292|CD166_CHICK | 1.297212 | up |
| molecular_function | binding | sp|P52162|MAX_CHICK | 1.334512 | up |
| molecular_function | binding | sp|Q05423|FABP7_CHICK | 1.9874 | up |
| molecular_function | binding | sp|Q5F3Z3|UB2V2_CHICK | 2.366484 | up |
| molecular_function | binding | sp|Q5F480|ITPK1_CHICK | 1.066359 | up |
| molecular_function | binding | sp|Q5ZHN3|WIPI2_CHICK | 1.61465 | up |
| molecular_function | binding | sp|Q5ZHT1|ACD11_CHICK | 1.653324 | up |
| molecular_function | binding | sp|Q5ZI08|SPT5H_CHICK | 1.235361 | up |
| molecular_function | binding | sp|Q5ZI74|DHX30_CHICK | 1.265146 | up |
| molecular_function | binding | sp|Q5ZIB9|ANM7_CHICK | 1.168118 | up |
| molecular_function | binding | sp|Q5ZID0|NMRL1_CHICK | 2.429369 | up |
| molecular_function | binding | sp|Q5ZII9|TFP11_CHICK | 1.172107 | up |
| molecular_function | binding | sp|Q5ZIK0|P4K2B_CHICK | 2.036191 | up |
| molecular_function | binding | sp|Q5ZIK2|PDZ11_CHICK | 1.259402 | up |
| molecular_function | binding | sp|Q5ZIP4|XRN2_CHICK | 1.286156 | up |
| molecular_function | binding | sp|Q5ZJ08|SYYC_CHICK | 1.23041 | up |
| molecular_function | binding | sp|Q5ZJH6|ULK3_CHICK | 1.593805 | up |
| molecular_function | binding | sp|Q5ZJH9|DKC1_CHICK | 1.436701 | up |
| molecular_function | binding | sp|Q5ZJJ1|ZC11A_CHICK | 2.191487 | up |
| molecular_function | binding | sp|Q5ZJW6|KBRS2_CHICK | 1.256362 | up |
| molecular_function | binding | sp|Q5ZK05|TOLIP_CHICK | 1.088317 | up |
| molecular_function | binding | sp|Q5ZK92|SPAST_CHICK | 1.665787 | up |
| molecular_function | binding | sp|Q5ZKA3|CWC22_CHICK | 1.734 | up |
| molecular_function | binding | sp|Q5ZKV4|NUBP2_CHICK | 1.740378 | up |
| molecular_function | binding | sp|Q5ZKV9|VPS50_CHICK | 1.324318 | up |
| molecular_function | binding | sp|Q5ZL36|ZFY27_CHICK | 1.948763 | up |
| molecular_function | binding | sp|Q5ZLT0|XPO7_CHICK | 1.469766 | up |
| molecular_function | binding | sp|Q5ZM83|MIRO2_CHICK | 1.659999 | up |
| molecular_function | binding | sp|Q5ZMG0|DOPD_CHICK | 2.121364 | up |
| molecular_function | binding | sp|Q5ZML0|BABA2_CHICK | 1.144731 | up |
| molecular_function | binding | sp|Q804X6|FA9_CHICK | -5.71386 | down |
| molecular_function | binding | sp|Q8AXL1|SAT1_CHICK | 1.12819 | up |
| molecular_function | binding | sp|Q90733|COT2_CHICK | 1.595685 | up |
| molecular_function | binding | sp|Q98SH2|AT2B1_CHICK | 1.799719 | up |
| molecular_function | binding | sp|Q9I8D0|VPP1_CHICK | 1.736096 | up |
| molecular_function | binding | sp|Q9IAM7|MRE11_CHICK | 1.337957 | up |
| molecular_function | binding | tr|A0A1D5NU82|A0A1D5NU82_CHICK | 1.12312 | up |
| molecular_function | binding | tr|A0A1D5NUD9|A0A1D5NUD9_CHICK | 1.157212 | up |
| molecular_function | binding | tr|A0A1D5NUU0|A0A1D5NUU0_CHICK | 1.000304 | up |
| molecular_function | binding | tr|A0A1D5NUV0|A0A1D5NUV0_CHICK | 2.157037 | up |
| molecular_function | binding | tr|A0A1D5NVT0|A0A1D5NVT0_CHICK | 1.421036 | up |
| molecular_function | binding | tr|A0A1D5NWV6|A0A1D5NWV6_CHICK | 1.553336 | up |
| molecular_function | binding | tr|A0A1D5NX04|A0A1D5NX04_CHICK | 2.142264 | up |
| molecular_function | binding | tr|A0A1D5NX89|A0A1D5NX89_CHICK | 1.420007 | up |
| molecular_function | binding | tr|A0A1D5NXG4|A0A1D5NXG4_CHICK | 1.245324 | up |
| molecular_function | binding | tr|A0A1D5NXJ2|A0A1D5NXJ2_CHICK | 1.224878 | up |
| molecular_function | binding | tr|A0A1D5NZB4|A0A1D5NZB4_CHICK | 1.671702 | up |
| molecular_function | binding | tr|A0A1D5NZB9|A0A1D5NZB9_CHICK | 1.567222 | up |
| molecular_function | binding | tr|A0A1D5NZG5|A0A1D5NZG5_CHICK | 1.205698 | up |
| molecular_function | binding | tr|A0A1D5NZS7|A0A1D5NZS7_CHICK | 1.185694 | up |
| molecular_function | binding | tr|A0A1D5NZW0|A0A1D5NZW0_CHICK | 1.956911 | up |
| molecular_function | binding | tr|A0A1D5NZZ9|A0A1D5NZZ9_CHICK | 2.286092 | up |
| molecular_function | binding | tr|A0A1D5P047|A0A1D5P047_CHICK | 1.659689 | up |
| molecular_function | binding | tr|A0A1D5P140|A0A1D5P140_CHICK | 1.368373 | up |
| molecular_function | binding | tr|A0A1D5P453|A0A1D5P453_CHICK | 1.553695 | up |
| molecular_function | binding | tr|A0A1D5P5X4|A0A1D5P5X4_CHICK | 1.559711 | up |
| molecular_function | binding | tr|A0A1D5P607|A0A1D5P607_CHICK | 1.51904 | up |
| molecular_function | binding | tr|A0A1D5P6Y6|A0A1D5P6Y6_CHICK | 1.32218 | up |
| molecular_function | binding | tr|A0A1D5P756|A0A1D5P756_CHICK | 1.203038 | up |
| molecular_function | binding | tr|A0A1D5P7C5|A0A1D5P7C5_CHICK | 1.273318 | up |
| molecular_function | binding | tr|A0A1D5P7E3|A0A1D5P7E3_CHICK | 1.017643 | up |
| molecular_function | binding | tr|A0A1D5P8H7|A0A1D5P8H7_CHICK | 1.530513 | up |
| molecular_function | binding | tr|A0A1D5P8V8|A0A1D5P8V8_CHICK | 1.608897 | up |
| molecular_function | binding | tr|A0A1D5P997|A0A1D5P997_CHICK | 1.563709 | up |
| molecular_function | binding | tr|A0A1D5P9G2|A0A1D5P9G2_CHICK | 1.768438 | up |
| molecular_function | binding | tr|A0A1D5PA08|A0A1D5PA08_CHICK | 1.946165 | up |
| molecular_function | binding | tr|A0A1D5PAM1|A0A1D5PAM1_CHICK | 2.577291 | up |
| molecular_function | binding | tr|A0A1D5PCZ1|A0A1D5PCZ1_CHICK | 1.542638 | up |
| molecular_function | binding | tr|A0A1D5PEE5|A0A1D5PEE5_CHICK | 1.102471 | up |
| molecular_function | binding | tr|A0A1D5PFS2|A0A1D5PFS2_CHICK | 1.463999 | up |
| molecular_function | binding | tr|A0A1D5PFX4|A0A1D5PFX4_CHICK | 1.061266 | up |
| molecular_function | binding | tr|A0A1D5PG53|A0A1D5PG53_CHICK | 2.149242 | up |
| molecular_function | binding | tr|A0A1D5PGB1|A0A1D5PGB1_CHICK | 1.43532 | up |
| molecular_function | binding | tr|A0A1D5PHB6|A0A1D5PHB6_CHICK | 1.553464 | up |
| molecular_function | binding | tr|A0A1D5PHE2|A0A1D5PHE2_CHICK | 2.577604 | up |
| molecular_function | binding | tr|A0A1D5PHL1|A0A1D5PHL1_CHICK | 1.487838 | up |
| molecular_function | binding | tr|A0A1D5PHS9|A0A1D5PHS9_CHICK | 1.645384 | up |
| molecular_function | binding | tr|A0A1D5PHV9|A0A1D5PHV9_CHICK | 1.32812 | up |
| molecular_function | binding | tr|A0A1D5PIB1|A0A1D5PIB1_CHICK | 2.10824 | up |
| molecular_function | binding | tr|A0A1D5PIT4|A0A1D5PIT4_CHICK | -1.97988 | down |
| molecular_function | binding | tr|A0A1D5PJL7|A0A1D5PJL7_CHICK | 1.22028 | up |
| molecular_function | binding | tr|A0A1D5PK48|A0A1D5PK48_CHICK | -5.8308 | down |
| molecular_function | binding | tr|A0A1D5PKI2|A0A1D5PKI2_CHICK | 1.392336 | up |
| molecular_function | binding | tr|A0A1D5PM26|A0A1D5PM26_CHICK | 2.591272 | up |
| molecular_function | binding | tr|A0A1D5PMK7|A0A1D5PMK7_CHICK | 1.899201 | up |
| molecular_function | binding | tr|A0A1D5PMR8|A0A1D5PMR8_CHICK | 1.213883 | up |
| molecular_function | binding | tr|A0A1D5PPH4|A0A1D5PPH4_CHICK | 1.254155 | up |
| molecular_function | binding | tr|A0A1D5PQ66|A0A1D5PQ66_CHICK | 1.664552 | up |
| molecular_function | binding | tr|A0A1D5PQG6|A0A1D5PQG6_CHICK | 2.091788 | up |
| molecular_function | binding | tr|A0A1D5PRI4|A0A1D5PRI4_CHICK | 1.28486 | up |
| molecular_function | binding | tr|A0A1D5PRI6|A0A1D5PRI6_CHICK | 1.580404 | up |
| molecular_function | binding | tr|A0A1D5PTE8|A0A1D5PTE8_CHICK | 1.960486 | up |
| molecular_function | binding | tr|A0A1D5PWD7|A0A1D5PWD7_CHICK | 1.676687 | up |
| molecular_function | binding | tr|A0A1D5PXN4|A0A1D5PXN4_CHICK | -1.85723 | down |
| molecular_function | binding | tr|A0A1D5PYB7|A0A1D5PYB7_CHICK | 1.489522 | up |
| molecular_function | binding | tr|A0A1D5PYU0|A0A1D5PYU0_CHICK | 1.138573 | up |
| molecular_function | binding | tr|A0A1D5PZ78|A0A1D5PZ78_CHICK | 1.936124 | up |
| molecular_function | binding | tr|A0A1D5Q008|A0A1D5Q008_CHICK | 2.962255 | up |
| molecular_function | binding | tr|A0A1L1RPB1|A0A1L1RPB1_CHICK | 1.864507 | up |
| molecular_function | binding | tr|A0A1L1RWR0|A0A1L1RWR0_CHICK | -2.84844 | down |
| molecular_function | binding | tr|A0A1L1RXF5|A0A1L1RXF5_CHICK | 1.278974 | up |
| molecular_function | binding | tr|A0A3Q2TVU0|A0A3Q2TVU0_CHICK | 1.280793 | up |
| molecular_function | binding | tr|A0A3Q2TVV0|A0A3Q2TVV0_CHICK | 1.145618 | up |
| molecular_function | binding | tr|A0A3Q2TY77|A0A3Q2TY77_CHICK | 1.82929 | up |
| molecular_function | binding | tr|A0A3Q2TYP7|A0A3Q2TYP7_CHICK | 2.159057 | up |
| molecular_function | binding | tr|A0A3Q2TZM7|A0A3Q2TZM7_CHICK | 1.419267 | up |
| molecular_function | binding | tr|A0A3Q2U0V6|A0A3Q2U0V6_CHICK | 1.411673 | up |
| molecular_function | binding | tr|A0A3Q2U3C4|A0A3Q2U3C4_CHICK | 1.094483 | up |
| molecular_function | binding | tr|A0A3Q2U504|A0A3Q2U504_CHICK | -2.35777 | down |
| molecular_function | binding | tr|A0A3Q2UB58|A0A3Q2UB58_CHICK | 2.070855 | up |
| molecular_function | binding | tr|A0A3Q2UCN1|A0A3Q2UCN1_CHICK | 1.92272 | up |
| molecular_function | binding | tr|A0A3Q2UCP8|A0A3Q2UCP8_CHICK | 2.280368 | up |
| molecular_function | binding | tr|A0A3Q2UCQ1|A0A3Q2UCQ1_CHICK | 1.349205 | up |
| molecular_function | binding | tr|A0A3Q2UG55|A0A3Q2UG55_CHICK | -2.8915 | down |
| molecular_function | binding | tr|A0A3Q2UK74|A0A3Q2UK74_CHICK | 2.254776 | up |
| molecular_function | binding | tr|A0A3Q2UPF3|A0A3Q2UPF3_CHICK | 1.295517 | up |
| molecular_function | binding | tr|A0A3Q3AB84|A0A3Q3AB84_CHICK | 1.170717 | up |
| molecular_function | binding | tr|A0A3Q3ALC8|A0A3Q3ALC8_CHICK | 2.188996 | up |
| molecular_function | binding | tr|A0A3Q3APG2|A0A3Q3APG2_CHICK | 1.640987 | up |
| molecular_function | binding | tr|A0A3S5ZPH2|A0A3S5ZPH2_CHICK | 1.677715 | up |
| molecular_function | binding | tr|A8VIF4|A8VIF4_CHICK | 1.024165 | up |
| molecular_function | binding | tr|D2D3P4|D2D3P4_CHICK | 2.146381 | up |
| molecular_function | binding | tr|E1BQC2|E1BQC2_CHICK | -2.52084 | down |
| molecular_function | binding | tr|E1BQI0|E1BQI0_CHICK | 1.067708 | up |
| molecular_function | binding | tr|E1BQU2|E1BQU2_CHICK | 1.629018 | up |
| molecular_function | binding | tr|E1BRR6|E1BRR6_CHICK | 1.332531 | up |
| molecular_function | binding | tr|E1BSH7|E1BSH7_CHICK | 1.527514 | up |
| molecular_function | binding | tr|E1BT44|E1BT44_CHICK | 1.432225 | up |
| molecular_function | binding | tr|E1BTI7|E1BTI7_CHICK | 1.813027 | up |
| molecular_function | binding | tr|E1BTV1|E1BTV1_CHICK | 2.001762 | up |
| molecular_function | binding | tr|E1BU89|E1BU89_CHICK | 1.303565 | up |
| molecular_function | binding | tr|E1BUJ1|E1BUJ1_CHICK | 2.709557 | up |
| molecular_function | binding | tr|E1BUX5|E1BUX5_CHICK | 1.099122 | up |
| molecular_function | binding | tr|E1BV97|E1BV97_CHICK | 2.036483 | up |
| molecular_function | binding | tr|E1BVQ3|E1BVQ3_CHICK | 1.650579 | up |
| molecular_function | binding | tr|E1BWB7|E1BWB7_CHICK | 1.446045 | up |
| molecular_function | binding | tr|E1BWG7|E1BWG7_CHICK | 2.421508 | up |
| molecular_function | binding | tr|E1BX21|E1BX21_CHICK | 1.42634 | up |
| molecular_function | binding | tr|E1BXC2|E1BXC2_CHICK | 1.767478 | up |
| molecular_function | binding | tr|E1BXY8|E1BXY8_CHICK | 1.106549 | up |
| molecular_function | binding | tr|E1BY22|E1BY22_CHICK | 1.367672 | up |
| molecular_function | binding | tr|E1BY40|E1BY40_CHICK | 1.129106 | up |
| molecular_function | binding | tr|E1BYQ3|E1BYQ3_CHICK | 1.202088 | up |
| molecular_function | binding | tr|E1BZ74|E1BZ74_CHICK | 1.217761 | up |
| molecular_function | binding | tr|E1BZ79|E1BZ79_CHICK | 2.388844 | up |
| molecular_function | binding | tr|E1C1G5|E1C1G5_CHICK | 1.360319 | up |
| molecular_function | binding | tr|E1C229|E1C229_CHICK | 1.473036 | up |
| molecular_function | binding | tr|E1C2E5|E1C2E5_CHICK | 1.182299 | up |
| molecular_function | binding | tr|E1C2F2|E1C2F2_CHICK | 1.546061 | up |
| molecular_function | binding | tr|E1C312|E1C312_CHICK | 1.811403 | up |
| molecular_function | binding | tr|E1C396|E1C396_CHICK | 1.08258 | up |
| molecular_function | binding | tr|E1C483|E1C483_CHICK | 1.301431 | up |
| molecular_function | binding | tr|E1C593|E1C593_CHICK | 1.922793 | up |
| molecular_function | binding | tr|E1C5B8|E1C5B8_CHICK | 2.15124 | up |
| molecular_function | binding | tr|E1C688|E1C688_CHICK | 1.775755 | up |
| molecular_function | binding | tr|E1C6S7|E1C6S7_CHICK | 1.1895 | up |
| molecular_function | binding | tr|E1C6V0|E1C6V0_CHICK | 1.577098 | up |
| molecular_function | binding | tr|E1C7A7|E1C7A7_CHICK | -1.02532 | down |
| molecular_function | binding | tr|E1C7J8|E1C7J8_CHICK | 1.184879 | up |
| molecular_function | binding | tr|E1C8U1|E1C8U1_CHICK | 1.411305 | up |
| molecular_function | binding | tr|E1C9H5|E1C9H5_CHICK | 1.195027 | up |
| molecular_function | binding | tr|E7EC82|E7EC82_CHICK | 1.586027 | up |
| molecular_function | binding | tr|F1N832|F1N832_CHICK | 1.427435 | up |
| molecular_function | binding | tr|F1N8V5|F1N8V5_CHICK | 2.344853 | up |
| molecular_function | binding | tr|F1N9S3|F1N9S3_CHICK | 1.256683 | up |
| molecular_function | binding | tr|F1N9U0|F1N9U0_CHICK | 1.038918 | up |
| molecular_function | binding | tr|F1N9Y3|F1N9Y3_CHICK | 1.33841 | up |
| molecular_function | binding | tr|F1NBX1|F1NBX1_CHICK | 1.439614 | up |
| molecular_function | binding | tr|F1NDH2|F1NDH2_CHICK | -1.95135 | down |
| molecular_function | binding | tr|F1NDZ7|F1NDZ7_CHICK | 1.988751 | up |
| molecular_function | binding | tr|F1NEQ3|F1NEQ3_CHICK | 1.139952 | up |
| molecular_function | binding | tr|F1NGU0|F1NGU0_CHICK | 1.010874 | up |
| molecular_function | binding | tr|F1NIK3|F1NIK3_CHICK | 1.065249 | up |
| molecular_function | binding | tr|F1NIY3|F1NIY3_CHICK | 1.568578 | up |
| molecular_function | binding | tr|F1NLM0|F1NLM0_CHICK | 1.884615 | up |
| molecular_function | binding | tr|F1NMJ9|F1NMJ9_CHICK | -2.4895 | down |
| molecular_function | binding | tr|F1NMM9|F1NMM9_CHICK | 1.449446 | up |
| molecular_function | binding | tr|F1NN63|F1NN63_CHICK | 1.72615 | up |
| molecular_function | binding | tr|F1NPS5|F1NPS5_CHICK | 3.319197 | up |
| molecular_function | binding | tr|F1NQY0|F1NQY0_CHICK | 1.686257 | up |
| molecular_function | binding | tr|F1NRZ4|F1NRZ4_CHICK | 1.14911 | up |
| molecular_function | binding | tr|F1NSF2|F1NSF2_CHICK | 1.708711 | up |
| molecular_function | binding | tr|F1NSI7|F1NSI7_CHICK | 1.77407 | up |
| molecular_function | binding | tr|F1NSQ4|F1NSQ4_CHICK | 1.21442 | up |
| molecular_function | binding | tr|F1NT80|F1NT80_CHICK | 1.404176 | up |
| molecular_function | binding | tr|F1NTQ2|F1NTQ2_CHICK | 1.236275 | up |
| molecular_function | binding | tr|F1NUY0|F1NUY0_CHICK | 1.570572 | up |
| molecular_function | binding | tr|F1NXV6|F1NXV6_CHICK | -4.87705 | down |
| molecular_function | binding | tr|F1NYG7|F1NYG7_CHICK | 1.537967 | up |
| molecular_function | binding | tr|F1P204|F1P204_CHICK | 2.129437 | up |
| molecular_function | binding | tr|F1P278|F1P278_CHICK | 1.88205 | up |
| molecular_function | binding | tr|F1P372|F1P372_CHICK | 1.525043 | up |
| molecular_function | binding | tr|F1P394|F1P394_CHICK | 1.286828 | up |
| molecular_function | binding | tr|F1P4A7|F1P4A7_CHICK | 2.008749 | up |
| molecular_function | binding | tr|F1P4D7|F1P4D7_CHICK | 1.032498 | up |
| molecular_function | binding | tr|F1P526|F1P526_CHICK | 1.506479 | up |
| molecular_function | binding | tr|F1P5B8|F1P5B8_CHICK | 1.715588 | up |
| molecular_function | binding | tr|F1P5J7|F1P5J7_CHICK | 1.075835 | up |
| molecular_function | binding | tr|F6R228|F6R228_CHICK | 1.426396 | up |
| molecular_function | binding | tr|I1SV11|I1SV11_CHICK | 1.313667 | up |
| molecular_function | binding | tr|M9NCD4|M9NCD4_CHICK | 1.22338 | up |
| molecular_function | binding | tr|O93466|O93466_CHICK | 1.669881 | up |
| molecular_function | binding | tr|Q2PUH1|Q2PUH1_CHICK | 1.468226 | up |
| molecular_function | binding | tr|Q4ADJ6|Q4ADJ6_CHICK | -4.04825 | down |
| molecular_function | binding | tr|Q5DWF6|Q5DWF6_CHICK | 1.289716 | up |
| molecular_function | binding | tr|Q5F351|Q5F351_CHICK | 1.342446 | up |
| molecular_function | binding | tr|Q5F3R8|Q5F3R8_CHICK | 2.332553 | up |
| molecular_function | binding | tr|Q5F3X1|Q5F3X1_CHICK | 1.19047 | up |
| molecular_function | binding | tr|Q5F4B6|Q5F4B6_CHICK | 1.904086 | up |
| molecular_function | binding | tr|Q5QRU7|Q5QRU7_CHICK | 2.017795 | up |
| molecular_function | binding | tr|Q5ZHP2|Q5ZHP2_CHICK | 2.412922 | up |
| molecular_function | binding | tr|Q5ZHP4|Q5ZHP4_CHICK | 2.101948 | up |
| molecular_function | binding | tr|Q5ZIL3|Q5ZIL3_CHICK | 1.863958 | up |
| molecular_function | binding | tr|Q5ZJ19|Q5ZJ19_CHICK | 2.470646 | up |
| molecular_function | binding | tr|Q5ZJ96|Q5ZJ96_CHICK | 1.306532 | up |
| molecular_function | binding | tr|Q5ZJA8|Q5ZJA8_CHICK | 1.093474 | up |
| molecular_function | binding | tr|Q5ZJQ4|Q5ZJQ4_CHICK | 1.678561 | up |
| molecular_function | binding | tr|Q5ZKI3|Q5ZKI3_CHICK | 1.326993 | up |
| molecular_function | binding | tr|Q5ZKY5|Q5ZKY5_CHICK | 1.620297 | up |
| molecular_function | binding | tr|Q5ZLI0|Q5ZLI0_CHICK | 1.097879 | up |
| molecular_function | binding | tr|Q5ZMA7|Q5ZMA7_CHICK | 1.172026 | up |
| molecular_function | binding | tr|Q5ZMG8|Q5ZMG8_CHICK | 1.771296 | up |
| molecular_function | binding | tr|Q5ZMP2|Q5ZMP2_CHICK | 1.165624 | up |
| molecular_function | binding | tr|Q90W83|Q90W83_CHICK | 2.57878 | up |
| molecular_function | binding | tr|Q9DDD4|Q9DDD4_CHICK | -1.44831 | down |
| molecular_function | binding | tr|Q9I882|Q9I882_CHICK | 2.21964 | up |
| molecular_function | binding | tr|Q9PUJ4|Q9PUJ4_CHICK | 1.664957 | up |
| molecular_function | binding | tr|R4GI86|R4GI86_CHICK | 2.078653 | up |
| molecular_function | binding | tr|V9GW30|V9GW30_CHICK | 1.560922 | up |
| molecular_function | catalytic activity | sp|O93602|ATF2_CHICK | 2.071233 | up |
| molecular_function | catalytic activity | sp|P00337|LDHB_CHICK | 2.131478 | up |
| molecular_function | catalytic activity | sp|P00523|SRC_CHICK | 1.599352 | up |
| molecular_function | catalytic activity | sp|P00940|TPIS_CHICK | 2.162937 | up |
| molecular_function | catalytic activity | sp|P18936|NU1M_CHICK | 2.034658 | up |
| molecular_function | catalytic activity | sp|P20136|GSTM2_CHICK | 2.226935 | up |
| molecular_function | catalytic activity | sp|P25155|FA10_CHICK | -3.77432 | down |
| molecular_function | catalytic activity | sp|P81475|CFBL_CHICK | -1.75388 | down |
| molecular_function | catalytic activity | sp|Q5F3Z3|UB2V2_CHICK | 2.366484 | up |
| molecular_function | catalytic activity | sp|Q5F480|ITPK1_CHICK | 1.066359 | up |
| molecular_function | catalytic activity | sp|Q5ZHT1|ACD11_CHICK | 1.653324 | up |
| molecular_function | catalytic activity | sp|Q5ZI74|DHX30_CHICK | 1.265146 | up |
| molecular_function | catalytic activity | sp|Q5ZIB9|ANM7_CHICK | 1.168118 | up |
| molecular_function | catalytic activity | sp|Q5ZIF1|APMAP_CHICK | 1.008442 | up |
| molecular_function | catalytic activity | sp|Q5ZIK0|P4K2B_CHICK | 2.036191 | up |
| molecular_function | catalytic activity | sp|Q5ZIP4|XRN2_CHICK | 1.286156 | up |
| molecular_function | catalytic activity | sp|Q5ZJ00|EM55_CHICK | 1.664901 | up |
| molecular_function | catalytic activity | sp|Q5ZJ08|SYYC_CHICK | 1.23041 | up |
| molecular_function | catalytic activity | sp|Q5ZJH6|ULK3_CHICK | 1.593805 | up |
| molecular_function | catalytic activity | sp|Q5ZJH9|DKC1_CHICK | 1.436701 | up |
| molecular_function | catalytic activity | sp|Q5ZJJ8|UBCP1_CHICK | 2.077308 | up |
| molecular_function | catalytic activity | sp|Q5ZJW6|KBRS2_CHICK | 1.256362 | up |
| molecular_function | catalytic activity | sp|Q5ZK92|SPAST_CHICK | 1.665787 | up |
| molecular_function | catalytic activity | sp|Q5ZLV2|PPIL3_CHICK | 1.29867 | up |
| molecular_function | catalytic activity | sp|Q5ZM83|MIRO2_CHICK | 1.659999 | up |
| molecular_function | catalytic activity | sp|Q5ZMG0|DOPD_CHICK | 2.121364 | up |
| molecular_function | catalytic activity | sp|Q804X6|FA9_CHICK | -5.71386 | down |
| molecular_function | catalytic activity | sp|Q8AXL1|SAT1_CHICK | 1.12819 | up |
| molecular_function | catalytic activity | sp|Q98SH2|AT2B1_CHICK | 1.799719 | up |
| molecular_function | catalytic activity | sp|Q9I8D0|VPP1_CHICK | 1.736096 | up |
| molecular_function | catalytic activity | sp|Q9IAM7|MRE11_CHICK | 1.337957 | up |
| molecular_function | catalytic activity | tr|A0A1D5NT70|A0A1D5NT70_CHICK | 1.819215 | up |
| molecular_function | catalytic activity | tr|A0A1D5NTT2|A0A1D5NTT2_CHICK | 2.201769 | up |
| molecular_function | catalytic activity | tr|A0A1D5NU82|A0A1D5NU82_CHICK | 1.12312 | up |
| molecular_function | catalytic activity | tr|A0A1D5NUD3|A0A1D5NUD3_CHICK | 1.402549 | up |
| molecular_function | catalytic activity | tr|A0A1D5NUK5|A0A1D5NUK5_CHICK | 2.534566 | up |
| molecular_function | catalytic activity | tr|A0A1D5NUU0|A0A1D5NUU0_CHICK | 1.000304 | up |
| molecular_function | catalytic activity | tr|A0A1D5NVT0|A0A1D5NVT0_CHICK | 1.421036 | up |
| molecular_function | catalytic activity | tr|A0A1D5NWJ7|A0A1D5NWJ7_CHICK | 1.195763 | up |
| molecular_function | catalytic activity | tr|A0A1D5NXJ2|A0A1D5NXJ2_CHICK | 1.224878 | up |
| molecular_function | catalytic activity | tr|A0A1D5NZB4|A0A1D5NZB4_CHICK | 1.671702 | up |
| molecular_function | catalytic activity | tr|A0A1D5NZG5|A0A1D5NZG5_CHICK | 1.205698 | up |
| molecular_function | catalytic activity | tr|A0A1D5P1D5|A0A1D5P1D5_CHICK | 2.084726 | up |
| molecular_function | catalytic activity | tr|A0A1D5P3Q3|A0A1D5P3Q3_CHICK | 2.685378 | up |
| molecular_function | catalytic activity | tr|A0A1D5P453|A0A1D5P453_CHICK | 1.553695 | up |
| molecular_function | catalytic activity | tr|A0A1D5P5L5|A0A1D5P5L5_CHICK | 2.521962 | up |
| molecular_function | catalytic activity | tr|A0A1D5P5X4|A0A1D5P5X4_CHICK | 1.559711 | up |
| molecular_function | catalytic activity | tr|A0A1D5P6L7|A0A1D5P6L7_CHICK | 1.520092 | up |
| molecular_function | catalytic activity | tr|A0A1D5P6M8|A0A1D5P6M8_CHICK | 1.82889 | up |
| molecular_function | catalytic activity | tr|A0A1D5P6S3|A0A1D5P6S3_CHICK | 1.183172 | up |
| molecular_function | catalytic activity | tr|A0A1D5P6Y6|A0A1D5P6Y6_CHICK | 1.32218 | up |
| molecular_function | catalytic activity | tr|A0A1D5P7C5|A0A1D5P7C5_CHICK | 1.273318 | up |
| molecular_function | catalytic activity | tr|A0A1D5P8P6|A0A1D5P8P6_CHICK | 1.435954 | up |
| molecular_function | catalytic activity | tr|A0A1D5P9G2|A0A1D5P9G2_CHICK | 1.768438 | up |
| molecular_function | catalytic activity | tr|A0A1D5PA08|A0A1D5PA08_CHICK | 1.946165 | up |
| molecular_function | catalytic activity | tr|A0A1D5PAM1|A0A1D5PAM1_CHICK | 2.577291 | up |
| molecular_function | catalytic activity | tr|A0A1D5PBU7|A0A1D5PBU7_CHICK | 2.092246 | up |
| molecular_function | catalytic activity | tr|A0A1D5PC23|A0A1D5PC23_CHICK | 2.344267 | up |
| molecular_function | catalytic activity | tr|A0A1D5PCZ1|A0A1D5PCZ1_CHICK | 1.542638 | up |
| molecular_function | catalytic activity | tr|A0A1D5PFS2|A0A1D5PFS2_CHICK | 1.463999 | up |
| molecular_function | catalytic activity | tr|A0A1D5PFX4|A0A1D5PFX4_CHICK | 1.061266 | up |
| molecular_function | catalytic activity | tr|A0A1D5PG53|A0A1D5PG53_CHICK | 2.149242 | up |
| molecular_function | catalytic activity | tr|A0A1D5PGB1|A0A1D5PGB1_CHICK | 1.43532 | up |
| molecular_function | catalytic activity | tr|A0A1D5PHB6|A0A1D5PHB6_CHICK | 1.553464 | up |
| molecular_function | catalytic activity | tr|A0A1D5PHE2|A0A1D5PHE2_CHICK | 2.577604 | up |
| molecular_function | catalytic activity | tr|A0A1D5PHH6|A0A1D5PHH6_CHICK | -2.20613 | down |
| molecular_function | catalytic activity | tr|A0A1D5PIN1|A0A1D5PIN1_CHICK | 1.417376 | up |
| molecular_function | catalytic activity | tr|A0A1D5PIT4|A0A1D5PIT4_CHICK | -1.97988 | down |
| molecular_function | catalytic activity | tr|A0A1D5PKQ7|A0A1D5PKQ7_CHICK | -1.34664 | down |
| molecular_function | catalytic activity | tr|A0A1D5PPH4|A0A1D5PPH4_CHICK | 1.254155 | up |
| molecular_function | catalytic activity | tr|A0A1D5PTW3|A0A1D5PTW3_CHICK | 1.808599 | up |
| molecular_function | catalytic activity | tr|A0A1D5PWT4|A0A1D5PWT4_CHICK | 1.101328 | up |
| molecular_function | catalytic activity | tr|A0A1D5PXN4|A0A1D5PXN4_CHICK | -1.85723 | down |
| molecular_function | catalytic activity | tr|A0A1D5PYU0|A0A1D5PYU0_CHICK | 1.138573 | up |
| molecular_function | catalytic activity | tr|A0A1D5Q008|A0A1D5Q008_CHICK | 2.962255 | up |
| molecular_function | catalytic activity | tr|A0A1D6UPT6|A0A1D6UPT6_CHICK | 1.170381 | up |
| molecular_function | catalytic activity | tr|A0A1L1RPB1|A0A1L1RPB1_CHICK | 1.864507 | up |
| molecular_function | catalytic activity | tr|A0A1L1RPD1|A0A1L1RPD1_CHICK | 1.396669 | up |
| molecular_function | catalytic activity | tr|A0A1L1RPT9|A0A1L1RPT9_CHICK | 1.353636 | up |
| molecular_function | catalytic activity | tr|A0A1L1RSG1|A0A1L1RSG1_CHICK | 1.889712 | up |
| molecular_function | catalytic activity | tr|A0A1L1RSS5|A0A1L1RSS5_CHICK | 1.143155 | up |
| molecular_function | catalytic activity | tr|A0A1L1RXA3|A0A1L1RXA3_CHICK | 1.394002 | up |
| molecular_function | catalytic activity | tr|A0A286QZR1|A0A286QZR1_CHICK | 1.043669 | up |
| molecular_function | catalytic activity | tr|A0A3Q2TRS4|A0A3Q2TRS4_CHICK | 1.770416 | up |
| molecular_function | catalytic activity | tr|A0A3Q2TS24|A0A3Q2TS24_CHICK | 1.398629 | up |
| molecular_function | catalytic activity | tr|A0A3Q2TY77|A0A3Q2TY77_CHICK | 1.82929 | up |
| molecular_function | catalytic activity | tr|A0A3Q2TYP7|A0A3Q2TYP7_CHICK | 2.159057 | up |
| molecular_function | catalytic activity | tr|A0A3Q2U0V6|A0A3Q2U0V6_CHICK | 1.411673 | up |
| molecular_function | catalytic activity | tr|A0A3Q2U3C4|A0A3Q2U3C4_CHICK | 1.094483 | up |
| molecular_function | catalytic activity | tr|A0A3Q2UBH5|A0A3Q2UBH5_CHICK | 1.115044 | up |
| molecular_function | catalytic activity | tr|A0A3Q2UBJ7|A0A3Q2UBJ7_CHICK | 1.945958 | up |
| molecular_function | catalytic activity | tr|A0A3Q2UCG6|A0A3Q2UCG6_CHICK | 1.033099 | up |
| molecular_function | catalytic activity | tr|A0A3Q2UCN1|A0A3Q2UCN1_CHICK | 1.92272 | up |
| molecular_function | catalytic activity | tr|A0A3Q2UG55|A0A3Q2UG55_CHICK | -2.8915 | down |
| molecular_function | catalytic activity | tr|A0A3Q2UIQ5|A0A3Q2UIQ5_CHICK | 1.516735 | up |
| molecular_function | catalytic activity | tr|A0A3Q2UK74|A0A3Q2UK74_CHICK | 2.254776 | up |
| molecular_function | catalytic activity | tr|A0A3Q3AIE0|A0A3Q3AIE0_CHICK | 1.346321 | up |
| molecular_function | catalytic activity | tr|A0A3Q3ALC8|A0A3Q3ALC8_CHICK | 2.188996 | up |
| molecular_function | catalytic activity | tr|A0A3Q3APG2|A0A3Q3APG2_CHICK | 1.640987 | up |
| molecular_function | catalytic activity | tr|A0A3Q3AQ10|A0A3Q3AQ10_CHICK | 1.000417 | up |
| molecular_function | catalytic activity | tr|A8VIF4|A8VIF4_CHICK | 1.024165 | up |
| molecular_function | catalytic activity | tr|D2D3P4|D2D3P4_CHICK | 2.146381 | up |
| molecular_function | catalytic activity | tr|E1BQI0|E1BQI0_CHICK | 1.067708 | up |
| molecular_function | catalytic activity | tr|E1BQU2|E1BQU2_CHICK | 1.629018 | up |
| molecular_function | catalytic activity | tr|E1BRT3|E1BRT3_CHICK | 2.214419 | up |
| molecular_function | catalytic activity | tr|E1BS94|E1BS94_CHICK | 1.70074 | up |
| molecular_function | catalytic activity | tr|E1BSH7|E1BSH7_CHICK | 1.527514 | up |
| molecular_function | catalytic activity | tr|E1BT44|E1BT44_CHICK | 1.432225 | up |
| molecular_function | catalytic activity | tr|E1BTI7|E1BTI7_CHICK | 1.813027 | up |
| molecular_function | catalytic activity | tr|E1BTT8|E1BTT8_CHICK | 1.708105 | up |
| molecular_function | catalytic activity | tr|E1BU27|E1BU27_CHICK | 3.035848 | up |
| molecular_function | catalytic activity | tr|E1BV97|E1BV97_CHICK | 2.036483 | up |
| molecular_function | catalytic activity | tr|E1BVD1|E1BVD1_CHICK | 3.053645 | up |
| molecular_function | catalytic activity | tr|E1BWG7|E1BWG7_CHICK | 2.421508 | up |
| molecular_function | catalytic activity | tr|E1BX21|E1BX21_CHICK | 1.42634 | up |
| molecular_function | catalytic activity | tr|E1BX85|E1BX85_CHICK | 2.029343 | up |
| molecular_function | catalytic activity | tr|E1BXC2|E1BXC2_CHICK | 1.767478 | up |
| molecular_function | catalytic activity | tr|E1BXT9|E1BXT9_CHICK | 1.600608 | up |
| molecular_function | catalytic activity | tr|E1BY22|E1BY22_CHICK | 1.367672 | up |
| molecular_function | catalytic activity | tr|E1BY40|E1BY40_CHICK | 1.129106 | up |
| molecular_function | catalytic activity | tr|E1BYQ3|E1BYQ3_CHICK | 1.202088 | up |
| molecular_function | catalytic activity | tr|E1BZ74|E1BZ74_CHICK | 1.217761 | up |
| molecular_function | catalytic activity | tr|E1BZ79|E1BZ79_CHICK | 2.388844 | up |
| molecular_function | catalytic activity | tr|E1C1I6|E1C1I6_CHICK | 2.427937 | up |
| molecular_function | catalytic activity | tr|E1C1T2|E1C1T2_CHICK | 1.594659 | up |
| molecular_function | catalytic activity | tr|E1C229|E1C229_CHICK | 1.473036 | up |
| molecular_function | catalytic activity | tr|E1C2E5|E1C2E5_CHICK | 1.182299 | up |
| molecular_function | catalytic activity | tr|E1C312|E1C312_CHICK | 1.811403 | up |
| molecular_function | catalytic activity | tr|E1C688|E1C688_CHICK | 1.775755 | up |
| molecular_function | catalytic activity | tr|E1C7J8|E1C7J8_CHICK | 1.184879 | up |
| molecular_function | catalytic activity | tr|E1C7P7|E1C7P7_CHICK | 2.341246 | up |
| molecular_function | catalytic activity | tr|E1C9H5|E1C9H5_CHICK | 1.195027 | up |
| molecular_function | catalytic activity | tr|F1N832|F1N832_CHICK | 1.427435 | up |
| molecular_function | catalytic activity | tr|F1N8Y3|F1N8Y3_CHICK | 2.225925 | up |
| molecular_function | catalytic activity | tr|F1N977|F1N977_CHICK | 1.720618 | up |
| molecular_function | catalytic activity | tr|F1N9S3|F1N9S3_CHICK | 1.256683 | up |
| molecular_function | catalytic activity | tr|F1NBX1|F1NBX1_CHICK | 1.439614 | up |
| molecular_function | catalytic activity | tr|F1NCM5|F1NCM5_CHICK | 2.577712 | up |
| molecular_function | catalytic activity | tr|F1NE88|F1NE88_CHICK | 1.362851 | up |
| molecular_function | catalytic activity | tr|F1NEQ3|F1NEQ3_CHICK | 1.139952 | up |
| molecular_function | catalytic activity | tr|F1NGU0|F1NGU0_CHICK | 1.010874 | up |
| molecular_function | catalytic activity | tr|F1NLM0|F1NLM0_CHICK | 1.884615 | up |
| molecular_function | catalytic activity | tr|F1NMJ9|F1NMJ9_CHICK | -2.4895 | down |
| molecular_function | catalytic activity | tr|F1NMM9|F1NMM9_CHICK | 1.449446 | up |
| molecular_function | catalytic activity | tr|F1NMU0|F1NMU0_CHICK | 2.467729 | up |
| molecular_function | catalytic activity | tr|F1NN63|F1NN63_CHICK | 1.72615 | up |
| molecular_function | catalytic activity | tr|F1NSB1|F1NSB1_CHICK | 1.692336 | up |
| molecular_function | catalytic activity | tr|F1NSQ4|F1NSQ4_CHICK | 1.21442 | up |
| molecular_function | catalytic activity | tr|F1NT80|F1NT80_CHICK | 1.404176 | up |
| molecular_function | catalytic activity | tr|F1NTQ2|F1NTQ2_CHICK | 1.236275 | up |
| molecular_function | catalytic activity | tr|F1NUY0|F1NUY0_CHICK | 1.570572 | up |
| molecular_function | catalytic activity | tr|F1NUY9|F1NUY9_CHICK | 1.592258 | up |
| molecular_function | catalytic activity | tr|F1NXV6|F1NXV6_CHICK | -4.87705 | down |
| molecular_function | catalytic activity | tr|F1NXZ7|F1NXZ7_CHICK | 1.050248 | up |
| molecular_function | catalytic activity | tr|F1NYG7|F1NYG7_CHICK | 1.537967 | up |
| molecular_function | catalytic activity | tr|F1P099|F1P099_CHICK | 1.399388 | up |
| molecular_function | catalytic activity | tr|F1P204|F1P204_CHICK | 2.129437 | up |
| molecular_function | catalytic activity | tr|F1P278|F1P278_CHICK | 1.88205 | up |
| molecular_function | catalytic activity | tr|F1P372|F1P372_CHICK | 1.525043 | up |
| molecular_function | catalytic activity | tr|F1P394|F1P394_CHICK | 1.286828 | up |
| molecular_function | catalytic activity | tr|F1P4D7|F1P4D7_CHICK | 1.032498 | up |
| molecular_function | catalytic activity | tr|F1P526|F1P526_CHICK | 1.506479 | up |
| molecular_function | catalytic activity | tr|F1P531|F1P531_CHICK | 1.370667 | up |
| molecular_function | catalytic activity | tr|F1P5A3|F1P5A3_CHICK | 2.127136 | up |
| molecular_function | catalytic activity | tr|F1P5J7|F1P5J7_CHICK | 1.075835 | up |
| molecular_function | catalytic activity | tr|F1P5S5|F1P5S5_CHICK | 1.528046 | up |
| molecular_function | catalytic activity | tr|F1P5X6|F1P5X6_CHICK | 1.295882 | up |
| molecular_function | catalytic activity | tr|H9L0M7|H9L0M7_CHICK | 1.164684 | up |
| molecular_function | catalytic activity | tr|O93466|O93466_CHICK | 1.669881 | up |
| molecular_function | catalytic activity | tr|Q2PUH1|Q2PUH1_CHICK | 1.468226 | up |
| molecular_function | catalytic activity | tr|Q5F3R8|Q5F3R8_CHICK | 2.332553 | up |
| molecular_function | catalytic activity | tr|Q5F3X1|Q5F3X1_CHICK | 1.19047 | up |
| molecular_function | catalytic activity | tr|Q5F420|Q5F420_CHICK | 1.387816 | up |
| molecular_function | catalytic activity | tr|Q5ZHP2|Q5ZHP2_CHICK | 2.412922 | up |
| molecular_function | catalytic activity | tr|Q5ZIL3|Q5ZIL3_CHICK | 1.863958 | up |
| molecular_function | catalytic activity | tr|Q5ZJ96|Q5ZJ96_CHICK | 1.306532 | up |
| molecular_function | catalytic activity | tr|Q5ZJQ4|Q5ZJQ4_CHICK | 1.678561 | up |
| molecular_function | catalytic activity | tr|Q5ZJW2|Q5ZJW2_CHICK | 1.71086 | up |
| molecular_function | catalytic activity | tr|Q5ZKI3|Q5ZKI3_CHICK | 1.326993 | up |
| molecular_function | catalytic activity | tr|Q5ZMA7|Q5ZMA7_CHICK | 1.172026 | up |
| molecular_function | catalytic activity | tr|Q5ZMG8|Q5ZMG8_CHICK | 1.771296 | up |
| molecular_function | catalytic activity | tr|Q7ZTS9|Q7ZTS9_CHICK | 1.923559 | up |
| molecular_function | catalytic activity | tr|Q90W83|Q90W83_CHICK | 2.57878 | up |
| molecular_function | catalytic activity | tr|Q9I882|Q9I882_CHICK | 2.21964 | up |
| molecular_function | catalytic activity | tr|Q9I8C8|Q9I8C8_CHICK | 1.2712 | up |
| molecular_function | catalytic activity | tr|R4GG24|R4GG24_CHICK | 3.018466 | up |
| molecular_function | catalytic activity | tr|R4GHY3|R4GHY3_CHICK | 1.375649 | up |
| molecular_function | catalytic activity | tr|R4GI86|R4GI86_CHICK | 2.078653 | up |
| molecular_function | catalytic activity | tr|R4GJH2|R4GJH2_CHICK | 1.058464 | up |
| molecular_function | catalytic activity | tr|R4GMH5|R4GMH5_CHICK | -2.32193 | down |
| molecular_function | catalytic activity | tr|V9GW30|V9GW30_CHICK | 1.560922 | up |
| molecular_function | molecular carrier activity | sp|P02197|MYG_CHICK | 2.885059 | up |
| molecular_function | molecular carrier activity | sp|Q5ZLT0|XPO7_CHICK | 1.469766 | up |
| molecular_function | molecular carrier activity | tr|A0A1D5P8H7|A0A1D5P8H7_CHICK | 1.530513 | up |
| molecular_function | molecular carrier activity | tr|Q5QRU7|Q5QRU7_CHICK | 2.017795 | up |
| molecular_function | molecular function regulator | sp|P02659|APOV1_CHICK | -11.5091 | down |
| molecular_function | molecular function regulator | tr|A0A1D5NUV0|A0A1D5NUV0_CHICK | 2.157037 | up |
| molecular_function | molecular function regulator | tr|A0A1D5PK72|A0A1D5PK72_CHICK | 2.032922 | up |
| molecular_function | molecular function regulator | tr|A0A1D5PU94|A0A1D5PU94_CHICK | -1.37297 | down |
| molecular_function | molecular function regulator | tr|A0A1D5PZ78|A0A1D5PZ78_CHICK | 1.936124 | up |
| molecular_function | molecular function regulator | tr|A0A3Q2TTT6|A0A3Q2TTT6_CHICK | 1.855872 | up |
| molecular_function | molecular function regulator | tr|A0A3Q2TZM7|A0A3Q2TZM7_CHICK | 1.419267 | up |
| molecular_function | molecular function regulator | tr|B3VE14|B3VE14_CHICK | -1.63112 | down |
| molecular_function | molecular function regulator | tr|E1BTE2|E1BTE2_CHICK | -2.67874 | down |
| molecular_function | molecular function regulator | tr|E1BUX5|E1BUX5_CHICK | 1.099122 | up |
| molecular_function | molecular function regulator | tr|E1BVQ3|E1BVQ3_CHICK | 1.650579 | up |
| molecular_function | molecular function regulator | tr|E1C592|E1C592_CHICK | 1.565005 | up |
| molecular_function | molecular function regulator | tr|E1C7H6|E1C7H6_CHICK | -1.05537 | down |
| molecular_function | molecular function regulator | tr|E7EC82|E7EC82_CHICK | 1.586027 | up |
| molecular_function | molecular function regulator | tr|F1N8V5|F1N8V5_CHICK | 2.344853 | up |
| molecular_function | molecular function regulator | tr|F1NBX1|F1NBX1_CHICK | 1.439614 | up |
| molecular_function | molecular function regulator | tr|F1NQ61|F1NQ61_CHICK | 1.080306 | up |
| molecular_function | molecular function regulator | tr|F1NQP5|F1NQP5_CHICK | 2.898371 | up |
| molecular_function | molecular function regulator | tr|F1NSI7|F1NSI7_CHICK | 1.77407 | up |
| molecular_function | molecular function regulator | tr|I1SV11|I1SV11_CHICK | 1.313667 | up |
| molecular_function | molecular function regulator | tr|Q5ZJQ4|Q5ZJQ4_CHICK | 1.678561 | up |
| molecular_function | molecular function regulator | tr|Q5ZMP2|Q5ZMP2_CHICK | 1.165624 | up |
| molecular_function | molecular function regulator | tr|R4GI86|R4GI86_CHICK | 2.078653 | up |
| molecular_function | molecular transducer activity | sp|Q90733|COT2_CHICK | 1.595685 | up |
| molecular_function | molecular transducer activity | tr|A0A1D5NZB4|A0A1D5NZB4_CHICK | 1.671702 | up |
| molecular_function | molecular transducer activity | tr|A0A1D5P453|A0A1D5P453_CHICK | 1.553695 | up |
| molecular_function | molecular transducer activity | tr|A0A1D5PIB1|A0A1D5PIB1_CHICK | 2.10824 | up |
| molecular_function | molecular transducer activity | tr|A0A1L1RWP3|A0A1L1RWP3_CHICK | -4.54587 | down |
| molecular_function | molecular transducer activity | tr|A0A1L1S0P1|A0A1L1S0P1_CHICK | -2.55147 | down |
| molecular_function | molecular transducer activity | tr|A0A3Q2UCN1|A0A3Q2UCN1_CHICK | 1.92272 | up |
| molecular_function | molecular transducer activity | tr|E1BRR6|E1BRR6_CHICK | 1.332531 | up |
| molecular_function | molecular transducer activity | tr|E1BTI7|E1BTI7_CHICK | 1.813027 | up |
| molecular_function | molecular transducer activity | tr|E1C688|E1C688_CHICK | 1.775755 | up |
| molecular_function | molecular transducer activity | tr|E1C7A7|E1C7A7_CHICK | -1.02532 | down |
| molecular_function | molecular transducer activity | tr|F1NXV6|F1NXV6_CHICK | -4.87705 | down |
| molecular_function | molecular transducer activity | tr|Q2PUH1|Q2PUH1_CHICK | 1.468226 | up |
| molecular_function | molecular transducer activity | tr|Q5ZL65|Q5ZL65_CHICK | 1.695268 | up |
| molecular_function | nutrient reservoir activity | sp|P02659|APOV1_CHICK | -11.5091 | down |
| molecular_function | nutrient reservoir activity | sp|P02845|VIT2_CHICK | -11.7249 | down |
| molecular_function | nutrient reservoir activity | tr|F1NFL6|F1NFL6_CHICK | -11.6512 | down |
| molecular_function | signal transducer activity | sp|Q90733|COT2_CHICK | 1.595685 | up |
| molecular_function | signal transducer activity | tr|A0A1D5NZB4|A0A1D5NZB4_CHICK | 1.671702 | up |
| molecular_function | signal transducer activity | tr|A0A1D5P453|A0A1D5P453_CHICK | 1.553695 | up |
| molecular_function | signal transducer activity | tr|A0A1D5PIB1|A0A1D5PIB1_CHICK | 2.10824 | up |
| molecular_function | signal transducer activity | tr|A0A3Q2UCN1|A0A3Q2UCN1_CHICK | 1.92272 | up |
| molecular_function | signal transducer activity | tr|A0A3Q2UM13|A0A3Q2UM13_CHICK | 3.560203 | up |
| molecular_function | signal transducer activity | tr|E1C3U6|E1C3U6_CHICK | 1.144268 | up |
| molecular_function | signal transducer activity | tr|E1C688|E1C688_CHICK | 1.775755 | up |
| molecular_function | signal transducer activity | tr|F1NXV6|F1NXV6_CHICK | -4.87705 | down |
| molecular_function | signal transducer activity | tr|F6YX81|F6YX81_CHICK | 1.194552 | up |
| molecular_function | signal transducer activity | tr|Q5ZL65|Q5ZL65_CHICK | 1.695268 | up |
| molecular_function | structural molecule activity | tr|A0A1D5P607|A0A1D5P607_CHICK | 1.51904 | up |
| molecular_function | structural molecule activity | tr|A0A1D5P6C6|A0A1D5P6C6_CHICK | 1.782047 | up |
| molecular_function | structural molecule activity | tr|A0A1D5P7E3|A0A1D5P7E3_CHICK | 1.017643 | up |
| molecular_function | structural molecule activity | tr|A0A1D5PF37|A0A1D5PF37_CHICK | 1.238601 | up |
| molecular_function | structural molecule activity | tr|A0A1D5PQG6|A0A1D5PQG6_CHICK | 2.091788 | up |
| molecular_function | structural molecule activity | tr|A0A3Q2UD87|A0A3Q2UD87_CHICK | 1.205498 | up |
| molecular_function | structural molecule activity | tr|E1BW27|E1BW27_CHICK | 1.938051 | up |
| molecular_function | structural molecule activity | tr|E1C1Y5|E1C1Y5_CHICK | 1.135922 | up |
| molecular_function | structural molecule activity | tr|F1NEA1|F1NEA1_CHICK | 1.395721 | up |
| molecular_function | structural molecule activity | tr|Q5ZKY5|Q5ZKY5_CHICK | 1.620297 | up |
| molecular_function | transcription regulator activity | sp|O42395|CNBP_CHICK | 1.365125 | up |
| molecular_function | transcription regulator activity | sp|O93602|ATF2_CHICK | 2.071233 | up |
| molecular_function | transcription regulator activity | sp|P17923|NFIA_CHICK | 1.346459 | up |
| molecular_function | transcription regulator activity | sp|Q90733|COT2_CHICK | 1.595685 | up |
| molecular_function | transcription regulator activity | tr|A0A1D5NZS7|A0A1D5NZS7_CHICK | 1.185694 | up |
| molecular_function | transcription regulator activity | tr|A0A1D5PIB1|A0A1D5PIB1_CHICK | 2.10824 | up |
| molecular_function | transcription regulator activity | tr|A0A1D5PRI4|A0A1D5PRI4_CHICK | 1.28486 | up |
| molecular_function | transcription regulator activity | tr|A0A3Q2TVU0|A0A3Q2TVU0_CHICK | 1.280793 | up |
| molecular_function | transcription regulator activity | tr|E1BX21|E1BX21_CHICK | 1.42634 | up |
| molecular_function | transcription regulator activity | tr|E1C3U6|E1C3U6_CHICK | 1.144268 | up |
| molecular_function | transcription regulator activity | tr|E1C6S7|E1C6S7_CHICK | 1.1895 | up |
| molecular_function | transcription regulator activity | tr|E7EC82|E7EC82_CHICK | 1.586027 | up |
| molecular_function | transcription regulator activity | tr|F1N9U0|F1N9U0_CHICK | 1.038918 | up |
| molecular_function | transcription regulator activity | tr|F1N9Y3|F1N9Y3_CHICK | 1.33841 | up |
| molecular_function | transcription regulator activity | tr|F1NSF2|F1NSF2_CHICK | 1.708711 | up |
| molecular_function | transcription regulator activity | tr|I1SV11|I1SV11_CHICK | 1.313667 | up |
| molecular_function | transcription regulator activity | tr|Q5DWF6|Q5DWF6_CHICK | 1.289716 | up |
| molecular_function | transcription regulator activity | tr|Q5ZLI0|Q5ZLI0_CHICK | 1.097879 | up |
| molecular_function | transporter activity | sp|P02845|VIT2_CHICK | -11.7249 | down |
| molecular_function | transporter activity | sp|Q05423|FABP7_CHICK | 1.9874 | up |
| molecular_function | transporter activity | sp|Q5ZJB7|CHMP7_CHICK | 1.815716 | up |
| molecular_function | transporter activity | sp|Q98SH2|AT2B1_CHICK | 1.799719 | up |
| molecular_function | transporter activity | sp|Q9I8D0|VPP1_CHICK | 1.736096 | up |
| molecular_function | transporter activity | tr|A0A1D5NVT0|A0A1D5NVT0_CHICK | 1.421036 | up |
| molecular_function | transporter activity | tr|A0A1D5NWJ7|A0A1D5NWJ7_CHICK | 1.195763 | up |
| molecular_function | transporter activity | tr|A0A1D5P453|A0A1D5P453_CHICK | 1.553695 | up |
| molecular_function | transporter activity | tr|A0A1D5PIT4|A0A1D5PIT4_CHICK | -1.97988 | down |
| molecular_function | transporter activity | tr|A0A1L1RSG1|A0A1L1RSG1_CHICK | 1.889712 | up |
| molecular_function | transporter activity | tr|A0A3Q2TUF8|A0A3Q2TUF8_CHICK | 1.02023 | up |
| molecular_function | transporter activity | tr|A0A3Q2TVV0|A0A3Q2TVV0_CHICK | 1.145618 | up |
| molecular_function | transporter activity | tr|A0A3Q2TYP7|A0A3Q2TYP7_CHICK | 2.159057 | up |
| molecular_function | transporter activity | tr|A0A3Q2U067|A0A3Q2U067_CHICK | 1.508572 | up |
| molecular_function | transporter activity | tr|A0A3Q2UD87|A0A3Q2UD87_CHICK | 1.205498 | up |
| molecular_function | transporter activity | tr|A0A3Q2UM13|A0A3Q2UM13_CHICK | 3.560203 | up |
| molecular_function | transporter activity | tr|E1BXY8|E1BXY8_CHICK | 1.106549 | up |
| molecular_function | transporter activity | tr|E1BY40|E1BY40_CHICK | 1.129106 | up |
| molecular_function | transporter activity | tr|E1C8U1|E1C8U1_CHICK | 1.411305 | up |
| molecular_function | transporter activity | tr|F1NFL6|F1NFL6_CHICK | -11.6512 | down |
| molecular_function | transporter activity | tr|F1NMU0|F1NMU0_CHICK | 2.467729 | up |
| molecular_function | transporter activity | tr|F1P151|F1P151_CHICK | 2.603153 | up |
| molecular_function | transporter activity | tr|F1P204|F1P204_CHICK | 2.129437 | up |
| molecular_function | transporter activity | tr|Q197X2|Q197X2_CHICK | -9.95256 | down |
| molecular_function | transporter activity | tr|Q5ZJ19|Q5ZJ19_CHICK | 2.470646 | up |
| molecular_function | transporter activity | tr|Q5ZMJ6|Q5ZMJ6_CHICK | 1.845871 | up |
| molecular_function | transporter activity | tr|Q9I8C8|Q9I8C8_CHICK | 1.2712 | up |

**Supplementary Table 2.** Up or down regulation of differential proteins in KEGG pathway classification.

| Pathway_level1 | Pathway_level2 | Protein_ID | log2  Foldchange | Up/  Down |
| --- | --- | --- | --- | --- |
| Cellular Processes | Cell growth and death | sp|P02789|TRFE_CHICK | -2.2371 | down |
| Cellular Processes | Cell growth and death | sp|Q5ZJB7|CHMP7_CHICK | 1.815716 | up |
| Cellular Processes | Cell growth and death | sp|Q8AXL1|SAT1_CHICK | 1.12819 | up |
| Cellular Processes | Cell growth and death | sp|Q9IAM7|MRE11_CHICK | 1.337957 | up |
| Cellular Processes | Cell growth and death | tr|A0A1D5NYD8|A0A1D5NYD8_CHICK | 1.246912 | up |
| Cellular Processes | Cell growth and death | tr|A0A1D5NZB4|A0A1D5NZB4_CHICK | 1.671702 | up |
| Cellular Processes | Cell growth and death | tr|A0A1D5NZW0|A0A1D5NZW0_CHICK | 1.956911 | up |
| Cellular Processes | Cell growth and death | tr|A0A1D5P6C6|A0A1D5P6C6_CHICK | 1.782047 | up |
| Cellular Processes | Cell growth and death | tr|A0A1D5P8V8|A0A1D5P8V8_CHICK | 1.608897 | up |
| Cellular Processes | Cell growth and death | tr|A0A1D5PA08|A0A1D5PA08_CHICK | 1.946165 | up |
| Cellular Processes | Cell growth and death | tr|A0A1D5PAM1|A0A1D5PAM1_CHICK | 2.577291 | up |
| Cellular Processes | Cell growth and death | tr|A0A1D5PB71|A0A1D5PB71_CHICK | 1.051487 | up |
| Cellular Processes | Cell growth and death | tr|A0A1D5PM87|A0A1D5PM87_CHICK | 1.108975 | up |
| Cellular Processes | Cell growth and death | tr|A0A1D5PZ78|A0A1D5PZ78_CHICK | 1.936124 | up |
| Cellular Processes | Cell growth and death | tr|A0A1L1RIY5|A0A1L1RIY5_CHICK | 751.4932 | up |
| Cellular Processes | Cell growth and death | tr|A0A1L1RTJ6|A0A1L1RTJ6_CHICK | 1.2568 | up |
| Cellular Processes | Cell growth and death | tr|A0A3Q2UBR5|A0A3Q2UBR5_CHICK | 1.516208 | up |
| Cellular Processes | Cell growth and death | tr|A0A3Q3AIE0|A0A3Q3AIE0_CHICK | 1.346321 | up |
| Cellular Processes | Cell growth and death | tr|A0A3S5ZPH2|A0A3S5ZPH2_CHICK | 1.677715 | up |
| Cellular Processes | Cell growth and death | tr|E1BQC2|E1BQC2_CHICK | -2.52084 | down |
| Cellular Processes | Cell growth and death | tr|E1BQW2|E1BQW2_CHICK | 1.823966 | up |
| Cellular Processes | Cell growth and death | tr|E1BSH7|E1BSH7_CHICK | 1.527514 | up |
| Cellular Processes | Cell growth and death | tr|E1BTE2|E1BTE2_CHICK | -2.67874 | down |
| Cellular Processes | Cell growth and death | tr|E1BTI7|E1BTI7_CHICK | 1.813027 | up |
| Cellular Processes | Cell growth and death | tr|F1NMJ9|F1NMJ9_CHICK | -2.4895 | down |
| Cellular Processes | Cell growth and death | tr|F1NPS5|F1NPS5_CHICK | 3.319197 | up |
| Cellular Processes | Cell growth and death | tr|F1NSQ4|F1NSQ4_CHICK | 1.21442 | up |
| Cellular Processes | Cell growth and death | tr|F1NT58|F1NT58_CHICK | 1.701877 | up |
| Cellular Processes | Cell growth and death | tr|O42417|O42417_CHICK | 1.118629 | up |
| Cellular Processes | Cell growth and death | tr|Q4ADJ6|Q4ADJ6_CHICK | -4.04825 | down |
| Cellular Processes | Cell growth and death | tr|Q5F420|Q5F420_CHICK | 1.387816 | up |
| Cellular Processes | Cell growth and death | tr|Q5ZMJ6|Q5ZMJ6_CHICK | 1.845871 | up |
| Cellular Processes | Cell motility | sp|P00523|SRC_CHICK | 1.599352 | up |
| Cellular Processes | Cell motility | tr|A0A1D5NZB4|A0A1D5NZB4_CHICK | 1.671702 | up |
| Cellular Processes | Cell motility | tr|A0A3Q3AN52|A0A3Q3AN52_CHICK | 1.965147 | up |
| Cellular Processes | Cell motility | tr|E1BTH9|E1BTH9_CHICK | 1.623876 | up |
| Cellular Processes | Cell motility | tr|F1NXV6|F1NXV6_CHICK | -4.87705 | down |
| Cellular Processes | Cell motility | tr|O93466|O93466_CHICK | 1.669881 | up |
| Cellular Processes | Cellular community - eukaryotes | sp|P00523|SRC_CHICK | 1.599352 | up |
| Cellular Processes | Cellular community - eukaryotes | tr|A0A1D5NZB4|A0A1D5NZB4_CHICK | 1.671702 | up |
| Cellular Processes | Cellular community - eukaryotes | tr|A0A1D5P6C6|A0A1D5P6C6_CHICK | 1.782047 | up |
| Cellular Processes | Cellular community - eukaryotes | tr|A0A1D5P8P6|A0A1D5P8P6_CHICK | 1.435954 | up |
| Cellular Processes | Cellular community - eukaryotes | tr|A0A1D5PCZ1|A0A1D5PCZ1_CHICK | 1.542638 | up |
| Cellular Processes | Cellular community - eukaryotes | tr|A0A1D5PQC1|A0A1D5PQC1_CHICK | 1.667257 | up |
| Cellular Processes | Cellular community - eukaryotes | tr|A0A1L1RTJ6|A0A1L1RTJ6_CHICK | 1.2568 | up |
| Cellular Processes | Cellular community - eukaryotes | tr|A0A3Q2U7Q5|A0A3Q2U7Q5_CHICK | 1.254495 | up |
| Cellular Processes | Cellular community - eukaryotes | tr|A0A3Q2UCN1|A0A3Q2UCN1_CHICK | 1.92272 | up |
| Cellular Processes | Cellular community - eukaryotes | tr|A0A3Q2UPF3|A0A3Q2UPF3_CHICK | 1.295517 | up |
| Cellular Processes | Cellular community - eukaryotes | tr|A0A3Q3AN52|A0A3Q3AN52_CHICK | 1.965147 | up |
| Cellular Processes | Cellular community - eukaryotes | tr|E1BR45|E1BR45_CHICK | 1.323412 | up |
| Cellular Processes | Cellular community - eukaryotes | tr|E1BTH9|E1BTH9_CHICK | 1.623876 | up |
| Cellular Processes | Cellular community - eukaryotes | tr|E1C296|E1C296_CHICK | 1.621433 | up |
| Cellular Processes | Cellular community - eukaryotes | tr|E1C7A7|E1C7A7_CHICK | -1.02532 | down |
| Cellular Processes | Cellular community - eukaryotes | tr|F1NJ25|F1NJ25_CHICK | 1.444652 | up |
| Cellular Processes | Cellular community - eukaryotes | tr|F1NMJ9|F1NMJ9_CHICK | -2.4895 | down |
| Cellular Processes | Cellular community - eukaryotes | tr|F1NZZ2|F1NZZ2_CHICK | 1.657798 | up |
| Cellular Processes | Cellular community - eukaryotes | tr|O93466|O93466_CHICK | 1.669881 | up |
| Cellular Processes | Cellular community - eukaryotes | tr|Q2PUH1|Q2PUH1_CHICK | 1.468226 | up |
| Cellular Processes | Cellular community - eukaryotes | tr|Q5F3X1|Q5F3X1_CHICK | 1.19047 | up |
| Cellular Processes | Transport and catabolism | sp|O93436|STAM2_CHICK | 2.376336 | up |
| Cellular Processes | Transport and catabolism | sp|P00523|SRC_CHICK | 1.599352 | up |
| Cellular Processes | Transport and catabolism | sp|Q5ZHN3|WIPI2_CHICK | 1.61465 | up |
| Cellular Processes | Transport and catabolism | sp|Q5ZJB7|CHMP7_CHICK | 1.815716 | up |
| Cellular Processes | Transport and catabolism | sp|Q5ZL36|ZFY27_CHICK | 1.948763 | up |
| Cellular Processes | Transport and catabolism | sp|Q5ZM83|MIRO2_CHICK | 1.659999 | up |
| Cellular Processes | Transport and catabolism | sp|Q9I8D0|VPP1_CHICK | 1.736096 | up |
| Cellular Processes | Transport and catabolism | tr|A0A1D5NTC8|A0A1D5NTC8_CHICK | 1.184237 | up |
| Cellular Processes | Transport and catabolism | tr|A0A1D5NU19|A0A1D5NU19_CHICK | 1.251921 | up |
| Cellular Processes | Transport and catabolism | tr|A0A1D5NU78|A0A1D5NU78_CHICK | 1.139159 | up |
| Cellular Processes | Transport and catabolism | tr|A0A1D5NUU0|A0A1D5NUU0_CHICK | 1.000304 | up |
| Cellular Processes | Transport and catabolism | tr|A0A1D5NYD8|A0A1D5NYD8_CHICK | 1.246912 | up |
| Cellular Processes | Transport and catabolism | tr|A0A1D5NZB4|A0A1D5NZB4_CHICK | 1.671702 | up |
| Cellular Processes | Transport and catabolism | tr|A0A1D5P2W5|A0A1D5P2W5_CHICK | 1.407792 | up |
| Cellular Processes | Transport and catabolism | tr|A0A1D5P6C6|A0A1D5P6C6_CHICK | 1.782047 | up |
| Cellular Processes | Transport and catabolism | tr|A0A1D5P6H2|A0A1D5P6H2_CHICK | 1.371328 | up |
| Cellular Processes | Transport and catabolism | tr|A0A1D5P8V8|A0A1D5P8V8_CHICK | 1.608897 | up |
| Cellular Processes | Transport and catabolism | tr|A0A1D5P9F9|A0A1D5P9F9_CHICK | -1.61299 | down |
| Cellular Processes | Transport and catabolism | tr|A0A1D5PAQ0|A0A1D5PAQ0_CHICK | -4.05766 | down |
| Cellular Processes | Transport and catabolism | tr|A0A1D5PEU0|A0A1D5PEU0_CHICK | 1.077518 | up |
| Cellular Processes | Transport and catabolism | tr|A0A1D5PFS2|A0A1D5PFS2_CHICK | 1.463999 | up |
| Cellular Processes | Transport and catabolism | tr|A0A1D5PHV9|A0A1D5PHV9_CHICK | 1.32812 | up |
| Cellular Processes | Transport and catabolism | tr|A0A1D5PMH0|A0A1D5PMH0_CHICK | 1.197966 | up |
| Cellular Processes | Transport and catabolism | tr|A0A1D5PXK6|A0A1D5PXK6_CHICK | 1.402857 | up |
| Cellular Processes | Transport and catabolism | tr|A0A1D5PXN4|A0A1D5PXN4_CHICK | -1.85723 | down |
| Cellular Processes | Transport and catabolism | tr|A0A1D5PYU0|A0A1D5PYU0_CHICK | 1.138573 | up |
| Cellular Processes | Transport and catabolism | tr|A0A1L1RJB1|A0A1L1RJB1_CHICK | 1.578007 | up |
| Cellular Processes | Transport and catabolism | tr|A0A3Q2TRS4|A0A3Q2TRS4_CHICK | 1.770416 | up |
| Cellular Processes | Transport and catabolism | tr|A0A3Q2TTN1|A0A3Q2TTN1_CHICK | -3.87735 | down |
| Cellular Processes | Transport and catabolism | tr|A0A3Q2TX54|A0A3Q2TX54_CHICK | -4.4331 | down |
| Cellular Processes | Transport and catabolism | tr|A0A3Q2TYP7|A0A3Q2TYP7_CHICK | 2.159057 | up |
| Cellular Processes | Transport and catabolism | tr|A0A3Q2TYZ3|A0A3Q2TYZ3_CHICK | -3.88695 | down |
| Cellular Processes | Transport and catabolism | tr|A0A3Q2U474|A0A3Q2U474_CHICK | -3.66327 | down |
| Cellular Processes | Transport and catabolism | tr|A0A3Q2U637|A0A3Q2U637_CHICK | -2.432 | down |
| Cellular Processes | Transport and catabolism | tr|A0A3Q2UAA5|A0A3Q2UAA5_CHICK | -3.02904 | down |
| Cellular Processes | Transport and catabolism | tr|A0A3Q2UAE7|A0A3Q2UAE7_CHICK | -3.38259 | down |
| Cellular Processes | Transport and catabolism | tr|A0A3Q2UED4|A0A3Q2UED4_CHICK | 1.445759 | up |
| Cellular Processes | Transport and catabolism | tr|A0A3Q2UEH0|A0A3Q2UEH0_CHICK | 1.684594 | up |
| Cellular Processes | Transport and catabolism | tr|A0A3Q2UFX5|A0A3Q2UFX5_CHICK | -3.47542 | down |
| Cellular Processes | Transport and catabolism | tr|A0A3Q2UHW8|A0A3Q2UHW8_CHICK | -4.15396 | down |
| Cellular Processes | Transport and catabolism | tr|A0A3Q2UJK8|A0A3Q2UJK8_CHICK | -3.86779 | down |
| Cellular Processes | Transport and catabolism | tr|A0A3Q3AB84|A0A3Q3AB84_CHICK | 1.170717 | up |
| Cellular Processes | Transport and catabolism | tr|A0A3Q3ABE7|A0A3Q3ABE7_CHICK | 1.137168 | up |
| Cellular Processes | Transport and catabolism | tr|A0A3Q3ALP4|A0A3Q3ALP4_CHICK | 1.886964 | up |
| Cellular Processes | Transport and catabolism | tr|A0A3Q3AMX7|A0A3Q3AMX7_CHICK | -3.6569 | down |
| Cellular Processes | Transport and catabolism | tr|E1BRR6|E1BRR6_CHICK | 1.332531 | up |
| Cellular Processes | Transport and catabolism | tr|E1BSH7|E1BSH7_CHICK | 1.527514 | up |
| Cellular Processes | Transport and catabolism | tr|E1BT44|E1BT44_CHICK | 1.432225 | up |
| Cellular Processes | Transport and catabolism | tr|E1BTH9|E1BTH9_CHICK | 1.623876 | up |
| Cellular Processes | Transport and catabolism | tr|E1BTI7|E1BTI7_CHICK | 1.813027 | up |
| Cellular Processes | Transport and catabolism | tr|E1BVK8|E1BVK8_CHICK | 1.186519 | up |
| Cellular Processes | Transport and catabolism | tr|E1BVQ3|E1BVQ3_CHICK | 1.650579 | up |
| Cellular Processes | Transport and catabolism | tr|E1BXS1|E1BXS1_CHICK | 1.514054 | up |
| Cellular Processes | Transport and catabolism | tr|E1BZ79|E1BZ79_CHICK | 2.388844 | up |
| Cellular Processes | Transport and catabolism | tr|E1C2E5|E1C2E5_CHICK | 1.182299 | up |
| Cellular Processes | Transport and catabolism | tr|E1C396|E1C396_CHICK | 1.08258 | up |
| Cellular Processes | Transport and catabolism | tr|F1N977|F1N977_CHICK | 1.720618 | up |
| Cellular Processes | Transport and catabolism | tr|F1NBY5|F1NBY5_CHICK | 1.813459 | up |
| Cellular Processes | Transport and catabolism | tr|F1NNV6|F1NNV6_CHICK | 1.809432 | up |
| Cellular Processes | Transport and catabolism | tr|F1NPS5|F1NPS5_CHICK | 3.319197 | up |
| Cellular Processes | Transport and catabolism | tr|F1NSQ4|F1NSQ4_CHICK | 1.21442 | up |
| Cellular Processes | Transport and catabolism | tr|F1NTQ2|F1NTQ2_CHICK | 1.236275 | up |
| Cellular Processes | Transport and catabolism | tr|F1NYG7|F1NYG7_CHICK | 1.537967 | up |
| Cellular Processes | Transport and catabolism | tr|F1P099|F1P099_CHICK | 1.399388 | up |
| Cellular Processes | Transport and catabolism | tr|F1P151|F1P151_CHICK | 2.603153 | up |
| Cellular Processes | Transport and catabolism | tr|F1P5B8|F1P5B8_CHICK | 1.715588 | up |
| Cellular Processes | Transport and catabolism | tr|F1P5J7|F1P5J7_CHICK | 1.075835 | up |
| Cellular Processes | Transport and catabolism | tr|Q2PUH1|Q2PUH1_CHICK | 1.468226 | up |
| Cellular Processes | Transport and catabolism | tr|Q5F3R8|Q5F3R8_CHICK | 2.332553 | up |
| Cellular Processes | Transport and catabolism | tr|Q5F420|Q5F420_CHICK | 1.387816 | up |
| Cellular Processes | Transport and catabolism | tr|Q5ZJ19|Q5ZJ19_CHICK | 2.470646 | up |
| Cellular Processes | Transport and catabolism | tr|Q5ZMP2|Q5ZMP2_CHICK | 1.165624 | up |
| Cellular Processes | Transport and catabolism | tr|Q9I8C8|Q9I8C8_CHICK | 1.2712 | up |
| Cellular Processes | Transport and catabolism | tr|R4GHH2|R4GHH2_CHICK | 1.778292 | up |
| Environmental Information Processing | Membrane transport | tr|A0A1D5NVT0|A0A1D5NVT0_CHICK | 1.421036 | up |
| Environmental Information Processing | Membrane transport | tr|A0A1D5P453|A0A1D5P453_CHICK | 1.553695 | up |
| Environmental Information Processing | Membrane transport | tr|A0A3Q2UP33|A0A3Q2UP33_CHICK | 2.057435 | up |
| Environmental Information Processing | Membrane transport | tr|E1BY40|E1BY40_CHICK | 1.129106 | up |
| Environmental Information Processing | Membrane transport | tr|F1P204|F1P204_CHICK | 2.129437 | up |
| Environmental Information Processing | Signal transduction | sp|O93436|STAM2_CHICK | 2.376336 | up |
| Environmental Information Processing | Signal transduction | sp|O93602|ATF2_CHICK | 2.071233 | up |
| Environmental Information Processing | Signal transduction | sp|P00523|SRC_CHICK | 1.599352 | up |
| Environmental Information Processing | Signal transduction | sp|P02789|TRFE_CHICK | -2.2371 | down |
| Environmental Information Processing | Signal transduction | sp|P52162|MAX_CHICK | 1.334512 | up |
| Environmental Information Processing | Signal transduction | sp|Q5F480|ITPK1_CHICK | 1.066359 | up |
| Environmental Information Processing | Signal transduction | sp|Q5ZIK0|P4K2B_CHICK | 2.036191 | up |
| Environmental Information Processing | Signal transduction | sp|Q98SH2|AT2B1_CHICK | 1.799719 | up |
| Environmental Information Processing | Signal transduction | tr|A0A1D5NTC8|A0A1D5NTC8_CHICK | 1.184237 | up |
| Environmental Information Processing | Signal transduction | tr|A0A1D5NU12|A0A1D5NU12_CHICK | 1.463781 | up |
| Environmental Information Processing | Signal transduction | tr|A0A1D5NUD3|A0A1D5NUD3_CHICK | 1.402549 | up |
| Environmental Information Processing | Signal transduction | tr|A0A1D5NUU0|A0A1D5NUU0_CHICK | 1.000304 | up |
| Environmental Information Processing | Signal transduction | tr|A0A1D5NV81|A0A1D5NV81_CHICK | 1.935393 | up |
| Environmental Information Processing | Signal transduction | tr|A0A1D5NZB4|A0A1D5NZB4_CHICK | 1.671702 | up |
| Environmental Information Processing | Signal transduction | tr|A0A1D5P6C3|A0A1D5P6C3_CHICK | 2.100747 | up |
| Environmental Information Processing | Signal transduction | tr|A0A1D5P6C6|A0A1D5P6C6_CHICK | 1.782047 | up |
| Environmental Information Processing | Signal transduction | tr|A0A1D5P8H7|A0A1D5P8H7_CHICK | 1.530513 | up |
| Environmental Information Processing | Signal transduction | tr|A0A1D5P8P6|A0A1D5P8P6_CHICK | 1.435954 | up |
| Environmental Information Processing | Signal transduction | tr|A0A1D5PA08|A0A1D5PA08_CHICK | 1.946165 | up |
| Environmental Information Processing | Signal transduction | tr|A0A1D5PAQ0|A0A1D5PAQ0_CHICK | -4.05766 | down |
| Environmental Information Processing | Signal transduction | tr|A0A1D5PCZ1|A0A1D5PCZ1_CHICK | 1.542638 | up |
| Environmental Information Processing | Signal transduction | tr|A0A1D5PD17|A0A1D5PD17_CHICK | 1.860269 | up |
| Environmental Information Processing | Signal transduction | tr|A0A1D5PDW6|A0A1D5PDW6_CHICK | 1.010784 | up |
| Environmental Information Processing | Signal transduction | tr|A0A1D5PFS2|A0A1D5PFS2_CHICK | 1.463999 | up |
| Environmental Information Processing | Signal transduction | tr|A0A1D5PHE2|A0A1D5PHE2_CHICK | 2.577604 | up |
| Environmental Information Processing | Signal transduction | tr|A0A1D5PJL7|A0A1D5PJL7_CHICK | 1.22028 | up |
| Environmental Information Processing | Signal transduction | tr|A0A1D5PQC1|A0A1D5PQC1_CHICK | 1.667257 | up |
| Environmental Information Processing | Signal transduction | tr|A0A1D5PWW9|A0A1D5PWW9_CHICK | 1.972994 | up |
| Environmental Information Processing | Signal transduction | tr|A0A1D5PYB7|A0A1D5PYB7_CHICK | 1.489522 | up |
| Environmental Information Processing | Signal transduction | tr|A0A1L1RTJ6|A0A1L1RTJ6_CHICK | 1.2568 | up |
| Environmental Information Processing | Signal transduction | tr|A0A1L1RWI9|A0A1L1RWI9_CHICK | 1.991255 | up |
| Environmental Information Processing | Signal transduction | tr|A0A1L1RXZ8|A0A1L1RXZ8_CHICK | 2.28751 | up |
| Environmental Information Processing | Signal transduction | tr|A0A1L1RZ25|A0A1L1RZ25_CHICK | 1.75764 | up |
| Environmental Information Processing | Signal transduction | tr|A0A286QZR1|A0A286QZR1_CHICK | 1.043669 | up |
| Environmental Information Processing | Signal transduction | tr|A0A3Q2TRS4|A0A3Q2TRS4_CHICK | 1.770416 | up |
| Environmental Information Processing | Signal transduction | tr|A0A3Q2TS24|A0A3Q2TS24_CHICK | 1.398629 | up |
| Environmental Information Processing | Signal transduction | tr|A0A3Q2TTN1|A0A3Q2TTN1_CHICK | -3.87735 | down |
| Environmental Information Processing | Signal transduction | tr|A0A3Q2TUF8|A0A3Q2TUF8_CHICK | 1.02023 | up |
| Environmental Information Processing | Signal transduction | tr|A0A3Q2TX28|A0A3Q2TX28_CHICK | 1.495625 | up |
| Environmental Information Processing | Signal transduction | tr|A0A3Q2TX54|A0A3Q2TX54_CHICK | -4.4331 | down |
| Environmental Information Processing | Signal transduction | tr|A0A3Q2TYZ3|A0A3Q2TYZ3_CHICK | -3.88695 | down |
| Environmental Information Processing | Signal transduction | tr|A0A3Q2U0I3|A0A3Q2U0I3_CHICK | 1.647665 | up |
| Environmental Information Processing | Signal transduction | tr|A0A3Q2U0V6|A0A3Q2U0V6_CHICK | 1.411673 | up |
| Environmental Information Processing | Signal transduction | tr|A0A3Q2U3K8|A0A3Q2U3K8_CHICK | 1.162767 | up |
| Environmental Information Processing | Signal transduction | tr|A0A3Q2U474|A0A3Q2U474_CHICK | -3.66327 | down |
| Environmental Information Processing | Signal transduction | tr|A0A3Q2U637|A0A3Q2U637_CHICK | -2.432 | down |
| Environmental Information Processing | Signal transduction | tr|A0A3Q2UAA5|A0A3Q2UAA5_CHICK | -3.02904 | down |
| Environmental Information Processing | Signal transduction | tr|A0A3Q2UAE7|A0A3Q2UAE7_CHICK | -3.38259 | down |
| Environmental Information Processing | Signal transduction | tr|A0A3Q2UBR5|A0A3Q2UBR5_CHICK | 1.516208 | up |
| Environmental Information Processing | Signal transduction | tr|A0A3Q2UCN1|A0A3Q2UCN1_CHICK | 1.92272 | up |
| Environmental Information Processing | Signal transduction | tr|A0A3Q2UFX5|A0A3Q2UFX5_CHICK | -3.47542 | down |
| Environmental Information Processing | Signal transduction | tr|A0A3Q2UGM5|A0A3Q2UGM5_CHICK | 1.351371 | up |
| Environmental Information Processing | Signal transduction | tr|A0A3Q2UHW8|A0A3Q2UHW8_CHICK | -4.15396 | down |
| Environmental Information Processing | Signal transduction | tr|A0A3Q2UJK8|A0A3Q2UJK8_CHICK | -3.86779 | down |
| Environmental Information Processing | Signal transduction | tr|A0A3Q3ALC8|A0A3Q3ALC8_CHICK | 2.188996 | up |
| Environmental Information Processing | Signal transduction | tr|A0A3Q3AMX7|A0A3Q3AMX7_CHICK | -3.6569 | down |
| Environmental Information Processing | Signal transduction | tr|A0A3Q3AN52|A0A3Q3AN52_CHICK | 1.965147 | up |
| Environmental Information Processing | Signal transduction | tr|A0A3Q3APG2|A0A3Q3APG2_CHICK | 1.640987 | up |
| Environmental Information Processing | Signal transduction | tr|A0A3Q3B0J7|A0A3Q3B0J7_CHICK | 1.055674 | up |
| Environmental Information Processing | Signal transduction | tr|A0A5P9VLA0|A0A5P9VLA0_CHICK | 1.601588 | up |
| Environmental Information Processing | Signal transduction | tr|E1BQC2|E1BQC2_CHICK | -2.52084 | down |
| Environmental Information Processing | Signal transduction | tr|E1BQI0|E1BQI0_CHICK | 1.067708 | up |
| Environmental Information Processing | Signal transduction | tr|E1BR45|E1BR45_CHICK | 1.323412 | up |
| Environmental Information Processing | Signal transduction | tr|E1BS74|E1BS74_CHICK | 3.418615 | up |
| Environmental Information Processing | Signal transduction | tr|E1BTF7|E1BTF7_CHICK | 1.810838 | up |
| Environmental Information Processing | Signal transduction | tr|E1BTQ4|E1BTQ4_CHICK | -4.65695 | down |
| Environmental Information Processing | Signal transduction | tr|E1BTW4|E1BTW4_CHICK | 2.41163 | up |
| Environmental Information Processing | Signal transduction | tr|E1BYQ3|E1BYQ3_CHICK | 1.202088 | up |
| Environmental Information Processing | Signal transduction | tr|E1C172|E1C172_CHICK | 1.05688 | up |
| Environmental Information Processing | Signal transduction | tr|E1C296|E1C296_CHICK | 1.621433 | up |
| Environmental Information Processing | Signal transduction | tr|E1C592|E1C592_CHICK | 1.565005 | up |
| Environmental Information Processing | Signal transduction | tr|E1C7A7|E1C7A7_CHICK | -1.02532 | down |
| Environmental Information Processing | Signal transduction | tr|E1C7H6|E1C7H6_CHICK | -1.05537 | down |
| Environmental Information Processing | Signal transduction | tr|E7EC82|E7EC82_CHICK | 1.586027 | up |
| Environmental Information Processing | Signal transduction | tr|F1N9Y3|F1N9Y3_CHICK | 1.33841 | up |
| Environmental Information Processing | Signal transduction | tr|F1NC81|F1NC81_CHICK | 1.284264 | up |
| Environmental Information Processing | Signal transduction | tr|F1NDH2|F1NDH2_CHICK | -1.95135 | down |
| Environmental Information Processing | Signal transduction | tr|F1NMJ9|F1NMJ9_CHICK | -2.4895 | down |
| Environmental Information Processing | Signal transduction | tr|F1NS24|F1NS24_CHICK | 1.16955 | up |
| Environmental Information Processing | Signal transduction | tr|F1NXV6|F1NXV6_CHICK | -4.87705 | down |
| Environmental Information Processing | Signal transduction | tr|F1NZZ2|F1NZZ2_CHICK | 1.657798 | up |
| Environmental Information Processing | Signal transduction | tr|F1P204|F1P204_CHICK | 2.129437 | up |
| Environmental Information Processing | Signal transduction | tr|O93466|O93466_CHICK | 1.669881 | up |
| Environmental Information Processing | Signal transduction | tr|Q2PUH1|Q2PUH1_CHICK | 1.468226 | up |
| Environmental Information Processing | Signal transduction | tr|Q4ADJ6|Q4ADJ6_CHICK | -4.04825 | down |
| Environmental Information Processing | Signal transduction | tr|Q5F3R8|Q5F3R8_CHICK | 2.332553 | up |
| Environmental Information Processing | Signal transduction | tr|Q5F3X1|Q5F3X1_CHICK | 1.19047 | up |
| Environmental Information Processing | Signal transduction | tr|Q5ZJ96|Q5ZJ96_CHICK | 1.306532 | up |
| Environmental Information Processing | Signal transduction | tr|Q5ZJQ4|Q5ZJQ4_CHICK | 1.678561 | up |
| Environmental Information Processing | Signal transduction | tr|Q5ZMJ6|Q5ZMJ6_CHICK | 1.845871 | up |
| Environmental Information Processing | Signal transduction | tr|R4GI86|R4GI86_CHICK | 2.078653 | up |
| Environmental Information Processing | Signaling molecules and interaction | sp|P10184|IOV7_CHICK | -2.4214 | down |
| Environmental Information Processing | Signaling molecules and interaction | sp|P42292|CD166_CHICK | 1.297212 | up |
| Environmental Information Processing | Signaling molecules and interaction | tr|A0A1D5P7U1|A0A1D5P7U1_CHICK | 1.721535 | up |
| Environmental Information Processing | Signaling molecules and interaction | tr|A0A1D5P9F9|A0A1D5P9F9_CHICK | -1.61299 | down |
| Environmental Information Processing | Signaling molecules and interaction | tr|A0A1D5PIB1|A0A1D5PIB1_CHICK | 2.10824 | up |
| Environmental Information Processing | Signaling molecules and interaction | tr|A0A3Q2UJ85|A0A3Q2UJ85_CHICK | -2.5117 | down |
| Environmental Information Processing | Signaling molecules and interaction | tr|A0A3Q3AN52|A0A3Q3AN52_CHICK | 1.965147 | up |
| Environmental Information Processing | Signaling molecules and interaction | tr|E1C592|E1C592_CHICK | 1.565005 | up |
| Environmental Information Processing | Signaling molecules and interaction | tr|E1C7A7|E1C7A7_CHICK | -1.02532 | down |
| Environmental Information Processing | Signaling molecules and interaction | tr|F1NC81|F1NC81_CHICK | 1.284264 | up |
| Environmental Information Processing | Signaling molecules and interaction | tr|F1NDH2|F1NDH2_CHICK | -1.95135 | down |
| Environmental Information Processing | Signaling molecules and interaction | tr|F1NXV6|F1NXV6_CHICK | -4.87705 | down |
| Environmental Information Processing | Signaling molecules and interaction | tr|F1NZZ2|F1NZZ2_CHICK | 1.657798 | up |
| Environmental Information Processing | Signaling molecules and interaction | tr|Q5ZL65|Q5ZL65_CHICK | 1.695268 | up |
| Environmental Information Processing | Signaling molecules and interaction | tr|R4GMH5|R4GMH5_CHICK | -2.32193 | down |
| Genetic Information Processing | Folding, sorting and degradation | sp|P41366|VMO1_CHICK | -5.73258 | down |
| Genetic Information Processing | Folding, sorting and degradation | sp|Q5ZIP4|XRN2_CHICK | 1.286156 | up |
| Genetic Information Processing | Folding, sorting and degradation | tr|A0A1D5NXG4|A0A1D5NXG4_CHICK | 1.245324 | up |
| Genetic Information Processing | Folding, sorting and degradation | tr|A0A1D5NXR2|A0A1D5NXR2_CHICK | 1.37138 | up |
| Genetic Information Processing | Folding, sorting and degradation | tr|A0A1D5P1D5|A0A1D5P1D5_CHICK | 2.084726 | up |
| Genetic Information Processing | Folding, sorting and degradation | tr|A0A1D5P1Q3|A0A1D5P1Q3_CHICK | 1.314659 | up |
| Genetic Information Processing | Folding, sorting and degradation | tr|A0A1D5P6H2|A0A1D5P6H2_CHICK | 1.371328 | up |
| Genetic Information Processing | Folding, sorting and degradation | tr|A0A1D5P6L7|A0A1D5P6L7_CHICK | 1.520092 | up |
| Genetic Information Processing | Folding, sorting and degradation | tr|A0A1D5PB71|A0A1D5PB71_CHICK | 1.051487 | up |
| Genetic Information Processing | Folding, sorting and degradation | tr|A0A1D5PHB6|A0A1D5PHB6_CHICK | 1.553464 | up |
| Genetic Information Processing | Folding, sorting and degradation | tr|A0A1D5PY43|A0A1D5PY43_CHICK | 2.581091 | up |
| Genetic Information Processing | Folding, sorting and degradation | tr|A0A1D5PZ78|A0A1D5PZ78_CHICK | 1.936124 | up |
| Genetic Information Processing | Folding, sorting and degradation | tr|A0A3Q2TX69|A0A3Q2TX69_CHICK | 1.419939 | up |
| Genetic Information Processing | Folding, sorting and degradation | tr|A0A3Q2UA24|A0A3Q2UA24_CHICK | 1.718411 | up |
| Genetic Information Processing | Folding, sorting and degradation | tr|A0A3Q2UAS1|A0A3Q2UAS1_CHICK | 1.28598 | up |
| Genetic Information Processing | Folding, sorting and degradation | tr|A0A3Q2UEW0|A0A3Q2UEW0_CHICK | 3.637252 | up |
| Genetic Information Processing | Folding, sorting and degradation | tr|A0A3Q2UFU4|A0A3Q2UFU4_CHICK | 1.101062 | up |
| Genetic Information Processing | Folding, sorting and degradation | tr|E1BUX5|E1BUX5_CHICK | 1.099122 | up |
| Genetic Information Processing | Folding, sorting and degradation | tr|E1BYQ3|E1BYQ3_CHICK | 1.202088 | up |
| Genetic Information Processing | Folding, sorting and degradation | tr|E1C310|E1C310_CHICK | 1.545191 | up |
| Genetic Information Processing | Folding, sorting and degradation | tr|F1ND07|F1ND07_CHICK | -1.08324 | down |
| Genetic Information Processing | Folding, sorting and degradation | tr|F1NJI6|F1NJI6_CHICK | 1.561914 | up |
| Genetic Information Processing | Folding, sorting and degradation | tr|F1NL19|F1NL19_CHICK | 1.203656 | up |
| Genetic Information Processing | Folding, sorting and degradation | tr|F1NQ61|F1NQ61_CHICK | 1.080306 | up |
| Genetic Information Processing | Folding, sorting and degradation | tr|F6YX81|F6YX81_CHICK | 1.194552 | up |
| Genetic Information Processing | Folding, sorting and degradation | tr|Q5ZIL3|Q5ZIL3_CHICK | 1.863958 | up |
| Genetic Information Processing | Folding, sorting and degradation | tr|Q5ZKI3|Q5ZKI3_CHICK | 1.326993 | up |
| Genetic Information Processing | Folding, sorting and degradation | tr|Q5ZMP2|Q5ZMP2_CHICK | 1.165624 | up |
| Genetic Information Processing | Folding, sorting and degradation | tr|V9GW30|V9GW30_CHICK | 1.560922 | up |
| Genetic Information Processing | Replication and repair | sp|Q5F3K4|WDR48_CHICK | 1.71564 | up |
| Genetic Information Processing | Replication and repair | sp|Q5ZML0|BABA2_CHICK | 1.144731 | up |
| Genetic Information Processing | Replication and repair | sp|Q9IAM7|MRE11_CHICK | 1.337957 | up |
| Genetic Information Processing | Replication and repair | tr|A0A1D5NY53|A0A1D5NY53_CHICK | 1.357776 | up |
| Genetic Information Processing | Replication and repair | tr|A0A3Q3AR14|A0A3Q3AR14_CHICK | 1.164862 | up |
| Genetic Information Processing | Replication and repair | tr|F1NLM0|F1NLM0_CHICK | 1.884615 | up |
| Genetic Information Processing | Replication and repair | tr|F1P394|F1P394_CHICK | 1.286828 | up |
| Genetic Information Processing | Transcription | sp|Q5ZJX4|RBM38_CHICK | 1.802689 | up |
| Genetic Information Processing | Transcription | tr|A0A1D5NV31|A0A1D5NV31_CHICK | 1.324369 | up |
| Genetic Information Processing | Transcription | tr|A0A1D5P6C3|A0A1D5P6C3_CHICK | 2.100747 | up |
| Genetic Information Processing | Transcription | tr|A0A1D5P7C5|A0A1D5P7C5_CHICK | 1.273318 | up |
| Genetic Information Processing | Transcription | tr|A0A3Q2TVU0|A0A3Q2TVU0_CHICK | 1.280793 | up |
| Genetic Information Processing | Transcription | tr|A0A3Q2UCQ1|A0A3Q2UCQ1_CHICK | 1.349205 | up |
| Genetic Information Processing | Transcription | tr|E1BUJ1|E1BUJ1_CHICK | 2.709557 | up |
| Genetic Information Processing | Transcription | tr|E1BV97|E1BV97_CHICK | 2.036483 | up |
| Genetic Information Processing | Transcription | tr|F1N9U0|F1N9U0_CHICK | 1.038918 | up |
| Genetic Information Processing | Transcription | tr|F1NPB8|F1NPB8_CHICK | 1.816074 | up |
| Genetic Information Processing | Transcription | tr|Q5ZLI0|Q5ZLI0_CHICK | 1.097879 | up |
| Genetic Information Processing | Translation | sp|Q5ZIP4|XRN2_CHICK | 1.286156 | up |
| Genetic Information Processing | Translation | sp|Q5ZJ08|SYYC_CHICK | 1.23041 | up |
| Genetic Information Processing | Translation | sp|Q5ZJ66|SYEM_CHICK | 1.236071 | up |
| Genetic Information Processing | Translation | sp|Q5ZJH9|DKC1_CHICK | 1.436701 | up |
| Genetic Information Processing | Translation | sp|Q5ZJX4|RBM38_CHICK | 1.802689 | up |
| Genetic Information Processing | Translation | tr|A0A1D5P330|A0A1D5P330_CHICK | 1.716147 | up |
| Genetic Information Processing | Translation | tr|A0A1D5P607|A0A1D5P607_CHICK | 1.51904 | up |
| Genetic Information Processing | Translation | tr|A0A1D5P6C6|A0A1D5P6C6_CHICK | 1.782047 | up |
| Genetic Information Processing | Translation | tr|A0A1D5P6Y6|A0A1D5P6Y6_CHICK | 1.32218 | up |
| Genetic Information Processing | Translation | tr|A0A1D5P7W0|A0A1D5P7W0_CHICK | 1.07962 | up |
| Genetic Information Processing | Translation | tr|A0A1D5P8H7|A0A1D5P8H7_CHICK | 1.530513 | up |
| Genetic Information Processing | Translation | tr|A0A1D5P9B9|A0A1D5P9B9_CHICK | 1.081114 | up |
| Genetic Information Processing | Translation | tr|A0A1D5PF91|A0A1D5PF91_CHICK | 1.455018 | up |
| Genetic Information Processing | Translation | tr|A0A1D5PHS9|A0A1D5PHS9_CHICK | 1.645384 | up |
| Genetic Information Processing | Translation | tr|A0A1D5PMR8|A0A1D5PMR8_CHICK | 1.213883 | up |
| Genetic Information Processing | Translation | tr|A0A1D5PUQ9|A0A1D5PUQ9_CHICK | 1.131018 | up |
| Genetic Information Processing | Translation | tr|A0A1D5PXQ1|A0A1D5PXQ1_CHICK | 1.358575 | up |
| Genetic Information Processing | Translation | tr|A0A1L1RL50|A0A1L1RL50_CHICK | 1.173616 | up |
| Genetic Information Processing | Translation | tr|A0A3Q2UCQ1|A0A3Q2UCQ1_CHICK | 1.349205 | up |
| Genetic Information Processing | Translation | tr|A0A3Q2UD87|A0A3Q2UD87_CHICK | 1.205498 | up |
| Genetic Information Processing | Translation | tr|E1C1Y5|E1C1Y5_CHICK | 1.135922 | up |
| Genetic Information Processing | Translation | tr|E1C229|E1C229_CHICK | 1.473036 | up |
| Genetic Information Processing | Translation | tr|E1C2F2|E1C2F2_CHICK | 1.546061 | up |
| Genetic Information Processing | Translation | tr|E1C593|E1C593_CHICK | 1.922793 | up |
| Genetic Information Processing | Translation | tr|E1C8U1|E1C8U1_CHICK | 1.411305 | up |
| Genetic Information Processing | Translation | tr|F1N832|F1N832_CHICK | 1.427435 | up |
| Genetic Information Processing | Translation | tr|F1N9H4|F1N9H4_CHICK | 1.764159 | up |
| Genetic Information Processing | Translation | tr|F1NDV3|F1NDV3_CHICK | 1.048737 | up |
| Genetic Information Processing | Translation | tr|F1NF19|F1NF19_CHICK | 1.471105 | up |
| Genetic Information Processing | Translation | tr|F1NQ46|F1NQ46_CHICK | 1.21543 | up |
| Genetic Information Processing | Translation | tr|F1NQY0|F1NQY0_CHICK | 1.686257 | up |
| Genetic Information Processing | Translation | tr|F1P137|F1P137_CHICK | 1.449133 | up |
| Genetic Information Processing | Translation | tr|F1P372|F1P372_CHICK | 1.525043 | up |
| Genetic Information Processing | Translation | tr|F1P4A7|F1P4A7_CHICK | 2.008749 | up |
| Genetic Information Processing | Translation | tr|Q5ZJA8|Q5ZJA8_CHICK | 1.093474 | up |
| Human Diseases | Cancers: Overview | sp|O93602|ATF2_CHICK | 2.071233 | up |
| Human Diseases | Cancers: Overview | sp|P00523|SRC_CHICK | 1.599352 | up |
| Human Diseases | Cancers: Overview | sp|P20136|GSTM2_CHICK | 2.226935 | up |
| Human Diseases | Cancers: Overview | sp|P52162|MAX_CHICK | 1.334512 | up |
| Human Diseases | Cancers: Overview | tr|A0A1D5NT70|A0A1D5NT70_CHICK | 1.819215 | up |
| Human Diseases | Cancers: Overview | tr|A0A1D5NTR1|A0A1D5NTR1_CHICK | 1.213885 | up |
| Human Diseases | Cancers: Overview | tr|A0A1D5NZB4|A0A1D5NZB4_CHICK | 1.671702 | up |
| Human Diseases | Cancers: Overview | tr|A0A1D5P2R0|A0A1D5P2R0_CHICK | 1.394929 | up |
| Human Diseases | Cancers: Overview | tr|A0A1D5P470|A0A1D5P470_CHICK | 2.193185 | up |
| Human Diseases | Cancers: Overview | tr|A0A1D5P6C6|A0A1D5P6C6_CHICK | 1.782047 | up |
| Human Diseases | Cancers: Overview | tr|A0A1D5P9F9|A0A1D5P9F9_CHICK | -1.61299 | down |
| Human Diseases | Cancers: Overview | tr|A0A1D5PAQ0|A0A1D5PAQ0_CHICK | -4.05766 | down |
| Human Diseases | Cancers: Overview | tr|A0A1D5PCZ1|A0A1D5PCZ1_CHICK | 1.542638 | up |
| Human Diseases | Cancers: Overview | tr|A0A1L1RTJ6|A0A1L1RTJ6_CHICK | 1.2568 | up |
| Human Diseases | Cancers: Overview | tr|A0A2Z5EM90|A0A2Z5EM90_CHICK | 1.136685 | up |
| Human Diseases | Cancers: Overview | tr|A0A3Q2TTN1|A0A3Q2TTN1_CHICK | -3.87735 | down |
| Human Diseases | Cancers: Overview | tr|A0A3Q2TWS4|A0A3Q2TWS4_CHICK | 1.99415 | up |
| Human Diseases | Cancers: Overview | tr|A0A3Q2TX28|A0A3Q2TX28_CHICK | 1.495625 | up |
| Human Diseases | Cancers: Overview | tr|A0A3Q2TX54|A0A3Q2TX54_CHICK | -4.4331 | down |
| Human Diseases | Cancers: Overview | tr|A0A3Q2TYZ3|A0A3Q2TYZ3_CHICK | -3.88695 | down |
| Human Diseases | Cancers: Overview | tr|A0A3Q2U0V6|A0A3Q2U0V6_CHICK | 1.411673 | up |
| Human Diseases | Cancers: Overview | tr|A0A3Q2U474|A0A3Q2U474_CHICK | -3.66327 | down |
| Human Diseases | Cancers: Overview | tr|A0A3Q2U637|A0A3Q2U637_CHICK | -2.432 | down |
| Human Diseases | Cancers: Overview | tr|A0A3Q2U6V5|A0A3Q2U6V5_CHICK | 3.448077 | up |
| Human Diseases | Cancers: Overview | tr|A0A3Q2UAA5|A0A3Q2UAA5_CHICK | -3.02904 | down |
| Human Diseases | Cancers: Overview | tr|A0A3Q2UAE7|A0A3Q2UAE7_CHICK | -3.38259 | down |
| Human Diseases | Cancers: Overview | tr|A0A3Q2UAS1|A0A3Q2UAS1_CHICK | 1.28598 | up |
| Human Diseases | Cancers: Overview | tr|A0A3Q2UBR5|A0A3Q2UBR5_CHICK | 1.516208 | up |
| Human Diseases | Cancers: Overview | tr|A0A3Q2UCN1|A0A3Q2UCN1_CHICK | 1.92272 | up |
| Human Diseases | Cancers: Overview | tr|A0A3Q2UFX5|A0A3Q2UFX5_CHICK | -3.47542 | down |
| Human Diseases | Cancers: Overview | tr|A0A3Q2UHW8|A0A3Q2UHW8_CHICK | -4.15396 | down |
| Human Diseases | Cancers: Overview | tr|A0A3Q2UJK8|A0A3Q2UJK8_CHICK | -3.86779 | down |
| Human Diseases | Cancers: Overview | tr|A0A3Q2UPF3|A0A3Q2UPF3_CHICK | 1.295517 | up |
| Human Diseases | Cancers: Overview | tr|A0A3Q3AMX7|A0A3Q3AMX7_CHICK | -3.6569 | down |
| Human Diseases | Cancers: Overview | tr|A0A3Q3AN52|A0A3Q3AN52_CHICK | 1.965147 | up |
| Human Diseases | Cancers: Overview | tr|E1BTE2|E1BTE2_CHICK | -2.67874 | down |
| Human Diseases | Cancers: Overview | tr|E1BTH9|E1BTH9_CHICK | 1.623876 | up |
| Human Diseases | Cancers: Overview | tr|E1BTQ4|E1BTQ4_CHICK | -4.65695 | down |
| Human Diseases | Cancers: Overview | tr|E1BX21|E1BX21_CHICK | 1.42634 | up |
| Human Diseases | Cancers: Overview | tr|E1BX85|E1BX85_CHICK | 2.029343 | up |
| Human Diseases | Cancers: Overview | tr|E1C688|E1C688_CHICK | 1.775755 | up |
| Human Diseases | Cancers: Overview | tr|E1C764|E1C764_CHICK | 1.131745 | up |
| Human Diseases | Cancers: Overview | tr|E1C7A7|E1C7A7_CHICK | -1.02532 | down |
| Human Diseases | Cancers: Overview | tr|F1N8Y3|F1N8Y3_CHICK | 2.225925 | up |
| Human Diseases | Cancers: Overview | tr|F1NDH2|F1NDH2_CHICK | -1.95135 | down |
| Human Diseases | Cancers: Overview | tr|F1NIY3|F1NIY3_CHICK | 1.568578 | up |
| Human Diseases | Cancers: Overview | tr|F1NJ25|F1NJ25_CHICK | 1.444652 | up |
| Human Diseases | Cancers: Overview | tr|F1NMJ9|F1NMJ9_CHICK | -2.4895 | down |
| Human Diseases | Cancers: Overview | tr|F1NXV6|F1NXV6_CHICK | -4.87705 | down |
| Human Diseases | Cancers: Overview | tr|F1NZZ2|F1NZZ2_CHICK | 1.657798 | up |
| Human Diseases | Cancers: Overview | tr|F1P204|F1P204_CHICK | 2.129437 | up |
| Human Diseases | Cancers: Overview | tr|O93466|O93466_CHICK | 1.669881 | up |
| Human Diseases | Cancers: Overview | tr|Q5F3X1|Q5F3X1_CHICK | 1.19047 | up |
| Human Diseases | Cancers: Specific types | sp|P00523|SRC_CHICK | 1.599352 | up |
| Human Diseases | Cancers: Specific types | sp|P20136|GSTM2_CHICK | 2.226935 | up |
| Human Diseases | Cancers: Specific types | sp|P52162|MAX_CHICK | 1.334512 | up |
| Human Diseases | Cancers: Specific types | tr|A0A1D5NT70|A0A1D5NT70_CHICK | 1.819215 | up |
| Human Diseases | Cancers: Specific types | tr|A0A1D5NTR1|A0A1D5NTR1_CHICK | 1.213885 | up |
| Human Diseases | Cancers: Specific types | tr|A0A1D5NZB4|A0A1D5NZB4_CHICK | 1.671702 | up |
| Human Diseases | Cancers: Specific types | tr|A0A1D5P470|A0A1D5P470_CHICK | 2.193185 | up |
| Human Diseases | Cancers: Specific types | tr|A0A1D5PCZ1|A0A1D5PCZ1_CHICK | 1.542638 | up |
| Human Diseases | Cancers: Specific types | tr|A0A1L1RWI9|A0A1L1RWI9_CHICK | 1.991255 | up |
| Human Diseases | Cancers: Specific types | tr|A0A3Q2TX28|A0A3Q2TX28_CHICK | 1.495625 | up |
| Human Diseases | Cancers: Specific types | tr|A0A3Q2U0V6|A0A3Q2U0V6_CHICK | 1.411673 | up |
| Human Diseases | Cancers: Specific types | tr|A0A3Q2U6V5|A0A3Q2U6V5_CHICK | 3.448077 | up |
| Human Diseases | Cancers: Specific types | tr|A0A3Q2UBR5|A0A3Q2UBR5_CHICK | 1.516208 | up |
| Human Diseases | Cancers: Specific types | tr|A0A3Q3AN52|A0A3Q3AN52_CHICK | 1.965147 | up |
| Human Diseases | Cancers: Specific types | tr|E1BTQ4|E1BTQ4_CHICK | -4.65695 | down |
| Human Diseases | Cancers: Specific types | tr|E1BX85|E1BX85_CHICK | 2.029343 | up |
| Human Diseases | Cancers: Specific types | tr|F1NMJ9|F1NMJ9_CHICK | -2.4895 | down |
| Human Diseases | Cancers: Specific types | tr|F1NZZ2|F1NZZ2_CHICK | 1.657798 | up |
| Human Diseases | Cancers: Specific types | tr|O93466|O93466_CHICK | 1.669881 | up |
| Human Diseases | Cancers: Specific types | tr|Q5F3X1|Q5F3X1_CHICK | 1.19047 | up |
| Human Diseases | Cardiovascular diseases | sp|P00523|SRC_CHICK | 1.599352 | up |
| Human Diseases | Cardiovascular diseases | sp|P20136|GSTM2_CHICK | 2.226935 | up |
| Human Diseases | Cardiovascular diseases | tr|A0A1D5NT70|A0A1D5NT70_CHICK | 1.819215 | up |
| Human Diseases | Cardiovascular diseases | tr|A0A1D5P470|A0A1D5P470_CHICK | 2.193185 | up |
| Human Diseases | Cardiovascular diseases | tr|A0A1D5P8P6|A0A1D5P8P6_CHICK | 1.435954 | up |
| Human Diseases | Cardiovascular diseases | tr|A0A1D5PAQ0|A0A1D5PAQ0_CHICK | -4.05766 | down |
| Human Diseases | Cardiovascular diseases | tr|A0A1D5PWT4|A0A1D5PWT4_CHICK | 1.101328 | up |
| Human Diseases | Cardiovascular diseases | tr|A0A1L1RTJ6|A0A1L1RTJ6_CHICK | 1.2568 | up |
| Human Diseases | Cardiovascular diseases | tr|A0A3Q2TTN1|A0A3Q2TTN1_CHICK | -3.87735 | down |
| Human Diseases | Cardiovascular diseases | tr|A0A3Q2TUF8|A0A3Q2TUF8_CHICK | 1.02023 | up |
| Human Diseases | Cardiovascular diseases | tr|A0A3Q2TX54|A0A3Q2TX54_CHICK | -4.4331 | down |
| Human Diseases | Cardiovascular diseases | tr|A0A3Q2TYZ3|A0A3Q2TYZ3_CHICK | -3.88695 | down |
| Human Diseases | Cardiovascular diseases | tr|A0A3Q2U474|A0A3Q2U474_CHICK | -3.66327 | down |
| Human Diseases | Cardiovascular diseases | tr|A0A3Q2U637|A0A3Q2U637_CHICK | -2.432 | down |
| Human Diseases | Cardiovascular diseases | tr|A0A3Q2UAA5|A0A3Q2UAA5_CHICK | -3.02904 | down |
| Human Diseases | Cardiovascular diseases | tr|A0A3Q2UAE7|A0A3Q2UAE7_CHICK | -3.38259 | down |
| Human Diseases | Cardiovascular diseases | tr|A0A3Q2UBR5|A0A3Q2UBR5_CHICK | 1.516208 | up |
| Human Diseases | Cardiovascular diseases | tr|A0A3Q2UFX5|A0A3Q2UFX5_CHICK | -3.47542 | down |
| Human Diseases | Cardiovascular diseases | tr|A0A3Q2UHW8|A0A3Q2UHW8_CHICK | -4.15396 | down |
| Human Diseases | Cardiovascular diseases | tr|A0A3Q2UJK8|A0A3Q2UJK8_CHICK | -3.86779 | down |
| Human Diseases | Cardiovascular diseases | tr|A0A3Q3AMX7|A0A3Q3AMX7_CHICK | -3.6569 | down |
| Human Diseases | Cardiovascular diseases | tr|A0A3Q3AN52|A0A3Q3AN52_CHICK | 1.965147 | up |
| Human Diseases | Cardiovascular diseases | tr|E1BWI0|E1BWI0_CHICK | 1.213764 | up |
| Human Diseases | Cardiovascular diseases | tr|E1BX85|E1BX85_CHICK | 2.029343 | up |
| Human Diseases | Cardiovascular diseases | tr|F1NDH2|F1NDH2_CHICK | -1.95135 | down |
| Human Diseases | Cardiovascular diseases | tr|F1P4A7|F1P4A7_CHICK | 2.008749 | up |
| Human Diseases | Cardiovascular diseases | tr|O93466|O93466_CHICK | 1.669881 | up |
| Human Diseases | Cardiovascular diseases | tr|Q2PUH1|Q2PUH1_CHICK | 1.468226 | up |
| Human Diseases | Drug resistance: Antineoplastic | sp|P00523|SRC_CHICK | 1.599352 | up |
| Human Diseases | Drug resistance: Antineoplastic | sp|P20136|GSTM2_CHICK | 2.226935 | up |
| Human Diseases | Drug resistance: Antineoplastic | tr|A0A1D5NT70|A0A1D5NT70_CHICK | 1.819215 | up |
| Human Diseases | Drug resistance: Antineoplastic | tr|A0A1D5NVT0|A0A1D5NVT0_CHICK | 1.421036 | up |
| Human Diseases | Drug resistance: Antineoplastic | tr|A0A1D5NZB4|A0A1D5NZB4_CHICK | 1.671702 | up |
| Human Diseases | Drug resistance: Antineoplastic | tr|A0A1D5P470|A0A1D5P470_CHICK | 2.193185 | up |
| Human Diseases | Drug resistance: Antineoplastic | tr|A0A1D5PCZ1|A0A1D5PCZ1_CHICK | 1.542638 | up |
| Human Diseases | Drug resistance: Antineoplastic | tr|A0A1L1RTJ6|A0A1L1RTJ6_CHICK | 1.2568 | up |
| Human Diseases | Drug resistance: Antineoplastic | tr|A0A3Q2TX28|A0A3Q2TX28_CHICK | 1.495625 | up |
| Human Diseases | Drug resistance: Antineoplastic | tr|A0A3Q2UBR5|A0A3Q2UBR5_CHICK | 1.516208 | up |
| Human Diseases | Drug resistance: Antineoplastic | tr|E1BTQ4|E1BTQ4_CHICK | -4.65695 | down |
| Human Diseases | Drug resistance: Antineoplastic | tr|E1BTS3|E1BTS3_CHICK | 1.090627 | up |
| Human Diseases | Drug resistance: Antineoplastic | tr|E1BX85|E1BX85_CHICK | 2.029343 | up |
| Human Diseases | Drug resistance: Antineoplastic | tr|F1NMJ9|F1NMJ9_CHICK | -2.4895 | down |
| Human Diseases | Drug resistance: Antineoplastic | tr|F1P204|F1P204_CHICK | 2.129437 | up |
| Human Diseases | Drug resistance: Antineoplastic | tr|Q5F3X1|Q5F3X1_CHICK | 1.19047 | up |
| Human Diseases | Endocrine and metabolic diseases | sp|O93602|ATF2_CHICK | 2.071233 | up |
| Human Diseases | Endocrine and metabolic diseases | tr|A0A1D5NUQ9|A0A1D5NUQ9_CHICK | 1.529462 | up |
| Human Diseases | Endocrine and metabolic diseases | tr|A0A1D5NWJ7|A0A1D5NWJ7_CHICK | 1.195763 | up |
| Human Diseases | Endocrine and metabolic diseases | tr|A0A1D5NZB4|A0A1D5NZB4_CHICK | 1.671702 | up |
| Human Diseases | Endocrine and metabolic diseases | tr|A0A1D5P4B1|A0A1D5P4B1_CHICK | 1.156059 | up |
| Human Diseases | Endocrine and metabolic diseases | tr|A0A1D5P8P6|A0A1D5P8P6_CHICK | 1.435954 | up |
| Human Diseases | Endocrine and metabolic diseases | tr|A0A1D5PCZ1|A0A1D5PCZ1_CHICK | 1.542638 | up |
| Human Diseases | Endocrine and metabolic diseases | tr|A0A1D5PXN4|A0A1D5PXN4_CHICK | -1.85723 | down |
| Human Diseases | Endocrine and metabolic diseases | tr|A0A1D6UPT6|A0A1D6UPT6_CHICK | 1.170381 | up |
| Human Diseases | Endocrine and metabolic diseases | tr|A0A1L1RSG1|A0A1L1RSG1_CHICK | 1.889712 | up |
| Human Diseases | Endocrine and metabolic diseases | tr|A0A1L1RTJ6|A0A1L1RTJ6_CHICK | 1.2568 | up |
| Human Diseases | Endocrine and metabolic diseases | tr|A0A3Q2TX28|A0A3Q2TX28_CHICK | 1.495625 | up |
| Human Diseases | Endocrine and metabolic diseases | tr|A0A3Q3AQ10|A0A3Q3AQ10_CHICK | 1.000417 | up |
| Human Diseases | Endocrine and metabolic diseases | tr|E1BT94|E1BT94_CHICK | 1.49966 | up |
| Human Diseases | Endocrine and metabolic diseases | tr|E1BXT9|E1BXT9_CHICK | 1.600608 | up |
| Human Diseases | Endocrine and metabolic diseases | tr|F1NDH2|F1NDH2_CHICK | -1.95135 | down |
| Human Diseases | Endocrine and metabolic diseases | tr|Q2PUH1|Q2PUH1_CHICK | 1.468226 | up |
| Human Diseases | Endocrine and metabolic diseases | tr|Q5F3X1|Q5F3X1_CHICK | 1.19047 | up |
| Human Diseases | Endocrine and metabolic diseases | tr|Q5ZJW2|Q5ZJW2_CHICK | 1.71086 | up |
| Human Diseases | Endocrine and metabolic diseases | tr|R4GJH2|R4GJH2_CHICK | 1.058464 | up |
| Human Diseases | Immune diseases | sp|Q9I8D0|VPP1_CHICK | 1.736096 | up |
| Human Diseases | Immune diseases | tr|A0A1D5P6C3|A0A1D5P6C3_CHICK | 2.100747 | up |
| Human Diseases | Immune diseases | tr|A0A1D5P9F9|A0A1D5P9F9_CHICK | -1.61299 | down |
| Human Diseases | Immune diseases | tr|A0A1D5PAQ0|A0A1D5PAQ0_CHICK | -4.05766 | down |
| Human Diseases | Immune diseases | tr|A0A1D5PEY8|A0A1D5PEY8_CHICK | -1.7126 | down |
| Human Diseases | Immune diseases | tr|A0A1D5PU94|A0A1D5PU94_CHICK | -1.37297 | down |
| Human Diseases | Immune diseases | tr|A0A3Q2TTN1|A0A3Q2TTN1_CHICK | -3.87735 | down |
| Human Diseases | Immune diseases | tr|A0A3Q2TX54|A0A3Q2TX54_CHICK | -4.4331 | down |
| Human Diseases | Immune diseases | tr|A0A3Q2TYZ3|A0A3Q2TYZ3_CHICK | -3.88695 | down |
| Human Diseases | Immune diseases | tr|A0A3Q2U474|A0A3Q2U474_CHICK | -3.66327 | down |
| Human Diseases | Immune diseases | tr|A0A3Q2U637|A0A3Q2U637_CHICK | -2.432 | down |
| Human Diseases | Immune diseases | tr|A0A3Q2UAA5|A0A3Q2UAA5_CHICK | -3.02904 | down |
| Human Diseases | Immune diseases | tr|A0A3Q2UAE7|A0A3Q2UAE7_CHICK | -3.38259 | down |
| Human Diseases | Immune diseases | tr|A0A3Q2UFX5|A0A3Q2UFX5_CHICK | -3.47542 | down |
| Human Diseases | Immune diseases | tr|A0A3Q2UHW8|A0A3Q2UHW8_CHICK | -4.15396 | down |
| Human Diseases | Immune diseases | tr|A0A3Q2UJK8|A0A3Q2UJK8_CHICK | -3.86779 | down |
| Human Diseases | Immune diseases | tr|A0A3Q3AMX7|A0A3Q3AMX7_CHICK | -3.6569 | down |
| Human Diseases | Immune diseases | tr|E1C6U2|E1C6U2_CHICK | -1.17102 | down |
| Human Diseases | Immune diseases | tr|E1C7C1|E1C7C1_CHICK | -1.84307 | down |
| Human Diseases | Immune diseases | tr|F1NJU5|F1NJU5_CHICK | -1.49981 | down |
| Human Diseases | Immune diseases | tr|F1NNV6|F1NNV6_CHICK | 1.809432 | up |
| Human Diseases | Immune diseases | tr|Q5ZJ19|Q5ZJ19_CHICK | 2.470646 | up |
| Human Diseases | Immune diseases | tr|Q9I8C8|Q9I8C8_CHICK | 1.2712 | up |
| Human Diseases | Infectious diseases: Bacterial | sp|P00523|SRC_CHICK | 1.599352 | up |
| Human Diseases | Infectious diseases: Bacterial | sp|P81475|CFBL_CHICK | -1.75388 | down |
| Human Diseases | Infectious diseases: Bacterial | sp|Q9I8D0|VPP1_CHICK | 1.736096 | up |
| Human Diseases | Infectious diseases: Bacterial | tr|A0A1D5NZB4|A0A1D5NZB4_CHICK | 1.671702 | up |
| Human Diseases | Infectious diseases: Bacterial | tr|A0A1D5P9F9|A0A1D5P9F9_CHICK | -1.61299 | down |
| Human Diseases | Infectious diseases: Bacterial | tr|A0A1D5PAQ0|A0A1D5PAQ0_CHICK | -4.05766 | down |
| Human Diseases | Infectious diseases: Bacterial | tr|A0A1D5PHV9|A0A1D5PHV9_CHICK | 1.32812 | up |
| Human Diseases | Infectious diseases: Bacterial | tr|A0A1D5PU94|A0A1D5PU94_CHICK | -1.37297 | down |
| Human Diseases | Infectious diseases: Bacterial | tr|A0A1L1RTJ6|A0A1L1RTJ6_CHICK | 1.2568 | up |
| Human Diseases | Infectious diseases: Bacterial | tr|A0A3Q2TRY3|A0A3Q2TRY3_CHICK | -1.3834 | down |
| Human Diseases | Infectious diseases: Bacterial | tr|A0A3Q2TTN1|A0A3Q2TTN1_CHICK | -3.87735 | down |
| Human Diseases | Infectious diseases: Bacterial | tr|A0A3Q2TVA9|A0A3Q2TVA9_CHICK | -1.89889 | down |
| Human Diseases | Infectious diseases: Bacterial | tr|A0A3Q2TX28|A0A3Q2TX28_CHICK | 1.495625 | up |
| Human Diseases | Infectious diseases: Bacterial | tr|A0A3Q2TX54|A0A3Q2TX54_CHICK | -4.4331 | down |
| Human Diseases | Infectious diseases: Bacterial | tr|A0A3Q2TYZ3|A0A3Q2TYZ3_CHICK | -3.88695 | down |
| Human Diseases | Infectious diseases: Bacterial | tr|A0A3Q2U474|A0A3Q2U474_CHICK | -3.66327 | down |
| Human Diseases | Infectious diseases: Bacterial | tr|A0A3Q2U637|A0A3Q2U637_CHICK | -2.432 | down |
| Human Diseases | Infectious diseases: Bacterial | tr|A0A3Q2UAA5|A0A3Q2UAA5_CHICK | -3.02904 | down |
| Human Diseases | Infectious diseases: Bacterial | tr|A0A3Q2UAE7|A0A3Q2UAE7_CHICK | -3.38259 | down |
| Human Diseases | Infectious diseases: Bacterial | tr|A0A3Q2UBR5|A0A3Q2UBR5_CHICK | 1.516208 | up |
| Human Diseases | Infectious diseases: Bacterial | tr|A0A3Q2UFX5|A0A3Q2UFX5_CHICK | -3.47542 | down |
| Human Diseases | Infectious diseases: Bacterial | tr|A0A3Q2UHW8|A0A3Q2UHW8_CHICK | -4.15396 | down |
| Human Diseases | Infectious diseases: Bacterial | tr|A0A3Q2UJK8|A0A3Q2UJK8_CHICK | -3.86779 | down |
| Human Diseases | Infectious diseases: Bacterial | tr|A0A3Q3AMX7|A0A3Q3AMX7_CHICK | -3.6569 | down |
| Human Diseases | Infectious diseases: Bacterial | tr|E1BTH9|E1BTH9_CHICK | 1.623876 | up |
| Human Diseases | Infectious diseases: Bacterial | tr|E1BXS1|E1BXS1_CHICK | 1.514054 | up |
| Human Diseases | Infectious diseases: Bacterial | tr|E7EC82|E7EC82_CHICK | 1.586027 | up |
| Human Diseases | Infectious diseases: Bacterial | tr|F1N9H4|F1N9H4_CHICK | 1.764159 | up |
| Human Diseases | Infectious diseases: Bacterial | tr|F1NMJ9|F1NMJ9_CHICK | -2.4895 | down |
| Human Diseases | Infectious diseases: Bacterial | tr|F1NNV6|F1NNV6_CHICK | 1.809432 | up |
| Human Diseases | Infectious diseases: Bacterial | tr|Q5F3X1|Q5F3X1_CHICK | 1.19047 | up |
| Human Diseases | Infectious diseases: Bacterial | tr|Q5ZJ19|Q5ZJ19_CHICK | 2.470646 | up |
| Human Diseases | Infectious diseases: Bacterial | tr|Q5ZMG8|Q5ZMG8_CHICK | 1.771296 | up |
| Human Diseases | Infectious diseases: Bacterial | tr|Q9I8C8|Q9I8C8_CHICK | 1.2712 | up |
| Human Diseases | Infectious diseases: Bacterial | tr|R4GMH5|R4GMH5_CHICK | -2.32193 | down |
| Human Diseases | Infectious diseases: Parasitic | tr|A0A1D5P9F9|A0A1D5P9F9_CHICK | -1.61299 | down |
| Human Diseases | Infectious diseases: Parasitic | tr|A0A1D5PAQ0|A0A1D5PAQ0_CHICK | -4.05766 | down |
| Human Diseases | Infectious diseases: Parasitic | tr|A0A1D5PCZ1|A0A1D5PCZ1_CHICK | 1.542638 | up |
| Human Diseases | Infectious diseases: Parasitic | tr|A0A1D5PEY8|A0A1D5PEY8_CHICK | -1.7126 | down |
| Human Diseases | Infectious diseases: Parasitic | tr|A0A3Q2TTN1|A0A3Q2TTN1_CHICK | -3.87735 | down |
| Human Diseases | Infectious diseases: Parasitic | tr|A0A3Q2TTT6|A0A3Q2TTT6_CHICK | 1.855872 | up |
| Human Diseases | Infectious diseases: Parasitic | tr|A0A3Q2TX54|A0A3Q2TX54_CHICK | -4.4331 | down |
| Human Diseases | Infectious diseases: Parasitic | tr|A0A3Q2TYZ3|A0A3Q2TYZ3_CHICK | -3.88695 | down |
| Human Diseases | Infectious diseases: Parasitic | tr|A0A3Q2U474|A0A3Q2U474_CHICK | -3.66327 | down |
| Human Diseases | Infectious diseases: Parasitic | tr|A0A3Q2U637|A0A3Q2U637_CHICK | -2.432 | down |
| Human Diseases | Infectious diseases: Parasitic | tr|A0A3Q2UAA5|A0A3Q2UAA5_CHICK | -3.02904 | down |
| Human Diseases | Infectious diseases: Parasitic | tr|A0A3Q2UAE7|A0A3Q2UAE7_CHICK | -3.38259 | down |
| Human Diseases | Infectious diseases: Parasitic | tr|A0A3Q2UBR5|A0A3Q2UBR5_CHICK | 1.516208 | up |
| Human Diseases | Infectious diseases: Parasitic | tr|A0A3Q2UCN1|A0A3Q2UCN1_CHICK | 1.92272 | up |
| Human Diseases | Infectious diseases: Parasitic | tr|A0A3Q2UFX5|A0A3Q2UFX5_CHICK | -3.47542 | down |
| Human Diseases | Infectious diseases: Parasitic | tr|A0A3Q2UHW8|A0A3Q2UHW8_CHICK | -4.15396 | down |
| Human Diseases | Infectious diseases: Parasitic | tr|A0A3Q2UJK8|A0A3Q2UJK8_CHICK | -3.86779 | down |
| Human Diseases | Infectious diseases: Parasitic | tr|A0A3Q3AMX7|A0A3Q3AMX7_CHICK | -3.6569 | down |
| Human Diseases | Infectious diseases: Parasitic | tr|E1BS74|E1BS74_CHICK | 3.418615 | up |
| Human Diseases | Infectious diseases: Parasitic | tr|E1C7C1|E1C7C1_CHICK | -1.84307 | down |
| Human Diseases | Infectious diseases: Parasitic | tr|F1NJU5|F1NJU5_CHICK | -1.49981 | down |
| Human Diseases | Infectious diseases: Parasitic | tr|F1NMJ9|F1NMJ9_CHICK | -2.4895 | down |
| Human Diseases | Infectious diseases: Parasitic | tr|F1NZZ2|F1NZZ2_CHICK | 1.657798 | up |
| Human Diseases | Infectious diseases: Parasitic | tr|Q5F3X1|Q5F3X1_CHICK | 1.19047 | up |
| Human Diseases | Infectious diseases: Viral | sp|O93602|ATF2_CHICK | 2.071233 | up |
| Human Diseases | Infectious diseases: Viral | sp|P00523|SRC_CHICK | 1.599352 | up |
| Human Diseases | Infectious diseases: Viral | sp|Q9I8D0|VPP1_CHICK | 1.736096 | up |
| Human Diseases | Infectious diseases: Viral | tr|A0A1D5NU19|A0A1D5NU19_CHICK | 1.251921 | up |
| Human Diseases | Infectious diseases: Viral | tr|A0A1D5NU82|A0A1D5NU82_CHICK | 1.12312 | up |
| Human Diseases | Infectious diseases: Viral | tr|A0A1D5NXR2|A0A1D5NXR2_CHICK | 1.37138 | up |
| Human Diseases | Infectious diseases: Viral | tr|A0A1D5NXS8|A0A1D5NXS8_CHICK | 1.203865 | up |
| Human Diseases | Infectious diseases: Viral | tr|A0A1D5NZB4|A0A1D5NZB4_CHICK | 1.671702 | up |
| Human Diseases | Infectious diseases: Viral | tr|A0A1D5P6C6|A0A1D5P6C6_CHICK | 1.782047 | up |
| Human Diseases | Infectious diseases: Viral | tr|A0A1D5P7W0|A0A1D5P7W0_CHICK | 1.07962 | up |
| Human Diseases | Infectious diseases: Viral | tr|A0A1D5P8H7|A0A1D5P8H7_CHICK | 1.530513 | up |
| Human Diseases | Infectious diseases: Viral | tr|A0A1D5P9B9|A0A1D5P9B9_CHICK | 1.081114 | up |
| Human Diseases | Infectious diseases: Viral | tr|A0A1D5P9F9|A0A1D5P9F9_CHICK | -1.61299 | down |
| Human Diseases | Infectious diseases: Viral | tr|A0A1D5PAM1|A0A1D5PAM1_CHICK | 2.577291 | up |
| Human Diseases | Infectious diseases: Viral | tr|A0A1D5PAQ0|A0A1D5PAQ0_CHICK | -4.05766 | down |
| Human Diseases | Infectious diseases: Viral | tr|A0A1D5PB71|A0A1D5PB71_CHICK | 1.051487 | up |
| Human Diseases | Infectious diseases: Viral | tr|A0A1D5PCZ1|A0A1D5PCZ1_CHICK | 1.542638 | up |
| Human Diseases | Infectious diseases: Viral | tr|A0A1D5PZ78|A0A1D5PZ78_CHICK | 1.936124 | up |
| Human Diseases | Infectious diseases: Viral | tr|A0A1L1RTJ6|A0A1L1RTJ6_CHICK | 1.2568 | up |
| Human Diseases | Infectious diseases: Viral | tr|A0A3Q2TTN1|A0A3Q2TTN1_CHICK | -3.87735 | down |
| Human Diseases | Infectious diseases: Viral | tr|A0A3Q2TVU0|A0A3Q2TVU0_CHICK | 1.280793 | up |
| Human Diseases | Infectious diseases: Viral | tr|A0A3Q2TX28|A0A3Q2TX28_CHICK | 1.495625 | up |
| Human Diseases | Infectious diseases: Viral | tr|A0A3Q2TX54|A0A3Q2TX54_CHICK | -4.4331 | down |
| Human Diseases | Infectious diseases: Viral | tr|A0A3Q2TYZ3|A0A3Q2TYZ3_CHICK | -3.88695 | down |
| Human Diseases | Infectious diseases: Viral | tr|A0A3Q2U474|A0A3Q2U474_CHICK | -3.66327 | down |
| Human Diseases | Infectious diseases: Viral | tr|A0A3Q2U637|A0A3Q2U637_CHICK | -2.432 | down |
| Human Diseases | Infectious diseases: Viral | tr|A0A3Q2UAA5|A0A3Q2UAA5_CHICK | -3.02904 | down |
| Human Diseases | Infectious diseases: Viral | tr|A0A3Q2UAE7|A0A3Q2UAE7_CHICK | -3.38259 | down |
| Human Diseases | Infectious diseases: Viral | tr|A0A3Q2UBR5|A0A3Q2UBR5_CHICK | 1.516208 | up |
| Human Diseases | Infectious diseases: Viral | tr|A0A3Q2UFX5|A0A3Q2UFX5_CHICK | -3.47542 | down |
| Human Diseases | Infectious diseases: Viral | tr|A0A3Q2UG55|A0A3Q2UG55_CHICK | -2.8915 | down |
| Human Diseases | Infectious diseases: Viral | tr|A0A3Q2UHW8|A0A3Q2UHW8_CHICK | -4.15396 | down |
| Human Diseases | Infectious diseases: Viral | tr|A0A3Q2UJK8|A0A3Q2UJK8_CHICK | -3.86779 | down |
| Human Diseases | Infectious diseases: Viral | tr|A0A3Q3AMX7|A0A3Q3AMX7_CHICK | -3.6569 | down |
| Human Diseases | Infectious diseases: Viral | tr|A0A3Q3AN52|A0A3Q3AN52_CHICK | 1.965147 | up |
| Human Diseases | Infectious diseases: Viral | tr|E1BR45|E1BR45_CHICK | 1.323412 | up |
| Human Diseases | Infectious diseases: Viral | tr|E1BTQ4|E1BTQ4_CHICK | -4.65695 | down |
| Human Diseases | Infectious diseases: Viral | tr|E1BX21|E1BX21_CHICK | 1.42634 | up |
| Human Diseases | Infectious diseases: Viral | tr|E1C296|E1C296_CHICK | 1.621433 | up |
| Human Diseases | Infectious diseases: Viral | tr|E1C310|E1C310_CHICK | 1.545191 | up |
| Human Diseases | Infectious diseases: Viral | tr|E1C4J0|E1C4J0_CHICK | 3.738401 | up |
| Human Diseases | Infectious diseases: Viral | tr|E1C7A7|E1C7A7_CHICK | -1.02532 | down |
| Human Diseases | Infectious diseases: Viral | tr|F1NIT0|F1NIT0_CHICK | 1.30851 | up |
| Human Diseases | Infectious diseases: Viral | tr|F1NL19|F1NL19_CHICK | 1.203656 | up |
| Human Diseases | Infectious diseases: Viral | tr|F1NMJ9|F1NMJ9_CHICK | -2.4895 | down |
| Human Diseases | Infectious diseases: Viral | tr|F1NNV6|F1NNV6_CHICK | 1.809432 | up |
| Human Diseases | Infectious diseases: Viral | tr|F1NZZ2|F1NZZ2_CHICK | 1.657798 | up |
| Human Diseases | Infectious diseases: Viral | tr|O93466|O93466_CHICK | 1.669881 | up |
| Human Diseases | Infectious diseases: Viral | tr|Q5F3X1|Q5F3X1_CHICK | 1.19047 | up |
| Human Diseases | Infectious diseases: Viral | tr|Q5ZJ19|Q5ZJ19_CHICK | 2.470646 | up |
| Human Diseases | Infectious diseases: Viral | tr|Q5ZJA8|Q5ZJA8_CHICK | 1.093474 | up |
| Human Diseases | Infectious diseases: Viral | tr|Q5ZLI0|Q5ZLI0_CHICK | 1.097879 | up |
| Human Diseases | Infectious diseases: Viral | tr|Q5ZMJ6|Q5ZMJ6_CHICK | 1.845871 | up |
| Human Diseases | Infectious diseases: Viral | tr|Q9I8C8|Q9I8C8_CHICK | 1.2712 | up |
| Human Diseases | Infectious diseases: Viral | tr|R4GMH5|R4GMH5_CHICK | -2.32193 | down |
| Human Diseases | Neurodegenerative diseases | sp|P11602|LIPL_CHICK | 1.271309 | up |
| Human Diseases | Neurodegenerative diseases | sp|P18936|NU1M_CHICK | 2.034658 | up |
| Human Diseases | Neurodegenerative diseases | tr|A0A1D5NWJ7|A0A1D5NWJ7_CHICK | 1.195763 | up |
| Human Diseases | Neurodegenerative diseases | tr|A0A1D5P4B1|A0A1D5P4B1_CHICK | 1.156059 | up |
| Human Diseases | Neurodegenerative diseases | tr|A0A1D5P7C5|A0A1D5P7C5_CHICK | 1.273318 | up |
| Human Diseases | Neurodegenerative diseases | tr|A0A1D5PEY8|A0A1D5PEY8_CHICK | -1.7126 | down |
| Human Diseases | Neurodegenerative diseases | tr|A0A1D5PQQ6|A0A1D5PQQ6_CHICK | 1.432587 | up |
| Human Diseases | Neurodegenerative diseases | tr|A0A1D6UPT6|A0A1D6UPT6_CHICK | 1.170381 | up |
| Human Diseases | Neurodegenerative diseases | tr|A0A1L1RSG1|A0A1L1RSG1_CHICK | 1.889712 | up |
| Human Diseases | Neurodegenerative diseases | tr|A0A3Q2U3K8|A0A3Q2U3K8_CHICK | 1.162767 | up |
| Human Diseases | Neurodegenerative diseases | tr|E1BS74|E1BS74_CHICK | 3.418615 | up |
| Human Diseases | Neurodegenerative diseases | tr|E1BT94|E1BT94_CHICK | 1.49966 | up |
| Human Diseases | Neurodegenerative diseases | tr|E1BTQ4|E1BTQ4_CHICK | -4.65695 | down |
| Human Diseases | Neurodegenerative diseases | tr|E1BV97|E1BV97_CHICK | 2.036483 | up |
| Human Diseases | Neurodegenerative diseases | tr|E1BX21|E1BX21_CHICK | 1.42634 | up |
| Human Diseases | Neurodegenerative diseases | tr|E1BXT9|E1BXT9_CHICK | 1.600608 | up |
| Human Diseases | Neurodegenerative diseases | tr|E1C6U2|E1C6U2_CHICK | -1.17102 | down |
| Human Diseases | Neurodegenerative diseases | tr|E1C7C1|E1C7C1_CHICK | -1.84307 | down |
| Human Diseases | Neurodegenerative diseases | tr|F1NJU5|F1NJU5_CHICK | -1.49981 | down |
| Human Diseases | Neurodegenerative diseases | tr|O42417|O42417_CHICK | 1.118629 | up |
| Human Diseases | Neurodegenerative diseases | tr|Q5ZJW2|Q5ZJW2_CHICK | 1.71086 | up |
| Human Diseases | Neurodegenerative diseases | tr|Q5ZMJ6|Q5ZMJ6_CHICK | 1.845871 | up |
| Human Diseases | Neurodegenerative diseases | tr|R4GJH2|R4GJH2_CHICK | 1.058464 | up |
| Human Diseases | Neurodegenerative diseases | tr|V9GW30|V9GW30_CHICK | 1.560922 | up |
| Human Diseases | Substance dependence | sp|O93602|ATF2_CHICK | 2.071233 | up |
| Human Diseases | Substance dependence | tr|A0A1D5NUQ9|A0A1D5NUQ9_CHICK | 1.529462 | up |
| Human Diseases | Substance dependence | tr|A0A1D5NUU0|A0A1D5NUU0_CHICK | 1.000304 | up |
| Human Diseases | Substance dependence | tr|A0A1D5PCZ1|A0A1D5PCZ1_CHICK | 1.542638 | up |
| Human Diseases | Substance dependence | tr|A0A1L1RTJ6|A0A1L1RTJ6_CHICK | 1.2568 | up |
| Human Diseases | Substance dependence | tr|A0A1L1RWI9|A0A1L1RWI9_CHICK | 1.991255 | up |
| Human Diseases | Substance dependence | tr|A0A1L1RXZ8|A0A1L1RXZ8_CHICK | 2.28751 | up |
| Human Diseases | Substance dependence | tr|F1P5A3|F1P5A3_CHICK | 2.127136 | up |
| Human Diseases | Substance dependence | tr|Q5F3X1|Q5F3X1_CHICK | 1.19047 | up |
| Metabolism | Amino acid metabolism | sp|P00337|LDHB_CHICK | 2.131478 | up |
| Metabolism | Amino acid metabolism | sp|P21265|PUR8_CHICK | 1.926426 | up |
| Metabolism | Amino acid metabolism | sp|Q8AXL1|SAT1_CHICK | 1.12819 | up |
| Metabolism | Amino acid metabolism | tr|A0A1D5NTU3|A0A1D5NTU3_CHICK | 1.538114 | up |
| Metabolism | Amino acid metabolism | tr|A0A1D5NUQ9|A0A1D5NUQ9_CHICK | 1.529462 | up |
| Metabolism | Amino acid metabolism | tr|A0A1D5P3Q3|A0A1D5P3Q3_CHICK | 2.685378 | up |
| Metabolism | Amino acid metabolism | tr|A0A1D5P6M8|A0A1D5P6M8_CHICK | 1.82889 | up |
| Metabolism | Amino acid metabolism | tr|A0A1D5PD17|A0A1D5PD17_CHICK | 1.860269 | up |
| Metabolism | Amino acid metabolism | tr|A0A1D5PF77|A0A1D5PF77_CHICK | 1.48755 | up |
| Metabolism | Amino acid metabolism | tr|E1BQW2|E1BQW2_CHICK | 1.823966 | up |
| Metabolism | Amino acid metabolism | tr|E1BTT8|E1BTT8_CHICK | 1.708105 | up |
| Metabolism | Amino acid metabolism | tr|E1BY22|E1BY22_CHICK | 1.367672 | up |
| Metabolism | Amino acid metabolism | tr|E1C9H5|E1C9H5_CHICK | 1.195027 | up |
| Metabolism | Amino acid metabolism | tr|F1NXZ7|F1NXZ7_CHICK | 1.050248 | up |
| Metabolism | Amino acid metabolism | tr|F1P531|F1P531_CHICK | 1.370667 | up |
| Metabolism | Biosynthesis of other secondary metabolites | tr|F1N977|F1N977_CHICK | 1.720618 | up |
| Metabolism | Carbohydrate metabolism | sp|P00337|LDHB_CHICK | 2.131478 | up |
| Metabolism | Carbohydrate metabolism | sp|P00940|TPIS_CHICK | 2.162937 | up |
| Metabolism | Carbohydrate metabolism | sp|Q5F480|ITPK1_CHICK | 1.066359 | up |
| Metabolism | Carbohydrate metabolism | sp|Q5ZIK0|P4K2B_CHICK | 2.036191 | up |
| Metabolism | Carbohydrate metabolism | tr|A0A1D5NTT2|A0A1D5NTT2_CHICK | 2.201769 | up |
| Metabolism | Carbohydrate metabolism | tr|A0A1D5NUD3|A0A1D5NUD3_CHICK | 1.402549 | up |
| Metabolism | Carbohydrate metabolism | tr|A0A1D5NUQ9|A0A1D5NUQ9_CHICK | 1.529462 | up |
| Metabolism | Carbohydrate metabolism | tr|A0A1D5NV81|A0A1D5NV81_CHICK | 1.935393 | up |
| Metabolism | Carbohydrate metabolism | tr|A0A1D5PC23|A0A1D5PC23_CHICK | 2.344267 | up |
| Metabolism | Carbohydrate metabolism | tr|A0A1D5PDW6|A0A1D5PDW6_CHICK | 1.010784 | up |
| Metabolism | Carbohydrate metabolism | tr|A0A1D5PG53|A0A1D5PG53_CHICK | 2.149242 | up |
| Metabolism | Carbohydrate metabolism | tr|A0A1D5PHE2|A0A1D5PHE2_CHICK | 2.577604 | up |
| Metabolism | Carbohydrate metabolism | tr|A0A1D5PJL7|A0A1D5PJL7_CHICK | 1.22028 | up |
| Metabolism | Carbohydrate metabolism | tr|A0A1D5Q008|A0A1D5Q008_CHICK | 2.962255 | up |
| Metabolism | Carbohydrate metabolism | tr|A0A3Q2TRS4|A0A3Q2TRS4_CHICK | 1.770416 | up |
| Metabolism | Carbohydrate metabolism | tr|A0A3Q2TX28|A0A3Q2TX28_CHICK | 1.495625 | up |
| Metabolism | Carbohydrate metabolism | tr|A0A3Q2U3C4|A0A3Q2U3C4_CHICK | 1.094483 | up |
| Metabolism | Carbohydrate metabolism | tr|A0A3Q2UCG6|A0A3Q2UCG6_CHICK | 1.033099 | up |
| Metabolism | Carbohydrate metabolism | tr|E1BQU2|E1BQU2_CHICK | 1.629018 | up |
| Metabolism | Carbohydrate metabolism | tr|E1BT44|E1BT44_CHICK | 1.432225 | up |
| Metabolism | Carbohydrate metabolism | tr|E1BTT8|E1BTT8_CHICK | 1.708105 | up |
| Metabolism | Carbohydrate metabolism | tr|E1BTW4|E1BTW4_CHICK | 2.41163 | up |
| Metabolism | Carbohydrate metabolism | tr|E1BVD1|E1BVD1_CHICK | 3.053645 | up |
| Metabolism | Carbohydrate metabolism | tr|E1BY22|E1BY22_CHICK | 1.367672 | up |
| Metabolism | Carbohydrate metabolism | tr|E1C1I6|E1C1I6_CHICK | 2.427937 | up |
| Metabolism | Carbohydrate metabolism | tr|F1NBX1|F1NBX1_CHICK | 1.439614 | up |
| Metabolism | Carbohydrate metabolism | tr|F1NN63|F1NN63_CHICK | 1.72615 | up |
| Metabolism | Carbohydrate metabolism | tr|F1NT57|F1NT57_CHICK | 2.838862 | up |
| Metabolism | Carbohydrate metabolism | tr|F1NTQ2|F1NTQ2_CHICK | 1.236275 | up |
| Metabolism | Carbohydrate metabolism | tr|F1P581|F1P581_CHICK | 1.52511 | up |
| Metabolism | Carbohydrate metabolism | tr|F1P5S5|F1P5S5_CHICK | 1.528046 | up |
| Metabolism | Carbohydrate metabolism | tr|Q90W83|Q90W83_CHICK | 2.57878 | up |
| Metabolism | Carbohydrate metabolism | tr|R4GG24|R4GG24_CHICK | 3.018466 | up |
| Metabolism | Energy metabolism | sp|P18936|NU1M_CHICK | 2.034658 | up |
| Metabolism | Energy metabolism | sp|Q9I8D0|VPP1_CHICK | 1.736096 | up |
| Metabolism | Energy metabolism | tr|A0A1D5NV81|A0A1D5NV81_CHICK | 1.935393 | up |
| Metabolism | Energy metabolism | tr|A0A1D5NWJ7|A0A1D5NWJ7_CHICK | 1.195763 | up |
| Metabolism | Energy metabolism | tr|A0A1D5P4B1|A0A1D5P4B1_CHICK | 1.156059 | up |
| Metabolism | Energy metabolism | tr|A0A1D6UPT6|A0A1D6UPT6_CHICK | 1.170381 | up |
| Metabolism | Energy metabolism | tr|A0A1L1RSG1|A0A1L1RSG1_CHICK | 1.889712 | up |
| Metabolism | Energy metabolism | tr|E1BT94|E1BT94_CHICK | 1.49966 | up |
| Metabolism | Energy metabolism | tr|E1BXT9|E1BXT9_CHICK | 1.600608 | up |
| Metabolism | Energy metabolism | tr|F1NMU0|F1NMU0_CHICK | 2.467729 | up |
| Metabolism | Energy metabolism | tr|F1NNV6|F1NNV6_CHICK | 1.809432 | up |
| Metabolism | Energy metabolism | tr|F1P2D2|F1P2D2_CHICK | 1.35416 | up |
| Metabolism | Energy metabolism | tr|Q5ZJ19|Q5ZJ19_CHICK | 2.470646 | up |
| Metabolism | Energy metabolism | tr|Q5ZJW2|Q5ZJW2_CHICK | 1.71086 | up |
| Metabolism | Energy metabolism | tr|Q9I8C8|Q9I8C8_CHICK | 1.2712 | up |
| Metabolism | Energy metabolism | tr|R4GJH2|R4GJH2_CHICK | 1.058464 | up |
| Metabolism | Global and overview maps | sp|P00337|LDHB_CHICK | 2.131478 | up |
| Metabolism | Global and overview maps | sp|P00940|TPIS_CHICK | 2.162937 | up |
| Metabolism | Global and overview maps | sp|P18936|NU1M_CHICK | 2.034658 | up |
| Metabolism | Global and overview maps | sp|P21265|PUR8_CHICK | 1.926426 | up |
| Metabolism | Global and overview maps | sp|Q5F480|ITPK1_CHICK | 1.066359 | up |
| Metabolism | Global and overview maps | sp|Q5ZIK0|P4K2B_CHICK | 2.036191 | up |
| Metabolism | Global and overview maps | sp|Q5ZJ66|SYEM_CHICK | 1.236071 | up |
| Metabolism | Global and overview maps | sp|Q8AXL1|SAT1_CHICK | 1.12819 | up |
| Metabolism | Global and overview maps | sp|Q9I8D0|VPP1_CHICK | 1.736096 | up |
| Metabolism | Global and overview maps | tr|A0A1D5NU78|A0A1D5NU78_CHICK | 1.139159 | up |
| Metabolism | Global and overview maps | tr|A0A1D5NUD3|A0A1D5NUD3_CHICK | 1.402549 | up |
| Metabolism | Global and overview maps | tr|A0A1D5NUK5|A0A1D5NUK5_CHICK | 2.534566 | up |
| Metabolism | Global and overview maps | tr|A0A1D5NUQ9|A0A1D5NUQ9_CHICK | 1.529462 | up |
| Metabolism | Global and overview maps | tr|A0A1D5NV81|A0A1D5NV81_CHICK | 1.935393 | up |
| Metabolism | Global and overview maps | tr|A0A1D5P3Q3|A0A1D5P3Q3_CHICK | 2.685378 | up |
| Metabolism | Global and overview maps | tr|A0A1D5P4B1|A0A1D5P4B1_CHICK | 1.156059 | up |
| Metabolism | Global and overview maps | tr|A0A1D5PC23|A0A1D5PC23_CHICK | 2.344267 | up |
| Metabolism | Global and overview maps | tr|A0A1D5PD08|A0A1D5PD08_CHICK | 2.003715 | up |
| Metabolism | Global and overview maps | tr|A0A1D5PD17|A0A1D5PD17_CHICK | 1.860269 | up |
| Metabolism | Global and overview maps | tr|A0A1D5PDP1|A0A1D5PDP1_CHICK | 1.270345 | up |
| Metabolism | Global and overview maps | tr|A0A1D5PDW6|A0A1D5PDW6_CHICK | 1.010784 | up |
| Metabolism | Global and overview maps | tr|A0A1D5PE83|A0A1D5PE83_CHICK | 1.167073 | up |
| Metabolism | Global and overview maps | tr|A0A1D5PF77|A0A1D5PF77_CHICK | 1.48755 | up |
| Metabolism | Global and overview maps | tr|A0A1D5PF81|A0A1D5PF81_CHICK | 1.604859 | up |
| Metabolism | Global and overview maps | tr|A0A1D5PG53|A0A1D5PG53_CHICK | 2.149242 | up |
| Metabolism | Global and overview maps | tr|A0A1D5PHE2|A0A1D5PHE2_CHICK | 2.577604 | up |
| Metabolism | Global and overview maps | tr|A0A1D5PHH6|A0A1D5PHH6_CHICK | -2.20613 | down |
| Metabolism | Global and overview maps | tr|A0A1D5PIT4|A0A1D5PIT4_CHICK | -1.97988 | down |
| Metabolism | Global and overview maps | tr|A0A1D5PJL7|A0A1D5PJL7_CHICK | 1.22028 | up |
| Metabolism | Global and overview maps | tr|A0A1D5PM44|A0A1D5PM44_CHICK | 1.054523 | up |
| Metabolism | Global and overview maps | tr|A0A1D5PTW3|A0A1D5PTW3_CHICK | 1.808599 | up |
| Metabolism | Global and overview maps | tr|A0A1D5PU00|A0A1D5PU00_CHICK | -2.66405 | down |
| Metabolism | Global and overview maps | tr|A0A1D5PWJ2|A0A1D5PWJ2_CHICK | 1.548676 | up |
| Metabolism | Global and overview maps | tr|A0A1D5Q008|A0A1D5Q008_CHICK | 2.962255 | up |
| Metabolism | Global and overview maps | tr|A0A1D6UPT6|A0A1D6UPT6_CHICK | 1.170381 | up |
| Metabolism | Global and overview maps | tr|A0A1L1RJB1|A0A1L1RJB1_CHICK | 1.578007 | up |
| Metabolism | Global and overview maps | tr|A0A1L1RPR1|A0A1L1RPR1_CHICK | 1.93845 | up |
| Metabolism | Global and overview maps | tr|A0A1L1RSG1|A0A1L1RSG1_CHICK | 1.889712 | up |
| Metabolism | Global and overview maps | tr|A0A1L1RSS5|A0A1L1RSS5_CHICK | 1.143155 | up |
| Metabolism | Global and overview maps | tr|A0A1L1RUR2|A0A1L1RUR2_CHICK | 1.699404 | up |
| Metabolism | Global and overview maps | tr|A0A2Z5EM90|A0A2Z5EM90_CHICK | 1.136685 | up |
| Metabolism | Global and overview maps | tr|A0A3Q2TRS4|A0A3Q2TRS4_CHICK | 1.770416 | up |
| Metabolism | Global and overview maps | tr|A0A3Q2TS24|A0A3Q2TS24_CHICK | 1.398629 | up |
| Metabolism | Global and overview maps | tr|A0A3Q2TX28|A0A3Q2TX28_CHICK | 1.495625 | up |
| Metabolism | Global and overview maps | tr|A0A3Q2U3C4|A0A3Q2U3C4_CHICK | 1.094483 | up |
| Metabolism | Global and overview maps | tr|A0A3Q2U942|A0A3Q2U942_CHICK | 2.018437 | up |
| Metabolism | Global and overview maps | tr|A0A3Q2UAS1|A0A3Q2UAS1_CHICK | 1.28598 | up |
| Metabolism | Global and overview maps | tr|A0A3Q2UK74|A0A3Q2UK74_CHICK | 2.254776 | up |
| Metabolism | Global and overview maps | tr|A0A3Q3AG70|A0A3Q3AG70_CHICK | -3.5746 | down |
| Metabolism | Global and overview maps | tr|A0A3Q3APG2|A0A3Q3APG2_CHICK | 1.640987 | up |
| Metabolism | Global and overview maps | tr|A0A3S5ZP86|A0A3S5ZP86_CHICK | 1.83944 | up |
| Metabolism | Global and overview maps | tr|A0A5P9VLA0|A0A5P9VLA0_CHICK | 1.601588 | up |
| Metabolism | Global and overview maps | tr|E1BQI0|E1BQI0_CHICK | 1.067708 | up |
| Metabolism | Global and overview maps | tr|E1BQU2|E1BQU2_CHICK | 1.629018 | up |
| Metabolism | Global and overview maps | tr|E1BQW2|E1BQW2_CHICK | 1.823966 | up |
| Metabolism | Global and overview maps | tr|E1BT44|E1BT44_CHICK | 1.432225 | up |
| Metabolism | Global and overview maps | tr|E1BT94|E1BT94_CHICK | 1.49966 | up |
| Metabolism | Global and overview maps | tr|E1BTF7|E1BTF7_CHICK | 1.810838 | up |
| Metabolism | Global and overview maps | tr|E1BTS3|E1BTS3_CHICK | 1.090627 | up |
| Metabolism | Global and overview maps | tr|E1BTT8|E1BTT8_CHICK | 1.708105 | up |
| Metabolism | Global and overview maps | tr|E1BTW4|E1BTW4_CHICK | 2.41163 | up |
| Metabolism | Global and overview maps | tr|E1BU27|E1BU27_CHICK | 3.035848 | up |
| Metabolism | Global and overview maps | tr|E1BVD1|E1BVD1_CHICK | 3.053645 | up |
| Metabolism | Global and overview maps | tr|E1BVK8|E1BVK8_CHICK | 1.186519 | up |
| Metabolism | Global and overview maps | tr|E1BXC2|E1BXC2_CHICK | 1.767478 | up |
| Metabolism | Global and overview maps | tr|E1BXT9|E1BXT9_CHICK | 1.600608 | up |
| Metabolism | Global and overview maps | tr|E1BY22|E1BY22_CHICK | 1.367672 | up |
| Metabolism | Global and overview maps | tr|E1BYU3|E1BYU3_CHICK | 1.170333 | up |
| Metabolism | Global and overview maps | tr|E1BZ79|E1BZ79_CHICK | 2.388844 | up |
| Metabolism | Global and overview maps | tr|E1C1I6|E1C1I6_CHICK | 2.427937 | up |
| Metabolism | Global and overview maps | tr|E1C1T2|E1C1T2_CHICK | 1.594659 | up |
| Metabolism | Global and overview maps | tr|E1C2E5|E1C2E5_CHICK | 1.182299 | up |
| Metabolism | Global and overview maps | tr|E1C483|E1C483_CHICK | 1.301431 | up |
| Metabolism | Global and overview maps | tr|E1C592|E1C592_CHICK | 1.565005 | up |
| Metabolism | Global and overview maps | tr|E1C9H5|E1C9H5_CHICK | 1.195027 | up |
| Metabolism | Global and overview maps | tr|F1N8Y3|F1N8Y3_CHICK | 2.225925 | up |
[truncated: 52,266 more chars]
